# Synthesis of (*Z*)-3-[amino(phenyl)methylidene]-1,3-dihydro-2*H*-indol-2-ones using an Eschenmoser coupling reaction

**DOI:** 10.3762/bjoc.17.47

**Published:** 2021-02-23

**Authors:** Lukáš Marek, Lukáš Kolman, Jiří Váňa, Jan Svoboda, Jiří Hanusek

**Affiliations:** 1 Institute of Organic Chemistry and Technology, Faculty of Chemical Technology, University of Pardubice, Studentská 573, CZ532 10 Pardubice, Czech Republic

**Keywords:** 3-bromooxindoles, Eschenmoser coupling reaction, thioamides, tyrosin kinase inhibitors, (*Z*)-3-[amino(phenyl)methylidene]-1,3-dihydro-2*H*-indol-2-ones

## Abstract

A highly modular method for the synthesis of (*Z*)-3-[amino(phenyl/methyl)methylidene]-1,3-dihydro-2*H*-indol-2-ones starting from easily available 3-bromooxindoles or (2-oxoindolin-3-yl)triflate and thioacetamides or thiobenzamides is described. A series of 49 compounds, several of which have previously been shown to possess significant tyrosin kinase inhibiting activity, was prepared in yields varying mostly from 70 to 97% and always surpassing those obtained by other published methods. The method includes an Eschenmoser coupling reaction, which is very feasible (even without using a thiophile except tertiary amides) and scalable. The (*Z*)-configuration of all products was confirmed by NMR techniques.

## Introduction

**Figure 1:**
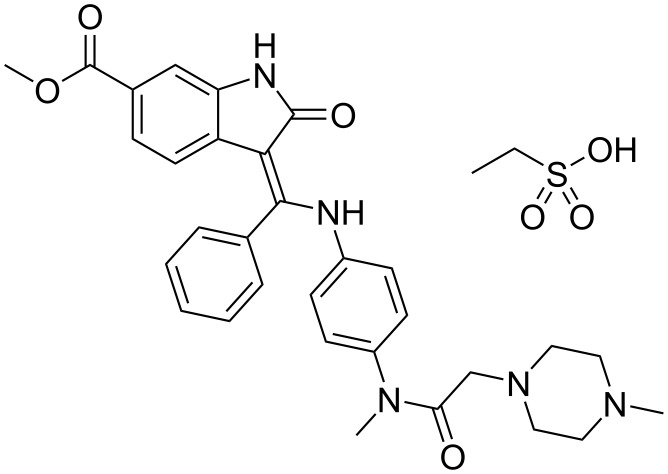
Nintedanib ethanesulfonate.

3-(Aminomethylidene)-1,3-dihydro-2*H*-indol-2-ones (3-(aminomethylidene)oxindoles) belong to a subclass of intensively studied heterocyclic compounds, especially due to their significant pharmacological activity. As early as in the middle of the 1980s researchers at Pfizer Inc. patented [1] these compounds as highly potent gabaergic agents, having possible therapeutic utility as anticonvulsants or anxiolytics [2]. However, the main therapeutic potential of these compounds was discovered ca 15 years later when several patents appeared claiming their inhibiting effect on various kinases [3–4]. Since that time many patents as well as papers have been published [5–11] on that topic but until present only Nintedanib (Figure 1) was approved [12–13] for the treatment of idiopathic pulmonary fibrosis in 2014.

**Scheme 1:**
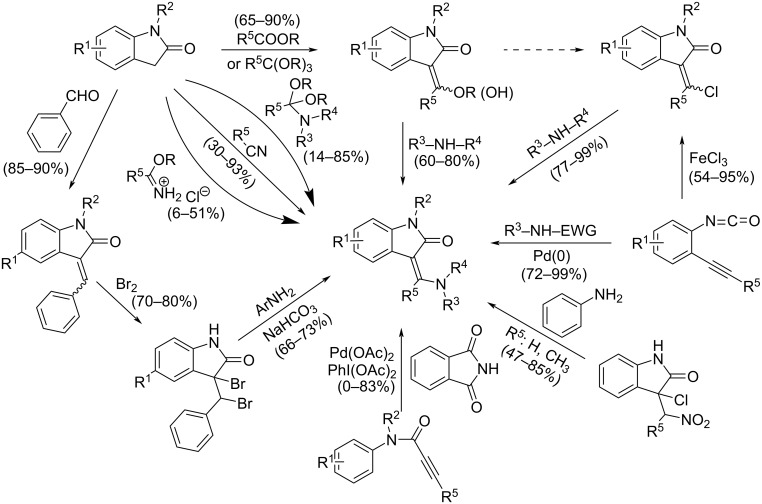
The known synthetic strategies leading to 3-(aminomethylidene)oxindoles.

The title compounds can be prepared using several synthetic approaches (Scheme 1) starting either from 1,3-dihydro-2*H*-indol-2-ones (oxindoles) and nitriles, amides, amide acetals, and imidoesters [1–3,5,11,14] or from their independently prepared 3-chloromethylidene- [4,9,15–17], 3-hydroxymethylidene- or 3-(alkoxymethylidene)oxindoles [2,5–6,18–20] and the appropriate amines. Recently, original methods involving the construction of the oxindole skeleton were also described starting from 2-alkynylphenyl isocyanates and their precursors [21–23], or from *N*-phenylpropiolamides [24–26]. The newest method of synthesis, starting from 3-bromo-3-[bromo(phenyl)methyl]oxindole and substituted anilines [27], is similar to a long-known preparation utilizing 3-chloro-3-(nitroalkyl)oxindoles and amines [28].

Except these synthetically valuable methods, several notes concerning the formation of 3-(aminomethylidene)-1,3-dihydro-2*H*-indol-2-ones under different conditions can be found in the literature [29–31].

**Scheme 2:**
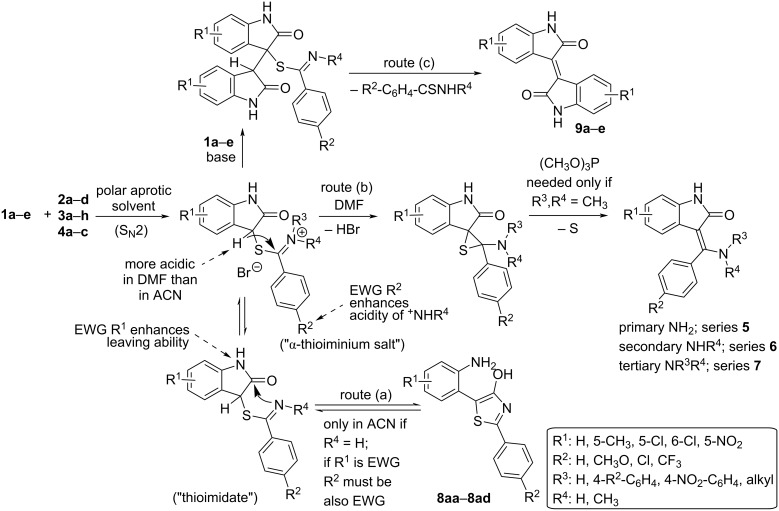
The possible intermediates and products occurring in the reactions of 3-bromooxindoles with thiobenzamides.

In our group we recently discovered [32–33] a new synthetic pathway involving a rearrangement of 2-aryl-5-(2-aminophenyl)-4-hydroxy-1,3-thiazoles (e.g., **8aa–ad** in Scheme 2) leading to the 3-[amino(aryl)methylidene]-1,3-dihydro-2*H*-indol-2-ones containing an unsubstituted amino group and the oxindole nucleus. In the present paper, we want to describe an optimized procedure avoiding the intermediary thiazoles and to demonstrate the usefulness and wide group tolerability of our new synthetic approach for the preparation of a library of substituted phenyl and amino derivatives (Table 1). A number of the compounds that have been prepared using this route (namely **5ac’**, **5ca**, **5cc’**, **5da**, **5db**, and **5dc’**) have significant tyrosin kinase inhibiting properties [11].

**Table 1:** A survey and labeling of synthesized 3-[amino(phenyl)methylidene]-1,3-dihydro-2*H*-indol-2-ones.^a^

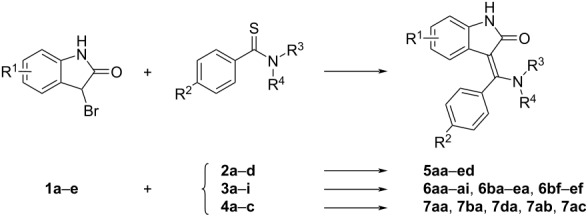											

entry	**1**	R^1^	**2**	R^2^	R^3^ = R^4^	**3**	R^2^ = R^3^	R^4^	**4**	R^2^	R^3^ = R^4^

1	**a**	H	**a**	H	H	**a**	H	C_6_H_5_	**a**	H	CH_3_
2	**b**	5-CH_3_	**b**	OCH_3_	H	**b**	H	4-OCH_3_-C_6_H_4_	**b**	OCH_3_	CH_3_
3	**c**	5-Cl	**c**	Cl	H	**c**	H	4-Cl-C_6_H_4_	**c**	Cl	CH_3_
4	**d**	6-Cl	**c’**	I	H	**d**	H	4-CF_3_-C_6_H_4_			
5	**e**	5-NO_2_	**d**	CF_3_	H	**e**	H	4-NO_2_-C_6_H_4_			
6						**f**	H	CH_3_			
7						**g**	H	CH_3_(CH_2_)_4_			
8						**h**	H	C_6_H_5_CH_2_			
9						**i**	H	cyclohexyl			

^a^Note: in all shown formulas of products the first letter relates to the substitution of compound **1**, whereas the second letter relates to a substitution of **2**–**4** (e.g., **7ba** means combination of **1b** + **4a**).

## Results and Discussion

In our previous paper [33] we reported the formation of 3-[amino(aryl)methylidene]-1,3-dihydro-2*H*-indol-2-ones (R^1^, R^3^, R^4^: H) through the intermediary (and in most cases isolable) 2-aryl-5-(2-aminophenyl)-4-hydroxy-1,3-thiazoles **8aa**–**ad** formed from 3-bromooxindole (**1a**) and various substituted primary aromatic thioamides (e.g., thiobenzamides **2a**–**d**) in acetonitrile (Scheme 2). Although the overall yields of such a two-step transformation was acceptable (around 70–80%), later we have found that a further substitution of the 3-bromooxindole (**1a**), especially with electron-withdrawing groups (R^1^ is EWG), causes either an exclusive formation of the corresponding 2-aryl-5-(2-aminophenyl)-4-hydroxy-1,3-thiazoles (when starting from **2c**,**d**) or leads to a complex and inseparable mixture of products (when starting from **2a**,**b**). The addition of a base (e.g., triethylamine, *N*-methylmorpholine, ammonia) which was originally found to be beneficial [33] for the rearrangement of the kinetically formed thiazole to the desired product now caused a complete decomposition giving mainly the isoindigo derivatives **9a–e**. Similar issues were observed when the secondary or tertiary thiobenzamides **3a**–**i** or **4a**–**c** were used. The following Scheme 2 summarizes the main possible reaction routes starting from 3-bromooxindoles **1a**–**e** and various primary, secondary, and tertiary thiobenzamides **2a**–**d**, **3a**–**i**, and **4a**–**c**.

The formation of the different products can be explained as follows. In acetonitrile, that was used in our previous study [33], the C^3^ hydrogen of the initially formed α-thioiminium salt is not acidic enough to form the carbanion that is necessary for the desired Eschenmoser coupling reaction (route b). Therefore, the nucleophilicity of the conjugated base of the nitrogen (benzenecarbimidothioate or thioimidate) is exerted towards the oxindole carbonyl to give the thiazole. Moreover, if both benzene rings contain electron-withdrawing groups, enhancing either the leaving ability of the oxindole nitrogen (R^1^: Cl, NO_2_) or the acidity of the >C=NH_2_^+^ group (R^2^: Cl, CF_3_) increasing the proportion of the nucleophile >C=NH, then the formation of the thiazole (route a) is further accelerated (Scheme 2). Although the addition of a base generates the required C^3^ carbanion, such a base also converts the reactive α-thioiminium group to a much less reactive thioimidate at the same time. The C^3^ carbanion then preferentially attacks (route c) another molecule of the starting material **1a**–**e** and the resulting adducts undergo a subsequent elimination to give the isoindigo derivatives **9a**–**e**. The substituent R^4^ strongly influences both the equilibrium between >C=N^+^HR^4^ and >C=NR^4^ as well as the nucleophilic attack of the internal C^3^ carbanion to give the thiirane intermediate in Scheme 2. While the EWG groups decrease the concentration of the α-thioiminium salt (that is much more prone to a nucleophilic addition than the free thioimidate group) they also make the α-thioiminium/thioimidate group more reactive. These two effects are antagonistic and therefore a thorough study of the R^4^ substituent influence is necessary. On the other hand, mono- as well as disubstitution of the thioamide nitrogen completely prevents the formation of the thiazole (route a).

The rather complicated product isolation procedure of the Eschenmoser coupling reaction (route b) and the long reaction time taken together with problems connected with changing substituents throughout the molecule, encouraged us to find better or even universal reaction conditions compatible with all substituents present in both benzene rings (R^1^ and R^2^) as well as at the thioamide nitrogen (R^3^ and R^4^).

In order to achieve the desired product of the Eschenmoser coupling reaction (route b), two main prerequisites must be fulfilled – i.e., the substantial acidity of a hydrogen at C^3^ and a more stable α-thioiminium group necessary for the internal nucleophilic addition giving the thiirane intermediate (route b). It is well known that the acidity of carbon acids in acetonitrile is much lower than in polar aprotic solvents, like DMSO or DMF or in polar protic solvents like alcohols. For carbon acids the Δp*K*_a_ is 12.9 between ACN and DMSO but the Δp*K*_a_ is only −1.5 between DMF and DMSO [34]. A change of the solvent from acetonitrile to DMF should therefore decrease the p*K*_a_ by ca. 11 units. In polar protic solvents (e.g., in methanol) such an increase of acidity should be also distinct but it is known that polar protic solvents much better solvate anions and thus decrease their nucleophilicity. Therefore, dimethylformamide has been chosen as the best solvent.

Indeed, when dimethylformamide (DMF) was used as the solvent for the reaction of the substituted 3-bromooxindoles **1a**–**e** with various aromatic primary (**2a**–**d**) and secondary (**3a**–**i**) thiobenzamides, the corresponding 3-[amino(phenyl)methylidene]oxindoles (series **5** and **6**) were obtained in good yields mostly exceeding 70% without the occurrence of intermediary thiazoles (route a). Only with a few secondary thiobenzamides (**3c**–**e**) or the tertiary thiobenzamides **4b**,**c**, the yields were lower.

### Reaction of **1a–e** with primary thiobenzamides **2a–d** (R^3^, R^4^: H)

**Figure 2:**
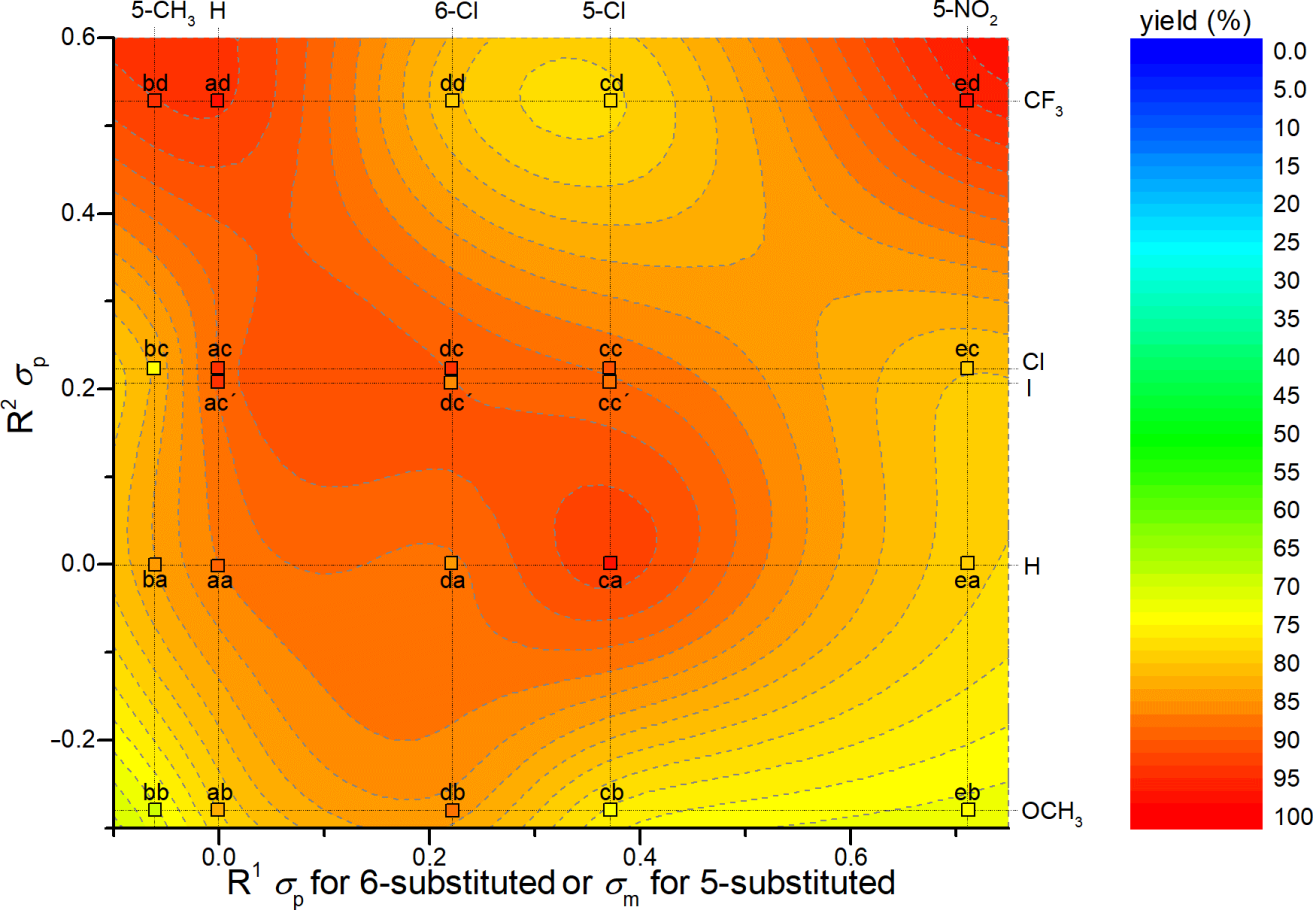
The R^1^ and R^2^ substitution influence on the isolated yields of products **5aa**–**ed**.

All possible combinations of the 3-bromooxindoles **1a**–**e** with the primary thiobenzamides **2a**–**d** were studied in order to test the R^1^ and R^2^ substituent tolerance. Moreover, in order to demonstrate the application potential of our method, a few more derivatives containing R^2^: I instead of Cl (**5ac’**, **5cc’**, **5dc’**) which display a significant kinase inhibition activity, have been prepared. It is worth noting that the existing literature synthetic pathway [11] gave only poor yields of these desirable compounds. From the data presented in Table 2, it is clear that the yields obtained in DMF are mostly very good and always much better than the previously reported yields by us in ACN [33] (see Table 2, entries 1–4) or by other authors starting from iminoesters [11] (see Table 2, entries 9, 13, 14, and 21–23). However, the inspection of Table 2 does not show any clear dependence between the electronic properties of the substituent R^1^ or R^2^ and the isolated yield. In most cases the yields are higher than 80%, but it seems that the presence of at least one EWG often gives an enhanced yield above 90% (cf. entries 3, 4, 8, 9, 11, 15, 20, and 21 in Table 2). The influence of a single substituent R^1^ is weaker (e.g., entries 1, 5, 13, and 17 in Table 2) because the electron-withdrawing groups enhance the C^3^–H acidity and an EDG enhances the C^3^ carbanion nucleophilicity and these two effects are opposing. The influence of the substituent effects, as quantified using Hammett’s substituent constants [35], on the yields can be represented graphically (Figure 2).

**Table 2:** The prepared 3-[amino(phenyl)methylidene]-1,3-dihydro-2*H*-indol-2-ones **5aa–ed** with a primary amino group (R^3^ and R^4^: H).

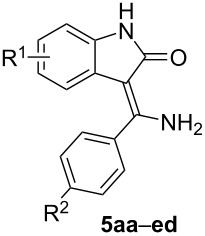								

entry	product	R^1^	R^2^	*n*_1a–e_/*n*_2a–d_ (mmol)	*m*_1a–e_ (mg)	*m*_2a–d_ (mg)	yield **5aa**–**ed** (mg)/(%)	mp (°C)

1	**5aa**	H	H	3.3/3	700	412	623/(88, 69^a^, 84^b^, 70^c^)	239–240
2	**5ab**	H	OCH_3_	3.3/3	700	502	655/(82, 73^a^, 10^d^)	229.5–231
3	**5ac**	H	Cl	3.3/3	700	515	754/(93, 71^a^)	237–238.5
4	**5ad**	H	CF_3_	3.3/3	700	616	884/(97, 77^a^)	244–245
5	**5ba**	5-CH_3_	H	2.75/2.5	622	343	526/(84)	206–208
6	**5bb**	5-CH_3_	OCH_3_	2.75/2.5	622	418	482/(69)	213–215
7	**5bc**	5-CH_3_	Cl	2.75/2.5	622	429	520/(73)	246–247.5
8	**5bd**	5-CH_3_	CF_3_	3.3/3	746	616	891/(93)	234–236
9	**5ca**	5-Cl	H	1.56/1.42	350	194	373/(97, 29^d^)	212–214
10	**5cb**	5-Cl	OCH_3_	3.3/3	813	502	658/(73, 18^d^)	235–237
11	**5cc**	5-Cl	Cl	3.3/3	813	515	824/(90)	281.5–283
12	**5cd**	5-Cl	CF_3_	3.3/3	813	616	770/(76)	235–238
13	**5da**	6-Cl	H	1.56/1.42	350	194	323/(84, 22^d^)	191–193
14	**5db**	6-Cl	OCH_3_	2.2/2	493	334	523/(87, 20^d^)	229.5–231
15	**5dc**	6-Cl	Cl	3.3/3	813	515	861/(94)	228–229.5
16	**5dd**	6-Cl	CF_3_	3.3/3	813	616	796/(78, 30^c^)	292–295
17	**5ea**	5-NO_2_	H	2.2/2	565	274	445/(79)	296–298
18	**5eb**	5-NO_2_	OCH_3_	2.2/2	565	334	455/(73)	328–331
19	**5ec**	5-NO_2_	Cl	2.2/2	565	343	489/(78)	>372 dec.
20	**5ed**	5-NO_2_	CF_3_	2.2/2	565	410	674/(97)	327–330
21	**5ac’**	H	I	1.65/1.5	350	395	500/(93, 6^d^)	242–245
22	**5cc’**	5-Cl	I	1.65/1.5	407	395	520/(87, 8^d^)	257–258
23	**5dc’**	6-Cl	I	1.65/1.5	407	395	510/(86, 15^d^)	257–260

^a^In ACN (cf. reference [33]); ^b^prepared from 2-(phenylethynyl)phenylisocyanate (see reference [22]); ^c^prepared from oxindole and the corresponding benzonitrile (see reference [14]); ^d^prepared from oxindole and benzoic acid imidoester (see reference [11]).

### Reaction of **1a–e** with secondary thiobenzamides **3a–i**

Secondary thiobenzamides **3a–i** also react well with 3-bromooxindoles **1a–e** (Table 3) although the average yields of the corresponding products **6aa–ef** are always 10–15% lower when comparing substrates with the same R^1^ (R^2^ and R^3^ are always H). For example, while for the reaction of **1a** with **2a** the yield is 88% (Table 2, entry 1), a nitrogen substitution (**3a**: R^4^: C_6_H_5_ or **3f**: R^4^: CH_3_) lowers the isolated yields to 77% and 76% (Table 3, entries 1 and 6), respectively. The further substitution of the *N*-phenyl group with an EWG causes a significant decrease in the isolated yield (cf. entries 1, and 3–5 in Table 3) from 77% to only 32% while an EDG substituent (4-OCH_3_; entry 2 in Table 3) enhances the yield to 88%. From these observations it is clear that the influence of R^4^ is much stronger than the influence of the substituent R^1^ (cf. entries 1 and 10–13 or entries 6 and 14–17 in Table 3). The attached EWG substituent R^4^ lowers the p*K*_a_ (and thus also the equilibrium concentration) of the intermediary α-thioiminium salt which is much more reactive towards an internal nucleophilic attack than the free thioimidate group. Although an electron-withdrawing substituent R^4^ also enhances the internal nucleophilic attack to both α-thioiminium and imine groups – such influence is weaker because this addition occurs on a carbon atom more distant from R^4^. The presumption that the presence of an α-thioiminium group is a necessary prerequisite for the successful reaction progress is further supported by the fact that all *N*-alkyl-substituted thiobenzamides **3f–i** (see entries 6–9 in Table 3) give comparable yields around 70%.

**Table 3:** The prepared (*Z*)-3-[amino(phenyl)methylidene]-1,3-dihydro-2*H*-indol-2-ones **6aa–ef** with a secondary amino group (R^2^ and R^3^: H).

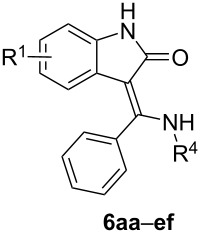								

entry	product	R^1^	R^4^	*n*_1a–e_/*n*_2a–d_ (mmol)	*m*_1a–e_ (mg)	*m*_3a–i_ (mg)	yield **6aa**–**ee** (mg)/(%)	mp (°C)

1	**6aa**	H	C_6_H_5_	3.3/3	700	640	721/(77%)	322.5–325
2	**6ab**	H	4-CH_3_O-C_6_H_4_	3.3/3	700	682	827/(88%, 82%^a^)	271–273
3	**6ac**	H	4-Cl-C_6_H_4_	3.3/3	700	743	503/(48%)	286.5–288
4	**6ad**	H	4-CF_3_-C_6_H_4_	3.3/3	700	844	488/(43%)	282–284
5	**6ae**	H	4-NO_2_-C_6_H_4_	3.3/3	700	775	339/(32%)	281–283.5
6	**6af**	H	CH_3_	2.75/2.5	556	378	475/(76%)	256–258
7	**6ag**	H	CH_3_(CH_2_)_4_	3.3/3	700	622	652/(71%)	214–216
8	**6ah**	H	C_6_H_5_CH_2_	3.3/3	700	724	696/(71%)	231–234
9	**6ai**	H	cyclo-C_6_H_11_	3.3/3	700	658	613/(64%)	343–346
10	**6ba**	5-CH_3_	C_6_H_5_	3.15/3	712	640	674/(69%)	258–261
11	**6ca**	5-Cl	C_6_H_5_	1.31/1.25	324	267	380/(89%, 68%^b^)	242–243
12	**6da**	6-Cl	C_6_H_5_	3.15/3	776	640	749/(72%)	319–322
13	**6ea**	5-NO_2_	C_6_H_5_	2.1/2	540	427	529/(74%)	267–269
14	**6bf**	5-CH_3_	CH_3_	3.15/3	712	454	521/(66%)	255–257.5
15	**6cf**	5-Cl	CH_3_	3.15/3	776	454	605/(71%)	256–258
16	**6df**	6-Cl	CH_3_	3.15/3	776	454	719/(84%)	274–276.5
17	**6ef**	5-NO_2_	CH_3_	3.15/3	810	454	656/(74%)	332.5–335

^a^Prepared from 2-(phenylethynyl)phenylisocyanate (see reference [23]); ^b^the *E*-isomer prepared from 3-bromo-3-(bromo(phenyl)methyl)-5-chlorooxindole (see reference [27]).

### Reaction of **1a–c** with tertiary thiobenzamides **4a–c**

The preliminary experiments involving the reaction of **1a** with *N*,*N*-dimethylthiobenzamide (**4a**) in DMF have shown that the reaction is much slower (>24 h) and also gives relatively high amounts of the undesirable isoindigo **9a** [32] (route c). In order to accelerate the Eschenmoser coupling reaction and to enhance the reaction yield (route b) an addition of a suitable thiophile assisting the sulfur extrusion should be beneficial [36]. Therefore, we tested two P(III) compounds as thiophiles. First, we added triphenylphosphine (1 equiv) but an even more complex mixture of products resulted. Much better results were obtained when trimethyl phosphite was used but this strongly depended on the used molar ratio: the best yield of the product **7aa** (83%) was achieved when using 1.5 equiv of trimethyl phosphite. While a lower amount (1 equiv) had only a moderate influence on the reaction progress (yield of **7aa** was 50%) a higher amount (3 equiv) caused a significant reduction of **1a** to the parent oxindole (yield of **7aa** was 44%). Therefore, the optimized amount (1.5 equiv) was used for the synthesis starting from the other thiobenzamides **4b** and **4c** and 3-bromooxindoles **1b** and **1c** (Table 4). From the inspection of Table 4 it is obvious that the isolated yields are lower (19–60%) but this is mainly due to the requirement for a more complex separation involving repeated column chromatography and/or crystallization.

**Table 4:** The prepared (*Z*)-3-[dimethylamino(phenyl)methylidene]-1,3-dihydro-2*H*-indol-2-ones **7aa–ca** with a tertiary amino group (R^3^ and R^4^: CH_3_).

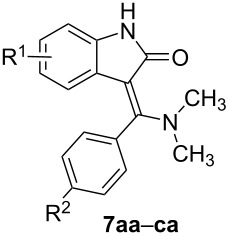								

entry	product	R^1^	R^2^	*n*_1a–e_/*n*_4a–c_ (mmol)	*m*_1a–e_ (mg)	*m*_4a–c_ (mg)	yield **7aa–ca** (mg)/(%)	mp (°C)

1	**7aa**	H	H	1.1/1	233	165	220/(83%)	239–240
2	**7ab**	H	CH_3_O	1.1/1	233	195	140/(48%)	272–273
3	**7ac**	H	Cl	1.1/1	233	200	179/(60%)	239.5–241
4	**7ba**	5-CH_3_	H	5.5/5	1243	825	740/(53%)	228–230
5	**7ca**	6-Cl	H	5.5/5	1360	825	280/(19%)	269–270.5

### Aliphatic thioamides

**Scheme 3:**
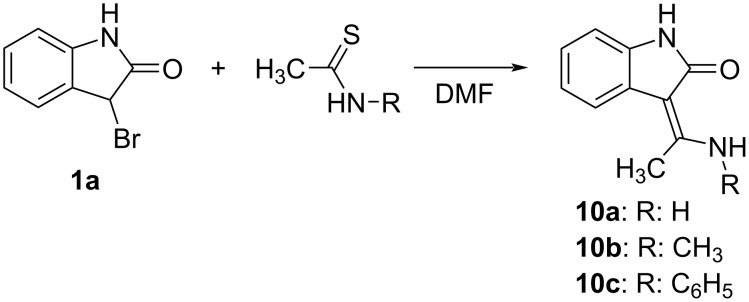
The Eschenmoser coupling reaction of 3-bromooxindole (**1a**) with thioacetamides.

Although our concern was mainly dedicated to the synthesis of 3-[amino(aryl)methylidene]oxindoles many of which display a significant tyrosin kinase inhibiting activity, we also verified the versatility of our method for the analogous oxindoles **10a–c** carrying a methyl group on the methylidene carbon (Scheme 3).

The replacement of thiobenzamide and its *N*-methyl or *N*-phenyl derivative with the analogous aliphatic thioacetamides has virtually no influence on the reaction progress and the corresponding oxindoles **10a–c** were obtained in 62–89% yields (Table 5).

**Table 5:** The prepared (*Z*)-3-[1-aminoethylidene]-1,3-dihydro-2*H*-indol-2-ones **10a–c**.

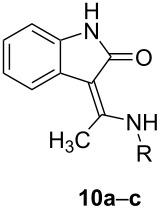							

entry	product	R	*n*_1a_/*n*_thioamide_ (mmol)	*m*_1a_ (mg)	*m*_thioamide_ (mg)	yield **10** (mg)/(%)	mp (°C)

1	**10a**	H	2/2	424	150	310/(89%, 65%^a^)	261–262.5
2	**10b**	CH_3_	2/2	424	178	320/(85%)	281–282
3	**10c**	C_6_H_5_	2/2	424	302	312/(62%)	222–223.5

^a^Prepared from oxindole and the corresponding acetonitrile (see reference [14]).

### Alternative electrophilic components for the Eschenmoser coupling reaction

The Eschenmoser coupling reaction [36–37] usually starts from α-substituted ketones, esters, malonates, or nitriles. The group in the α-position must be a good leaving group enabling a facile nucleophilic substitution giving the α-thioiminium salt and the cleaved leaving group should be a poor nucleophile in order to suppress the reverse reaction. In most cases halogens (mainly Br and I) fulfill these criteria but for highly congested thioamides only triflates gave desirable yields [36]. Moreover, several new alternatives to the Eschenmoser coupling reaction can be found in the recent literature [38–40].

**Scheme 4:**
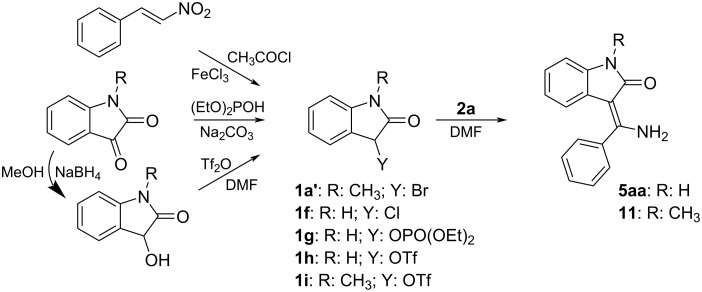
The synthesis of alternative 3-substituted oxindoles and their Eschenmoser coupling reaction with thiobenzamide (**2a**).

In our case, the 3-bromooxindoles **1a**–**e** worked very well but they are accessible from commercially available isatins only through a three-step synthesis in an acceptable overall yield 51–76% (see Table 6 in the Experimental). In order to avoid the relatively long synthesis of the precursors we tried to change Br for another leaving group Y (Scheme 4).

First, we prepared 3-chlorooxindole (**1f**) which is available from β-nitrostyrene and acetyl chloride under FeCl_3_ catalysis [41]. Unfortunately, the Eschenmoser coupling reaction with thiobenzamide (**2a**) did not proceed at all and only the formation of isoindigo **9a** was observed. However, a very easy synthesis of diethyl (2-oxoindolin-3-yl)phosphate (**1g**) from isatin has recently appeared [42] which encouraged us to submit this reagent to the Eschenmoser reaction. Unfortunately, this reagent reacts with the parent thiobenzamide (**2a**) very slowly even after heating to 80 °C. A maximum (chromatographic) yield 38% of **5aa** was achieved after 32 h when a double molar excess of phosphate **1g** was used. A further increase in the excess of **1g** had no positive effect on the yield of **5aa**. This result is in accordance with the relatively low nucleofugality of diethyl phosphate which is roughly quantified by the p*K*_a_ = 1.39 of its conjugated acid [43]. We have also examined an addition of 20% of tetrabutylammonium bromide as a nucleophilic catalyst which could transform the phosphate **1g** in situ to the more reactive bromo derivative **1a**. It was found that such addition has only a small positive effect on the reaction progress, most probably due to only a slightly higher nucleophilicity of the bromide (*n* = 3.9) [44] in comparison with the thiobenzamide (*n* = 3.1) [45].

The last possibility for the activation of the oxindole component involves the introduction of the triflate group into position 3. The synthesis of triflates according to the standard protocol involves the reaction of an alcohol with triflic chloride or anhydride. However, these very reactive reagents could react with 3-hydroxyoxindole both on the oxygen as well as on the nitrogen. Therefore, we first examined a protocol starting from *N*-methylisatin which was reduced to 3-hydroxy-*N*-methyloxindole [46] (yield 75%; note: the reduction of other isatins gives lower yields) and then treated with triflic anhydride under inert atmosphere at −20 °C. Quite a clean formation of the triflate **1i** was observed in an NMR tube but all attempts to isolate the pure compound **1i** in a preparative scale failed. Therefore, we decided to carry out an Eschenmoser coupling reaction in a one-pot manner. Into a chilled solution of 3-hydroxy-*N*-methyloxindole in DMF, one equivalent of triflic anhydride was added first and, after reaction completion, one equivalent of thiobenzamide (**2a**) was added. By this route, the desired product of the Eschenmoser coupling **11** was isolated in a very good yield of 87% (cf. with 78% in reference [33] and 64% in reference [14]). As a result, a good overall yield of 65% was achieved over three steps starting from *N*-methylisatin. For comparison, the four-step synthesis starting from *N*-methylisatin and giving **11** through 3-bromo-*N*-methyloxindole (**1a’**), has an overall yield of only 48%. Moreover, no formation of the isoindigo derivative was observed under such conditions – probably due to the presence of triflic acid evolved during the first step. This new route is therefore suitable for the synthesis of *N*-alkyl-3-(aminomethylidene)oxindoles.

We also tried to apply the same protocol for the parent (i.e. *N*-unprotected) 3-hydroxyoxindole and thiobenzamide (**2a**). We partly succeeded because we isolated the coupling product **5aa** in 65% yield (48% from isatin) which is still less than when starting from isatin through **1a** (57%). For a further two representatives, namely 4’-trifluoromethylthiobenzanilide (**3d**) and *N*,*N*-dimethylthiobenzamide (**4a**) the same one-pot method gives only 18% of the product **6ad** (cf. with 43% from **1a**) and 27% (without thiophile) of **7aa** (cf. with 86% from **1a**). It was obvious that an improved synthetic approach would necessitate the protection of the oxindole nitrogen avoiding the formation of the undesired *N*-Tf derivative. Such nitrogen protection/deprotection (e.g., acetylation/deacetylation) is often used in the other synthetic strategies described in Scheme 1. Therefore, the starting isatin was acetylated first with acetic anhydride [47] in 95% yield and then reduced with various complex borohydrides (e.g., sodium borohydride, sodium cyanoborohydride, or sodium triacetoxyborohydride) in methanol. Unfortunately, formation of the desired *N*-acetyl-3-hydroxyoxindole was not observed at all, because a base-catalyzed addition of methanol to the C^3^=O and subsequent intramolecular rearrangement of the acetyl group occurs to give 3-methoxy-2-oxoindolin-3-yl acetate. Other reducing agents recommended in the literature for the reduction of unprotected isatin (sodium dithionite, zinc, phosphinic acid, etc.) also failed or caused an acetyl group removal. Therefore, we abandoned this strategy.

### Configuration of products **5–7**

All 3-(aminomethylidene)oxindoles can formally exist as (*Z*)- or (*E*)-isomers. Their actual configuration depends on the substitution at the methylidene carbon as well as the nitrogen. While derivatives containing an unsubstituted methylidene carbon and tertiary nitrogen exist as a mixture of rapidly equilibrating (*Z*)/(*E*)-isomers [2], the derivatives containing a secondary nitrogen slightly prefer [2,9,48] the (*Z*)-configuration due to an intramolecular hydrogen bond. A substitution of hydrogen at the methylidene carbon by an aryl group and a secondary nitrogen, enabling an intramolecular hydrogen bond, results in the geometry of a double bond that is locked in a (*Z*)-configuration [12]. The same (*Z*)-configuration was observed by us for the primary 3-[amino(aryl)methylidene]oxindoles [33].

The usual method used for the configuration assignment of 3-amino(aryl)methylidene derivatives involves a simple ^1^H NMR experiment showing either an upfield (δ ≈ 5.7–6.4 ppm for (*Z*)-configuration [5,12,32–33]) or a downfield shift (δ ≈ 7.7–7.9 ppm for (*E*)-configuration [27]) of the proton in position 4 of the oxindole moiety. In our previous paper [32] such an assignment was also confirmed in the solid state by X-ray diffraction. The important role of the intramolecular hydrogen bond fixing the (*Z*)-configuration has also been observed in the crystal structures of other 3-aminomethylidene derivatives containing a primary or secondary nitrogen [14,49–52].

The (*Z*)-configuration of the newly prepared 3-[amino(aryl)methylidene]oxindoles with primary, secondary, and tertiary nitrogen (series **5**–**7**) has been therefore confirmed by ^1^H NMR spectroscopy in CDCl_3_ or DMSO-*d*_6_ solution. In all spectra the proton in position 4 of the oxindole moiety shows a typical upfield shift in the range of 5.7–6.4 (series **5**), 5.3–5.9 (series **6**), and 5.0–5.5 ppm (series **7**), except for the nitro derivatives (R^1^: 5-NO_2_) where the nitro group always enhances the Ar–H_4_ chemical shift by about 0.7 ppm. Therefore, a 2D-NOESY experiment was performed for some representative 5-NO_2_ derivatives (**5eb–ed**) confirming their (*Z*)-configuration (see Supporting Information File 1).

## Conclusion

We have developed an efficient and versatile synthesis of (*Z*)-3-[amino(phenyl/methyl)methylidene]-1,3-dihydro-2*H*-indol-2-ones (series **5**, **6**, **7**, **10**, and compound **11**) starting from 3-bromooxindoles **1a**–**e** and primary, secondary or tertiary thioacetamides and thiobenzamides **2**–**4**. The superiority of our method in terms of the isolated yield (mostly >70% but often >85%) is clearly seen in all relevant cases (see Table 2 and Table 3) in which a direct comparison with other preparative routes [11,14,22–23] is possible. Although the starting 3-bromooxindoles **1a–e** are available from the corresponding isatins by a three-step synthesis (overall yield 51–76%), the total yields related to these commercially available and cheap starting compounds are better or at least competitive to the methods described in Scheme 1 of which several have serious disadvantages. For example, the method starting from *N*-phenylpropiolamides [24] is not applicable (i.e., 0% yield) for the preparation of 1-unsubstituted 3-(aminomethylidene)oxindoles and the method involving the transformation of 3-bromo-3-[bromo(phenyl)methyl]oxindoles [27] gives the products with inversed configuration of the double bond. Several synthetic strategies starting from isocyanate [22–23] or oxindole [4,12] precursors work well only with protected nitrogen(s) in the starting compounds whose preparation and protection/deprotection also lengthens the total synthesis and decreases the overall isolated yield of the desired 3-(aminomethylidene)oxindoles.

The main advantages of our synthetic approach using an Eschenmoser coupling reaction involve the easy elaboration with no need for an inert atmosphere, extra dry solvents or toxic starting compounds (e.g*.,* triphosgene needed for the synthesis of isocyanates) and (except for tertiary thioamides) addition of a thiophile. Moreover, our method has almost no limitation in terms of substitutions on each part of the skeleton.

Our alternative and shorter synthetic approach also starts from isatin and involves its reduction to 3-hydroxyoxindole and a one-pot triflation/Eschenmoser coupling with thiobenzamides under an inert atmosphere. However, this procedure is only suited for the preparation starting from *N*-methyl-3-hydroxyoxindole in which excessive *N*-triflication is blocked and the overall yield of 1-methyl-3-(aminomethylidene)-1,3-dihydro-2*H*-indol-2-one (**11**) (to isatin) is better than those obtained through 3-bromo-*N*-methyloxindole (**1a’**). In the case of parent isatin such a strategy also gives the desired products **5aa**, **6ad**, and **7aa** but in a lower overall yield than the yield obtained through **1a**. Other leaving groups in position 3 of the oxindole are unsuitable because only very slow (phosphate) or even no Eschenmoser coupling reaction (chloride) occurs with them.

## Experimental

The starting 3-bromooxindoles **1a–e** and 3-bromo-*N*-methyloxindole (**1a’**) were synthesized from the corresponding isatins using the procedure described in our previous paper [33] (see also Supporting Information File 1). The following Table 6 summarizes the overall yields after three steps.

**Table 6:** The yields of the synthesized 3-bromooxindoles **1a–e**.^a^

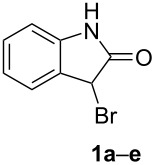			

entry	product **1**	R^1^	overall yield (%)

1	**1a**	H	65*
2	**1b**	5-CH_3_	54
3	**1c**	5-Cl	76
4	**1d**	6-Cl	64
5	**1e**	5-NO_2_	55

^a^The corresponding 3-bromo-*N*-methyloxindole (**1a’**) was obtained in 55% yield.

3-Hydroxyoxindole and 3-hydroxy-*N*-methyloxindole were prepared [46] from the corresponding isatins through the reduction with sodium borohydride in methanol (see also Supporting Information File 1). The primary thiobenzamides **2a–d** were prepared by the magnesium chloride-catalyzed thiolysis of commercially available benzonitriles [53] and the secondary thiobenzamides **3a**–**h** were obtained by the thionation of the corresponding *N*-substituted amides [54] using pyridine–P_4_S_10_ as sulfurization agent. The tertiary thiobenzamides **4a–c** were synthetized by a one-pot acylation/thionation from the corresponding acid chlorides and dimethylamine [55]. Other chemicals and solvents were purchased from Acros Organics, Sigma-Aldrich, or Fluorochem and were used as received. The melting points are uncorrected. ^1^H and ^13^C (APT) NMR spectra were recorded on a Bruker Avance III 400 MHz or on a Bruker Ascend 500 MHz instrument. The chemical shifts δ are referenced to TMS (δ = 0) or solvent residual peaks δ(CDCl_3_) = 7.24 ppm (^1^H) and 77.0 ppm (^13^C), δ(DMSO-*d*_6_) = 2.50 ppm (^1^H) and 39.6 ppm (^13^C). High-resolution mass spectra were recorded on a MALDI LTQ Orbitrap XL equipped with a nitrogen UV laser (337 nm, 60 Hz, 8–20 μJ) in the positive ion mode. For the CID experiment using the linear trap quadrupole (LTQ) helium was used as the collision gas and 2,5-dihydroxybenzoic acid (DHB) or (2-methylprop-2-en-1-yliden)malononitrile (DCTB) as the MALDI matrix. Elemental analyses were performed on a Flash 2000 Organic Elemental Analyser (Thermofisher). For samples containing chlorine mercurimetric titration was used. IR spectra were recorded on a Nicolet iS50 equipped with an ATR diamond crystal (neat solid samples). Flash chromatography was performed using a Büchi Reveleris^®^ X2 flash chromatography system equipped with a UV–vis/ELSD detector and Reveleris Flash pure cartridges (12–40 g, 35–45 μm, 53–80 Å) or puriFlash^®^ Alumine N 32/63 µm cartridges (12 g).

### General procedure for the synthesis of 3-[amino(phenyl)methylidene]-1,3-dihydro-2*H*-indol-2-ones (**5aa–ed** and **6aa–ef**)

In a 20 mL flask with a magnetic stirrer the solutions of the corresponding 3-bromooxindole **1a**–**e** and substituted thiobenzamide **2a–d** or **3a–i** (amounts specified in Table 2 and Table 3) in dry DMF (5 + 5 mL for 3 mmol scale) were mixed and the mixture stirred for 5 h (primary thiobenzamides) or 12 h (secondary thiobenzamides) at room temperature. Then, TEA (2 equiv) was added and the mixture stirred for additional 5 min and diluted with water (100 mL). The aqueous solution was extracted with DCM (3 × 60 mL) and the combined organic layers were washed with water (2 × 40 mL) and brine (40 mL), dried with anhydrous Na_2_SO_4_ and evaporated. The solid residue was dissolved in methanol and filtered through a plug of Celite. The filtrate was evaporated with silica gel and submitted to preparative flash chromatography (silica gel or alumina cartridge; mobile phase and gradient are specified in Supporting Information File 1). Analytically pure samples were obtained by crystallization from the appropriate solvent.

Compounds **5ea** and **5eb** were isolated as follows: After the reaction of **1e** with **2a**,**b** (4 h) the reaction mixture was poured into cold EtOAc/EtOH 20:1 (80 mL) and stirred for 30 min. The precipitated product was filtered off and washed with EtOAc (2 × 5 mL). The crude **5ea** or **5eb** was dissolved in boiling CHCl_3_/CH_3_OH 1:1 (ca 125 mL) and filtered through a short plug of silica gel which was then washed with another portion (50 mL) of CHCl_3_/CH_3_OH 1:1. The collected filtrates were dried and evaporated.

### Synthesis of 3-[dimethylamino(phenyl)methylidene]-1,3-dihydro-2*H*-indol-2-ones (**7aa–ca**)

The 4-substituted *N*,*N*-dimethylthiobenzamide (**4a–c**, *n***_4a–c_**) and trimethyl phosphite (1.5 equiv) were dissolved in a minimum amount of dry DMF and a saturated solution of 3-bromooxindole (**1a–c**, *n***_1a–c_**) in dry DMF was added. The reaction mixture was stirred for 20 h at room temperature (TLC monitoring; silica gel plates/EtOAc/hexane) and then diluted with an aqueous NaHCO_3_ solution (5%, 50 mL). The suspension was extracted with EtOAc (3 × 150 mL) and the combined organic layer was washed with water (5 × 100 mL), brine (2 × 100 mL), dried with anhydrous Na_2_SO_4_ and evaporated. The residue was repeatedly evaporated with EtOH to remove trimethyl thiophosphate. The crude product was then submitted to preparative flash chromatography (silica gel or alumina cartridge; mobile phase and gradient are specified in the Supporting Information File 1).

### Synthesis of 3-[amino(methyl)methylidene]-1,3-dihydro-2*H*-indol-2-ones (**10a–c**)

The corresponding thioacetamide (2 mmol) was dissolved in DMF (5 mL) and a solution of **1a** (424 mg, 2 mmol) in dry DMF (5 mL) was added. The mixture was stirred overnight at room temperature and then evaporated in vacuum. The residue was dissolved in DCM (40 mL), evaporated with neutral alumina (5 g) and submitted to preparative flash chromatography (silica gel or alumina cartridge; mobile phase and gradient are specified in Supporting Information File 1).

## Supporting Information

File 1 Synthetic procedures, characterization data and copies of spectra.
