# Supplementary material for: Synthesis of (Z)-3-[amino(phenyl)methylidene]-1,3-dihydro-2H-indol-2-ones using an Eschenmoser coupling reaction
Source: Beilstein J Org Chem. 2021 Feb 23;17:527–39. doi: 10.3762/bjoc.17.47 (PMC7934781; doi:10.3762/bjoc.17.47)
Supplement: File 1 — Synthetic procedures, characterization data and copies of spectra. [file Beilstein_J_Org_Chem-17-527-s001.pdf]

BEILSTEIN JOURNAL OF ORGANIC CHEMISTRY

Supporting Information

for

Synthesis of (*Z*)-3-[amino(phenyl)methylidene]-1,3-dihydro-2*H*-indol-2-ones using an Eschenmoser coupling reaction

Lukáš Marek, Lukáš Kolman, Jiří Váňa, Jan Svoboda and Jiří Hanusek

Beilstein J. Org. Chem. **2021,** *17,* 527–539. doi:10.3762/bjoc.17.47

Synthetic procedures, characterization data and copies of spectra

License and Terms: This is a supporting information file under the terms of the Creative Commons Attribution License (https://creativecommons.org/licenses/by/4.0). Please note that the reuse, redistribution and reproduction in particular requires that the author(s) and source are credited and that individual graphics may be subject to special legal provisions.
The license is subject to the *Beilstein Journal of Organic Chemistry* terms and conditions: (https://www.beilstein-journals.org/bjoc/terms)

Table of contents

Synthesis and characterization of starting compounds... S2–S6

Characterization of Eschenmoser coupling products **5aa**–**ed**... S7–S12

Characterization of Eschenmoser coupling products **6aa**–**ef**.. S13–S17

Characterization of Eschenmoser coupling products **7aa**–**ca** S17–S18

Characterization of Eschenmoser coupling products **10a**–**c** S19

Eschenmoser coupling of **1g** with **2a** S20

^{1}H and ^{13}C NMR spectra of starting compounds S21–S35

^{1}H and ^{13}C NMR spectra of Eschenmoser coupling products **5aa**–**ed**.......................... S36– S56

^{1}H and ^{13}C NMR spectra of Eschenmoser coupling products **6aa**–**ef**............................ S57–S73

^{1}H and ^{13}C NMR spectra of Eschenmoser coupling products **7aa**–**ca** S74–S78

^{1}H and ^{13}C NMR spectra of Eschenmoser coupling products **10a**–**c**, **11** S79–S82

2D NOESY NMR spectra of Eschenmoser coupling products **5eb**–**ed**......................... S82–S83

MALDI-HRMS spectra of Eschenmoser coupling products **5aa**–**ed** S84–S95

MALDI-HRMS spectra of Eschenmoser coupling products **6aa**–**ef** S95–S103

MALDI-HRMS spectra of Eschenmoser coupling products **7aa**–**ca**......................... S104–S106

MALDI-HRMS spectra of Eschenmoser coupling products **10a**–**c**, **11**..................... S106–S107

IR spectra of new Eschenmoser coupling products series **5**, **6**, **7**, **10** S108–S122

References S122

Synthesis of starting 3-bromooxindoles 1a–e

1. step:

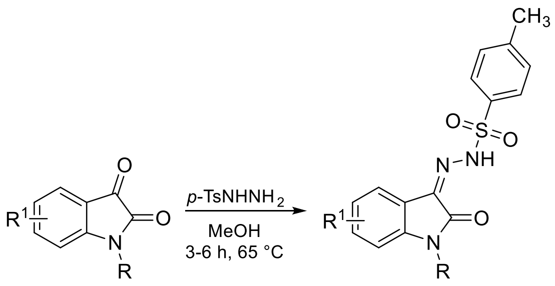

R: H and R^1: **a**) H, **b**) 5-CH_3, **c**) 5-Cl, **d**) 6-Cl, **e**) 5-NO_2
R: CH_3 and R_1: H

The corresponding isatin (*n*; mmol) was suspended in CH_3OH (V_{CH_3OH}; mL) at 50 °C. *p*-Toluenesulfonylhydrazine (1.1 equiv) was added in one portion and the reaction mixture was refluxed (t_{reflux}) and the reaction progress was monitored using TLC. The reaction mixture was concentrated to half of its volume and cooled to 0–5 °C. The precipitated mixture of (*Z*)/(*E*)-isomers of isatin *p*-tosylhydrazone was filtered off and washed with cold CH_3OH.

Table S1 – Molar and weight amounts of reactants and product yields and their melting points.

**R**	**R^1**	**n_{isatin} [mmol]**	**m_{isatin} [g]**	**V_{CH_3OH} [ml]**	**t_{reflux} [h]**	**Yield [g]**	**Yield [%]**	**m.p. [°C]**
H	H	24	3.53	100	3	6.2	82	199-202
H	5-CH_3	12.5	2.01	40	6	3.3	79	194-196
H	5-Cl	41	7.44	135	3	12.8	89	232-233
H	6-Cl	14	2.54	65	3	3.7	75	210-211
H	5-NO_2	20	3.84	90*	4	5.4	75	209-210
CH_3	H	30	5.00	170*	1	8.4	83	194-196

*In THF

2. step:

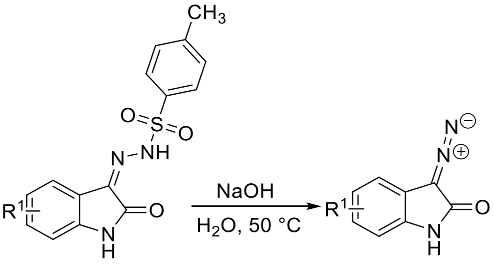

R: H and R^1: **a**) H, **b**) 5-CH_3, **c**) 5-Cl, **d**) 6-Cl, **e**) 5-NO_2
R: CH_3 and R_1: H

The corresponding isatin *p*-tosylhydrazone (*n*; mmol) was suspended in water (10 mL per each 1 mmol of *p*-tosylhydrazone) and 10% aqueous NaOH (4.5 mmol per each 1 mmol of tosylhyrazone; 4.5 equiv) was added in one portion. The suspension was stirred at 50 °C until dissolution of all solid material accompanied by a change of the solution color to orange ($t_{heating}$). Then, the reaction mixture was cooled and extracted with EtOAc (4 × 75 mL). The combined organic layers were washed with water (50 mL), dried with anhydrous Na_2SO_4, and evaporated. Yields and ^1H NMR data are given below.

Table S2 – Molar and weight amounts of reactants and product yields and their melting points.

R	R^1	$n_{hydrazone}$ [mmol]	$m_{hydrazone}$ [g]	m_{NaOH} [g]	$t_{heating}$ [h]	Yield [g]	Yield [%]	m.p. [°C]
H	H	15.8	5.00	2.84	3	2.4	96	165-167
H	5-Me	9.1	3.00	1.63	3	1.5	96	190-194
H	5-Cl	20.0	7.00	3.60	2	3.5	89	219-224
H	6-Cl	8.6	3.00	1.55	2	1.5	92	192 (dec.)
H	5-NO_2	13.9	5.00	2.50	5	2.3	81	255 (dec.)
CH_3	H	6.0	2.00	1.10	3	0.9	86	83-85

3-Diazooxindole:
^{1}H NMR (400 MHz, DMSO-d_6) δ: 10.66 (bs, 1H, NH), 7.39 (d, *J* 7.6 Hz, 1H, ArH), 7.09 (t, *J* 7.6 Hz, 1H, ArH), 6.99 (t, *J* 7.6 Hz, 1H, ArH), 6.91 (d, *J* 7.8 Hz, 1H, ArH). ^{13}C NMR (100 MHz, DMSO-d_6) δ: ^{13}C NMR (100 MHz, DMSO-d_6) δ: 167.9 (C=O), 132.7, 125.2, 121.3, 119.3, 117.1, 110.0, 60.3 (>C=N$^+$=N$^-$) correspond to Ref. [1].

5-Methyl-3-diazooxindole:
^{1}H NMR (500 MHz, DMSO-d_6) δ: 10.53 (bs, 1H, NH), 7.18 (s, 1H, ArH), 6.88 (d, *J* 8.0, 1H, ArH), 6.79 (d, *J* 7.9 Hz, 1H, ArH). ^{13}C NMR (100 MHz, DMSO-d_6) δ: 168.1 (C=O), 130.5, 130.4, 125.9, 119.7, 117.2, 109.8, 60.1 (>C=N$^+$=N$^-$), 20.9 (CH_3).

5-Chloro-3-diazooxindole:
^{1}H NMR (400 MHz, DMSO-d_6) δ: 10.77 (bs, 1H, NH), 7.53 (d, *J* 2.1 Hz, 1H, ArH), 7.09 (dd, *J* 8.3 and 2.2 Hz, 1H, ArH), 6.87 (d, *J* 8.3 Hz, 1H, ArH). ^{13}C NMR (100 MHz, DMSO-d_6) δ: 167.4 (C=O), 131.4 (C_{quart}), 125.3 (C_{quart}), 124.7 (CH), 119.1 (C_{quart}), 119.0 (CH), 111.1 (CH), 60.8 (>C=N$^+$=N$^-$) correspond to Ref. [2]

6-Chloro-3-diazooxindole:
^{1}H NMR (400 MHz, DMSO-d_6) δ: 10.79 (bs, 1H, NH), 7.38 (d, *J* 8.2 Hz, 1H, ArH), 7.00 (dd, *J* 8.2 and 1.6 Hz, 1H, ArH), 6.89 (d, *J* 1.5 Hz, 1H, ArH) corresponds to Ref [3]. ^{13}C NMR (100 MHz, DMSO-d_6) δ: 167.7 (C=O), 133.7, 129.4, 121.0, 120.4, 116.1, 110.0, 60.6 (>C=N$^+$=N$^-$).

5-Nitro-3-diazooxindole:
^{1}H NMR (400 MHz, DMSO-d_6) δ: 8.44 (d, *J* 2.3 Hz, 1H, ArH), 8.01 (dd, *J* 8.7 and 2.4 Hz, 1H, ArH), 7.05 (d, *J* 8.7 Hz, 1H, ArH), 3.62 (vbs, 1H, NH + H_2O) corresponds to Ref [2]. ^{13}C NMR (100 MHz, DMSO-d_6) δ: 167.8 (C=O), 141.8, 138.5, 121.6, 118.5, 114.9, 109.7, 61.7 (>C=N$^+$=N$^-$).

3-Diazo-*N*-methyloxindole:
^{1}H NMR (400 MHz, $CDCl_3$) δ: 7.16-7.24 (m, 2H, ArH), 7.10 (t, *J* 7.5 Hz, 1H, ArH), 6.93 (d, *J* 8.0 Hz, 1H, ArH), 3.34 (s, 3H, CH_3) corresponds to Ref. [4].

3. step:

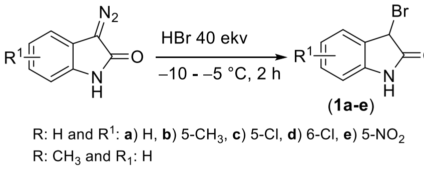

The corresponding well-grinded diazooxindole (*n*; mmol) was added in small portions to a stirred cold (−10 °C) aqueous 46% HBr (40 equiv.; V_{HBr}) solution until the nitrogen evolution ceased. Then, the reaction mixture was stirred for the next 2 hours at room temperature to complete the reaction (monitoring by TLC; SiO_2 plates, $CHCl_3/CH_3OH$ 9:1). The final suspension was filtered and washed with water until neutral pH of the filtrate and the solid residue was dried.

Table S3 – Molar and weight amounts of reactants and product yields and their melting points.

	R	R1	*n* [mmol]	*m* [g]	V_{HBr} [ml]	Yield [g]	Yield [%]	M. p. [°C]
**1a**	H	H	15.1	2.4	72	2.6	82	162-163
**1b**	H	5-Me	15.0	2.6	68	2.4	71	173-174
**1c**	H	5-Cl	13.0	2.5	61	3.1	96	179 (dec.)
**1d**	H	6-Cl	10.5	2.0	50	2.4	92	162-163
**1e**	H	5-NO_2	10.8	2.2	51	3.1	91	205 (dec.)
**1a'**	CH_3	H	3.6	0.6	30	0.6	76	123-124

3-Bromooxindole (1a)

^{1}H NMR (500 MHz, DMSO-d_6) δ: 10.79 (bs, 1H, NH), 7.33 (d, *J* = 7.59 Hz, 1H, ArH), 7.29-7.25 (m, 1H, ArH), 7.02 (td, *J* = 1.02 and 7.59 Hz, 1H, ArH), 6.85 (d, *J* = 7.59 Hz, 1H, ArH), 5.72 (s, 1H, CH). ^{13}C NMR-APT (125 MHz, $CDCl_3$) δ (ppm) 174.7 (C=O), 140.9 (C_{quart}), 130.4 (CH), 126.6 (C_{quart}), 126.3 (CH), 123.4 (CH), 110.6 (CH), 38.9 (CH) correspond to Ref. [1]

3-Bromo-5-methyloxindole (1b)

^{1}H NMR (400 MHz, DMSO-d_6) δ: 10.67 (bs, 1H, NH), 7.14 (s, 1H, Ar-H_4), 7.06 (d, *J* 7.9 Hz 1H, Ar-H), 6.74 (d, *J* 7.9 Hz, 1H, Ar-H), 5.66 (s, 1H, CH), 2.25 (s, 3H, CH_3). ^{13}C NMR (100 MHz, DMSO-d_6) δ: 173.7 (C=O), 140.0 (C_{quart}), 131.4 (C_{quart}), 130.6 (CH), 127.2 (C_{quart}), 126.6 (CH), 110.0 (CH), 40.8 (CH), 20.6 (CH_3).

3-Bromo-5-chlorooxindole (1c)

^{1}H NMR (400 MHz, DMSO-d_6) δ: 10.90 (bs, 1H, NH), 7.38 (s, 1H, Ar-H_4), 7.31 (dd, *J* 8.3 and 2.0 Hz, 1H, Ar-H_6), 6.87 (d, *J* 8.3 Hz, 1H, Ar-H_7), 5.69 (s, 1H, CH). ^{13}C NMR (100 MHz, DMSO-d_6) δ: 173.4 (C=O), 141.3 (C_{quart}), 130.1 (CH), 129.2 (C_{quart}), 126.2 (C_{quart}), 126.0 (CH), 111.8 (CH), 39.6 (CH).

3-Bromo-6-chlorooxindole (1d)

^{1}H NMR (400 MHz, DMSO-d_6) δ: 10.92 (bs, 1H, NH), 7.35 (d, J = 8.0 Hz, 1H, Ar-H_4), 7.07 (dd, J = 8.0 and 1.7 Hz, 1H, Ar-H_5), 6.88 (d, J = 1.4 Hz, 1H, Ar-H_7), 5.71 (s, 1H, CH). ^{13}C NMR (100 MHz, DMSO-d_6) δ: 173.7 (C=O), 143.9 (C_{quart}), 134.4 (C_{quart}), 127.5 (CH), 126.1 (C_{quart}), 122.1 (CH), 110.3 (CH), 39.6 (CH).

3-Bromo-5-nitrooxindole (1e)

^{1}H NMR (500 MHz, DMSO-d_6) δ: 11.49 (bs, 1H, NH), 8.22 (dd, J = 8.7 and 1.8 Hz, 1H, ArH), 8.18 (s, 1H, ArH), 7.06 (d, J = 8.7 Hz, 1H, ArH), 5.82 (s, 1H, CH). ^{13}C NMR (125 MHz, DMSO-d_6) δ: 174.1 (C=O), 148.8 (C_{quart}), 142.6 (C_{quart}), 128.4 (C_{quart}), 127.3 (CH), 121.6 (CH), 110.6 (CH), 38.7 (CH).

3-Bromo-*N*-methyloxindole (1a')

^{1}H NMR (400 MHz, $CDCl_3$) δ: 7.41 (d, J = 7.5 Hz, 1H, ArH), 7.35 (t, J = 7.8 Hz, 1H, ArH), 7.11 (t, J = 7.3 Hz, 1H, ArH), 6.83 (d, J = 7.8 Hz, 1H, ArH), 5.27 (s, 1H, CH), 3.23 (s, 3H, CH_3) corresponds to corresponds to Ref. [1].

3-Hydroxy-*N*-methyloxindole

N-Methylisatin (2.5 g, 15.5 mmol) was suspended in CH_3OH (45 mL) and $NaBH_4$ (0.75 g, 19.8 mmol) was added in one portion at 0 °C under vigorous stirring. After 5 min, the homogeneous decolorized solution was evaporated at 30 °C under vacuum and the residue was extracted with EtOAc (100 mL). The organic phase was washed with water (2 × 20 mL) and brine (40 mL), dried with anhydrous Na_2SO_4, and evaporated. The crude product was crystallized from CH_3OH (5 mL) by cooling to −20 °C. White crystals (1.9 g, 75%); m.p. 151-152 °C; ^{1}H NMR (400 MHz, $CDCl_3$) δ 7.47 (d, J 7.3 Hz, 1H, Ar-H), 7.33 (t, J 7.7 Hz, 1H, Ar-H), 7.11 (t, J 7.5 Hz, 1H, Ar-H), 6.82 (d, J 7.8 Hz, 1H, Ar-H), 5.12 (s, 1H, CH), 4.71 (bs, 1H, OH), 3.18 (s, 3H, CH_3); ^{13}C NMR (100 MHz, $CDCl_3$) δ (ppm): 177.4, 143.7, 129.7, 127.1, 125.1, 123.2, 108.4, 69.8, 26.2 corresponds to Ref. [5].

3-Hydroxyoxindole

Prepared from isatin (4.56 g, 31 mmol) and $NaBH_4$ (1.50 g, 19.8 mol) in CH_3OH (60 ml) at 0 °C using the same procedure as for 3-hydroxy-*N*-methyloxindole. The solid residue was triturated with Et_2O (15 mL) at 0 °C. White crystals (2.3 g, 50%); m. p. 165-167 °C; ^{1}H NMR (400 MHz, DMSO-d_6) δ: 10.23 (bs, 1H, NH), 7.28 (d, J = 7.3 Hz, 1H, ArH), 7.20 (t, J = 7.6 Hz, 1H, ArH), 6.95 (t, J = 7.4 Hz, 1H, ArH), 6.78 (d, J = 7.6 Hz, 1H, ArH), 6.18 (d, J = 7.6 Hz, 1H, CH), 4.82 (d, J = 7.6 Hz, 1H, OH).). ^{13}C NMR (100 MHz, DMSO-d_6) δ: 178.1 (C=O), 142.3, 129.4, 129.1, 124.9, 121.7, 109.6, 69.3 correspond to Ref. [6]

3-Chlorooxindole (1f)

trans-β-Nitrostyrene (3.2 g, 14 mmol) was dissolved in DCM (50 mL) and acetyl chloride (2.1 mL, 30 mmol) was added in one portion. Anhydrous $FeCl_3$ (4.6 g, 29 mmol) was added in three

portions at 25 °C during 15 min and the resulting mixture was stirred for 5 hours. Then, the reaction mixture was diluted with 3% aq. HCl (200 mL) and extracted with DCM (3 × 50 mL). The organic layer was washed with water (2 × 50 mL), brine (50 mL), dried with anhydrous Na_2SO_4, and evaporated. The crude product was dissolved in DCM (70 mL), evaporated with SiO_2 (15 g) and purified using preparative flash liquid chromatography (silica gel cartridge 40 g, mobile phase $CHCl_3$/CH_3OH, gradient 0–5% CH_3OH during 15 min). The pure product was obtained by crystallization from hot toluene. White crystals (0.8 g, 35%); m.p. 161-162 °C; ^{1}H NMR (400 MHz, $CDCl_3$) δ 10.77 (bs, 1H, NH), 7.36 (d, *J* 7.4 Hz, 1H, Ar-H), 7.29 (t, *J* 7.6 Hz, 1H, Ar-H), 7.03 (td, *J* 7.6 and 0.8 Hz, 1H, Ar-H), 6.87 (d, *J* 7.8 Hz, 1H, Ar-H), 5.57 (s, 1H, CH). ^{13}C NMR (100 MHz, $CDCl_3$) δ (ppm): 173.3, 142.5, 130.4, 126.6, 125.7, 122.4, 110.2, 52.3 corresponds to Ref. [7].

Diethyl (2-oxoindolin-3-yl)phosphate (1g)

Isatin (4.4 g, 30 mmol) was suspended in dry acetonitrile (50 mL) and diethyl phosphite (4.15 g; 30 mmol) and anhydrous well-grinded K_2CO_3 (0.44 g, 3 mmol) were added under argon atmosphere. The reaction mixture was stirred at 60 °C for 1.5 h and then filtered. Residual salts were washed with acetonitrile (2 × 10 mL) and the combined filtrates were evaporated. The crude oily product (8.5 g, 99%) was dissolved in EtOAc (40 mL), evaporated with neutral alumina (30 g) and submitted to preparative flash chromatography (silica gel cartridge 80 g, mobile phase *n*-hexane/EtOAc, gradient 0–100% EtOAc during 20 min). The resulting colorless oil (7.1 g, 84%) slowly solidified to white solid with m.p. 86 – 88 °C. ^{1}H NMR (500 MHz, $CDCl_3$) δ 9.59 (bs, 1H, NH), 7.50 (d, *J* 7.5 Hz, 1H, Ar-H), 7.25 (t, *J* 7.8 Hz, 1H, Ar-H), 7.02 (t, *J* 7.5 Hz, 1H, Ar-H), 6.91 (d, *J* 7.8 Hz, 1H, Ar-H), 5.58 (d, *J* 12.8 Hz, 1H, C-H), 4.16-4.34 (m, 4H, 2× OCH_2), 1.30-1.40 (m, 6H, 2× CH_3) correspond to [8].

(*Z*)-3-[Amino(phenyl)methylidene]-1,3-dihydro-2*H*-indol-2-one (5aa)
The crude product was purified using preparative flash liquid chromatography (silica gel, mobile phase DCM/EtOAc + 3% TEA, gradient 0–20% EtOAc during 20 min). Yellow crystals (623 mg, 88%); mp 239 – 240 °C (EtOH) (Ref. [1] gives 238-239 °C); ^{1}H NMR (400 MHz, DMSO-d_6) δ 10.36 (bs, 1H, NH), 9.45 (bd, *J* = 3.2 Hz, 1H, ½NH_2), 8.03 (bd, *J* = 2.8 Hz, 1H, ½NH_2), 7.55-7.64 (m, 3H, Ar-H), 7.48-7.55 (m, 2H, Ar-H), 6.76-6.88 (m, 2H, Ar-H), 6.52 (t, 1H, *J* = 7.6 Hz, Ar-H_6), 6.01 (d, *J* = 7.7 Hz, 1H, Ar-H_4) correspond to Ref. [1].

(*Z*)-3-[Amino(4-methoxyphenyl)methylidene]-1,3-dihydro-2*H*-indol-2-one (5ab)
The crude product was purified using preparative flash liquid chromatography (silica gel, mobile phase DCM/EtOAc + 3% TEA, gradient 0–30% EtOAc during 20 min). Yellow crystals (655 mg, 82%); mp 229.5 – 231 °C (toluene/CH_3OH) (mp 178-180 °C given in Ref. [32] is probably interchanged with thiazole derivative with mp 230-233 °C also cited therein); ^{1}H NMR (400 MHz, DMSO-d_6) δ 10.34 (bs, 1H, NH), 9.47 (bd, *J* = 3.3 Hz, 1H, ½NH_2), 7.91 (bd, *J* = 3.0 Hz, 1H, ½NH_2), 7.48 (AA'XX', *J* = 8.7 Hz, 2H, Ar-H), 7.12 (AA'XX', *J* = 8.7 Hz, 2H, Ar-H), 6.72-6.88 (m, 2H, Ar-H), 6.56 (t, *J* = 7.5 Hz, 1H, Ar-H), 6.24 (d, *J* = 7.7 Hz, 1H, Ar-H_4), 3.85 (s, 3H, OCH_3) correspond to Ref. [9].

(*Z*)-3-[Amino(4-chlorophenyl)methylidene]-1,3-dihydro-2*H*-indol-2-one (5ac)
The crude product was purified using preparative flash liquid chromatography (silica gel, mobile phase DCM/EtOAc + 3% TEA, gradient 0–40% EtOAc during 20 min). Yellow crystals (754 mg, 93%); mp 237.5 – 238.5 °C (toluene/CH_3OH) (Ref. [1] gives 234-236 °C dec.); ^{1}H NMR (400 MHz, DMSO-d_6) δ 10.39 (bs, 1H, NH), 9.41 (bd, *J* = 3.2 Hz, 1H, ½NH_2), 8.03 (bs, 1H, ½NH_2), 7.66 (AA'XX', *J* = 8.4 Hz, 2H, Ar-H), 7.55 (AA'XX', *J* = 8.4 Hz, 2H, Ar-H), 6.78-6.88 (m, 2H, Ar-H), 6.57 (t, *J* = 7.5 Hz, 1H, Ar-H), 6.06 (d, *J* = 7.8 Hz, 1H, Ar-H_4), corresponds to Ref. [1].

(*Z*)-3-[Amino(4-iodophenyl)methylidene]-1,3-dihydro-2*H*-indol-2-one (5ac')
The crude product was purified using column chromatography (silica gel, EtOAc). Yellow solid 500 mg, 93%); mp 242 – 245 °C; ^{1}H NMR (500 MHz, $CDCl_3$) δ 9.47 (bs, 1H, ½NH_2), 7.90 (d, *J* = 8.3 Hz, 1H, Ar-H), 7.86 (bs, 1H, NH), 7.69 (AA'XX', *J* = 8,4 Hz, 2H, Ar-H), 7.46 (AA'XX', *J* = 8.4 Hz, 2H, Ar-H), 7.32 (d, *J* = 8.3 Hz, 1H, Ar-H), 6.99 (t, *J* = 7.6 Hz, 1H, Ar-H), 6.89 (d, *J* = 7.7 Hz, 1H, Ar-H), 6.72 (t, *J* = 7.6 Hz, 1H, Ar-H), 6.36 (t, *J* = 7.6 Hz, 1H, Ar-H_4), 5.13 (bs, 1H, ½NH_2); ^{13}C NMR (125 MHz, $CDCl_3$) δ (ppm): 170.7, 158.0, 157.9, 138.5, 135.6, 134.7, 132.5, 129.6, 124.9, 123.8, 120.8, 118.5, 109.1, 96.9; HRMS: calcd. for $C_{15}H_{12}IN_2O$ [M+H$^+$]: calcd. 362.9989, found 362.9989; Elemental analysis: calcd. C: 49.75, H: 3.06, N: 7.73, I: 35.00; found C: 49.64, H: 3.01, N: 7.68, I: 34.85 (for sample cryst. from CH_3OH/toluene). IR (cm^{-1}): 3349 (s), 3261 (s), 1615 (s).

(*Z*)-3-[Amino(4-trifluoromethylphenyl)methylidene]-1,3-dihydro-2*H*-indol-2-one (5ad)
The crude product was purified using preparative flash liquid chromatography (silica gel, mobile phase DCM/EtOAc + 3% TEA, gradient 0–80% EtOAc during 20 min). Yellow crystals

(884 mg, 97%); mp 244 – 245 °C (toluene) (Ref. [1] gives 233-237 °C dec.); ^{1}H NMR (400 MHz, DMSO-d_6) δ 10.43 (bs, 1H, NH), 9.43 (bs, 1H, ½NH_2), 8.09 (bs, 1H, ½NH_2), 7.97 (AA'XX', J = 8.0 Hz, 2H, Ar-H), 7.77 (AA'XX', J = 7.9 Hz, 2H, Ar-H), 6.78-6.89 (m, 2H, Ar-H), 6.55 (t, 1H, J = 7.3 Hz, Ar-H_6), 5.95 (d, J = 7.7 Hz, 1H, Ar-H_4) correspond to Ref. [1].

(*Z*)-3-[Amino(phenyl)methylidene]-5-methyl-1,3-dihydro-2*H*-indol-2-one (5ba)

The crude product was purified using column chromatography (silica gel, EtOAc/*n*-hexane 90:10). Beige solid (526 mg, 84%); mp 206 – 208 °C; ^{1}H NMR (500 MHz, $CDCl_3$) δ: 9.51 (bs, 1H, ½NH_2), 8.37 (bs, 1H, NH), 7.47 – 7.68 (m, 5H, Ar-H), 6.80 (d, J = 8.1 Hz, 1H, Ar-H), 6.10 (s, 1H, Ar-H_4), 5.23 (bs, 1H, ½NH_2), 2.08 (s, 3H, CH_3). ^{13}C NMR (125 MHz, $CDCl_3$) δ (ppm): 171.2, 159.3, 136.0, 133.5, 130.4, 129.6, 129.1, 127.8, 124.7, 124.0, 119.2, 108.8, 95.9, 21.4; HRMS: calcd. for $C_{16}H_{15}N_2O$ [M+H$^+$]: calcd. 251.1179, found 251.1175; Elemental analysis: calcd. C: 76.78, H: 5.64, N: 11.19; found C: 76.70, H: 5.65, N: 11.16 (for sample cryst. from MeCN/*n*-hexane). IR (cm^{-1}): 3354 (s), 3265 (s), 1643 (s).

(*Z*)-3-[Amino(4-methoxyphenyl)methylidene]-5-methyl-1,3-dihydro-2*H*-indol-2-one (5bb)

The crude product was purified using column chromatography (silica gel, EtOAc/*n*-hexane/MeCN 75:20:5). Yellow solid (482 mg, 69%); mp 213 – 215 °C; ^{1}H NMR (500 MHz, $CDCl_3$) δ: 9.52 (bs, 1H, ½NH_2), 8.27 (bs, 1H, NH), 7.53 (AA'XX', J = 8.6 Hz, 2H, Ar-H), 7.04 (AA'XX', J = 8.7 Hz, 2H, Ar-H), 6.79 (s, 2H, Ar-$H_{6,7}$), 6.32 (s, 1H, Ar-H_4), 5.19 (bs, 1H, ½NH_2), 3.91 (s, 3H, CH_3); ^{13}C NMR (125 MHz, $CDCl_3$) δ (ppm): 171.2, 161.3, 159.4, 133.4, 129.6, 129.5, 128.3, 124.9, 123.9, 119.1, 114.3, 108.7, 95.7, 55.5, 21.4; HRMS: calcd. for $C_{16}H_{17}N_2O_2$ [M+H$^+$]: calcd. 281.1285, found 281.1279; Elemental analysis: calcd. C: 72.84, H: 5.75, N: 9.99; found C: 72.44, H: 5.60, N: 9.58 (for sample cryst. from MeCN/EtOAc). IR (cm^{-1}): 3374 (s), 3282 (s), 1609 (s).

(*Z*)-3-[Amino(4-chlorophenyl)methylidene]-5-methyl-1,3-dihydro-2*H*-indol-2-one (5bc)

The crude product was purified using column chromatography (silica gel, EtOAc/CH_3OH 99:1). Yellow solid (520 mg, 73%); mp 246 – 247.5 °C; ^{1}H NMR (500 MHz, $CDCl_3$) δ: 9.49 (bs, 1H, ½NH_2), 8.27 (bs, 1H, NH), 7.53 (s, 4H, Ar-H), 6.80 (s, 2H, Ar-H), 6.18 (s, 1H, Ar-H_4), 5.14 (bs, 1H, ½NH_2), 2.12 (s, 3H, CH_3); ^{13}C NMR (125 MHz, $CDCl_3$) δ (ppm): 171.1, 157.7, 136.6, 134.3, 133.6, 129.84, 129.45, 129.37, 124.38, 124.29, 119.1, 108.9, 96.2, 21.4; HRMS: calcd. for $C_{16}H_{14}ClN_2O$ [M+H$^+$]: calcd. 285.0789, found 285.0784; Elemental analysis: calcd. C: 67.49, H: 4.60, N: 9.84, Cl: 12.45; found C: 67.70, H: 4.57, N: 9.58, Cl: 12.29 (for sample cryst. from MeCN/EtOAc). IR (cm^{-1}): 3362 (s), 3269 (s), 1640 (s).

(*Z*)-3-[Amino(4-trifluoromethylphenyl)methylidene]-5-methyl-1,3-dihydro-2*H*-indol-2-one (5bd)

The crude product was purified using preparative flash liquid chromatography (silica gel, mobile phase DCM/EtOAc + 3% TEA, gradient 0–80% EtOAc during 15 min). Yellow solid (891 mg, 93%); mp 234 – 236 °C; ^{1}H NMR (500 MHz, $CDCl_3$) δ: 10.31 (bs, 1H, NH), 9.38

(bd, J = 4.0 Hz, 1H, ½NH_2), 8.06 (bd, J = 4.0 Hz, 1H, ½NH_2), 7.97 (AA'XX', J = 8.1 Hz, 2H, Ar-H), 7.75 (AA'XX', J = 8.0 Hz, 2H, Ar-H), 6.65-6.61 (m, 2H, Ar-$H_{6,7}$), 5.68 (s, 1H, Ar-H_4), 1.93 (s, 3H, CH_3).; ^{13}C NMR (125 MHz, $CDCl_3$) δ (ppm): 170.3, 158.2, 139.7, 134.3, 130.4 (q, $^2J_{C-F}$ = 32.0 Hz), 129.1, 127.9, 126.0 (q, $^3J_{C-F}$ = 3.7 Hz), 124.4, 124.1 (q, $^1J_{C-F}$ = 272 Hz), 123.4, 117.9, 108.9, 94.5, 21.2.; HRMS: calcd. for $C_{17}H_{14}F_3N_2O$ [M+H$^+$]: calcd. 319.1058, found 319.1059; Elemental analysis: not measured due to fluorine content damaging column. IR (cm^{-1}): 3497 (w), 3441 (s), 1652 (s), 1605 (s).

(*Z*)-3-[Amino(phenyl)methylidene]-5-chloro-1,3-dihydro-2*H*-indol-2-one (5ca)
The crude product was purified using column chromatography (silica gel, EtOAc/*n*-hexane/THF 70:25:5). Yellow solid (373 mg, 97%); mp 192.5 – 193 °C; ^{1}H NMR (500 MHz, $CDCl_3$) δ 9.60 (bs, 1H, ½ NH_2), 9.07 (bs, 1H, CONH), 7.45 – 7.68 (m, 5H, Ar-H), 6.92 (dd, J = 8.2, 1.9 Hz, 1H, Ar-H_6), 6.83 (d, J = 8.2 Hz, 1H, Ar-H_7), 6.24 (d, J = 1.7 Hz, 1H, Ar-H_4), 5.42 (s, 1H, ½ NH_2); ^{13}C NMR (125 MHz, $CDCl_3$) δ 171.2, 160.6, 135.3, 134.1, 130.9, 129.3, 127.6, 126.1, 125.8, 123.0, 118.4, 109.9, 95.3; HRMS: calcd. for $C_{15}H_{12}ClN_2O$ [M+H$^+$]: calcd. 271.0633, found 271.0652; Elemental analysis: calcd. C: 66.55, H: 4.10, N: 10.35, Cl: 13.10; found C: 66.30, H: 3.99, N: 10.18, Cl: 13.05 (for sample cryst. from *n*-hexane/EtOAc). IR (cm^{-1}): 3356 (m), 3271 (s), 1636 (s), 1612 (s).

(*Z*)-3-[Amino(4-methoxyphenyl)methylidene]-5-chloro-1,3-dihydro-2*H*-indol-2-one (5cb)
The crude product was purified using using preparative flash liquid chromatography (silica gel, mobile phase DCM/EtOAc + 3% TEA, gradient 0–50% EtOAc during 20 min). Yellow solid (658 mg, 73%); mp 235 – 237 °C (toluene/CH_3OH); ^{1}H NMR (400 MHz, DMSO-d_6) δ: 10.48 (bs, 1H, NH), 9.54 (bd, J = 3.2 Hz, 1H, ½NH_2), 8.21 (bs, 1H, ½NH_2), 7.49 (AA'XX', J = 8.6 Hz, 2H, Ar-H), 7.16 (AA'XX', J = 8.6 Hz, 2H, Ar-H), 6.85 (dd, J = 8.2, 1.8 Hz, 2H, Ar-H_6), 6.78 (d, J = 8.2 Hz, 1H, Ar-H_7), 6.13 (d, J = 1.4 Hz, 1H, Ar-H_4), 3.87 (s, 3H, OCH_3); ^{13}C NMR (100 MHz, DMSO-d_6) δ (ppm): 170.1, 161.6, 161.0, 134.5, 129.6, 127.3, 126.8, 123.7, 121.5, 116.6, 114.4, 109.8, 93.0, 55.5; HRMS: calcd. for $C_{16}H_{14}ClN_2O_2$ [M+H$^+$]: calcd. 301.0744, found 301.0744; Elemental analysis: calcd. C: 63.90, H: 4.36, N: 9.31, Cl: 11.79; found C: 63.70, H: 4.37, N: 9.31, Cl: 11.48 (cryst. from toluene/CH_3OH). IR (cm^{-1}): 3348 (s), 1605 (s).

(*Z*)-3-[Amino(4-chlorophenyl)methylidene]-5-chloro-1,3-dihydro-2*H*-indol-2-one (5cc)
The crude product was purified using using preparative flash liquid chromatography (silica gel, mobile phase DCM/EtOAc + 3% TEA, gradient 0–40% EtOAc during 20 min). Yellow solid (824 mg, 90%); mp 281.5 – 283 °C (EtOH/H_2O); ^{1}H NMR (500 MHz, DMSO-d_6) δ: 10.55 (bs, 1H, NH), 9.51 (bd, J = 3.9 Hz, 1H, ½NH_2), 8.35 (bd, J = 3.8 Hz, 1H, ½NH_2), 7.70 (AA'XX', J = 8.5 Hz, 2H, Ar-H), 7.57 (AA'XX', J = 8.5 Hz, 2H, Ar-H), 6.87 (dd, J = 8.2, 2.1 Hz, 2H, Ar-H_6), 6.80 (d, J = 8.2 Hz, 1H, Ar-H_7), 5.93 (d, J = 2.0 Hz, 1H, Ar-H_4); ^{13}C NMR (125 MHz, DMSO-d_6) δ (ppm): 170.2, 160.4, 135.4, 135.0, 134.1, 130.1, 129.5, 126.6, 124.0, 122.1, 116.7, 110.2, 93.5; HRMS: calcd. for $C_{15}H_{11}Cl_2N_2O$ [M+H$^+$]: calcd. 305.0249, found 305.0251; Elemental analysis: calcd. C: 59.04, H: 3.30, N: 9.18, Cl: 23.24; found C: 59.41, H: 3.17, N: 8.95, Cl: 22.92 (for sample cryst. from EtOH/H_2O). IR (cm^{-1}): 3460 (s), 1609 (s).

(*Z*)-3-[Amino(4-iodophenyl)methylidene]-5-chloro-1,3-dihydro-2*H*-indol-2-one (5cc')

The crude product was purified using column chromatography (silica gel, EtOAc/*n*-hexane 90:10). Yellow solid (520 mg, 87%); mp 257 – 258 °C; ^{1}H NMR (500 MHz, $CDCl_3$) δ (ppm): 9.59 (bs, 1H, ½NH_2), 7.99 (bs, 1H, NH), 7.93 (d, *J* = 8.2 Hz, 2H, AA'XX', Ar-H), 7.31 (d, *J* = 8.2 Hz, 2H, AA'XX', Ar-H), 6.95 (dd, *J* = 8.3 and 2.0 Hz, 1H, Ar-H), 6.81 (d, *J* = 8.3 Hz, 1H, Ar-H), 6.34 (d, *J* = 1.8 Hz, 1H, Ar-H_4); 5.25 (bs, 1H, ½NH_2); ^{13}C NMR (125 MHz, $CDCl_3$) δ (ppm): 170.6, 159.0, 138.6, 134.6, 133.9, 129.3, 126.1, 125.8, 123.4, 118.4, 109.9, 97.4; HRMS: calcd. for $C_{15}H_{11}ClIN_2O$ [M+H$^+$]: calcd. 396.9599, found 396.9600; Elemental analysis: calcd. C: 45.43, H: 2.54, N: 7.06, Cl: 8.94, I: 32.00; found C: 45.12, H: 2.37; N: 6.85 (for sample cryst. from CH_3OH/*i*-PrOH). IR (cm^{-1}): 3339 (s), 1646 (s), 1603 (s).

(*Z*)-3-[Amino(4-trifluoromethylphenyl)methylidene]-5-chloro-1,3-dihydro-2*H*-indol-2-one (5cd)

The crude product was purified using using preparative flash liquid chromatography (silica gel, mobile phase DCM/EtOAc + 3% TEA, gradient 0–80% EtOAc during 15 min). Yellow solid (770 mg, 76%); mp 235 – 238 °C (toluene/CH_3OH); ^{1}H NMR (400 MHz, DMSO-d_6) δ: 10.58 (bs, 1H, NH), 9.52 (bs, 1H, ½NH_2), 8.41 (bs, 1H, ½NH_2), 8.00 (AA'XX', *J* = 8.0 Hz, 2H, Ar-H), 7.78 (AA'XX', *J* = 7.9 Hz, 2H, Ar-H), 6.87 (dd, *J* = 8.2, 1.7 Hz, 2H, Ar-H_6), 6.87 (d, *J* = 8.2 Hz, 1H, Ar-H_7), 5.76 (s, 1H, Ar-H_4); ^{13}C NMR (125 MHz, DMSO-d_6) δ (ppm): 170.0, 159.6, 139.1, 135.0, 130.7 (q, $^2J_{C\text{-}F}$ = 32.1 Hz), 129.0, 126.1 (q, $^3J_{C\text{-}F}$ = 5.0 Hz), 124.0 (q, $^1J_{C\text{-}F}$ = 270 Hz), 123.8, 122.1, 116.5, 110.0, 93.5; HRMS: calcd. for $C_{16}H_{11}ClF_3N_2O$ [M+H$^+$]: calcd. 339.0512, found 339.0516; Elemental analysis: not measured due to fluorine content damaging column. IR (cm^{-1}): 3350 (s), 1651 (s), 1606 (s).

(*Z*)-3-[Amino(phenyl)methylidene]-6-chloro-1,3-dihydro-2*H*-indol-2-one (5da)

The crude product was purified using column chromatography (silica gel, EtOAc/*n*-hexane 80:20). Yellow solid (323 mg, 84%); mp 212 – 214 °C; ^{1}H NMR (500 MHz, $CDCl_3$) δ 9.51 (bs, 1H, ½ NH_2), 9.35 (bs, 1H, NH), 7.44 – 7.65 (m, 5H, Ar-H), 6.94 (d, *J* = 1.9 Hz, 1H, Ar-H_7), 6.63 (dd, *J* = 8.3, 1.9 Hz, 1H, Ar-H_5), 6.18 (d, *J* = 8.3 Hz, 1H, Ar-H_4), 5.39 (bs, 1H, ½ NH_2); ^{13}C NMR (125 MHz, $CDCl_3$) δ 171.4, 159.9, 136.7, 135.6, 130.7, 129.3, 128.7, 127.7, 123.1, 120.4, 119.0, 109.6, 95.3; HRMS: calcd. for $C_{15}H_{12}ClN_2O$ [M+H$^+$]: calcd. 271.0633, found 271.0633; Elemental analysis: calcd. C: 66.55, H: 4.10, N: 10.35, Cl: 13.10, found C: 66.20, H: 3.95, N: 10.19, Cl: 12.89 (for sample cryst. from CH_3OH/toluene). IR (cm^{-1}): 3406 (m), 1609 (s).

(*Z*)-3-[Amino(4-methoxyphenyl)methylidene]-6-chloro-1,3-dihydro-2*H*-indol-2-one (5db)

The crude product was purified using column chromatography (silica gel, EtOAc/*n*-hexane 80:20). Yellow solid (523 mg, 87%); mp 229.5 – 231 °C; ^{1}H NMR (500 MHz, $CDCl_3$) δ: 9.50 (bs, 1H, ½NH_2), 8.38 (bs, 1H, NH), 7.50 (AA'XX', *J* = 8.7 Hz, 2H, Ar-H), 7.04 (AA'XX', *J* = 8.7 Hz, 2H, Ar-H), 6.90 (d, *J* = 1.9 Hz, 1H, Ar-H_7), 6.67 (dd, *J* = 8.3 and 1.9 Hz, 1H, Ar-H_5), 6.37 (d, *J* = 8.3 Hz, 1H, Ar-H_4), 5.28 (bs, 1H, ½NH_2), 3.91 (s, 3H, CH_3); ^{13}C NMR (125 MHz, $CDCl_3$) δ (ppm): 170.9, 161.5, 160.0, 136.2, 129.4, 128.5, 127.8, 123.4, 120.5, 119.1, 114.5,

109.4, 95.0, 55.5. HRMS: calcd. for $C_{16}H_{14}ClN_2O_2$ [M+H$^+$]: calcd. 301.0738, found 301.0744; Elemental analysis: calcd. C: 63.90, H: 4.36, N: 9.31, Cl: 11.79; found C: 63.87, H: 4.25, N: 9.09, Cl: 11.68 (for sample cryst. from CH_3OH/toluene). IR (cm^{-1}): 3406 (m), 1610 (s).

(*Z*)-3-[Amino(4-chlorophenyl)methylidene]-6-chloro-1,3-dihydro-2*H*-indol-2-one (5dc)
The crude product was purified using preparative flash liquid chromatography (silica gel, mobile phase DCM/EtOAc + 3% TEA, gradient 0–60% EtOAc during 20 min). Yellow solid (861 mg, 94%); mp 228 – 229.5 °C (CH_3OH/H_2O); ^{1}H NMR (400 MHz, DMSO-d_6) δ (ppm): 10.54 (bs, 1H, NH), 9.43 (bd, *J* = 2.6 Hz, 1H, ½NH_2), 8.23 (bs, 1H, ½NH_2), 7.65 (AA'XX', *J* = 8.4 Hz, 2H, Ar-H), 7.55 (AA'XX', *J* = 8.4 Hz, 2H, Ar-H), 6.81 (d, *J* = 1.7 Hz, 1H, Ar-H_7); 6.62 (dd, *J* = 8.3 and 1.9 Hz, 1H, Ar-H_5), 6.02 (d, *J* = 8.3 Hz, 1H, Ar-H_4); ^{13}C NMR (100 MHz, DMSO-d_6) δ (ppm): 170.1, 159.6, 137.2, 135.1, 134.1, 129.9, 129.2, 126.7, 123.4, 119.4, 118.0, 108.7, 93.2; HRMS: calcd. for $C_{15}H_{11}Cl_2N_2O$ [M+H$^+$]: calcd. 305.0249, found 305.0247; Elemental analysis: calcd. C: 59.04, H: 3.30, N: 9.18, Cl: 23.23; found C: 59.41, H: 3.19, N: 8.85, Cl: 22.97. IR (cm^{-1}): 3457 (m), 1651 (s), 1596 (s).

(*Z*)-3-[Amino(4-iodophenyl)methylidene]-6-chloro-1,3-dihydro-2*H*-indol-2-one (5dc')
The crude product was purified using column chromatography (silica gel, EtOAc/*n*-hexane/THF 70:25.5). Orange solid (510 mg, 86%); mp 256 – 260 °C; ^{1}H NMR (500 MHz, $CDCl_3$) δ (ppm): 9.46 (bs, 1H, ½NH_2), 8.09 (bs, 1H, NH), 7.90 (d, *J* = 8.2 Hz, 2H, AA'XX', Ar-H), 7.30 (d, *J* = 8.2 Hz, 2H, AA'XX', Ar-H), 6.90 (d, *J* = 1.8 Hz, 1H, Ar-H_7); 6.69 (dd, *J* = 8.3 and 1.9 Hz, 1H, Ar-H_5), 6.25 (d, *J* = 8.3 Hz, 1H, Ar-H_4), 5.20 (bs, 1H, ½NH_2); ^{13}C NMR (125 MHz, $CDCl_3$) δ (ppm): 170.7, 158.3, 138.6, 136.4, 134.9, 129.4, 129.1, 122.8, 120.7, 119.1, 109.5, 97.1, 95.6; HRMS: calcd. for $C_{15}H_{11}ClIN_2O$ [M+H$^+$]: calcd. 396.9599, found 396.9605; Elemental analysis: calcd. C: 45.43, H: 2.54, N: 7.06, Cl: 8.94, I: 32.00; found C: 45.33, H: 2.39, N: 6.90 (for sample cryst. from CH_3OH/ toluene and triturated with *n*-hexane). IR (cm^{-1}): 3452 (m), 1662 (s), 1605 (s).

(*Z*)-3-[Amino(4-trifluoromethylphenyl)methylidene]-6-chloro-1,3-dihydro-2*H*-indol-2-one (5dd)
The crude product was purified using preparative flash liquid chromatography (silica gel, mobile phase DCM/EtOAc + 3% TEA, gradient 0–60% EtOAc during 20 min). Yellow solid (796 mg, 78%); mp 292 – 295 °C (toluene/CH_3OH); ^{1}H NMR (400 MHz, DMSO-d_6) δ (ppm): 10.59 (bs, 1H, NH), 9.46 (bs, 1H, ½NH_2), 8.29 (bs, 1H, ½NH_2), 7.96 (AA'XX', *J* = 8.0 Hz, 2H, Ar-H), 7.76 (AA'XX', *J* = 8.0 Hz, 2H, Ar-H), 6.82 (s, 1H, Ar-H_7); 6.60 (d, *J* = 8.3 Hz, 1H, Ar-H_5), 5.91 (d, *J* = 8.4 Hz, 1H, Ar-H_4); ^{13}C NMR (100 MHz, DMSO-d_6) δ (ppm): 170.1, 159.1, 139.3, 137.4, 130.6 (q, $^2J_{C-F}$ = 32.1 Hz), 129.0, 126.9, 126.1 (q, $^3J_{C-F}$ = 3.7 Hz), 124.1 ($^1J_{C-F}$ = 271 Hz), 123.2, 119.4, 117.9, 108.8, 93.4; HRMS: calcd. for $C_{16}H_{11}ClF_3N_2O$ [M+H$^+$]: calcd. 339.0512, found 339.0518; Elemental analysis: not measured due to fluorine content damaging column. IR (cm^{-1}): 3497 (m), 1663 (s), 1605 (s).

(*Z*)-3-[Amino(phenyl)methylidene]-5-nitro-1,3-dihydro-2*H*-indol-2-one (5ea)
Yellow solid (445 mg, 79%); mp 295 - 298 °C; ^{1}H NMR (500 MHz, DMSO-d$_6$) δ: 11.12 (bs, 1H, NH), 9.56 (bd, *J* = 3.7 Hz, 1H, ½NH$_2$), 8.69 (bd, *J* = 3.4 Hz, 1H, ½NH$_2$), 7.81 (dd, *J* = 8.6 and 2.4 Hz, 1H, Ar-H$_6$), 7.70 (m, 1H, Ar-H$_p$), 7.64 (m, 2H, Ar-H$_m$), 7.57 (m, 2H, Ar-H$_o$), 6.95 (d, *J* = 8.6 Hz, 1H, Ar-H$_7$), 6.84 (d, *J* = 2.3 Hz, 1H, Ar-H$_4$); ^{13}C NMR (125 MHz, DMSO-*d*$_6$) δ (ppm): 170.5, 162.9, 141.4, 140.7, 134.6, 130.8, 129.3, 127.8, 125.3, 119.0, 112.0, 108.4, 92.5; HRMS: calcd. for $C_{15}H_{11}N_3O_3Na$ [M+Na$^+$]: calcd. 304.0698, found 304.0696; Elemental analysis: calcd. C: 64.05, H: 3.94, N: 14.94; found C: 64.20, H: 3.90, N: 14.55. IR (cm^{-1}): 3430 (s), 1661 (s), 1616 (s).

(*Z*)-3-[Amino(4-methoxyphenyl)methylidene]-5-nitro-1,3-dihydro-2*H*-indol-2-one (5eb)
The crude product was purified using flash chromatography (silica gel, $CHCl_3$/CH_3OH; gradient changing from 99:1 to 90:10 during 25 min). Yellow solid (455 mg, 73%); mp 328 - 331 °C; ^{1}H NMR (500 MHz, $CDCl_3$) δ: 11.11 (bs, 1H, NH), 9.56 (bs, 1H, ½NH$_2$), 8.58 (bs, 1H, ½NH$_2$), 7.82 (dd, *J* = 8.6 and 2.3 Hz, 1H, Ar-H$_6$), 7.53 (AA'XX', *J* = 8.6 Hz, 2H, Ar-H$_{3',5'}$), 7.18 (AA'XX', *J* = 8.7 Hz, 2H, Ar-H$_{2',6'}$), 7.09 (d, *J* = 2.0 Hz, 1H, Ar-H$_4$), 6.95 (d, *J* = 8.6 Hz, 1H, Ar-H$_7$), 3.88 (s, 3H, OCH$_3$); ^{13}C NMR (125 MHz, DMSO-*d*$_6$) δ (ppm): 170.9, 163.3, 161.6, 141.5, 141.0, 130.0, 127.0, 125.8, 119.2, 114.9, 112.4, 108.7, 92.7, 55.9; HRMS: calcd. for $C_{16}H_{14}N_3O_4$ [M+H$^+$]: calcd. 312.0979, found 312.0981; Elemental analysis: calcd. C: 61.73, H: 4.21, N: 13.50; found C: 61.68, H: 4.17, N: 13.44 (for sample cryst. from EtOH/toluene). IR (cm^{-1}): 3464 (m), 1659 (s), 1605 (s).

(*Z*)-3-[Amino(4-chlorophenyl)methylidene]-5-nitro-1,3-dihydro-2*H*-indol-2-one (5ec)
Yellow solid (489 mg, 78%); mp >372 °C (dec.); ^{1}H NMR (500 MHz, $CDCl_3$) δ: 11.14 (bs, 1H, NH), 9.52 (bd, *J* = 3.6 Hz, 1H, ½NH$_2$), 8.69 (bd, *J* = 3.3 Hz, 1H, ½NH$_2$), 7.82 (dd, *J* = 8.6 and 2.3 Hz, 1H, Ar-H), 7.53 (AA'XX', *J* = 8.6 Hz, 2H, Ar-H), 7.82 (dd, *J* = 8.6 and 2.3 Hz, 1H, Ar-H$_6$), 7.72 (AA'XX', *J* = 8.5 Hz, 2H, Ar-H), 7.61 (AA'XX', *J* = 8.5 Hz, 2H, Ar-H), 6.95 (d, *J* = 8.7 Hz, 1H, Ar-H$_7$), 6.87 (d, *J* = 2.3 Hz, 1H, Ar-H$_4$); ^{13}C NMR (125 MHz, DMSO-*d*$_6$) δ (ppm): 170.6, 161.6, 141.7, 140.9, 135.7, 133.6, 130.1, 129.6, 125.3, 119.4, 112.1, 108.7, 92.8; HRMS: calcd. for $C_{15}H_{11}ClN_3O_3$ [M+H$^+$]: calcd. 316.0489, found 316.0490; Elemental analysis: calcd. C: 57.07, H: 3.19, N: 13.31; found C: 57.13, H: 3.10, N: 13.10. IR (cm^{-1}): 3454 (s), 1652 (s), 1609 (s).

(*Z*)-3-[Amino(4-trifluoromethylphenyl)methylidene]-5-nitro-1,3-dihydro-2*H*-indol-2-one (5ed)
The crude product was purified using flash chromatography (silica gel, *n*-hexane/EtOAc; gradient changing from 85:15 to 20:80 during 20 min). Yellow solid (674 mg, 97%); mp 345 °C (decomp.); ^{1}H NMR (500 MHz, $CDCl_3$) δ: 11.18 (bs, 1H, NH), 9.50 (bs, 1H, NH), 8.78 (s, 1H, NH), 8.03 (AA'XX', *J* = 8.1 Hz, 2H, Ar-H), 7.78 – 7.85 (m, 4H, Ar-H, AA'XX' + Ar-H), 6.96 (d, *J* = 8.6 Hz, 1H, Ar-H), 6.63 (d, *J* = 2.1 Hz, 1H, Ar-H$_4$); ^{1}H NMR (125 MHz, DMSO-*d*$_6$) δ (ppm): 170.3, 160.9, 141.6, 140.7, 138.6, 130.9 (q, $^{2}J_{C–F}$ = 32.1 Hz), 128.9, 126.3, 124.9, 124.0 (q, $^{1}J_{C-F}$ = 272.5 Hz), 119.3, 111.8, 108.5, 92.8; HRMS: calcd. for $C_{16}H_{11}F_3N_3O_3$ [M+H$^+$]:

calcd. 350.0747, found 350.0748; Elemental analysis: not measured due to fluorine content damaging analyzer. IR (cm^{-1}): 3478 (m), 3403 (m), 1667 (s), 1609 (m).

(*Z*)-3-[Phenyl(phenylamino)methylidene]-1,3-dihydro-2*H*-indol-2-one (6aa)
The crude product was purified using column chromatography (neutral alumina, *n*-hexane/EtOAc 3:2). Yellow solid (721 mg, 77%); mp 322.5 – 325 °C; ^{1}H NMR (500 MHz, DMSO-d_6) δ 12.01 (bs, 1H, NH), 10.73 (bs, 1H, NH), 7.52 – 7.61 (m, 3H, Ar-H), 7.44 – 7.51 (m, 2H, Ar-H), 7.14 (t, *J* = 7.7 Hz, 2H, Ar-H), 6.98 (t, *J* = 7.3 Hz, 1H, Ar-H), 6.90 (t, *J* = 7.5 Hz, 1H, Ar-H_6), 6.85 (d, *J* = 7.5 Hz, 1H, Ar-H_7), 6.77 – 6.82 (m, 2H, Ar-H), 6.53 (t, *J* = 7.5 Hz, 1H, Ar-H_5), 5.77 (d, *J* = 7.8 Hz, 1H, Ar-H_4); ^{13}C NMR (125 MHz, DMSO-d_6) δ (ppm): 170.3, 155.5, 138.3, 136.9, 132.5, 131.8, 130.3, 129.6, 128.7, 124.7, 124.0, 123.8, 120.2, 118.3, 116.4, 109.3, 98.7; HRMS: calcd. for $C_{21}H_{17}N_2O$ [M+H$^+$]: calcd. 313.1341, found 313.1343; Elemental analysis: calcd. C: 80.75, H: 5.16, N: 8.97; found C: 80.80, H: 5.09, N: 8.74 (for sample cryst. from EtOAc/EtOH). IR (cm^{-1}): 3083 (m), 1641 (s), 1607 (s).

(*Z*)-3-[4-Methoxyphenylamino(phenyl)methylidene]-1,3-dihydro-2*H*-indol-2-one (6ab)
The crude product was purified using column chromatography (neutral alumina, DCM/*i*-PrOH/conc. NH_3 20:1:0.1). Yellow solid (827 mg, 88%); mp 271 – 273 °C; ^{1}H NMR (400 MHz, DMSO-d_6) δ 11.89 (bs, 1H, NH), 10.66 (bs, 1H, NH), 7.48 – 7.56 (m, 3H, Ar-H), 7.39 – 7.47 (m, 2H, Ar-H), 6.79 – 6.91 (m, 4H, Ar-H), 6.72 (AA'XX', *J* = 9.0 Hz, 2H, Ar-H), 6.52 (dt, *J* = 7.3 and 1.2 Hz, 1H, Ar-H), 5.74 (d, *J* = 7.8 Hz, 1H, Ar-H_4), 3.65 (s, 3H, OCH_3); ^{13}C NMR (100 MHz, DMSO-d_6) δ (ppm): 170.2, 157.2, 156.4, 136.4, 132.8, 131.4, 129.9, 129.3, 128.6, 125.2, 124.2, 123.2, 119.9, 117.8, 114.1, 109.1, 96.9, 55.2; HRMS: calcd. for $C_{22}H_{19}N_2O_2$ [M+H$^+$]: calcd. 343.1441, found 343.1448; Elemental analysis: calcd. C: 77.17, H: 5.30, N: 8.18; found C: 77.56, H: 5.25, N: 8.20. IR (cm^{-1}): 3063 (m), 1641 (s), 1607 (s).

(*Z*)-3-[4-Chlorophenylamino(phenyl)methylidene]-1,3-dihydro-2*H*-indol-2-one (6ac)
The crude product was purified using column chromatography (neutral alumina, DCM/*i*-PrOH/conc. NH_3 10:1:0.1). Yellow solid (503 mg, 48%); mp 286.5 – 288 °C; ^{1}H NMR (400 MHz, DMSO-d_6) δ 11.95 (bs, 1H, NH), 10.75 (bs, 1H, NH), 7.51 – 7.62 (m, 3H, Ar-H), 7.41 – 7.51 (m, 2H, Ar-H), 7.18 (AA'X'X', *J* = 8.8 Hz, 2H, Ar-H), 6.91 (t, *J* = 7.5 Hz, 1H, Ar-H_6), 6.85 (d, *J* = 7.5 Hz, 1H, Ar-H_7), 6.81 (AA'X'X', *J* = 8.8 Hz, 2H, Ar-H), 6.54 (t, *J* = 7.5 Hz, 1H, Ar-H_5), 5.81 (d, *J* = 7.7 Hz, 1H, Ar-H_4); ^{13}C NMR (100 MHz, DMSO-d_6) δ (ppm): 170.3, 155.7, 137.8, 136.9, 132.5, 130.3, 129.6, 128.9, 128.7, 128.3, 124.4, 123.96, 123.87, 120.2, 118.3, 109.4, 98.6; HRMS: calcd. for $C_{21}H_{16}ClN_2O$ [M+H$^+$]: calcd. 347.0946, found 347.0953; Elemental analysis: calcd. C: 72.73, H: 4.36, N: 8.08, Cl: 10.22; found C: 72.76, H: 4.33, N: 7.99, Cl: 9.93. IR (cm^{-1}): 3087 (m), 1642 (s), 1613 (s).

(*Z*)-3-[Phenyl(4-trifluoromethylphenylamino)methylidene]-1,3-dihydro-2*H*-indol-2-one (6ad)
The crude product was purified using column chromatography (neutral alumina, DCM/*i*-PrOH/conc. NH_3 20:1:0.1). Yellow solid (488 mg, 43%); mp 282 – 284 °C; ^{1}H NMR (400

MHz, DMSO-d_6) δ 12.11 (bs, 1H, NH), 10.83 (bs, 1H, NH), 7.56 – 7.67 (m, 3H, Ar-H), 7.50 – 7.55 (m, 2H, Ar-H), 7.47 (AA'XX', *J* = 8.6 Hz, 2H, Ar-H), 6.94 (t, *J* = 7.5 Hz, 1H, Ar-H_6), 6.84 – 6.91 (m, 3H, Ar-H), 6.56 (t, *J* = 7.6 Hz, 1H, Ar-H_5), 5.85 (d, *J* = 7.9 Hz, 1H, Ar-H_4); ^{13}C NMR (100 MHz, DMSO-d_6) δ (ppm): 170.3, 154.5, 142.7, 137.3, 132.5, 130.6, 129.8, 128.6, 126.1 (q, *J* = 3.6 Hz), 124.3 (q, *J* = 272 Hz, CF_3), 124.5, 123.6, 123.6 (q, *J* = 32.1 Hz), 121.7, 120.3, 118.7, 109.5, 100.1; HRMS: calcd. for $C_{22}H_{16}F_3N_2O$ [M+H$^+$]: calcd. 381.1209, found 381.1216; Elemental analysis: not measured due to fluorine content damaging column. IR (cm^{-1}): 3149 (m), 1650 (m), 1611 (s).

(*Z*)-3-[4-Nitrophenylamino(phenyl)methylidene]-1,3-dihydro-2*H*-indol-2-one (6ae)

The crude product was purified using column chromatography (silica gel, DCM/CH_3OH/TEA 20:1:0.05). Orange solid (339 mg, 32%); mp 281 – 283.5 °C; ^{1}H NMR (400 MHz, DMSO-d_6) δ 12.19 (bs, 1H, NH), 10.89 (bs, 1H, NH), 7.97 (AA'XX', *J* = 9.1 Hz, 2H, Ar-H), 7.60 – 7.70 (m, 3H, Ar-H), 7.53 – 7.59 (m, 2H, Ar-H), 6.97 (t, *J* = 7.6 Hz, 1H, Ar-H_6), 6.87 (d, *J* = 7.7 Hz, 1H, Ar-H_7), 6.82 (AA'XX', *J* = 9.1 Hz, 2H, Ar-H), 6.58 (t, *J* = 7.6 Hz, 1H, Ar-H_5), 5.88 (d, *J* = 7.7 Hz, 1H, Ar-H_4); ^{13}C NMR (100 MHz, DMSO-d_6) δ (ppm): 170.2, 153.0, 145.6, 142.0, 137.8, 132.4, 130.9, 130.0, 128.6, 125.2, 125.0, 123.3, 120.52, 120.48, 119.1, 109.7, 102.0; HRMS: calcd. for $C_{21}H_{16}N_3O_3$ [M+H$^+$]: calcd. 358.1186, found 358.1195; Elemental analysis: calcd. for hemihydrate C: 68.84, H: 4.40, N: 11.47; found C: 68.82, H: 4.37, N: 11.25. IR (cm^{-1}): 3100 (m), 1645 (s), 1583 (s).

(*Z*)-3-[Methylamino(phenyl)methylidene]-1,3-dihydro-2*H*-indol-2-one (6af)

The crude product was purified using column chromatography (silica gel, EtOAc/*n*-hexane 4:1). Yellow solid (475 mg, 76%); mp 256 – 258 °C; ^{1}H NMR (400 MHz, DMSO-d_6) δ 10.40 (bs, 1H, NH), 10.09 (d, *J* = 5.0 Hz, 1H, NH), 7.59 – 7.65 (m, 3H, Ar-H), 7.34 – 7.47 (m, 2H, Ar-H), 6.73 – 6.83 (m, 2H, Ar-H), 6.40 – 6.50 (m, 1H, Ar-H), 5.52 (d, *J* = 7.7 Hz, 1H, Ar-H), 2.74 (d, *J* = 4.8 Hz, 3H, CH_3); ^{13}C NMR (100 MHz, DMSO-d_6) δ 170.0, 161.9, 135.7, 132.9, 129.9, 129.6, 127.5, 125.0, 122.0, 119.6, 116.7, 108.8, 94.1, 30.5; HRMS: calcd. for $C_{16}H_{15}N_2O$ [M+H$^+$]: calcd. 251.1184, found 251.1191; Elemental analysis: calcd. C: 76.78, H: 5.64, N: 11.19; found C: 76.46, H: 5.54, N: 11.11 (for sample cryst. from toluene/CH_3OH). IR (cm^{-1}): 3139 (m), 1634 (s), 1610 (s).

(*Z*)-3-[*n*-Pentylamino(phenyl)methylidene]-1,3-dihydro-2*H*-indol-2-one (6ag)

The crude product was purified using column chromatography (neutral alumina, DCM/i-PrOH/conc. NH_3 20:1:0.1). Yellow solid (652 mg, 71%); mp 214 – 216 °C; ^{1}H NMR (400 MHz, $CDCl_3$) δ 10.21 (t, *J* = 5.6 Hz, 1H, NH), 9.29 (bs, 1H, CONH), 7.52 – 7.63 (m, 3H, Ar-H), 7.34 – 7.45 (m, 2H, Ar-H), 6.84 – 6.98 (m, 2H, Ar-H), 6.54 – 6.61 (m, 1H, Ar-H), 5.68 (d, *J* = 7.8 Hz, 1H, Ar-H_4), 3.11 (dd, *J* = 13.5, 6.8 Hz, 2H, NCH_2), 1.51 – 1.63 (m, 2H, CH_2), 1.19 – 1.38 (m, 4H, 2×CH_2), 0.87 (t, *J* = 7.0 Hz, 3H, CH_3); ^{13}C NMR-APT (100 MHz, $CDCl_3$) δ (ppm): 170.8 (C=O), 161.9 (C_q), 135.0 (C_q), 133.2 (C_q), 129.6 (C-H), 129.3 (C-H), 127.6 (C-H), 125.2 (C_q), 122.1 (C-H), 120.2 (C-H), 117.4 (C-H), 109.0 (C-H), 94.6 (C_q), 43.9 (CH_2), 30.4 (CH_2), 28.7 (CH_2), 22.2 (CH_2), 13.9 (CH_3); HRMS: calcd. for $C_{20}H_{21}N_2O$ [M+H$^+$]: calcd.

307.1805, found 307.1809; Elemental analysis: calcd. for hemihydrate C: 78.40, H: 7.24, N: 9.14; found C: 78.40, H: 7.27, N: 8.98. IR (cm^{-1}): 3137 (m), 1643 (s), 1589 (s).

(*Z*)-3-[Benzylamino(phenyl)methylidene]-1,3-dihydro-2*H*-indol-2-one (6ah)

The crude product was purified using column chromatography (neutral alumina, DCM/i-PrOH/conc. NH_3 30:1:0.1). Light yellow solid (696 mg, 71%); mp 231 – 234 °C; ^{1}H NMR (400 MHz, $CDCl_3$) δ 10.52 (bt, *J* = 5.8 Hz, 1H, NH), 8.85 and 8.91 (2×bs, 1H, NH), 7.40 – 7.59 (m, 3H, Ar-H), 7.28 – 7.37 (m, 4H, Ar-H), 7.23 – 7.28 (m, 1H, Ar-H), 7.17 – 7.23 (m, 2H, Ar-H), 6.85 – 6.93 (m, 2H, Ar-H), 6.58 (dt, *J* = 7.0 and 1.9 Hz, 1H, Ar-H), 5.70 (d, *J* = 7.8 Hz, 1H, Ar-H_4), 4.34 (d, *J* = 6.5 Hz, 2H, CH_2); ^{13}C NMR-APT (100 MHz, $CDCl_3$) δ (ppm): 170.8, 161.6, 138.3, 135.1, 132.7, 129.8, 129.4, 128.7, 127.7, 127.4, 126.8, 125.1, 122.6, 120.4, 117.7, 109.1, 95.6, 47.6; HRMS: calcd. for $C_{22}H_{19}N_2O$ [M+H$^+$]: calcd. 327.1492, found 327.1498; Elemental analysis: calcd. C: 80.96, H: 5.56, N: 8.58; found C: 81.30, H: 5.41, N: 8.35 (for sample cryst. from CH_3OH/H_2O). IR (cm^{-1}): 3131 (m), 1640 (s), 1606 (s).

(*Z*)-3-[Cyclohexylamino(phenyl)methylidene]-1,3-dihydro-2*H*-indol-2-one (6ai)

The crude product was purified using column chromatography (neutral alumina, DCM/i-PrOH/conc. NH_3 20:1:0.1). Yellowish solid (613 mg, 64%); mp 343 – 346 °C; ^{1}H NMR (400 MHz, DMSO-d_6) δ 10.47 (bs, 1H, NH), 10.41 (d, *J* = 9.6 Hz, 1H, NH), 7.66 – 7.71 (m, 3H, Ar-H), 7.47 – 7.51 (m, 2H, Ar-H), 6.80 – 6.85 (m, 2H, Ar-H), 6.44 – 6.53 (m, 1H, Ar-H), 5.47 (d, *J* = 7.7 Hz, 1H, Ar-H_4), 3.05 - 3.16 (m, 1H, NCH), 1.76 - 1.84 (m, 2H, 2×½CH_2), 1.63 – 1.72 (m, 2H, 2×½CH_2), 1.07 – 1.52 (m, 6H, 3 × CH_2); ^{13}C NMR-APT (100 MHz, DMSO-d_6) δ (ppm): 169.9, 160.1, 135.6, 133.0, 129.9, 129.6, 127.4, 124.8, 122.0, 119.5, 116.6, 108.7, 94.0, 51.4, 33.7, 24.8, 23.9; HRMS: calcd. for $C_{21}H_{23}N_2O$ [M+H$^+$]: calcd. 319.1805, found 319.1810; Elemental analysis: calcd. C: 79.21, H: 6.96, N: 8.80; found C: 79.31, H: 6.99, N: 8.69. IR (cm^{-1}): 3137 (m), 1638 (s), 1605 (s), 1582 (s).

(*Z*)-5-Methyl-3-[phenyl(phenylamino)methylidene]-1,3-dihydro-2*H*-indol-2-one (6ba)

The crude product was purified using column chromatography (silica gel, DCM/CH_3OH/TEA 20:1:0.05). Yellow solid (674 mg, 69%); mp 258 – 261 °C; ^{1}H NMR (400 MHz, DMSO-d_6) δ 12.03 (bs, 1H, CONH), 10.62 (bs, 1H, NH), 7.52 – 7.59 (m, 3H, Ar-H), 7.46 (dd, *J* = 7.5 and 1.3 Hz, 2H, Ar-H), 7.13 (t, *J* = 7.8 Hz, 2H, Ar-H), 6.97 (t, *J* = 7.4 Hz, 1H, Ar-H), 6.80 (d, *J* = 7.8 Hz, 2H, Ar-H), 6.73 (q, *J* = 7.9 Hz, 2H, Ar-H), 5.60 (s, 1H, Ar-H_4), 1.92 (s, 3H, CH_3); ^{13}C NMR (100 MHz, DMSO-d_6) δ (ppm): 170.4, 155.9, 138.8, 134.6, 132.9, 130.1, 129.4, 129.0, 128.66, 128.29, 124.22, 124.16, 124.13, 122.6, 119.04, 119.01, 108.9, 98.2, 21.2; HRMS: calcd. for $C_{22}H_{19}N_2O$ [M+H$^+$]: calcd. 327.1492, found 327.1501; Elemental analysis: calcd. C: 80.96, H: 5.56, N: 8.58; found C: 81.10, H: 5.52, N: 8.58. IR (cm^{-1}): 3222 (m), 1641 (s), 1626 (s).

(*Z*)-5-Chloro-3-[phenyl(phenylamino)methylidene]-1,3-dihydro-2*H*-indol-2-one (6ca)

The crude product was purified using column chromatography (silica gel, DCM/CH_3OH 99:1). Yellow solid (380 mg, 89%); mp 242 - 243 °C; ^{1}H NMR (500 MHz, $CDCl_3$) δ 11.98 (bs, 1H, NH), 8.62 (bs, 1H, NH), 7.50 – 7.59 (m, 3H, Ar-H), 7.39 – 7.43 (m, 2H, Ar-H), 7.13 (t, *J* = 7.9

Hz, 2H, Ar-H), 7.01 (t, *J* = 7.3 Hz, 1H, Ar-H), 6.92 (dd, *J* = 8.2 and 2.0 Hz, 1H, Ar-H_6), 6.84 (d, *J* = 8.3 Hz, 1H, Ar-H_7), 6.78 – 6.82 (m, 2H, Ar-H), 5.88 (d, *J* = 1.9 Hz, 1H, Ar-H_4); ^{13}C NMR (125 MHz, $CDCl_3$) δ (ppm): 170.7, 158.2, 138.4, 133.8, 132.4, 130.3, 129.5, 128.8, 128.5, 126.14, 126.09, 124.8, 123.3, 123.1, 118.8, 109.9, 97.1; HRMS: calcd. for $C_{21}H_{16}ClN_2O$ [M+H$^+$]: calcd. 347.0946, found 347.0958; Elemental analysis: calcd. C: 72.73, H: 4.36, N: 8.08, Cl: 10.22; found C: 72.28, H: 4.19, N: 7.97, Cl: 10.52 (for sample cryst. from EtOAc/*n*-hexane). IR (cm^{-1}): 3148 (m), 1645 (s), 1608 (s).

(*Z*)-6-Chloro-3-[phenyl(phenylamino)methylidene]-1,3-dihydro-2*H*-indol-2-one (6da)
The crude product was purified using column chromatography (neutral alumina, DCM/*i*-PrOH/conc. NH_3 20:1:0.1). Yellow solid (749 mg, 72%); mp 319 – 322 °C; ^{1}H NMR (400 MHz, DMSO-d_6) δ 11.98 (bs, 1H, NH), 10.87 (bs, 1H, NH), 7.51 – 7.59 (m, 3H, Ar-H), 7.46 (m, 2H, Ar-H), 7.15 (t, *J* = 7.8 Hz, 2H, Ar-H), 7.00 (t, *J* = 7.3 Hz, 1H, Ar-H), 6.86 (d, *J* = 1.8 Hz, 1H, Ar-H_7), 6.82 (d, *J* = 7.9 Hz, 2H, Ar-H), 6.60 (dd, *J* = 8.3 and 1.8 Hz, 1H, Ar-H_5), 5.71 (d, *J* = 8.3 Hz, 1H, Ar-H_4); ^{13}C NMR (100 MHz, DMSO-d_3) δ (ppm): 170.2, 156.9, 138.4, 137.7, 132.5, 130.3, 129.5, 129.0, 128.5, 127.6, 124.6, 123.0, 123.0, 119.7, 118.9, 109.1, 97.1; HRMS: calcd. for $C_{21}H_{16}ClN_2O$ [M+H$^+$]: calcd. 347.0946, found 347.0954; Elemental analysis: calcd. C: 72.73, H: 4.36, N: 8.08, Cl: 10.22; found C: 72.82, H: 4.36, N: 8.09, Cl: 10.01 (for sample cryst. from EtOAc/*n*-hexane). IR (cm^{-1}): 3064 (m), 1646 (s), 1605 (s).

(*Z*)-5-Nitro-3-[phenyl(phenylamino)methylidene]-1,3-dihydro-2*H*-indol-2-one (6ea)
The crude product was purified using flash chromatography (silica gel, DCM/EtOAc + 3% TEA; gradient changing from 0–50% EtOAc during 20 min). Orange solid (529 mg, 74%); mp 267 – 269 °C; ^{1}H NMR: 11.98 (vbs, 2H, 2× NH), 7.85 (dd, *J* = 8.6 and 2.2 Hz, 1H, Ar-H_6), 7.54 – 7.67 (m, 3H, Ar-H), 7.50 (d, *J* = 7.0 Hz, 2H, Ar-H_o, 7.18 (d, *J* = 7.7 Hz, 2H, Ar-H_m), 7.05 (t, *J* = 7.6 Hz, 1H, Ar-H_p), 7.00 (d, *J* = 8.7 Hz, 1H, Ar-H_7), 6.91 (d, *J* = 7.7 Hz, 2H, Ar-H_o), 6.60 (s, 1H, Ar-H_4). ^{13}C NMR (100 MHz, DMSO-d_6,-60 °C) δ (ppm): 170.5, 158.6, 141.8, 141.0, 138.0, 131.8, 130.2, 129.3, 128.7, 128.2, 124.9, 124.6, 123.3, 119.5, 112.8, 108.6, 96.4 HRMS: calcd. for $C_{21}H_{15}NaN_3O_3$ [M+Na$^+$]: calcd. 380.1011, found 380.1016; Elemental analysis: calcd. C: 70.58, H: 4.23, N: 11.76; found C: 70.79, H: 4.22, N: 11.61. IR (cm^{-1}): 3351 (m), 1654 (s), 1622 (m). IR (cm^{-1}): 3351 (m), 1654 (s), 1622 (m).

(*Z*)-5-Methyl-3-[methylamino(phenyl)methylidene]-1,3-dihydro-2*H*-indol-2-one (6bf)
The crude product was purified using column chromatography (neutral alumina, DCM/*i*-PrOH/conc. NH_3 25:1:0.1). Beige solid (521 mg, 66%); mp 255 – 257.5 °C; ^{1}H NMR (400 MHz, DMSO-d_6) δ 10.27 (bs, 1H, NH), 10.06 (bq, *J* = 5.1 Hz, 1H, NH), 7.58 - 7.67 (m, 3H, Ar-H), 7.37 – 7.44 (m, 2H, Ar-H), 6.66 (d, *J* = 7.8 Hz, 1H, Ar-H_7), 6.59 (d, *J* = 7.8 Hz, 1H, Ar-H_6), 5.31 (s, 1H, Ar-H_4), 2.74 (d, *J* = 5.1 Hz, 3H, NCH_3), 1.87 (s, 3H, CH_3); ^{13}C NMR (100 MHz, DMSO-d_6) δ (ppm): 170.1, 161.6, 133.6, 133.0, 129.7, 129.6, 127.6, 127.5, 125.1, 122.5, 117.5, 108.4, 94.2, 30.4, 21.3; HRMS: calcd. for $C_{17}H_{17}N_2O$ [M+H$^+$]: calcd. 265.1335, found 265.1339; Elemental analysis: calcd. C: 77.25, H: 6.10, N: 10.60; found C: 77.36, H: 6.08, N: 10.43 (for sample cryst. from $CH_3OH/CHCl_3$. IR (cm^{-1}): 3137 (m), 1628 (s), 1607 (s).

(*Z*)-5-Chloro-3-[methylamino(phenyl)methylidene]-1,3-dihydro-2*H*-indol-2-one (6cf)
The crude product was purified using column chromatography (neutral alumina, DCM/*i*-PrOH/conc. NH_3 30:1:0.1). Yellow solid (605 mg, 71%); mp 256 – 258 °C; ^{1}H NMR (400 MHz, DMSO-d_6) δ 10.54 (bs, NH, 1H), 10.17 (bq, *J* = 5.1 Hz, 1H, NH), 7.61 – 7.71 (m, 3H, Ar-H), 7.39 – 7.46 (m, 2H, Ar-H), 6.72 – 6.81 (m, 2H, Ar-H), 5.36 (d, *J* = 1.3 Hz, 1H, Ar-H_4), 2.77 (d, *J* = 5.1 Hz, 3H, NCH_3); ^{13}C NMR (100 MHz, DMSO-d_6) δ (ppm): 169.8, 162.8, 134.2, 132.5, 130.1, 129.8, 127.3, 126.8, 123.8, 121.2, 116.1, 109.8, 93.5, 30.6; HRMS: calcd. for $C_{16}H_{14}ClN_2O$ [M+H$^+$]: calcd. 285.0789, found 285.0793; Elemental analysis: calcd. C: 67.49, H: 4.60, N: 9.84, Cl: 12.45; found C: 67.30, H: 4.57, N: 10.08, Cl: 12.38 (for sample cryst. from CH_3OH/H_2O.

(*Z*)-6-Chloro-3-[methylamino(phenyl)methylidene]-1,3-dihydro-2*H*-indol-2-one (6df)
The crude product was purified using column chromatography (neutral alumina, DCM/*i*-PrOH/conc. NH_3 15:1:0.1). Yellow solid (719 mg, 84%); mp 274 – 276.5 °C; ^{1}H NMR (400 MHz, DMSO-d_6) δ 10.54 (bs, 1H, NH), 10.10 (bq, *J* = 5.1 Hz, 1H, NH), 7.57 – 7.71 (m, 3H, Ar-H), 7.38 – 7.45 (m, 2H, Ar-H), 6.77 (d, *J* = 1.9 Hz, 1H, Ar-H_7), 6.49 (dd, *J* = 8.3 and 2.0 Hz, 1H, Ar-H_6), 5.44 (d, *J* = 8.3 Hz, 1H, Ar-H_4), 2.75 (d, *J* = 5.1 Hz, 3H, NCH_3); ^{13}C NMR (100 MHz, DMSO-d_6) δ (ppm): 169.8 162.4, 136.6, 132.6, 130.1, 129.7, 127.4, 126.0, 124.0, 119.2, 117.3, 108.6, 93.6, 30.6; HRMS: calcd. for $C_{16}H_{14}ClN_2O$ [M+H$^+$]: calcd. 285.0789, found 285.0795; Elemental analysis: calcd. C: 67.49, H: 4.60, N: 9.84, Cl: 12.45; found C: 67.76, H: 4.56, N: 10.01, Cl: 12.27. IR (cm^{-1}): 3143 (m), 1641 (s), 1608 (s), 1591 (s).

(*Z*)-3-[Methylamino(phenyl)methylidene]-5-nitro-1,3-dihydro-2*H*-indol-2-one (6ef)
The crude product was purified using column chromatography (neutral alumina, DCM/*i*-PrOH/conc. NH_3 15:1:0.1). Orange solid (656 mg, 74%); mp 332.5 – 335.5 °C; ^{1}H NMR (400 MHz, DMSO-d_6) δ: 11.12 (bs, 1H, CONH), 10.21 (bq, *J* = 5.1 Hz, 1H, NH), 7.76 (dd, *J* = 8.6 and 2.3 Hz, 1H, Ar-H_6), 7.65 – 7.73 (m, 3H, Ar-H), 7.42 – 7.52 (m, 2H, Ar-H), 6.92 (d, *J* = 8.6 Hz, 1H, Ar-H_7), 6.33 (d, *J* = 2.2 Hz, 1H, Ar-H_4), 2.82 (d, *J* = 5.1 Hz, 3H, NCH_3); ^{13}C NMR (100 MHz, DMSO-d_6) δ (ppm): 170.2, 163.9, 140.78, 140.76, 132.0, 130.4, 129.9, 127.2, 125.5, 118.5, 111.5, 108.3, 92.9, 30.9; HRMS: calcd. for $C_{16}H_{14}N_3O_3$ [M+H$^+$]: calcd. 296.1030, found 296.1023; Elemental analysis: calcd. C: 65.08, H: 4.44, N: 14.24; found C: 65.40, H: 4.27, N: 14.09. IR (cm^{-1}): 3107 (m), 1644 (s), 1585 (s).

(*Z*)-3-[Dimethylamino(phenyl)methylidene]-1,3-dihydro-2*H*-indol-2-one (7aa)
The crude product was purified using column chromatography (silica gel, EtOAc/*n*-hexane 2:1). Yellow solid (220 mg, 83%); mp 239 – 240 °C; ^{1}H NMR (400 MHz, DMSO-d_6) δ: 9.99 (bs, 1H, NH), 7.58 – 7.64 (m, 1H, Ar-H), 7.54 (t, *J* = 7.4 Hz, 2H, Ar-H_m), 7.44 (d, *J* = 7.6 Hz, 2H, Ar-H_o), 6.66 – 6.72 (m, 2H, Ar-H), 6.29 – 6.37 (m, 1H, Ar-H), 5.35 (d, *J* = 7.8 Hz, 1H, Ar-H_4), 3.11 (bs, 6H, 2×CH_3); ^{13}C NMR (100 MHz, DMSO-d_6, 60 °C) δ: 165.3, 160.9, 136.9,

135.5, 130.4, 129.9, 128.9, 127.3, 121.3, 118.5, 117.8, 107.7, 97.2, 43.5; HRMS: calcd. for $C_{17}H_{17}N_2O$ [M+H$^+$]: calcd. 265.1341, found 265.1341; Elemental analysis: calcd. C: 77.25, H: 6.10, N: 10.60; found C: 76.65, H: 6.07, N: 10.43. IR (cm^{-1}): 3123 (m), 1644 (s), 1540 (s).

(*Z*)-3-[Dimethylamino(4-methoxyphenyl)methylidene]-1,3-dihydro-2*H*-indol-2-one (7ab)
The crude product was purified using using preparative flash liquid chromatography (silica gel, mobile phase DCM/CH_3OH, gradient 0–5% CH_3OH during 20 min). Yellow solid (140 mg, 48%); mp 272 – 273 °C; ^{1}H NMR (400 MHz, DMSO-d_6) δ: 9.76 (bs, 1H, NH), 7.37 (AA'XX', *J* = 8.6 Hz, 2H, Ar-H), 7.07 (AA'XX', *J* = 8.5 Hz, 2H, Ar-H), 6.66 – 6.74 (m, 2H, Ar-H), 6.73 – 6.83 (m, 2H, Ar-H), 6.39 (m, 1H, Ar-H), 5.56 (d, *J* = 7.0 Hz, 1H, Ar-H), 3.86 (s, 3H, OCH_3), 3.11 (bs, 6H, 2×NCH_3); ^{13}C NMR (100 MHz, DMSO-d_6) δ 165.4, 161.1, 136.7, 131.9, 127.7, 127.4, 121.0, 118.5, 117.7, 114.4, 107.6, 96.8, 55.2, 43.4 (bs); HRMS: calcd. for $C_{18}H_{19}N_2O_2$ [M+H$^+$]: calcd. 295.1447, found 295.1448; Elemental analysis: calcd. C: 73.45, H: 6.16, N: 9.52; found C: 73.36, H: 6.22, N: 9.46. IR (cm^{-1}): 3007 (m), 1643 (s), 1606 (s), 1538 (s).

(*Z*)-3-[4-Chlorophenyl(dimethylamino)methylidene]-1,3-dihydro-2*H*-indol-2-one (7ac)
The crude product was purified using using preparative flash liquid chromatography (silica gel, mobile phase DCM/CH_3OH, gradient 0–5% CH_3OH during 20 min).Yellow solid (179 mg, 60%); mp 239.5 – 241 °C; ^{1}H NMR (400 MHz, DMSO-d_6) δ: 9.84 (bs, 1H, NH), 7.59 (AA'XX', *J* = 7.9 Hz, 2H, Ar-H), 7.47 (AA'XX', *J* = 8.1 Hz, 2H, Ar-H), 6.68 – 6.75 (m, 2H, Ar-H), 6.41 (m, 1H, Ar-H), 5.52 (d, *J* = 7.6 Hz, 1H, Ar-H), 3.11 (bs, 6H, 2×NCH_3); ^{13}C NMR (100 MHz, DMSO-d_6, 60 °C) δ 165.3, 159.4, 137.0, 135.3, 134.1, 132.0, 129.1, 127.0, 121.6, 118.7, 117.8, 107.8, 97.3, 43.6 (bs); HRMS: calcd. for $C_{17}H_{16}ClN_2O$ [M+H$^+$]: calcd. 299.0951, found 299.0954; Elemental analysis: calcd. C: 68.34, H: 5.06, N: 9.38, Cl: 11.87; found C: 68.67, H: 5.09, N: 9.30, Cl: 11.69. IR (cm^{-1}): 3085 (m), 1651 (s), 1544 (s).

(*Z*)-3-[Dimethylamino(phenyl)methylidene]-5-methyl-1,3-dihydro-2*H*-indol-2-one (7ba)
The crude product was purified using column chromatography (neutral alumina, DCM/*i*-PrOH/ conc. NH_3 10:1:0.1). Yellow solid (740 mg, 53%); mp 214 – 215 °C; ^{1}H NMR (400 MHz, DMSO-d_6) δ: 9.69 (bs, 1H, NH), 7.40 – 7.62 (m, 5H, Ar-H), 6.50 – 6.60 (m, 2H, Ar-H), 5.20 (s, 1H, Ar-H_4), 3.11 (s, 6H, 2×NCH_3); ^{13}C NMR (100 MHz, DMSO-d_6, 60 °C) δ: 165.6, 160.7, 135.6, 134.8, 130.3, 130.0, 128.9, 127.5, 126.6, 121.9, 118.8, 107.3, 97.4, 43.5, 20.8; HRMS: calcd. for $C_{18}H_{19}N_2O$ [M+H$^+$]: calcd. 279.1492, found 279.1497; Elemental analysis: calcd. C: 77.67, H: 6.52, N: 10.06; found C: 77.79, H: 6.56, N: 9.99. IR (cm^{-1}): 3388 (m), 1639 (s)..

(*Z*)-6-Chloro-3-[dimethylamino(phenyl)methylidene]-1,3-dihydro-2*H*-indol-2-one (7ca)
The crude product was purified using column chromatography (neutral alumina, DCM/*i*-PrOH/conc. NH_3 10:1:0.1). Yellow solid (280 mg, 19%); mp 269 – 270.5 °C; ^{1}H NMR (400 MHz, DMSO-d_6) δ: 9.95 (bs, 1H, NH), 7.61 (t, *J* = 7.2 Hz, 1H, Ar-H_p), 7.54 (t, *J* = 7.3 Hz, 2H, Ar-H_m), 7.44 (d, *J* = 7.3 Hz, 2H, Ar-H_o), 6.71 (d, *J* = 1.5 Hz, 1H, Ar-H_7), 6.36 (d, *J* = 7.0 Hz, 1H, Ar-H_5), 5.33 (d, *J* = 6.4 Hz, 1H, Ar-H_4), 3.13 (s, 6H, 2×NCH_3); ^{13}C NMR (100 MHz, DMSO-d_6, 60 °C) δ: 165.1, 161.7, 137.8, 135.1, 130.6, 129.9, 129.0, 126.4, 125.4, 118.5, 118.1,

107.5, 96.1, 43.7; HRMS: calcd. for $C_{17}H_{16}ClN_2O$ [M+H$^+$]: calcd. 299.0951, found 299.0952; Elemental analysis: calcd. C: 68.34, H: 5.06, N: 9.38, Cl: 11.87; found C: 68.63, H: 5.17, N: 9.20, Cl: 11.71. IR (cm^{-1}): 3068 (m), 1652 (s), 1606 (m), 1542 (s).

(*Z*)-3-[1-Aminoethylidene]-1,3-dihydro-2*H*-indol-2-one (10a)

The crude product was purified using column chromatography (neutral alumina, EtOAc/CH_3OH/TEA 6:1:0.05). White solid (310 mg, 89%); mp 261 – 262.5 °C (Ref. [10] gives 225 °C); ^{1}H NMR (500 MHz, DMSO-*d*$_6$) δ 10.18 (bs, 1H, NH), 9.31 (vbs, 1H, ½NH_2), 7.95 (vbs, 1H, ½NH_2), 7.22 (d, *J* = 6.7 Hz, 1H, Ar-H), 6.90 (t, *J* = 7.3 Hz, 1H, Ar-H), 6.86 (d, *J* = 7.3 Hz, 1H, Ar-H), 6.82 (t, *J* = 7.5 Hz, 1H, Ar-H), 2.37 (s, 3H, CH_3) corresponds to Ref. [11]. HRMS: calcd. for $C_{10}H_{11}N_2O$ [M+H$^+$]: calcd. 175.0866, found 175.0865; Elemental analysis: calcd. C: 68.95, H: 5.79, N: 16.08; found C: 68.86, H: 5.76, N: 16.14. IR (cm^{-1}): 3359 (m), 1633 (s), 1538 (s).

(*Z*)-3-[(1-Methylamino)ethylidene]-1,3-dihydro-2*H*-indol-2-one (10b)

The crude product was purified using preparative flash liquid chromatography (silica gel, mobile phase DCM/CH_3OH + 5% TEA, gradient 0–5% CH_3OH during 15 min).White solid (320 mg, 85%); mp 281 - 282 °C (Ref. [12] gives 280 °C); ^{1}H NMR (400 MHz, DMSO-*d*$_6$) δ 10.33 (bs, 1H, NH), 10.05 (bs, 1H, NH), 7.31 (d, *J* = 6.8 Hz, 1H, Ar-H), 6.81 - 6.93 (m, 3H, Ar-H), 3.06 (d, *J* = 5.1 Hz, 3H, NCH_3), 2.42 (s, 3H, CH_3) corresponds to Ref. [12]; ^{13}C NMR (100 MHz, DMSO-*d*$_6$, 60 °C) δ: 169.4, 161.8, 135.0, 125.2, 121.0, 119.5, 117.4, 108.3, 93.2, 28.9, 15.1. HRMS: calcd. for $C_{11}H_{13}N_2O$ [M+H$^+$]: calcd. 189.1022, found 189.1020; Elemental analysis: calcd. C: 70.19, H: 6.43, N: 14.88; found C: 70.07, H: 6.42, N: 15.00. IR (cm^{-1}): 3131 (m), 1630 (s), 1590 (s).

(*Z*)-3-[(1-Phenylamino)ethylidene]-1,3-dihydro-2*H*-indol-2-one (10c)

The crude product was purified using preparative flash liquid chromatography (silica gel, mobile phase DCM/CH_3OH + 5% TEA, gradient 0–5% CH_3OH during 20 min). Yellow solid (312 mg, 62%); mp 222 - 223 °C (Ref. [13] gives 215-216 °C); ^{1}H NMR (500 MHz, $CDCl_3$) δ 12.02 (bs, 1H, NH), 8.74 (bs, 1H, NH), 7.41 (t, *J* = 7.8 Hz, 2H, Ar-H_m), 7.37 (d, 1H, Ar-H), 7.26 (t, *J* = 7.5 Hz, 1H, Ar-H_p), 7.20 (d, *J* = 7.6 Hz, 2H, Ar-H_o), 6.97 – 7.10 (m, 3H, Ar-H), 2.51 (s, 3H, CH_3) corresponds to Ref. [13]; ^{13}C NMR (100 MHz, DMSO-*d*$_6$, 60 °C) δ: 169.6, 156.9, 137.7, 136.0, 129.1, 125.3, 124.5, 124.2, 122.5, 120.1, 118.7, 108.8, 96.8, 16.9. HRMS: calcd. for $C_{16}H_{15}N_2O$ [M+H$^+$]: calcd. 251.1179, found 251.1177; Elemental analysis: calcd. C: 76.78, H: 5.64, N: 11.19; found C: 76.56, H: 5.59, N: 11.35. IR (cm^{-1}): 3155 (m), 1651 (s), 1613 (s).

Eschenmoser coupling of diethyl (2-oxoindolin-3-yl)phosphate (1g) with thiobenzamide (2a)

A closeable vial was charged with thiobenzamide (**2a**, 343 mg, 0.25 mmol) and DMF (2.5 mL). Diethyl (2-oxoindolin-3-yl)phosphate (**1g**, 0.25–1 mmol, 1–4 equiv) and in one case also tetrabutylamonium bromide (161 mg, 20 mol %) were added. The closed vial was stirred at the given temperature and time below. Samples of the reaction mixture were analyzed by HPLC (Phenomenex Synergi Polar-RP 250 × 4.6 mm column; isocratic mode, mobile phase acetonitrile/water 1:1, flow rate 1 mL·min^{-1}) equipped with a UV–vis detector (245 nm). Calibration series for starting compounds and products were determined. Results are summarized in the following Table 4:

Table S4 – HPLC monitoring of reaction **1g** with **2a**.

Run	Temperature [°C]	Phosphate [equiv.]	Time [h]	Conversion (%) Thiobenzamide	Phosphate	**5aa** (%)
1	25	1	150	5	4	traces
2	80	1	6	25	17	2
			32	91	>99	21
3	80	2	6	29	22	5
			32	98	>99	38
4	80	3	6	36	18	9
			32	>99	>99	37
5	80	4	6	41	10	15
			32	>99	>99	31
6	80	1*	19	27	94	17

*addition of 20 mol % TBAB

Eschenmoser coupling of 3-hydroxy-*N*-methyloxindole with thiobenzamide (2a) giving (*Z*)-3-[amino(phenyl)methylidene]-1-methyl-1,3-dihydro-2*H*-indol-2-one (11)

3-Hydroxy-*N*-methyloxindole (163 mg, 1 mmol) was dissolved in dry DMF (10 mL) under argon atmosphere. The solution was cooled to −20 °C and triflic anhydride (160 µL, 1 mmol) was added dropwise through a gas-tight syringe during 10 min. The reaction mixture was stirred for 40 min, during which time the temperature reached 0 °C. Then, thiobenzamide (**2a**, 137 mg, 1 mmol) was added and reaction mixture was further stirred overnight at room temperature. The reaction mixture was evaporated and the residue was dissolved in DCM (20 mL) and evaporated with silica gel (5 g). Preparative flash chromatography (silica gel cartridge; mobile phase DCM/EtOAc + 3 % TEA, gradient of EtOAc 0–20% during 20 min) gave 220 mg (87%) of yellow crystals of **11** with m.p. 174 – 176.5 °C. ^{1}H NMR (400 MHz, DMSO-*d*$_6$) δ 9.47 (bd, *J* 3.7 Hz, 1H, ½NH$_2$), 8.16 (bd, *J* 3.4 Hz, 1H, ½NH$_2$), 7.43-7.67 (m, 5H, Ar-H), 6.82-7.02 (m, 2H, Ar-H), 6.57-6.65 (m, 1H, Ar-H), 6.10 (d, *J* 7.8 Hz, 1H, Ar-H), 3.27 (s, 3H, CH$_3$) corresponds to Ref. [1].

The Rhe reaction of 3-hydroxyoxindole with thiobenzamide (**2a**) giving (*Z*)-3-[amino(phenyl)-methylidene]-1,3-dihydro-2*H*-indol-2-one (**5aa**) was carried out in the same manner.

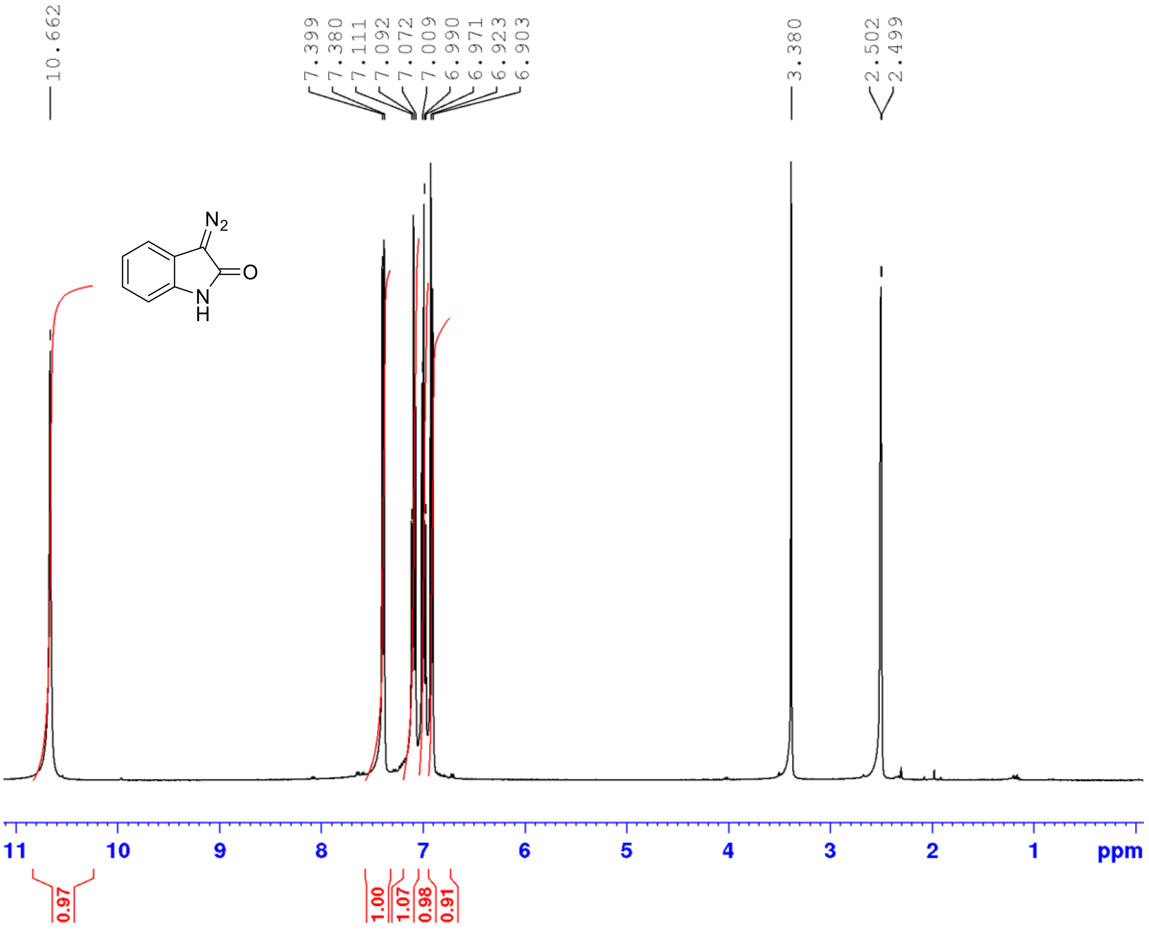

10.662
7.399
7.380
7.111
7.092
7.072
7.009
6.990
6.971
6.923
6.903
3.380
2.502
2.499
N2
O
N
H
11
10
9
8
7
6
5
4
3
2
1
ppm
0.97
1.00
1.07
0.98
0.91

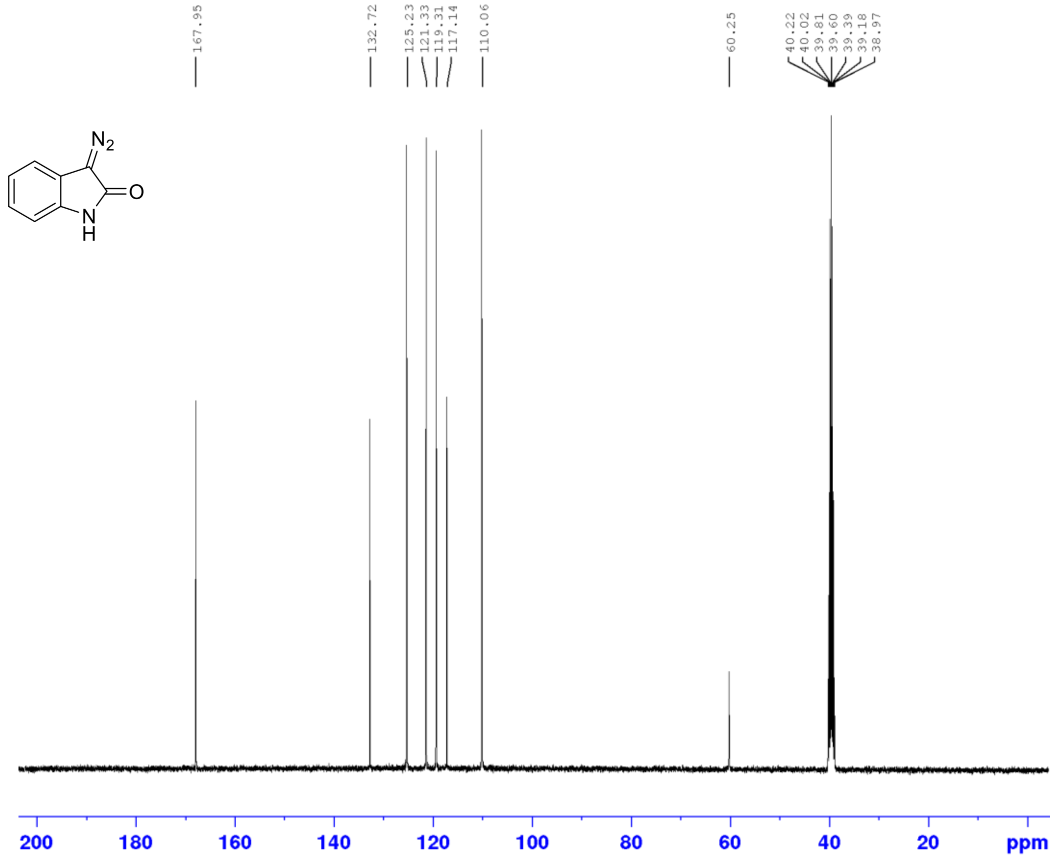

167.95
132.72
125.23
121.33
119.31
117.14
110.06
60.25
40.22
40.02
39.81
39.60
39.39
39.18
38.97
N2
O
N
H
200
180
160
140
120
100
80
60
40
20
ppm

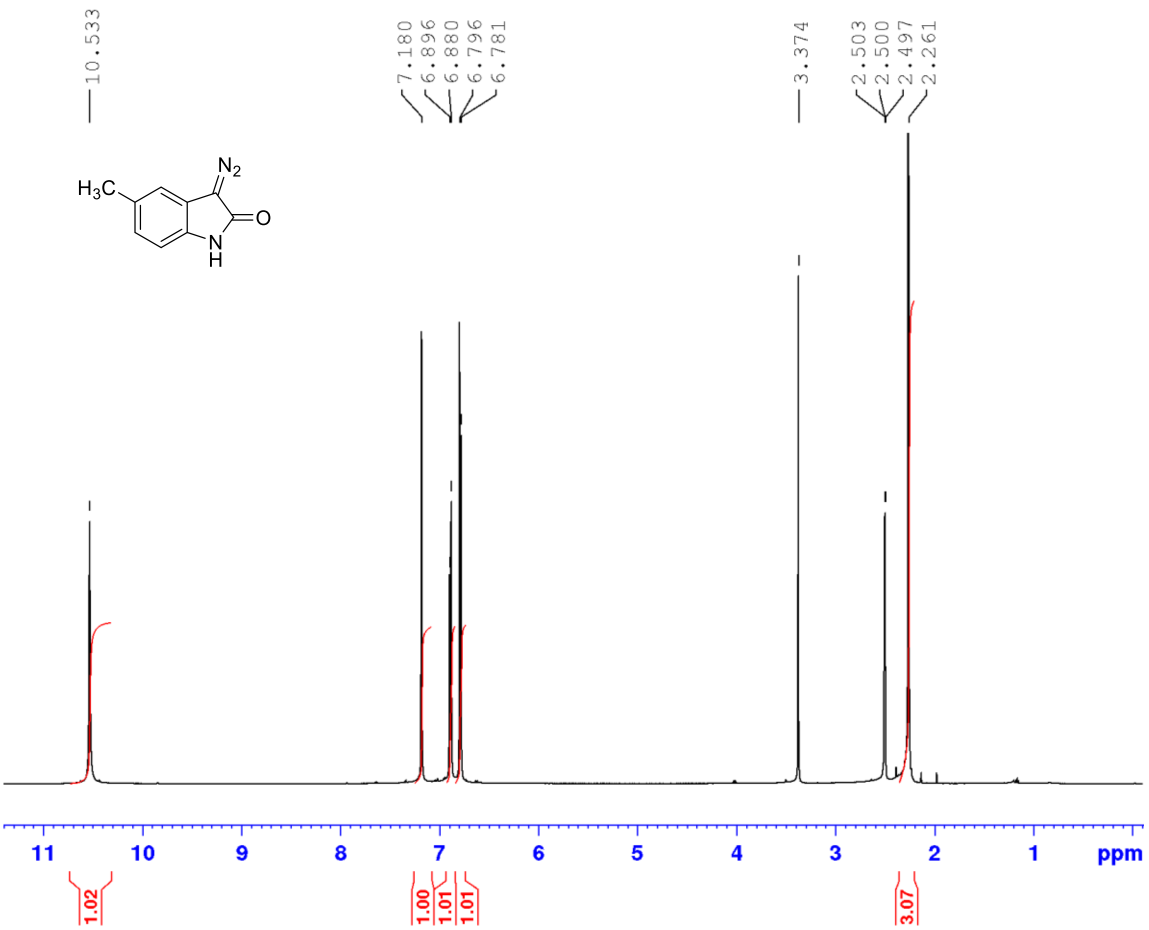
10.533
7.180
6.896
6.880
6.796
6.781
3.374
2.503
2.500
2.497
2.261
H_3C
N_2
O
N
H
11
10
9
8
7
6
5
4
3
2
1
ppm
1.02
1.00
1.01
1.01
3.07

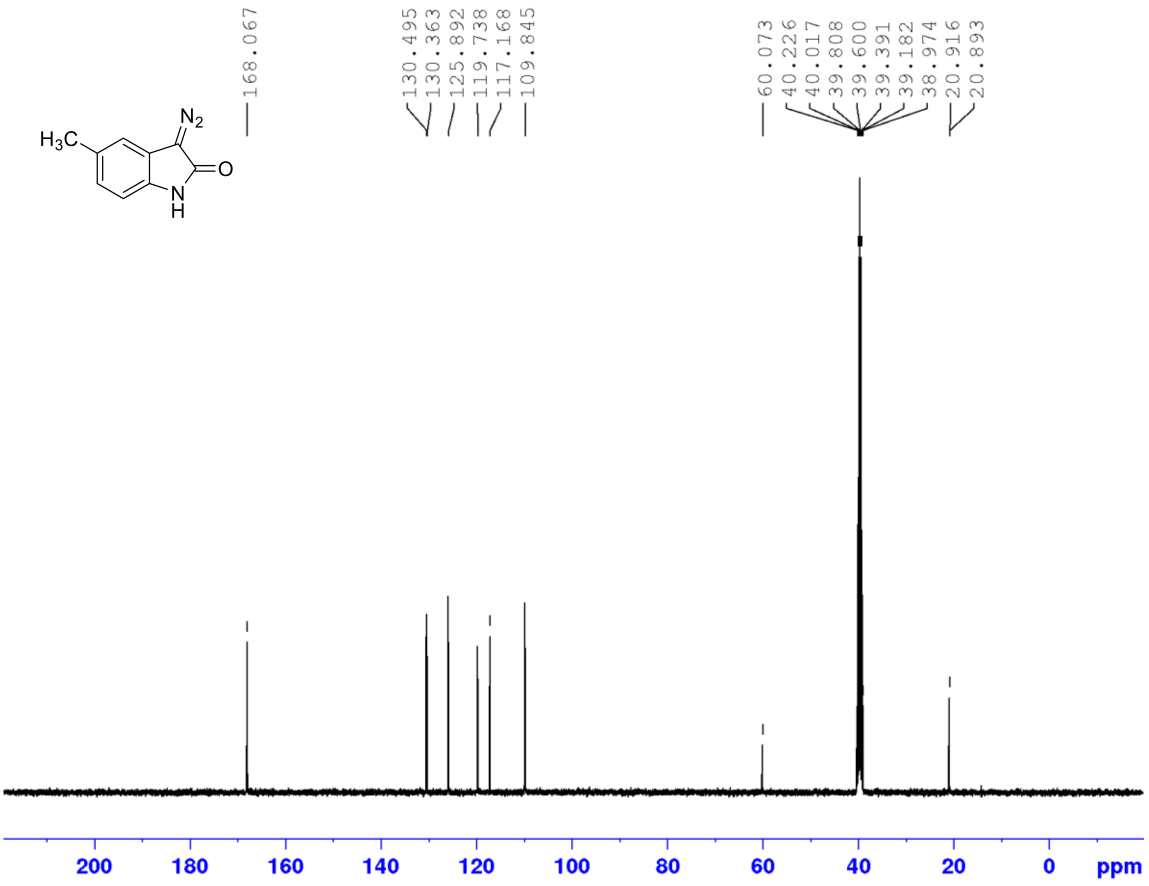
168.067
130.495
130.363
125.892
119.738
117.168
109.845
60.073
40.226
40.017
39.808
39.600
39.391
39.182
38.974
20.916
20.893
H_3C
N_2
O
N
H
200
180
160
140
120
100
80
60
40
20
0
ppm

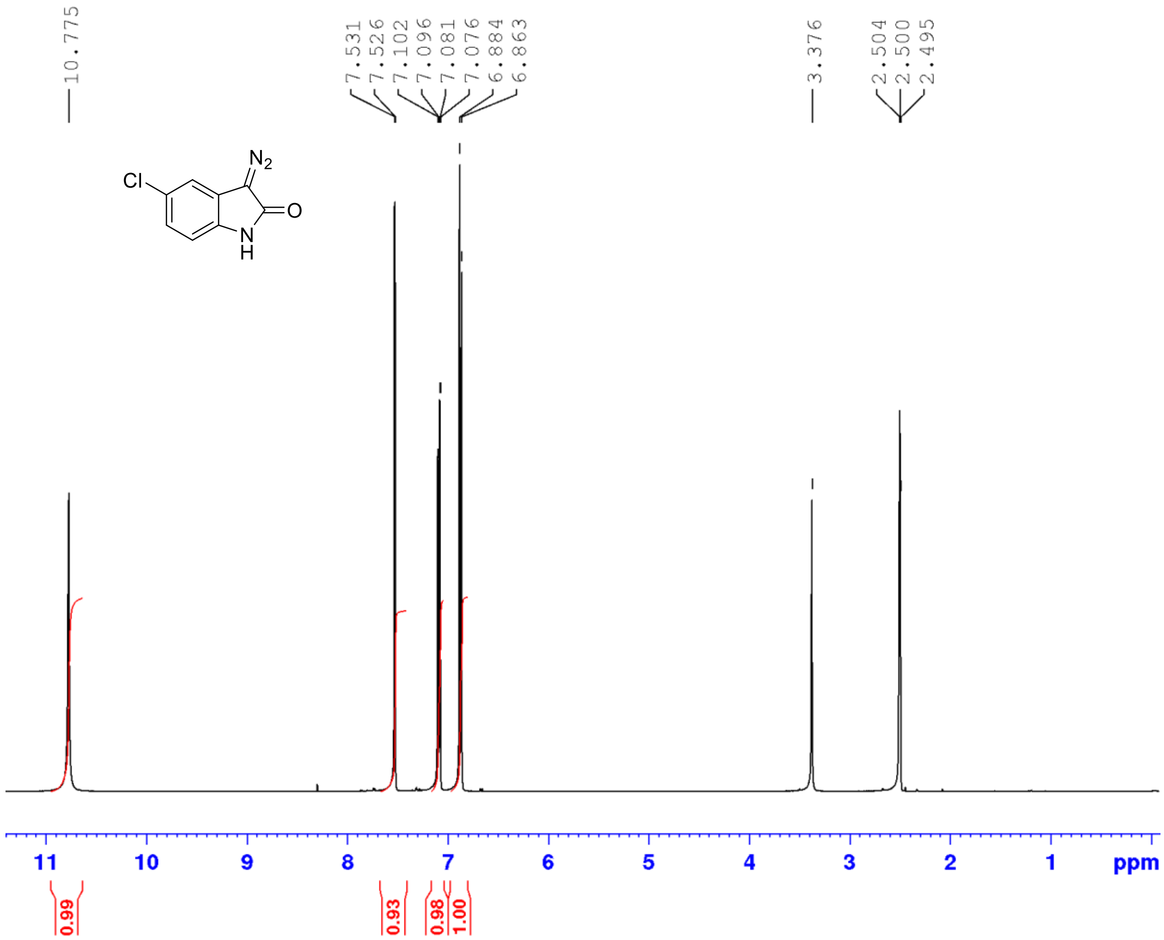
10.775
7.531
7.526
7.102
7.096
7.081
7.076
6.884
6.863
3.376
2.504
2.500
2.495
Cl
N_2
O
N
H
11
10
9
8
7
6
5
4
3
2
1
ppm
0.99
0.93
0.98
1.00

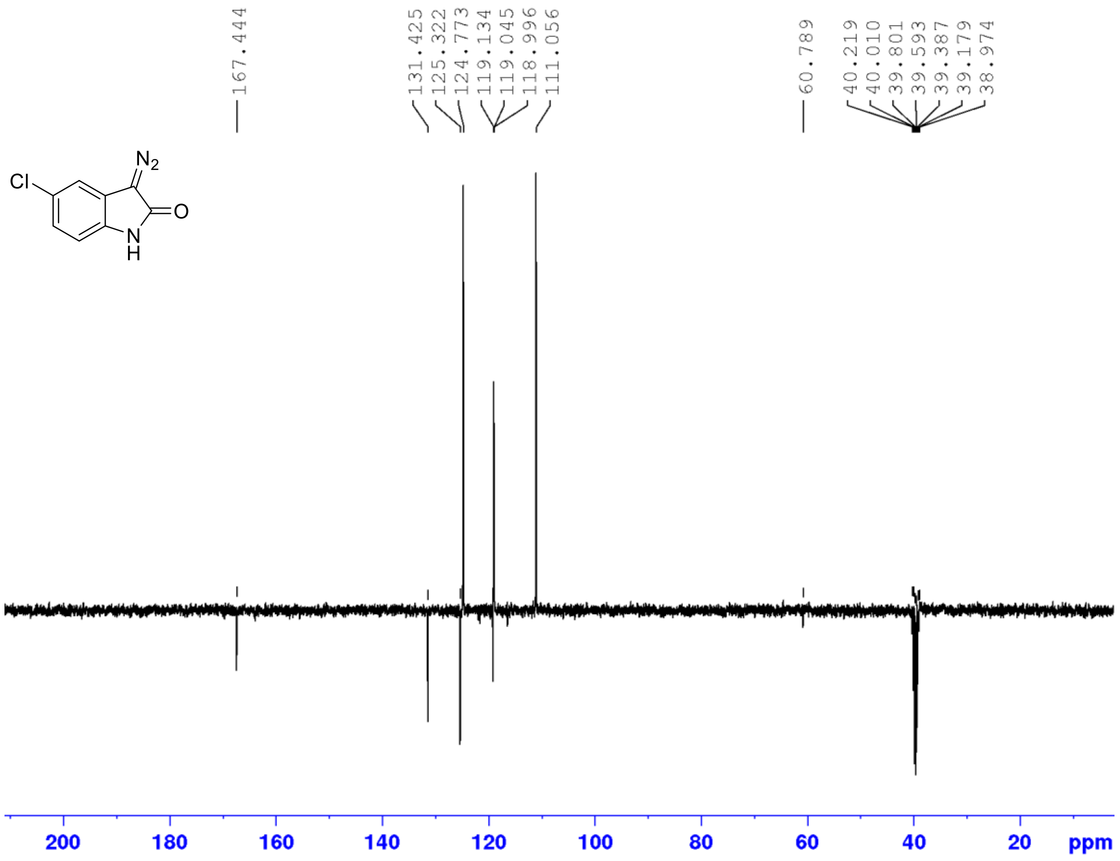
167.444
131.425
125.322
124.773
119.134
119.045
118.996
111.056
60.789
40.219
40.010
39.801
39.593
39.387
39.179
38.974
Cl
N_2
O
N
H
200
180
160
140
120
100
80
60
40
20
ppm

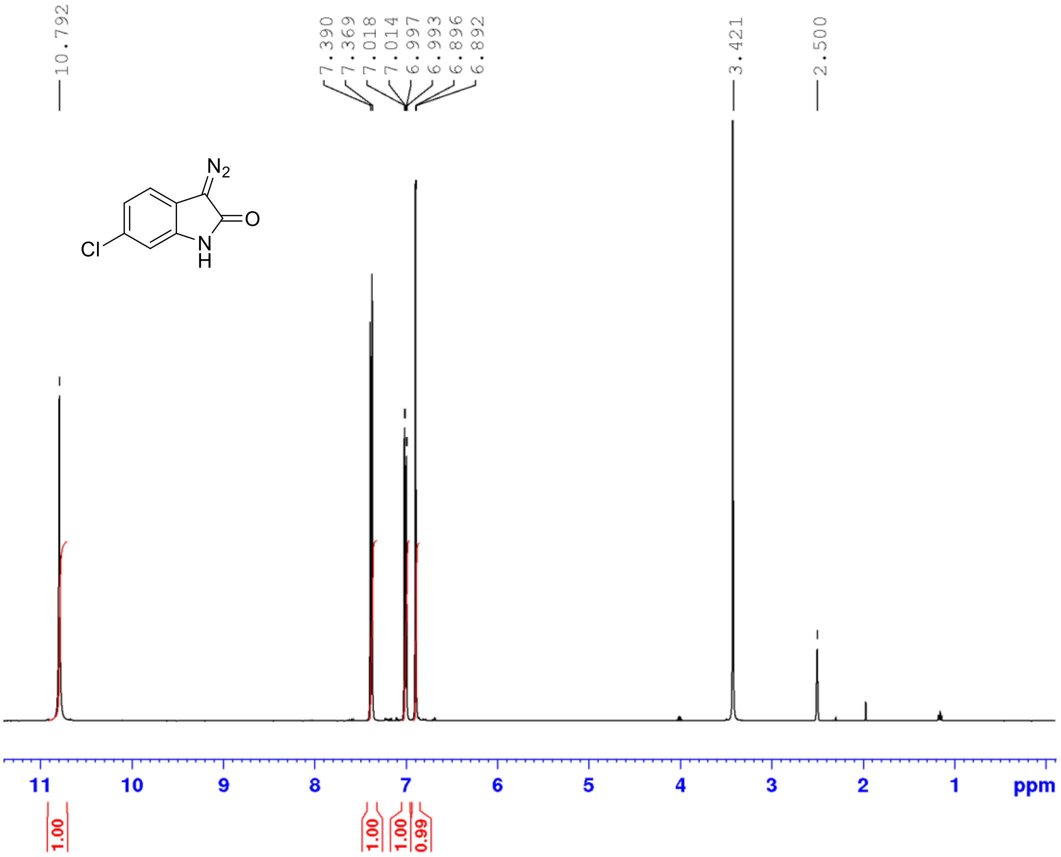
10.792
7.390
7.369
7.018
7.014
6.997
6.993
6.896
6.892
3.421
2.500
N_2
O
Cl
N
H
11 10 9 8 7 6 5 4 3 2 1 ppm
1.00
1.00
1.00
0.99

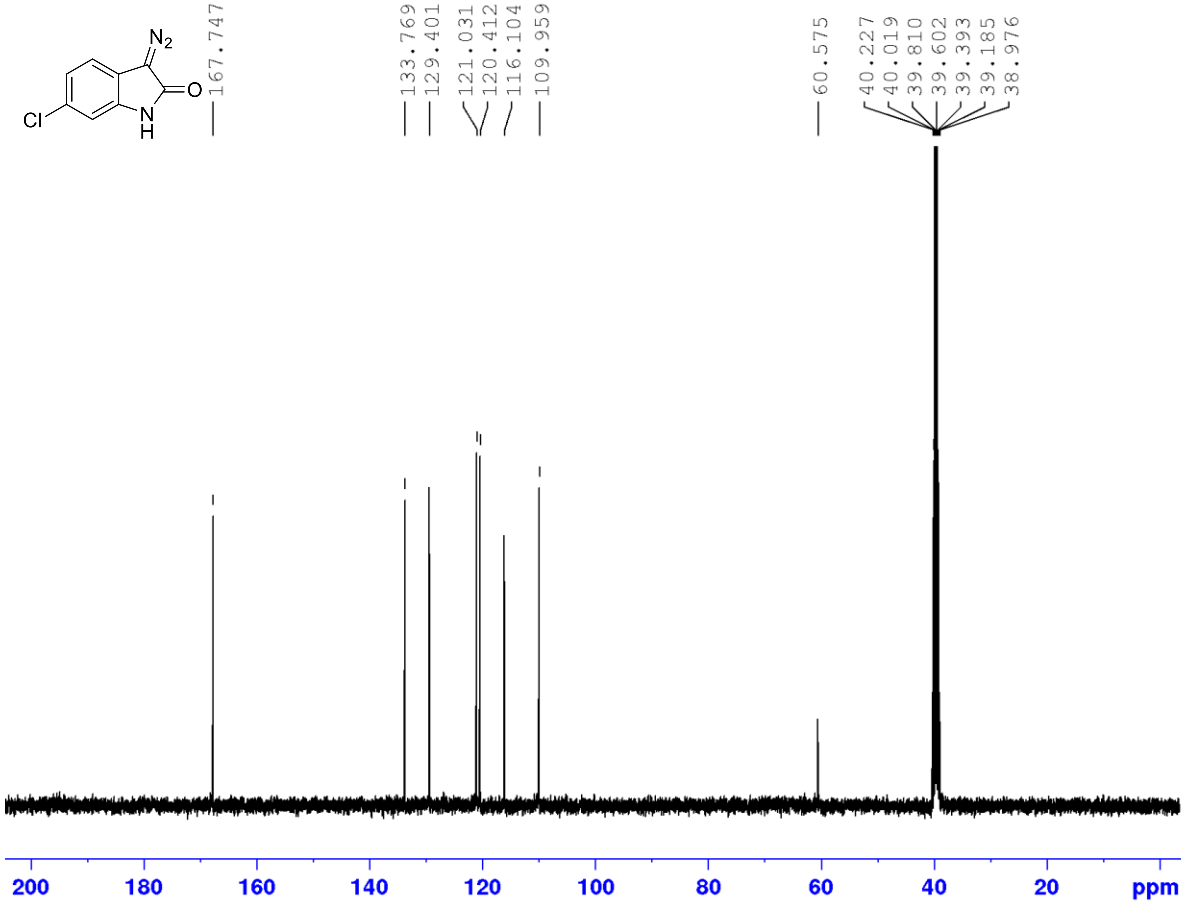
N_2
O
Cl
N
H
167.747
133.769
129.401
121.031
120.412
116.104
109.959
60.575
40.227
40.019
39.810
39.602
39.393
39.185
38.976
200 180 160 140 120 100 80 60 40 20 ppm

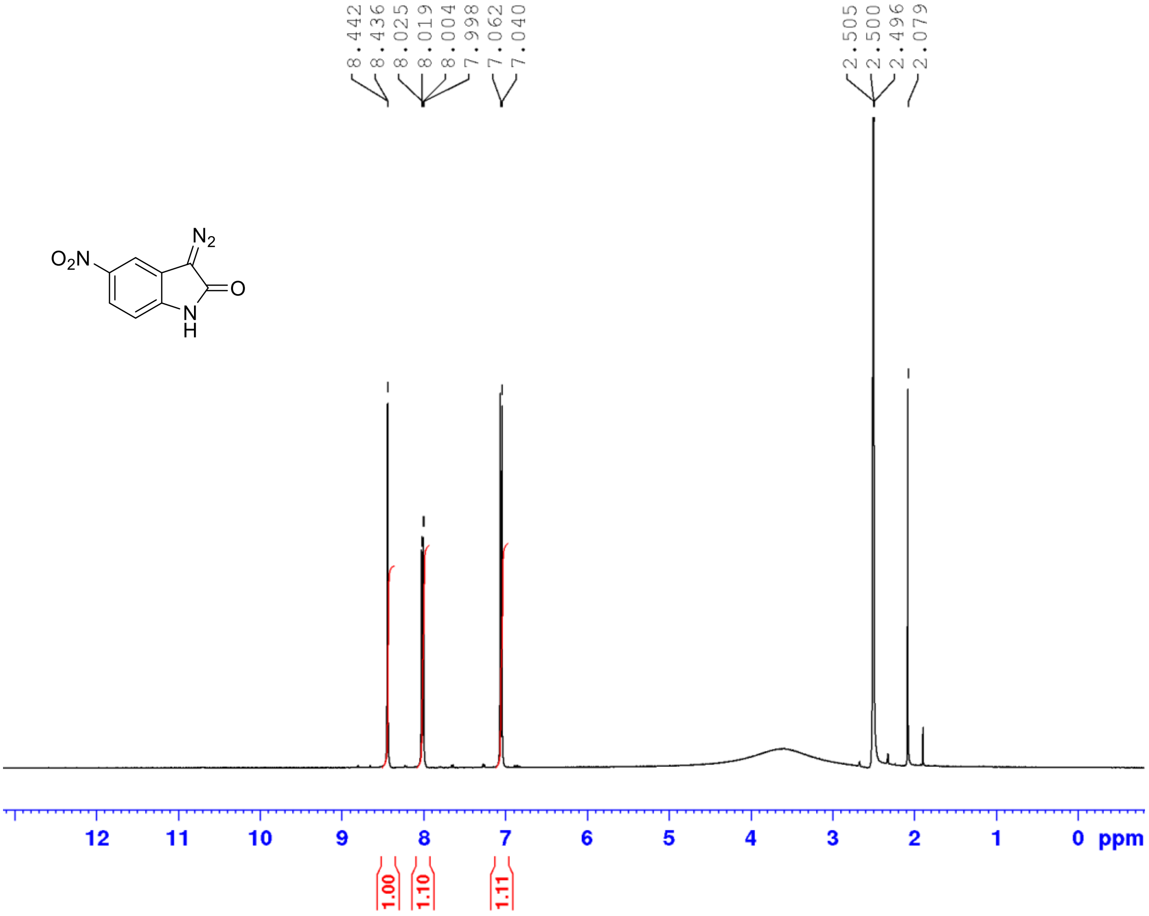

8.442
8.436
8.025
8.019
8.004
7.998
7.062
7.040
2.505
2.500
2.496
2.079
O_2N
N_2
O
N
H
12 11 10 9 8 7 6 5 4 3 2 1 0 ppm
1.00
1.10
1.11

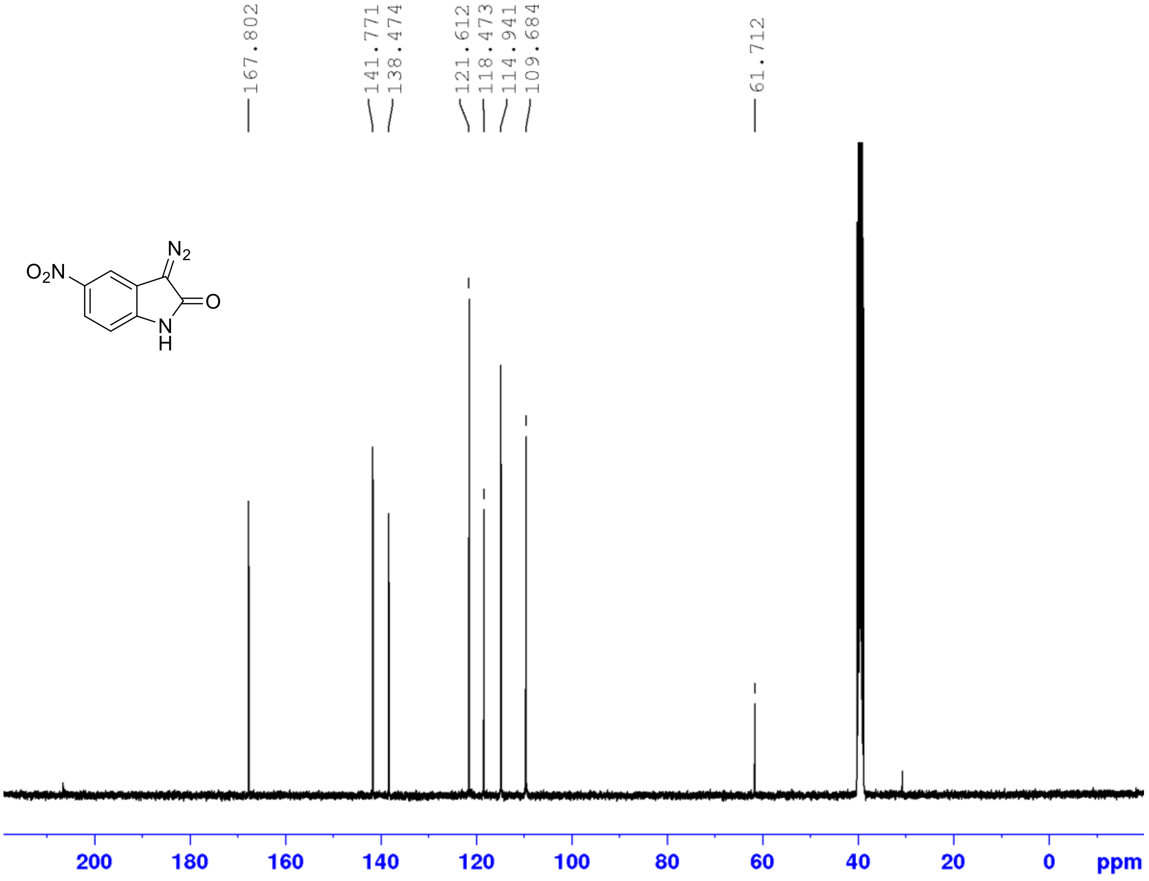

167.802
141.771
138.474
121.612
118.473
114.941
109.684
61.712
O_2N
N_2
O
N
H
200 180 160 140 120 100 80 60 40 20 0 ppm

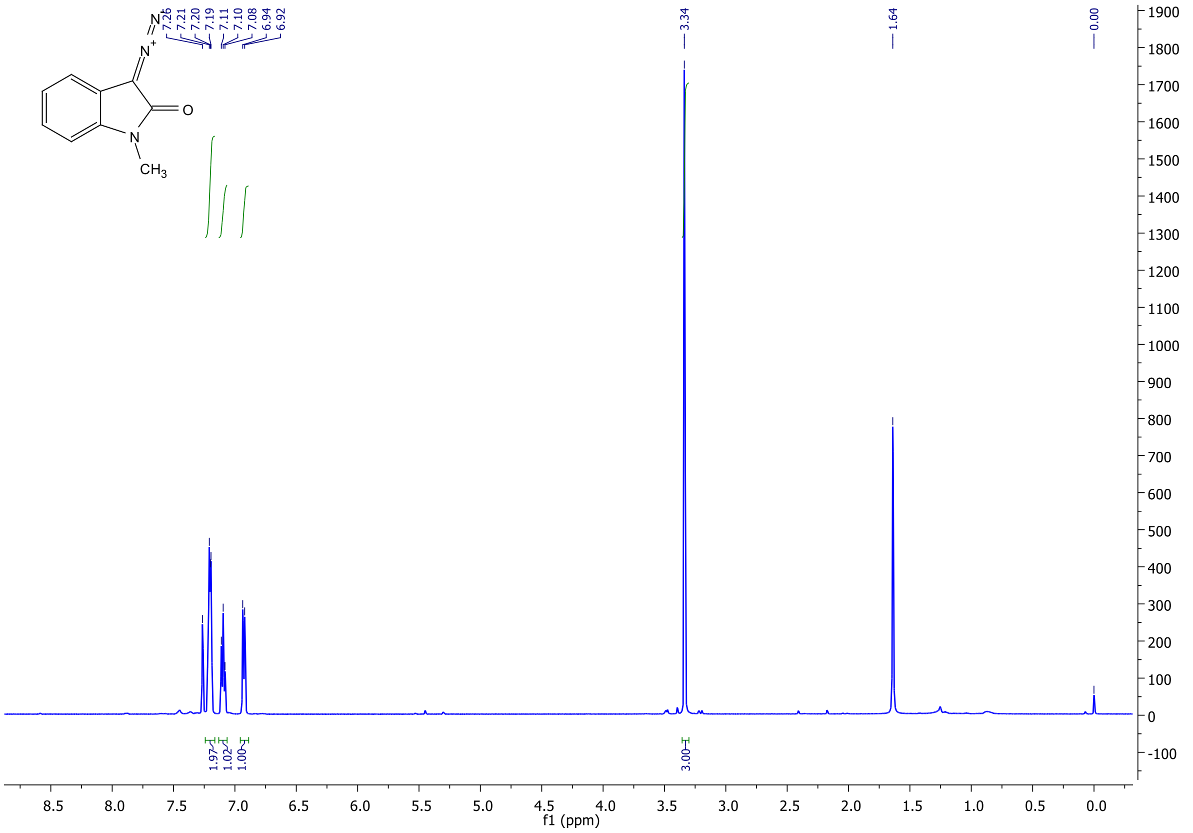

7.26
7.21
7.20
7.19
7.11
7.10
7.08
6.94
6.92
3.34
1.64
0.00
1.97
1.02
1.00
3.00
f1 (ppm)

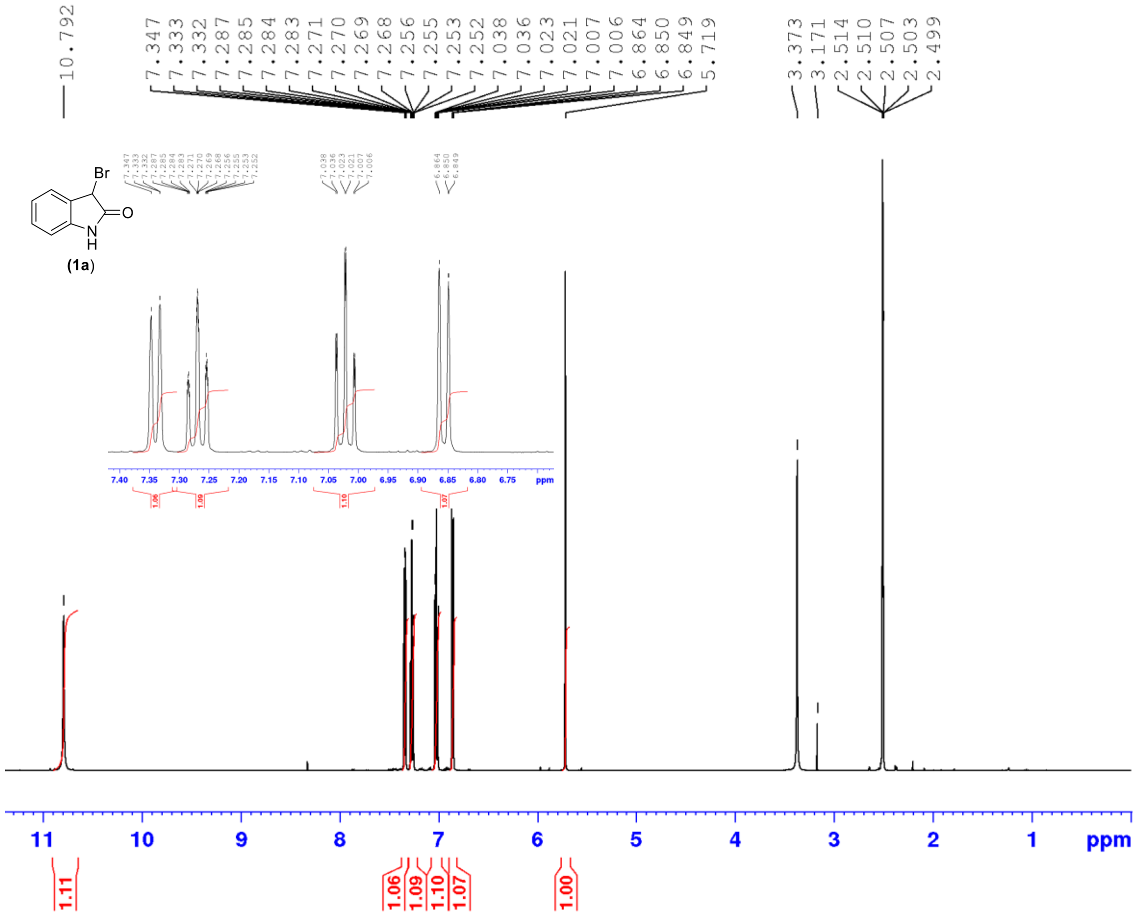

10.792
7.347
7.333
7.332
7.287
7.285
7.284
7.283
7.271
7.270
7.269
7.268
7.256
7.255
7.253
7.252
7.038
7.036
7.023
7.021
7.007
7.006
6.864
6.850
6.849
5.719
3.373
3.171
2.514
2.510
2.507
2.503
2.499
Br
O
N
H
(1a)
1.11
1.06
1.09
1.10
1.07
1.00
ppm

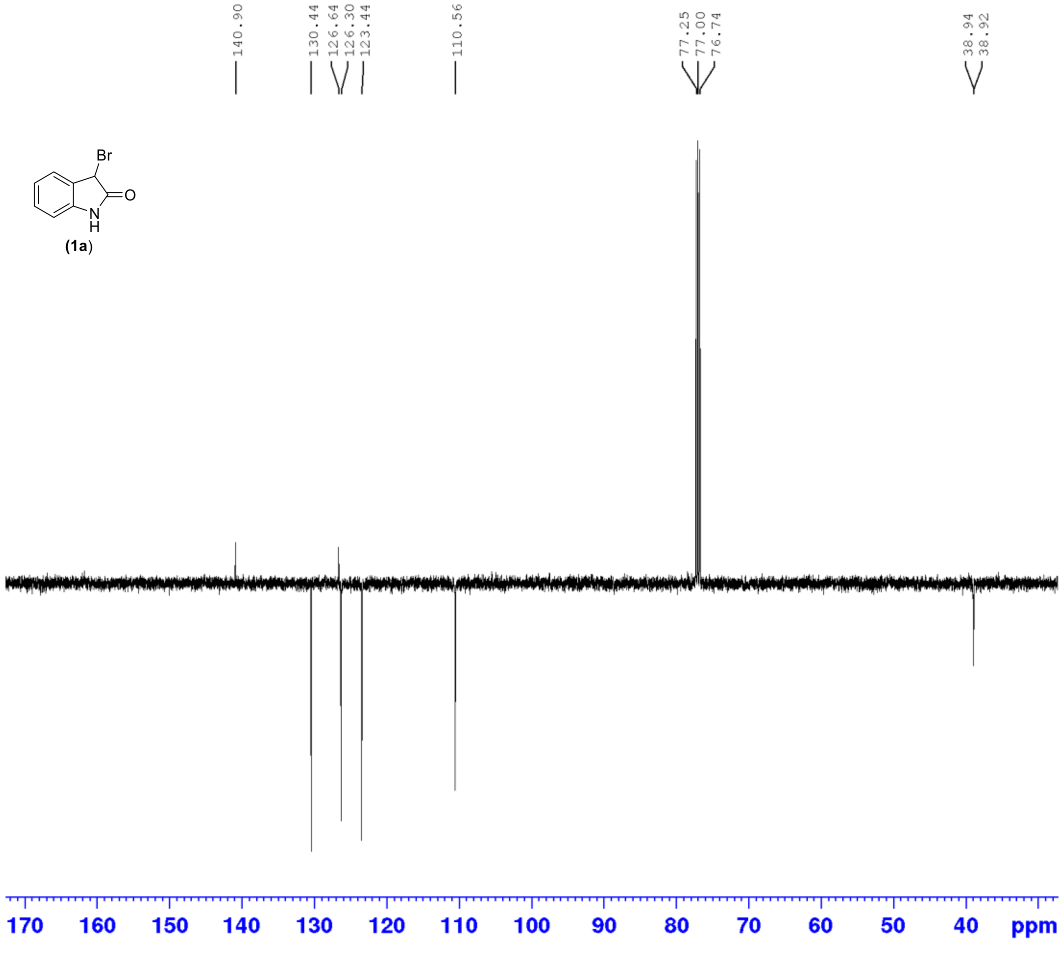

140.90
130.44
126.64
126.30
123.44
110.56
77.25
77.00
76.74
38.94
38.92
Br
O
N
H
(1a)
ppm

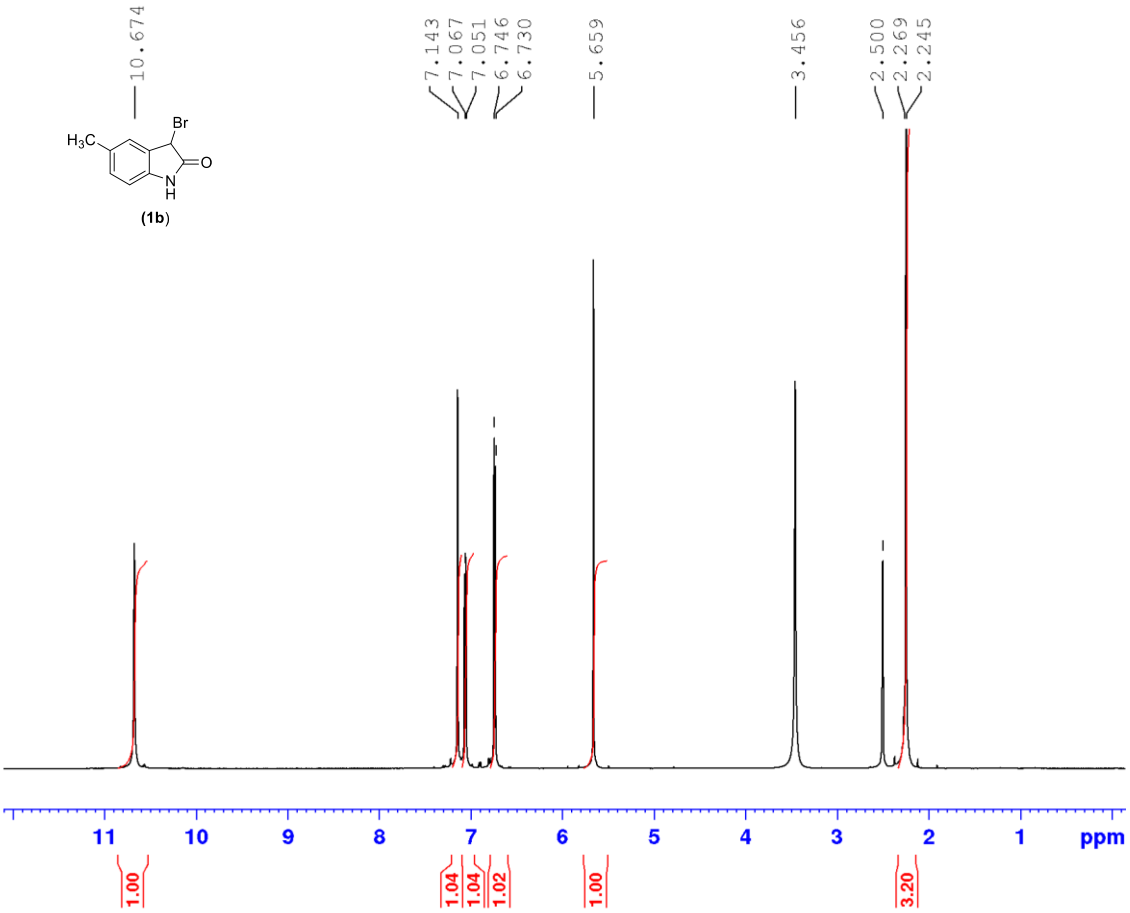
10.674
7.143
7.067
7.051
6.746
6.730
5.659
3.456
2.500
2.269
2.245
H_3C
Br
O
N
H
(1b)
11
10
9
8
7
6
5
4
3
2
1
ppm
1.00
1.04
1.04
1.02
1.00
3.20

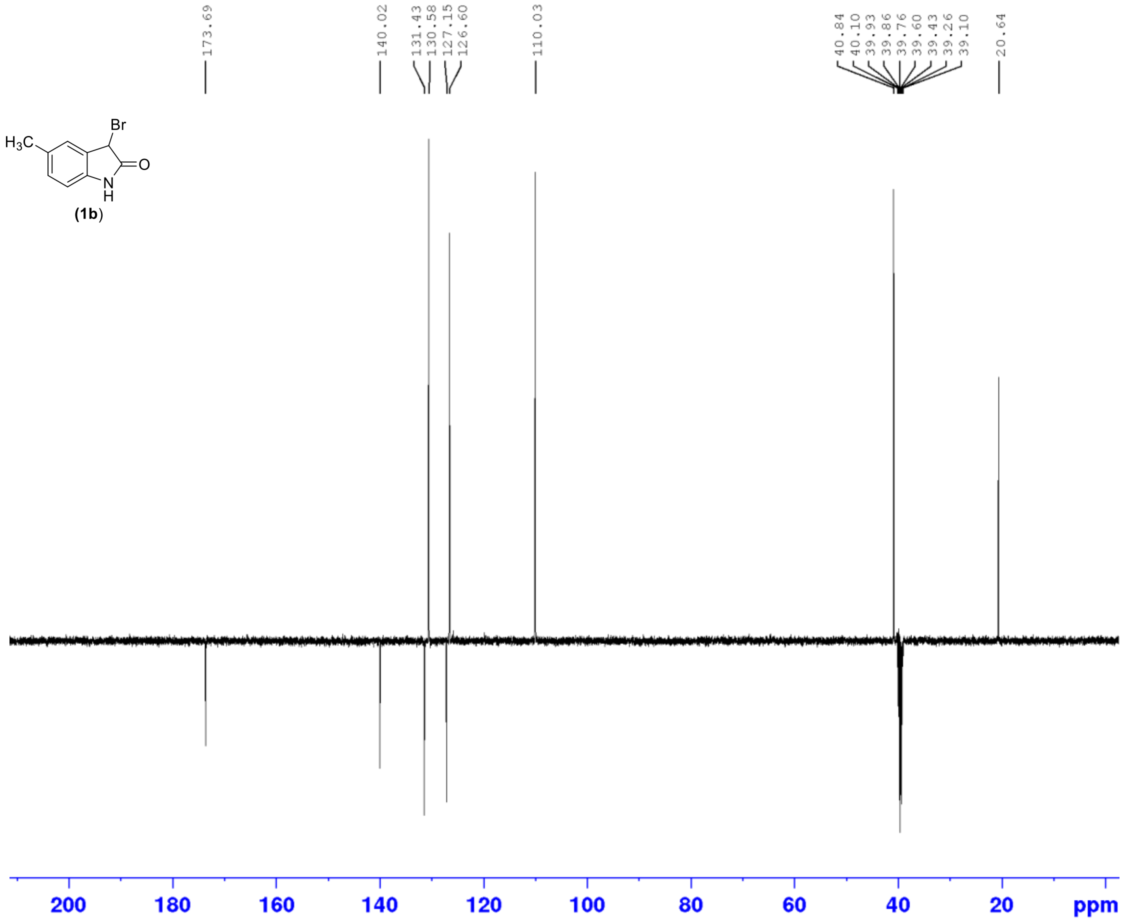
173.69
140.02
131.43
130.58
127.15
126.60
110.03
40.84
40.10
39.93
39.86
39.76
39.60
39.43
39.26
39.10
20.64
H_3C
Br
O
N
H
(1b)
200
180
160
140
120
100
80
60
40
20
ppm

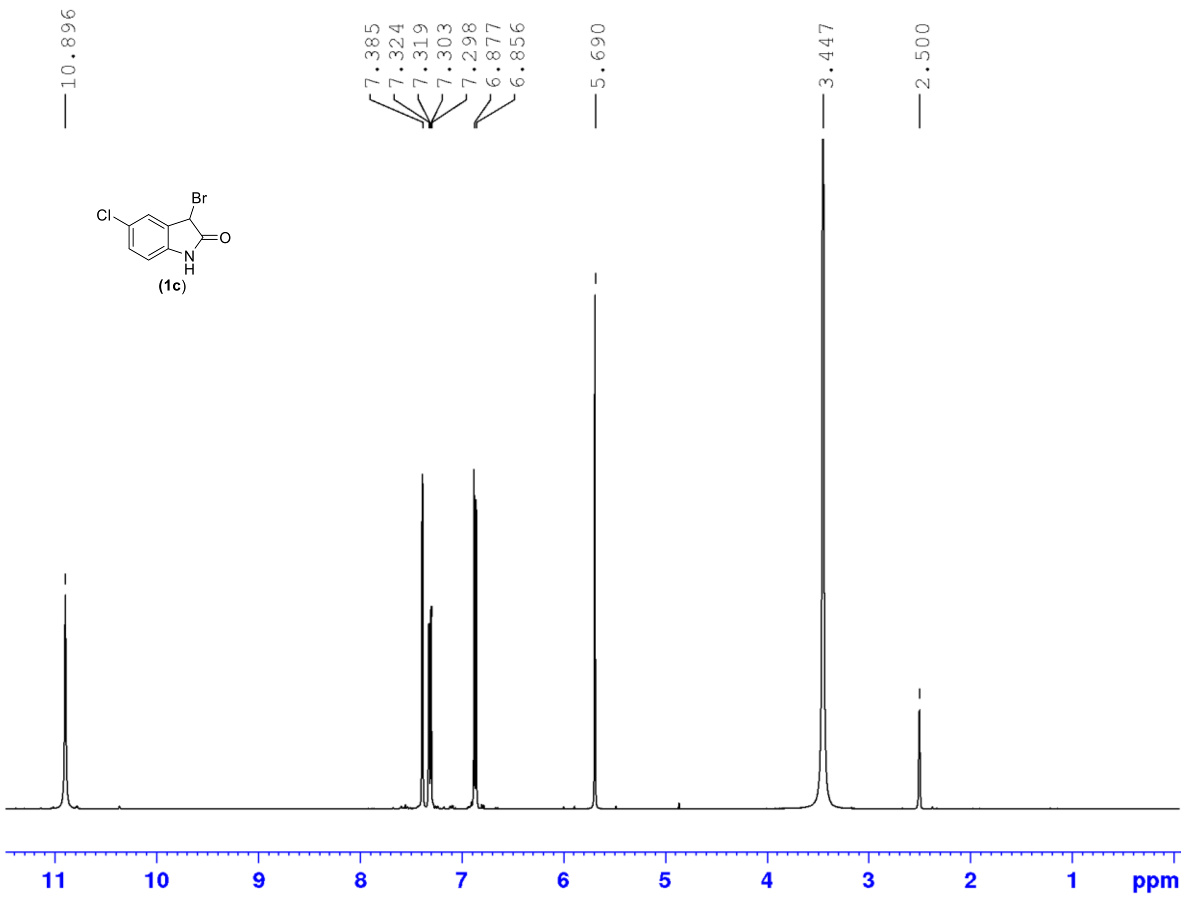
10.896
7.385
7.324
7.319
7.303
7.298
6.877
6.856
5.690
3.447
2.500
Cl
Br
O
N
H
(1c)
11
10
9
8
7
6
5
4
3
2
1
ppm

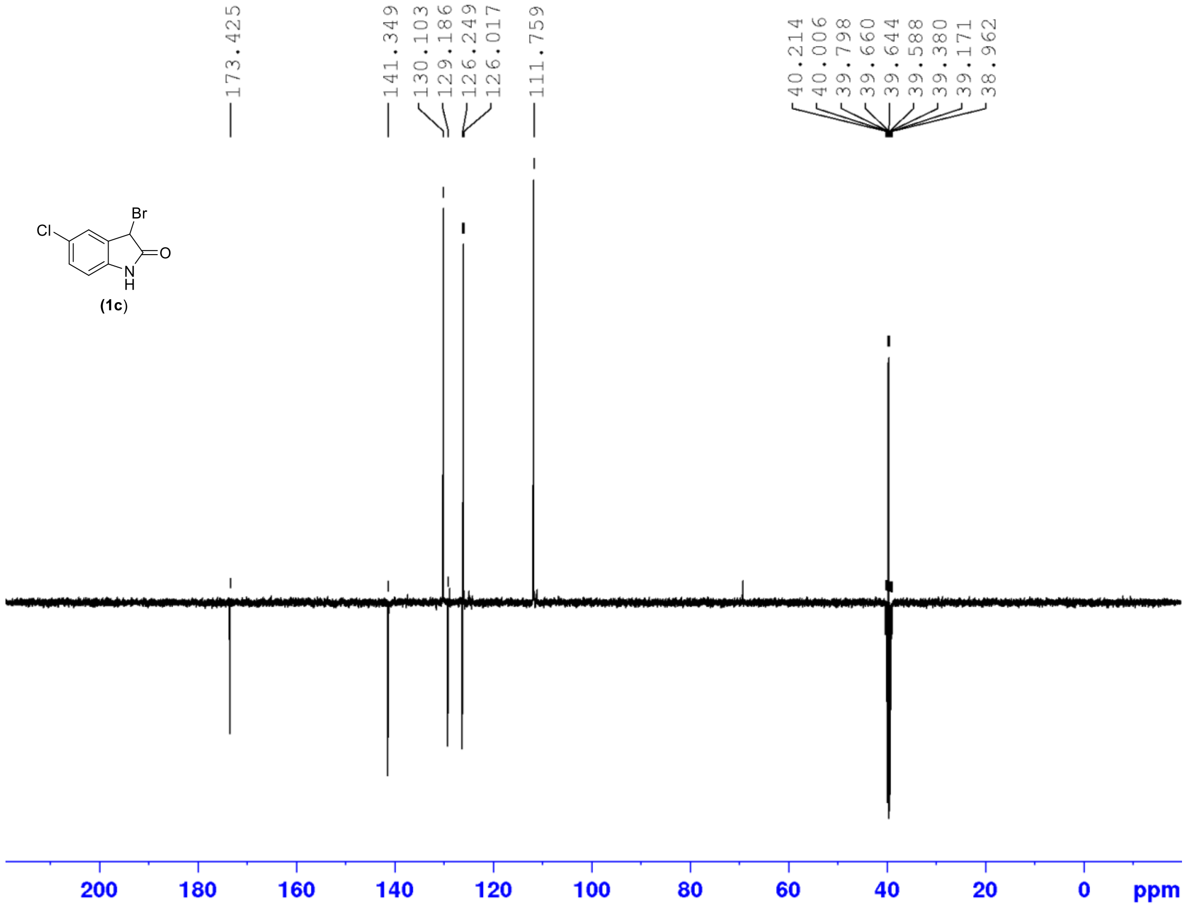
173.425
141.349
130.103
129.186
126.249
126.017
111.759
40.214
40.006
39.798
39.660
39.644
39.588
39.380
39.171
38.962
Cl
Br
O
N
H
(1c)
200
180
160
140
120
100
80
60
40
20
0
ppm

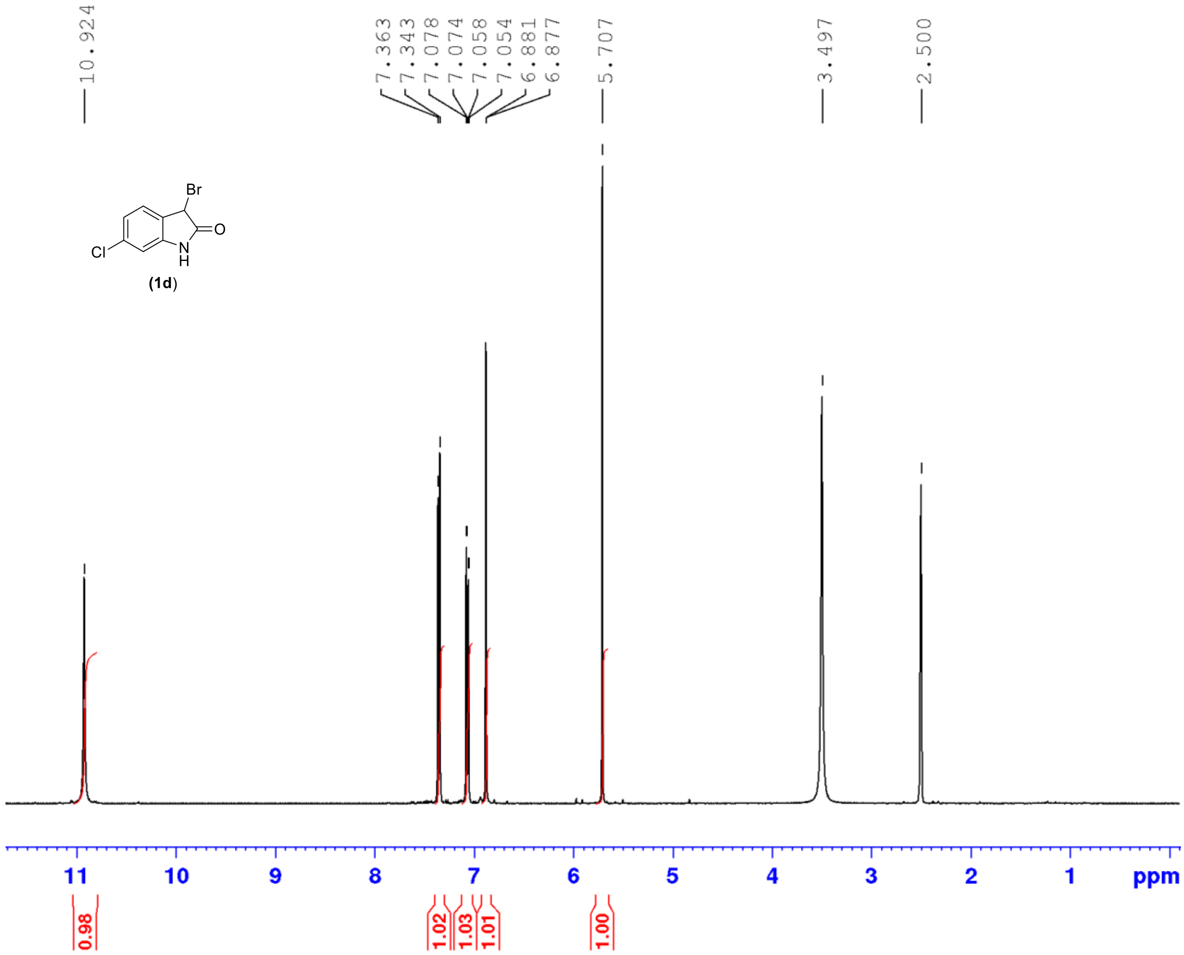
10.924
7.363
7.343
7.078
7.074
7.058
7.054
6.881
6.877
5.707
3.497
2.500
Br
O
Cl
N
H
(1d)
11 10 9 8 7 6 5 4 3 2 1 ppm
0.98
1.02
1.03
1.01
1.00

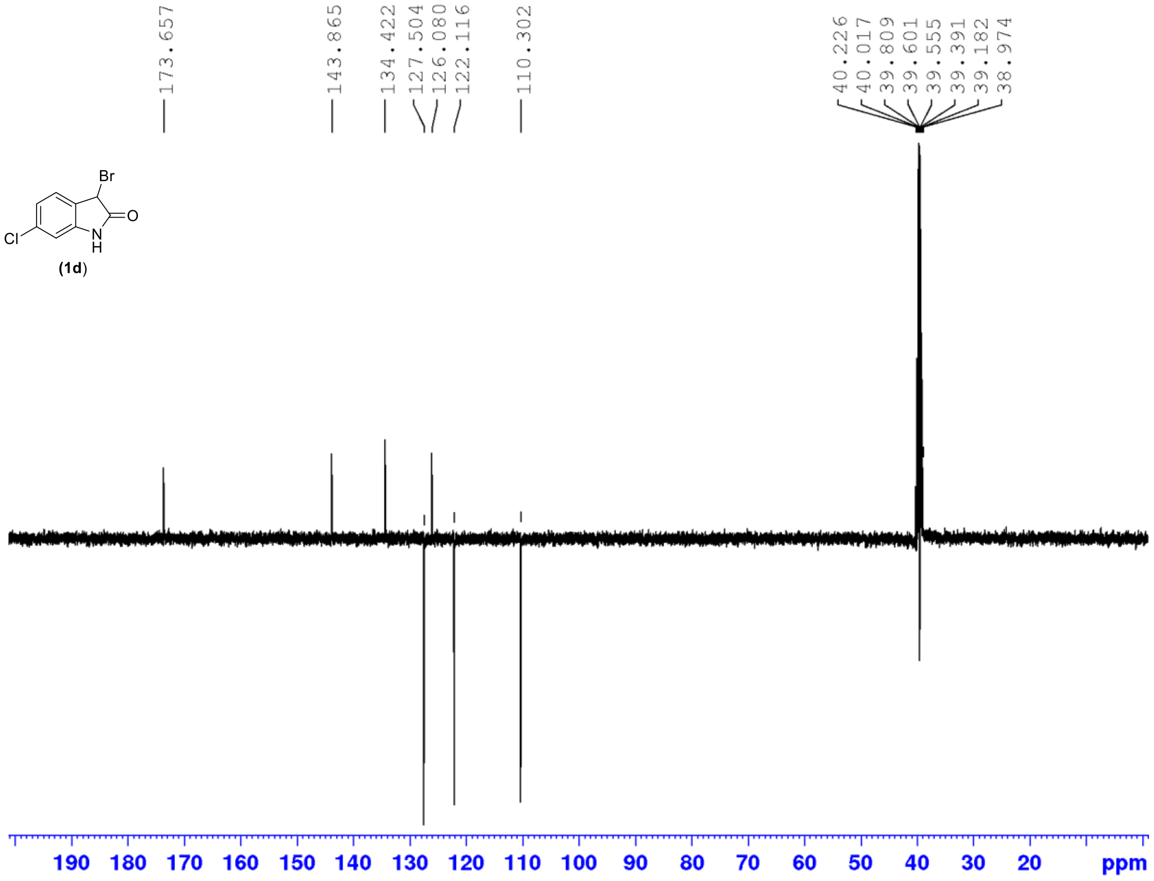
173.657
143.865
134.422
127.504
126.080
122.116
110.302
40.226
40.017
39.809
39.601
39.555
39.391
39.182
38.974
Br
O
Cl
N
H
(1d)
190 180 170 160 150 140 130 120 110 100 90 80 70 60 50 40 30 20 ppm

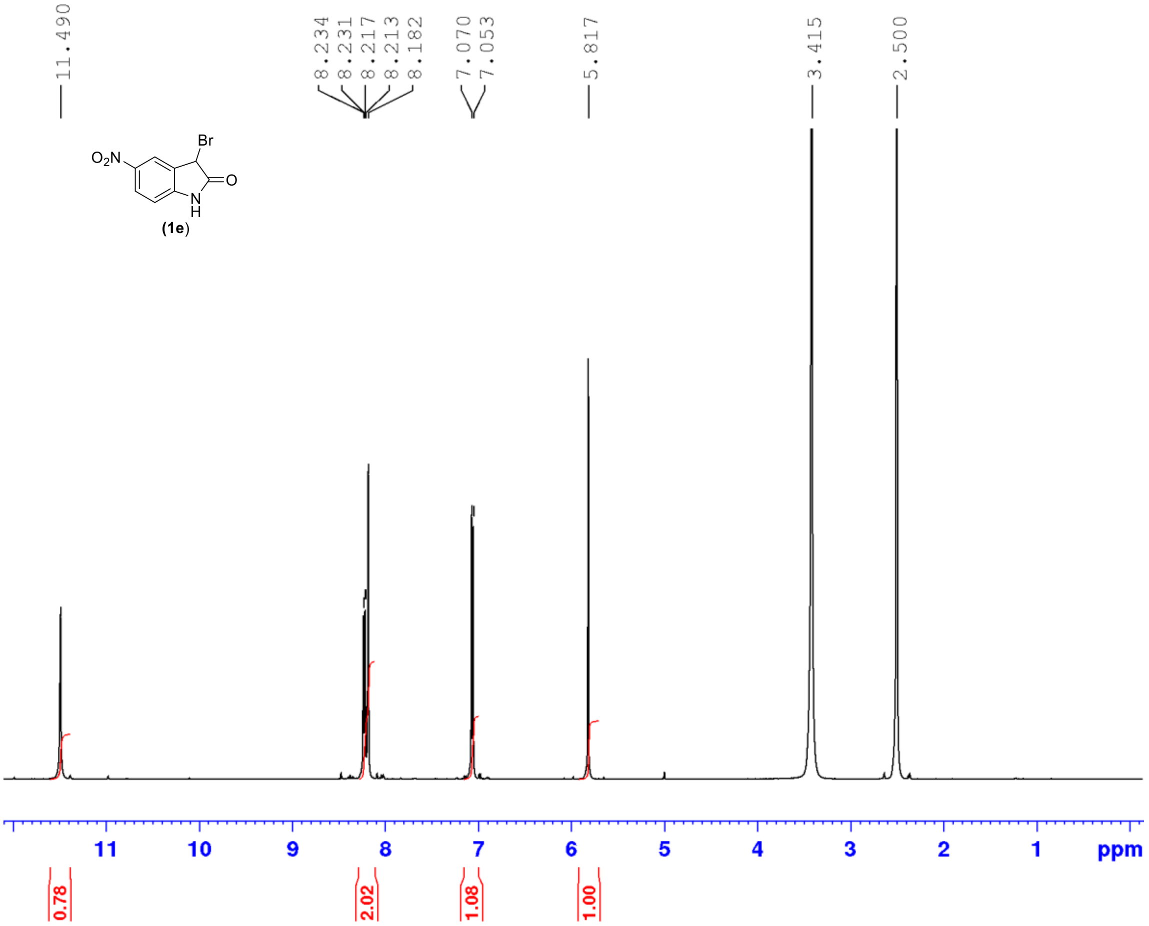
11.490
8.234
8.231
8.217
8.213
8.182
7.070
7.053
5.817
3.415
2.500
O2N
Br
O
N
H
(1e)
11
10
9
8
7
6
5
4
3
2
1
ppm
0.78
2.02
1.08
1.00

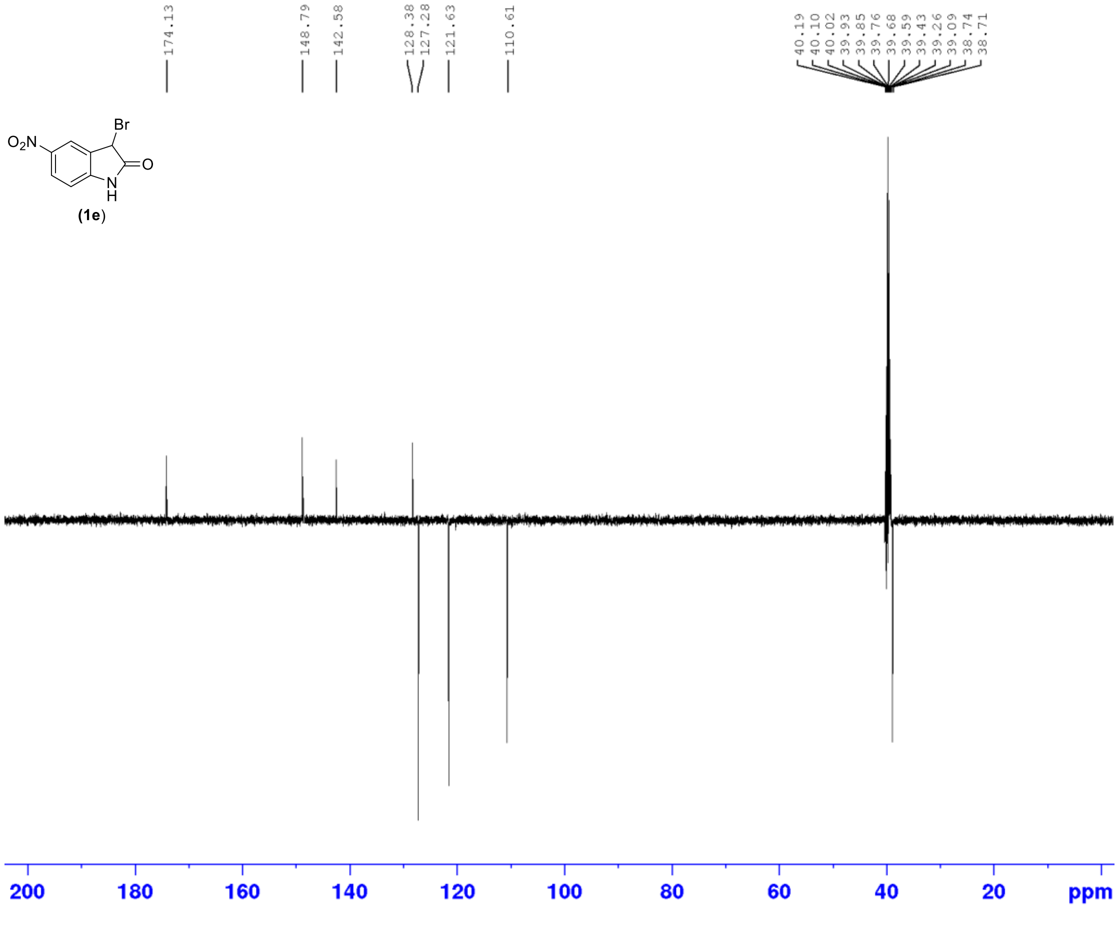
174.13
148.79
142.58
128.38
127.28
121.63
110.61
40.19
40.10
40.02
39.93
39.85
39.76
39.68
39.59
39.43
39.26
39.09
38.74
38.71
O2N
Br
O
N
H
(1e)
200
180
160
140
120
100
80
60
40
20
ppm

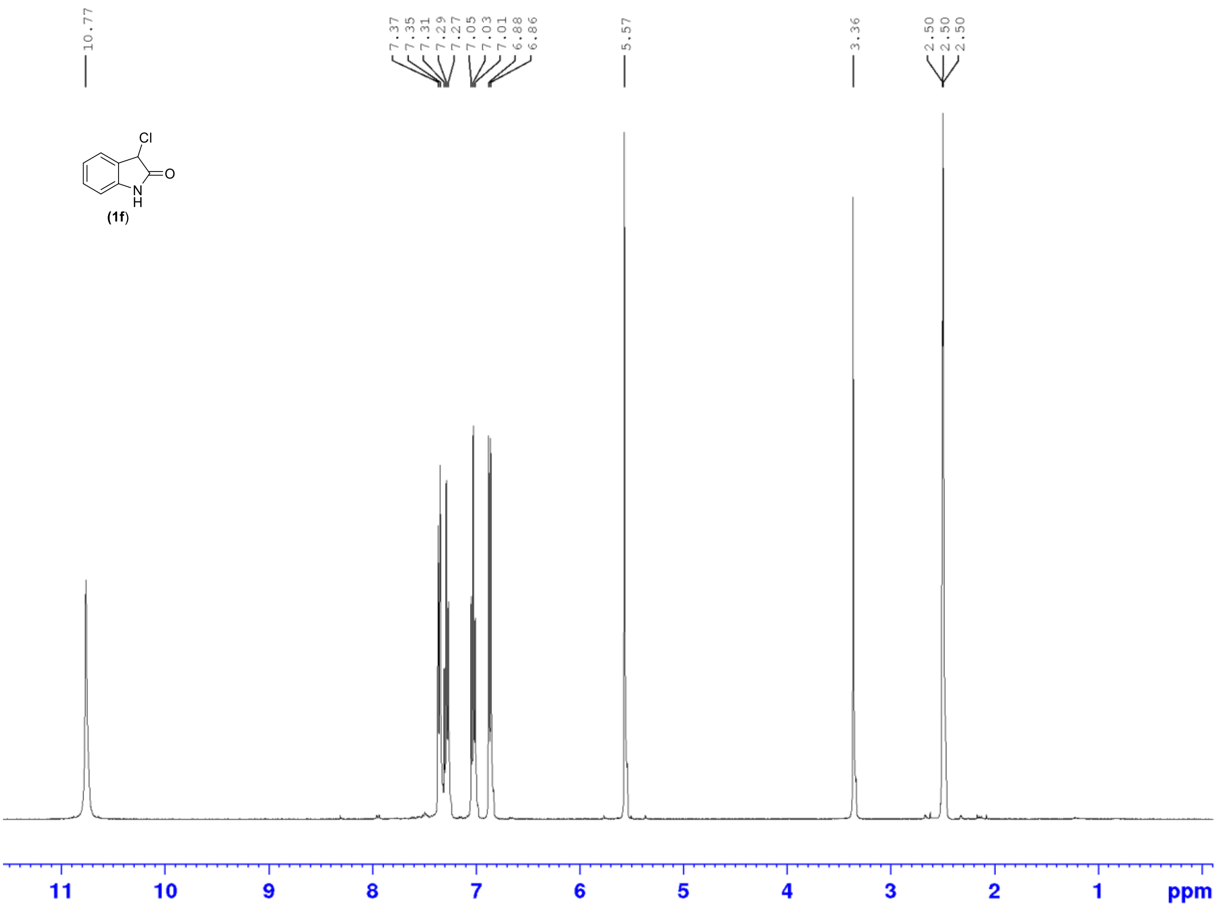
10.77
7.37
7.35
7.31
7.29
7.27
7.05
7.03
7.01
6.88
6.86
5.57
3.36
2.50
2.50
2.50
Cl
O
N
H
(1f)
11
10
9
8
7
6
5
4
3
2
1
ppm

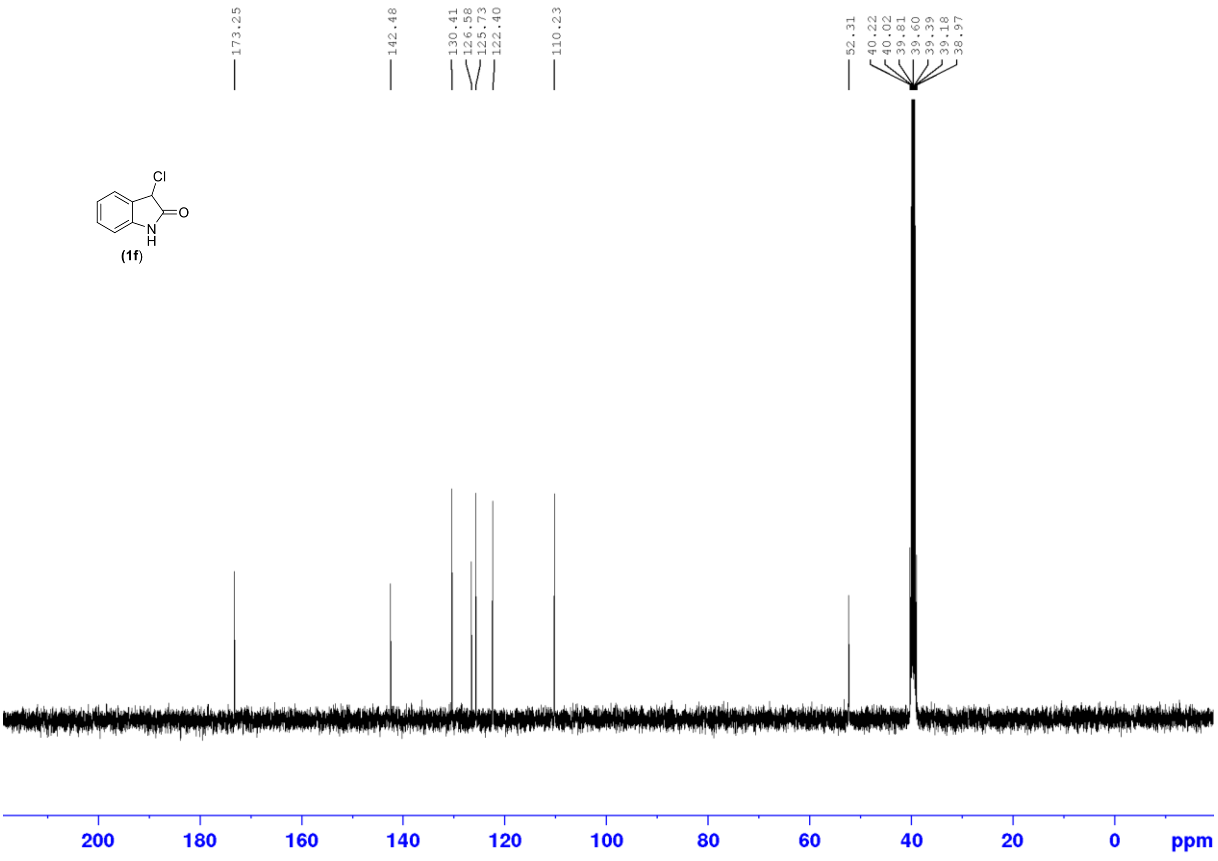
173.25
142.48
130.41
126.58
125.73
122.40
110.23
52.31
40.22
40.02
39.81
39.60
39.39
39.18
38.97
Cl
O
N
H
(1f)
200
180
160
140
120
100
80
60
40
20
0
ppm

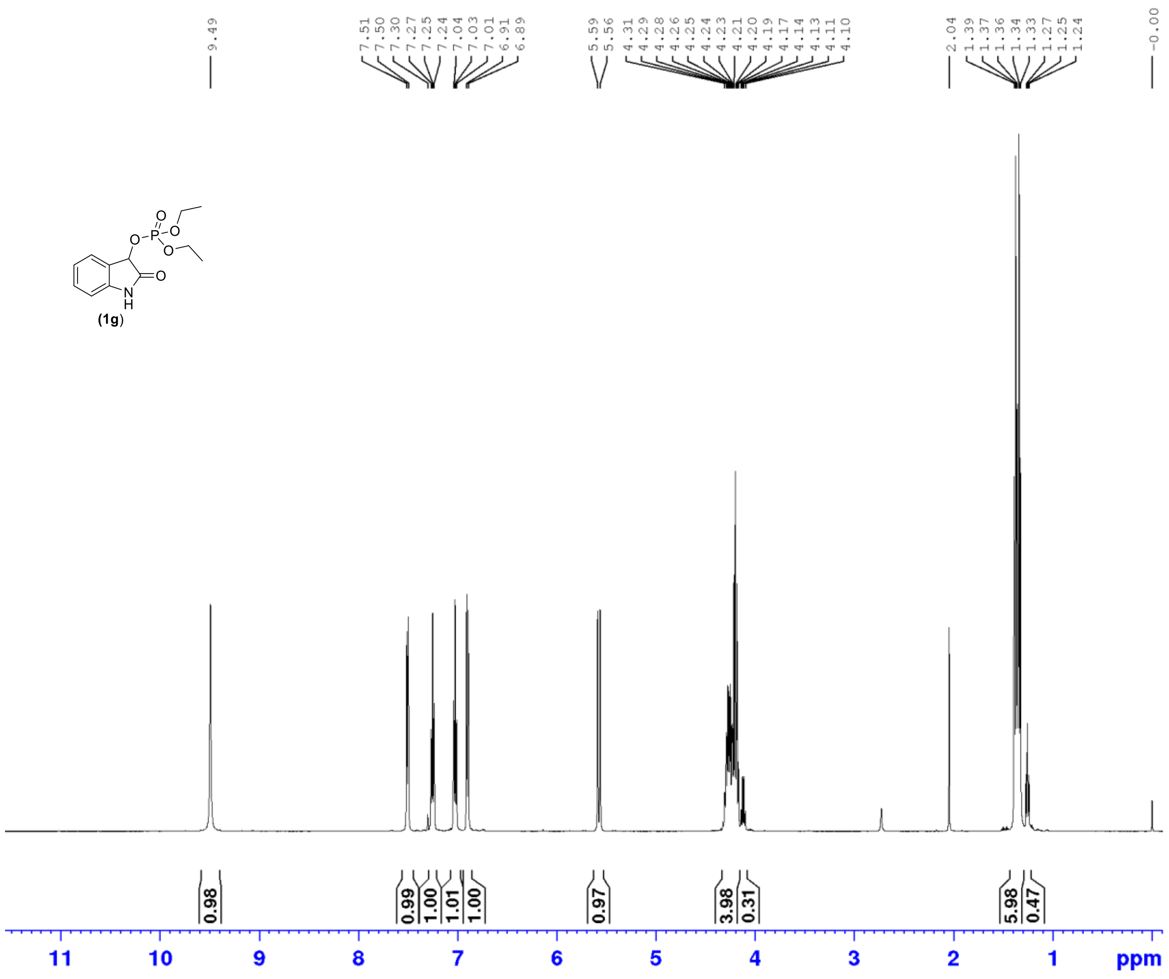

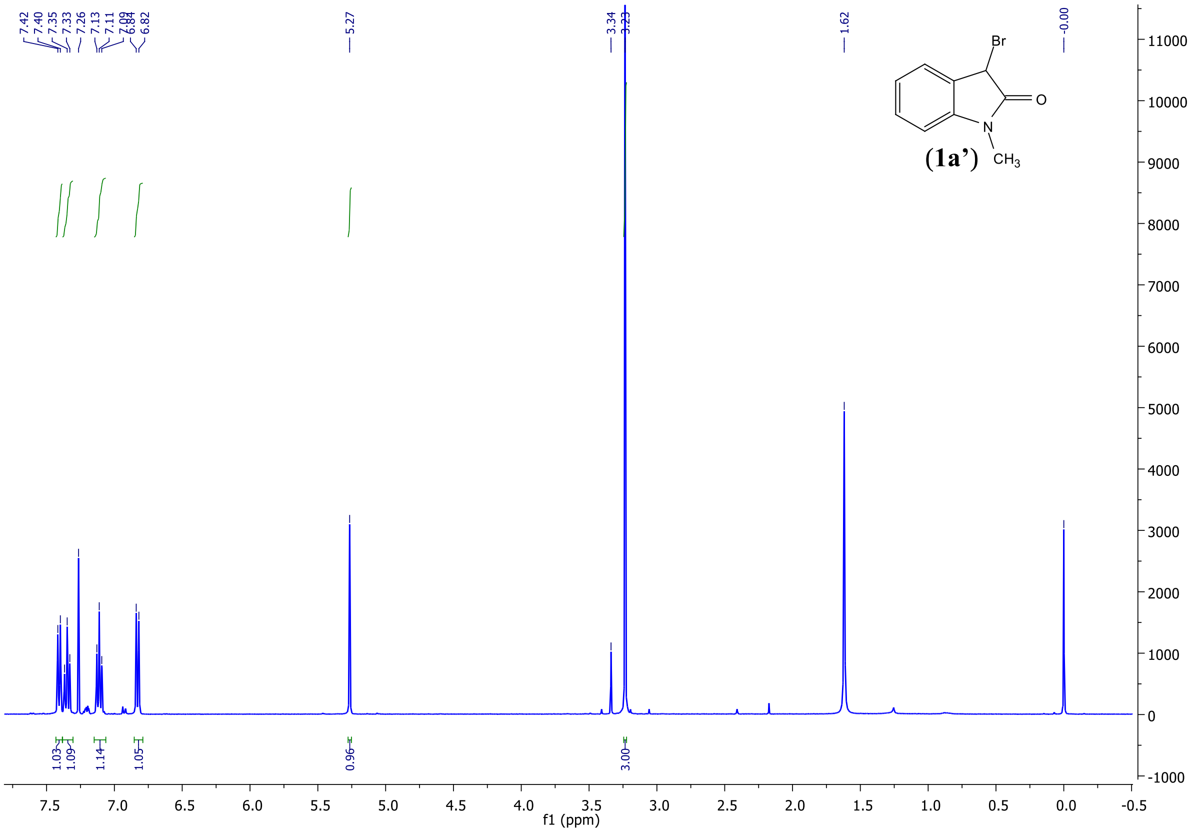

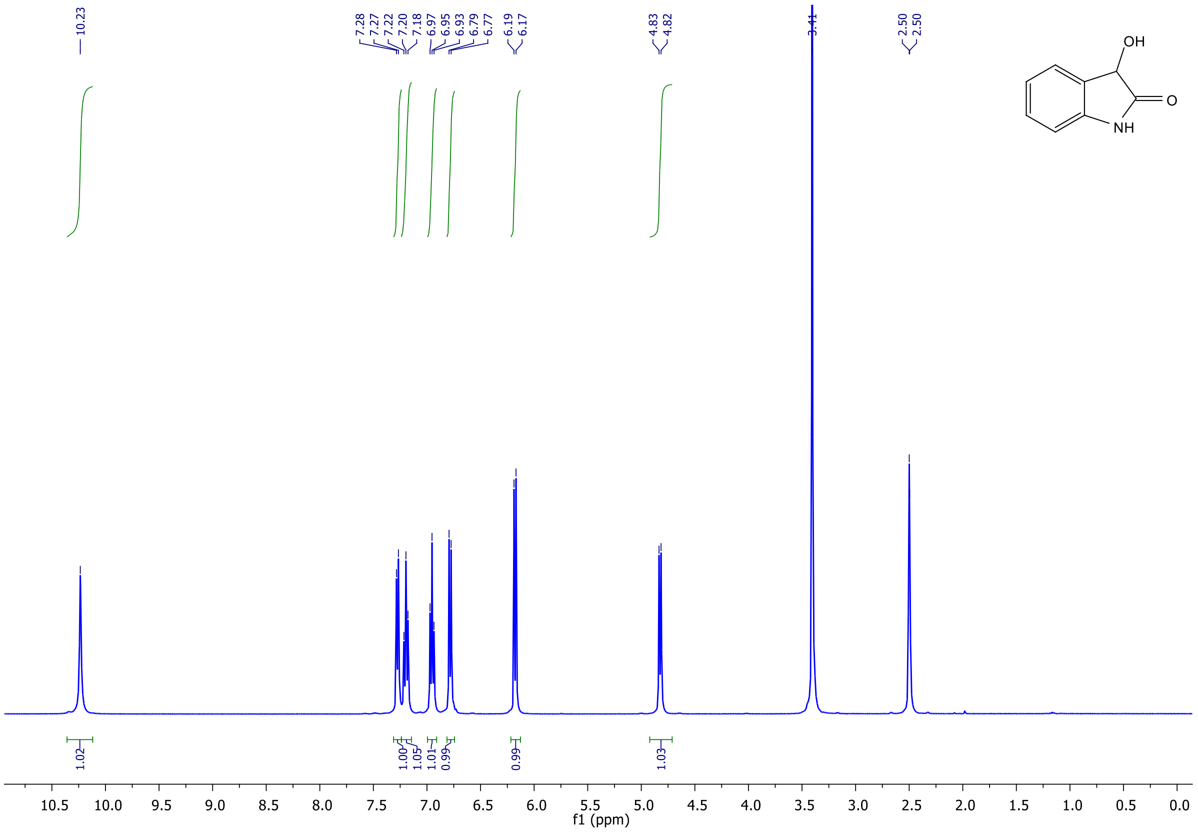
10.23
7.28
7.27
7.22
7.20
7.18
6.97
6.95
6.93
6.79
6.77
6.19
6.17
4.83
4.82
3.41
2.50
2.50
OH
O
NH
1.02
1.00
1.05
1.01
0.99
0.99
1.03
f1 (ppm)

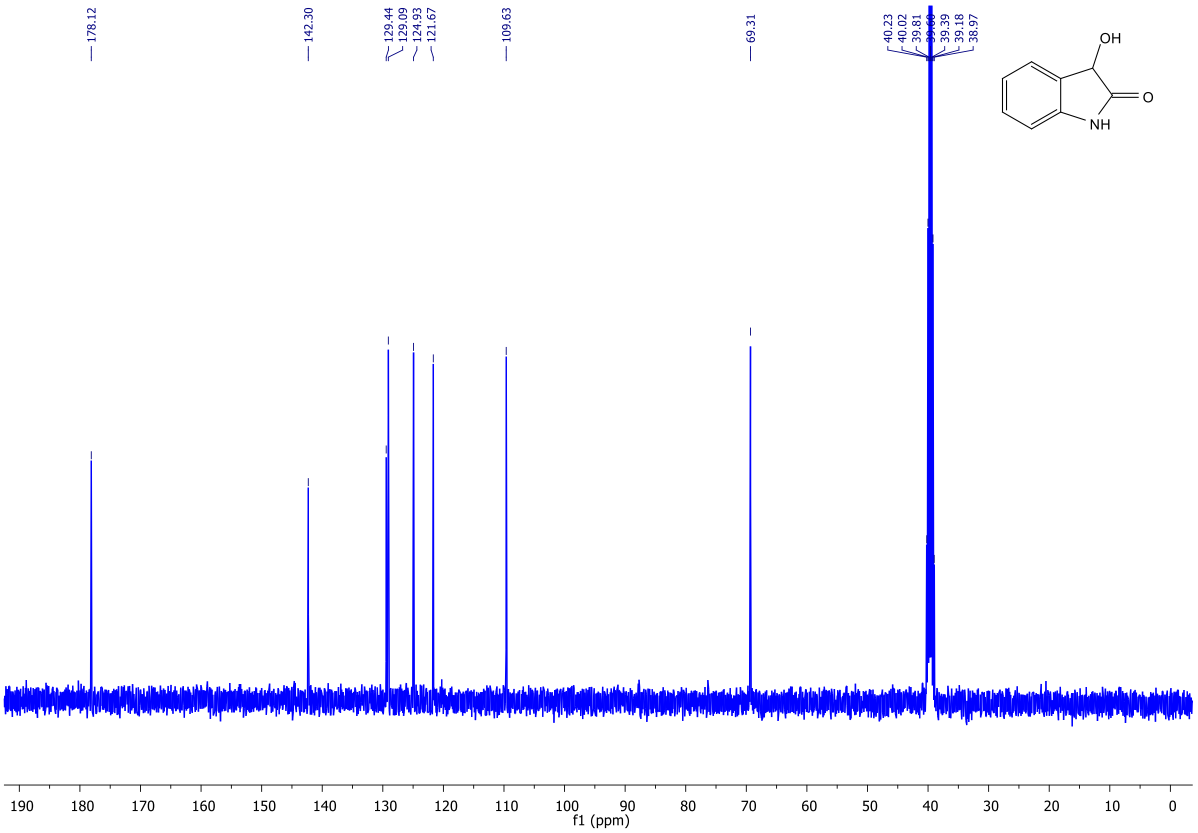
178.12
142.30
129.44
129.09
124.93
121.67
109.63
69.31
40.23
40.02
39.81
39.39
39.18
38.97
OH
O
NH
f1 (ppm)

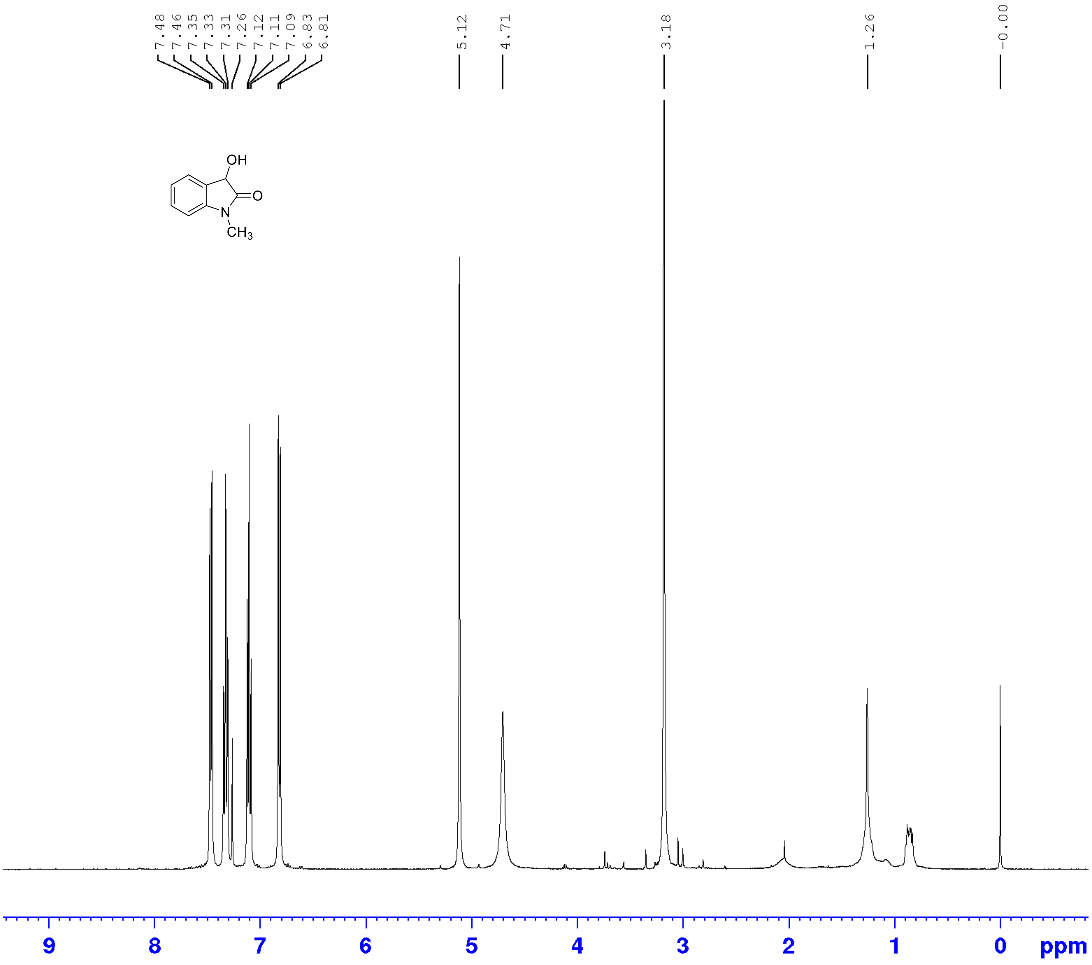
7.48
7.46
7.35
7.33
7.31
7.26
7.12
7.11
7.09
6.83
6.81
5.12
4.71
3.18
1.26
-0.00
OH
O
N
CH3
9
8
7
6
5
4
3
2
1
0
ppm

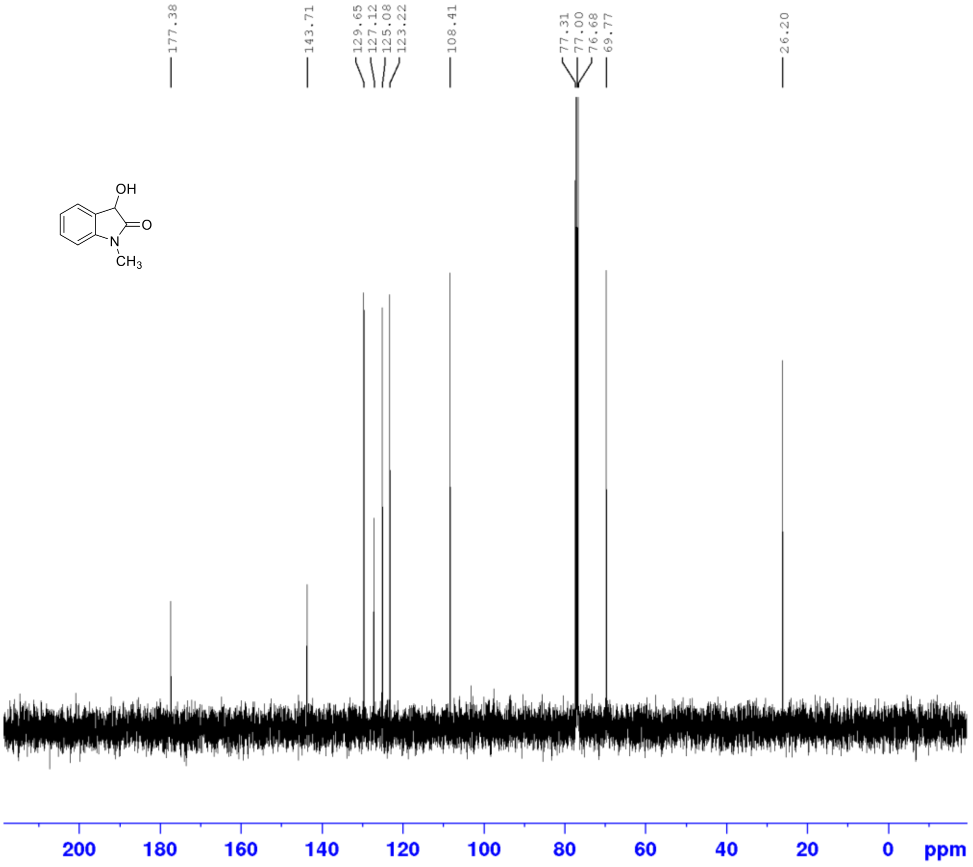
177.38
143.71
129.65
127.12
125.08
123.22
108.41
77.31
77.00
76.68
69.77
26.20
OH
O
N
CH3
200
180
160
140
120
100
80
60
40
20
0
ppm

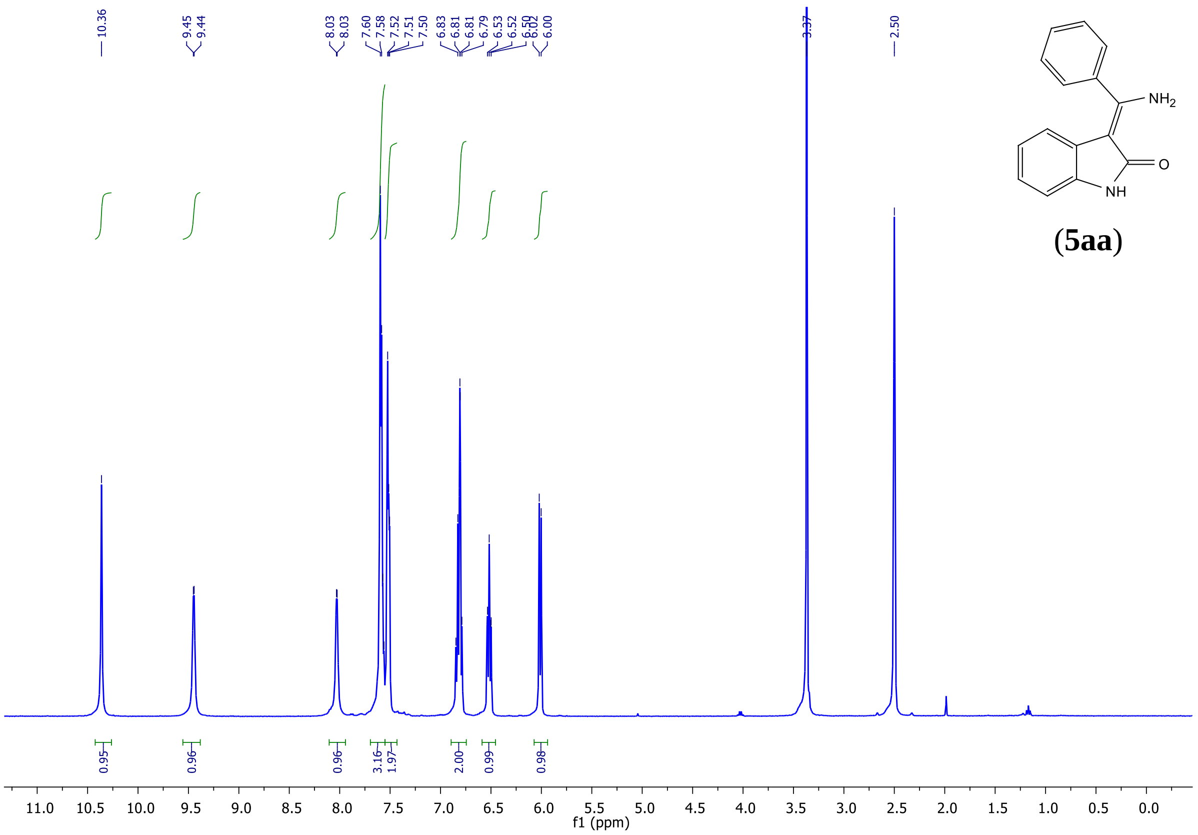

(5aa)

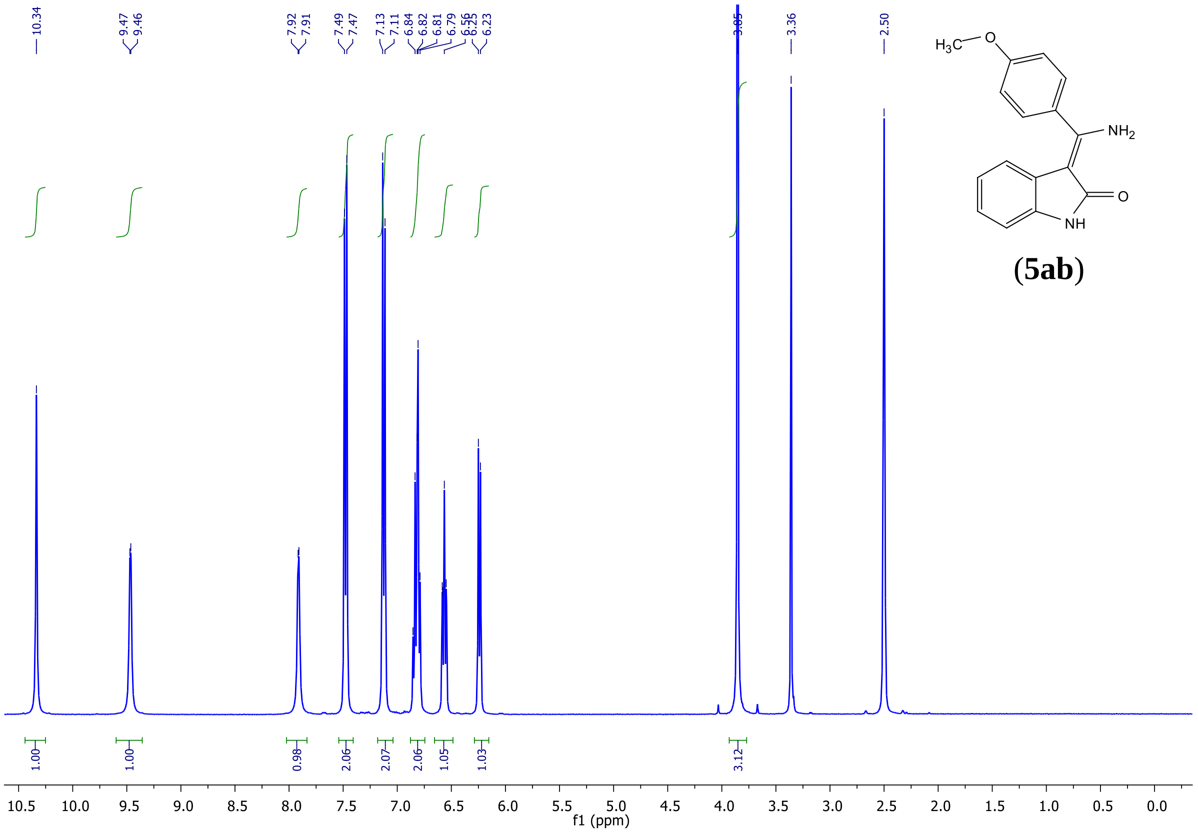

(5ab)

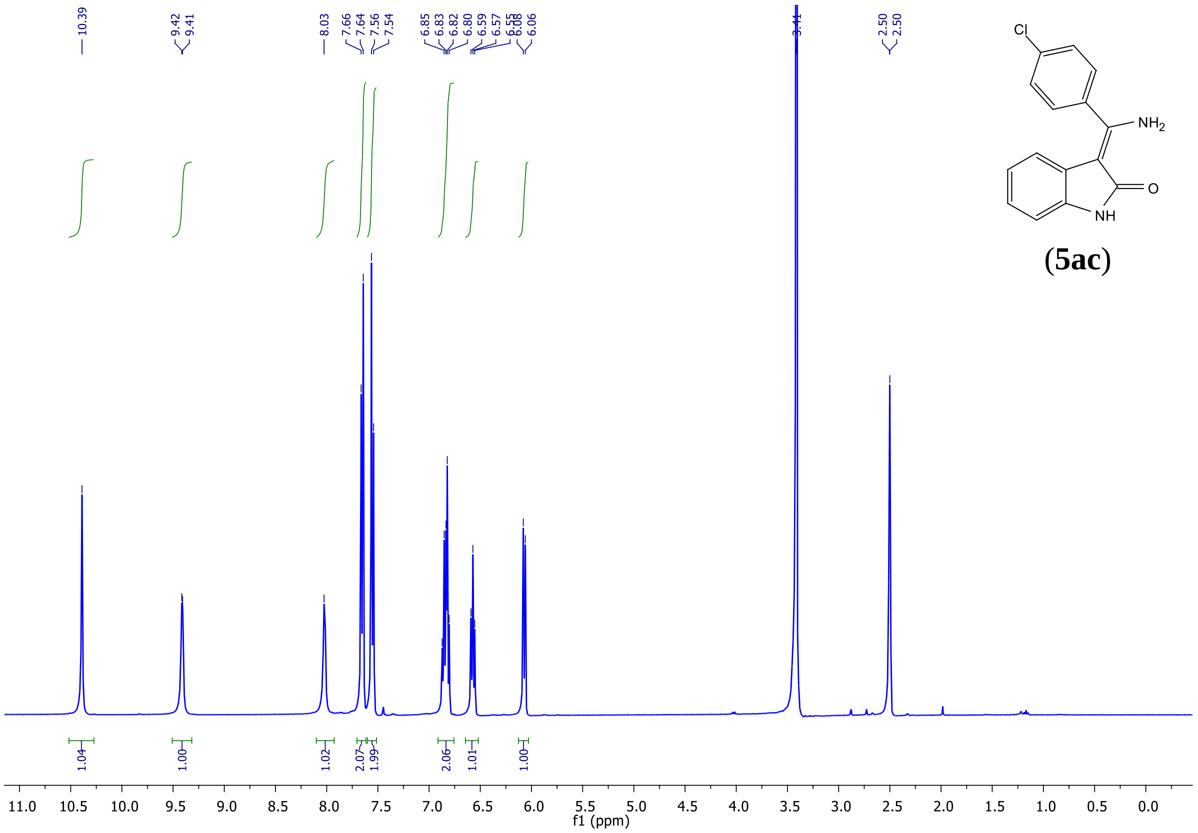
Cl
NH2
O
NH
(5ac)
f1 (ppm)

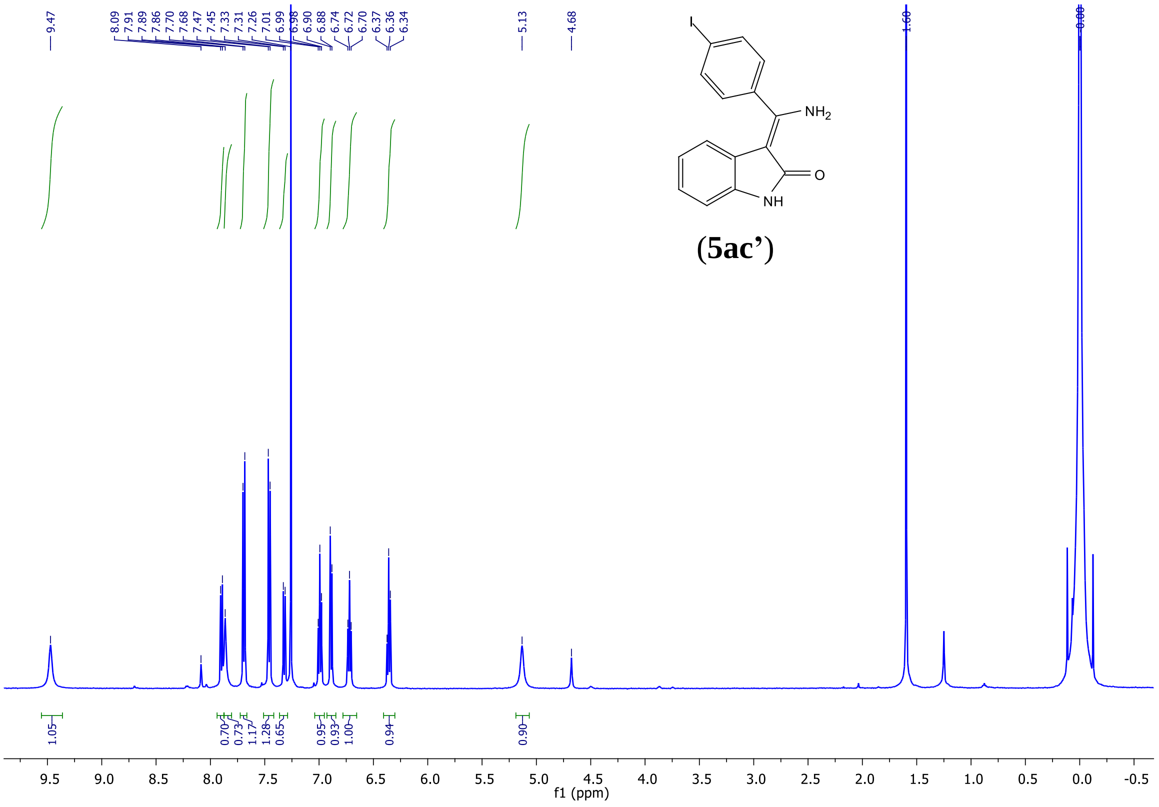
I
NH2
O
NH
(5ac')
f1 (ppm)

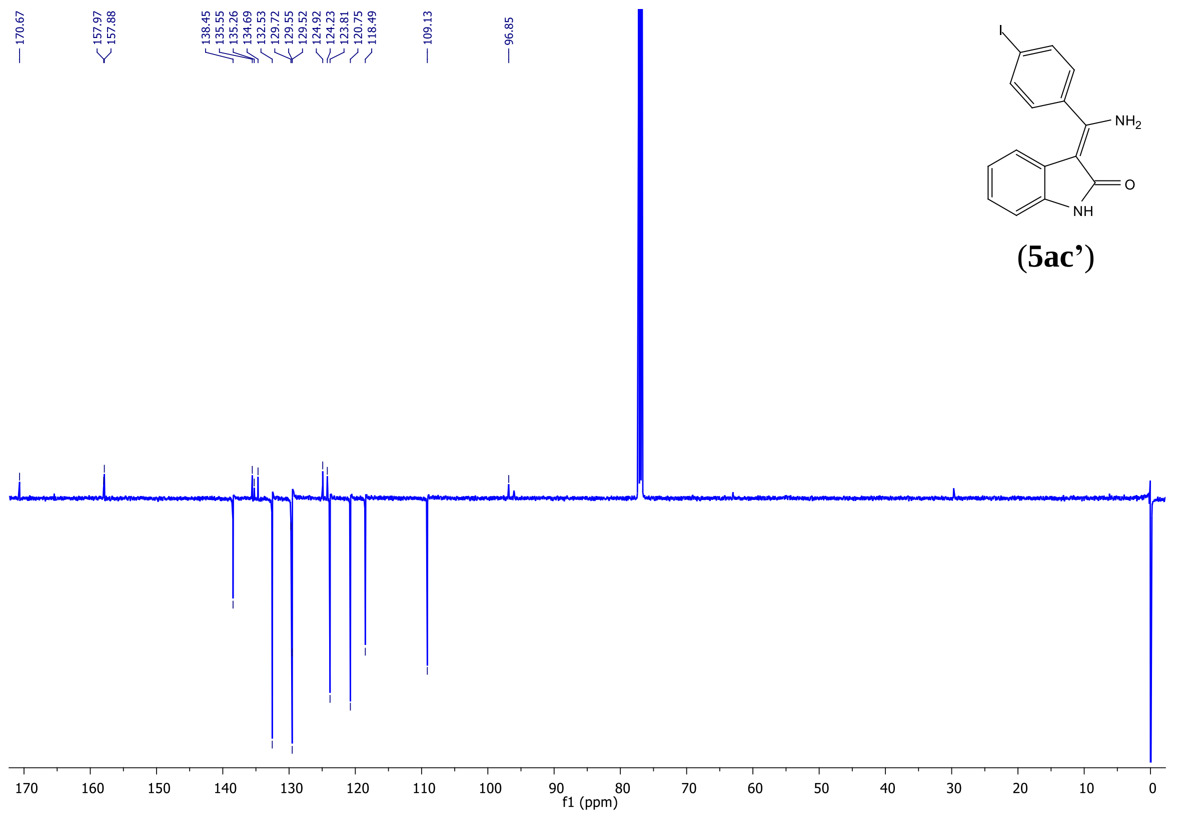

(5ac’)

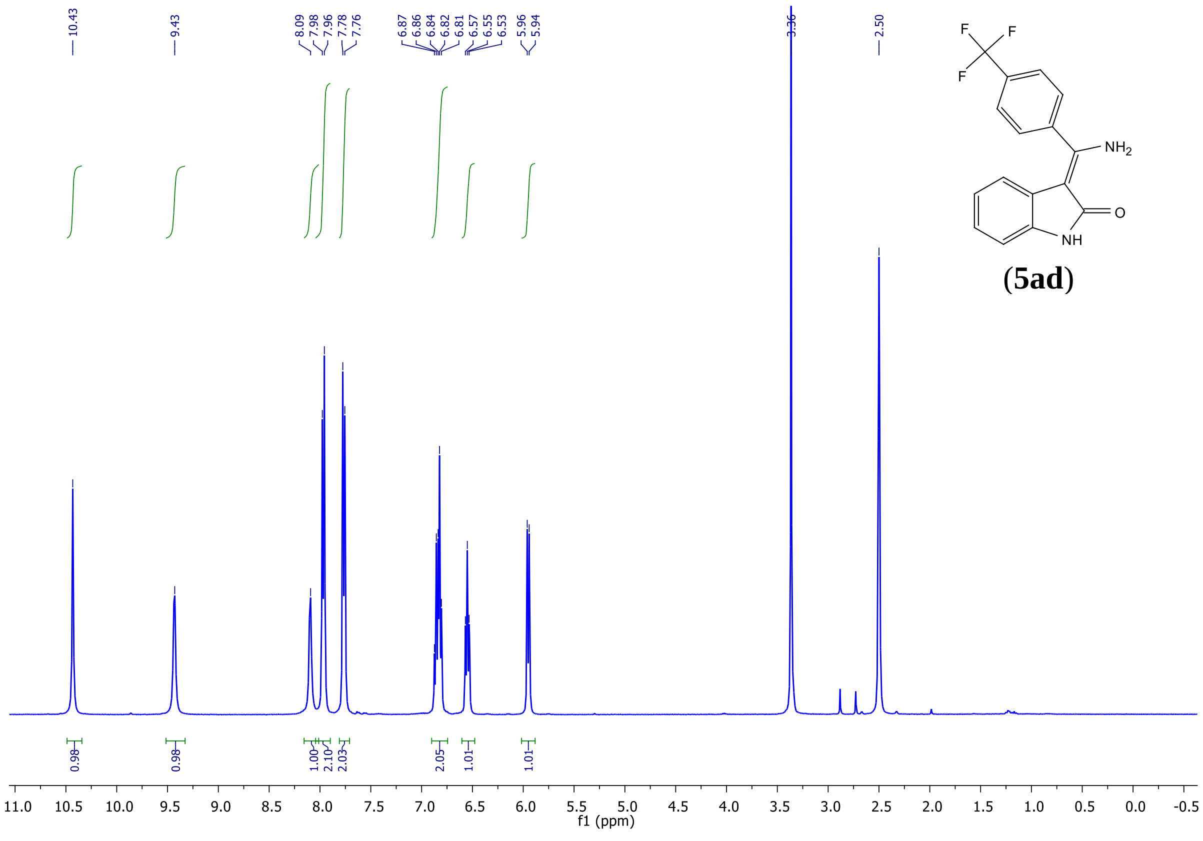

(5ad)

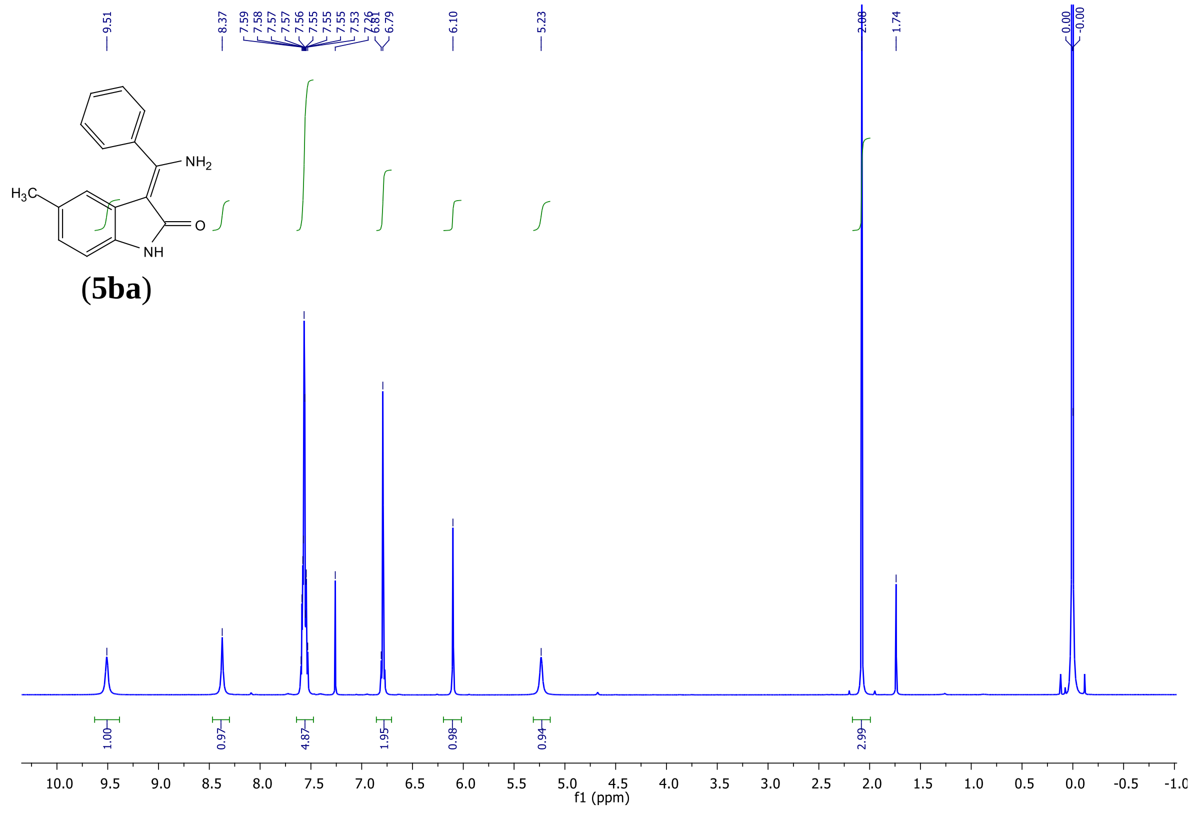
(5ba)
NH2
H3C
O
NH
9.51
8.37
7.59
7.58
7.57
7.57
7.56
7.55
7.55
7.55
7.53
7.26
6.81
6.79
6.10
5.23
2.08
1.74
0.00
-0.00
1.00
0.97
4.87
1.95
0.98
0.94
2.99
f1 (ppm)

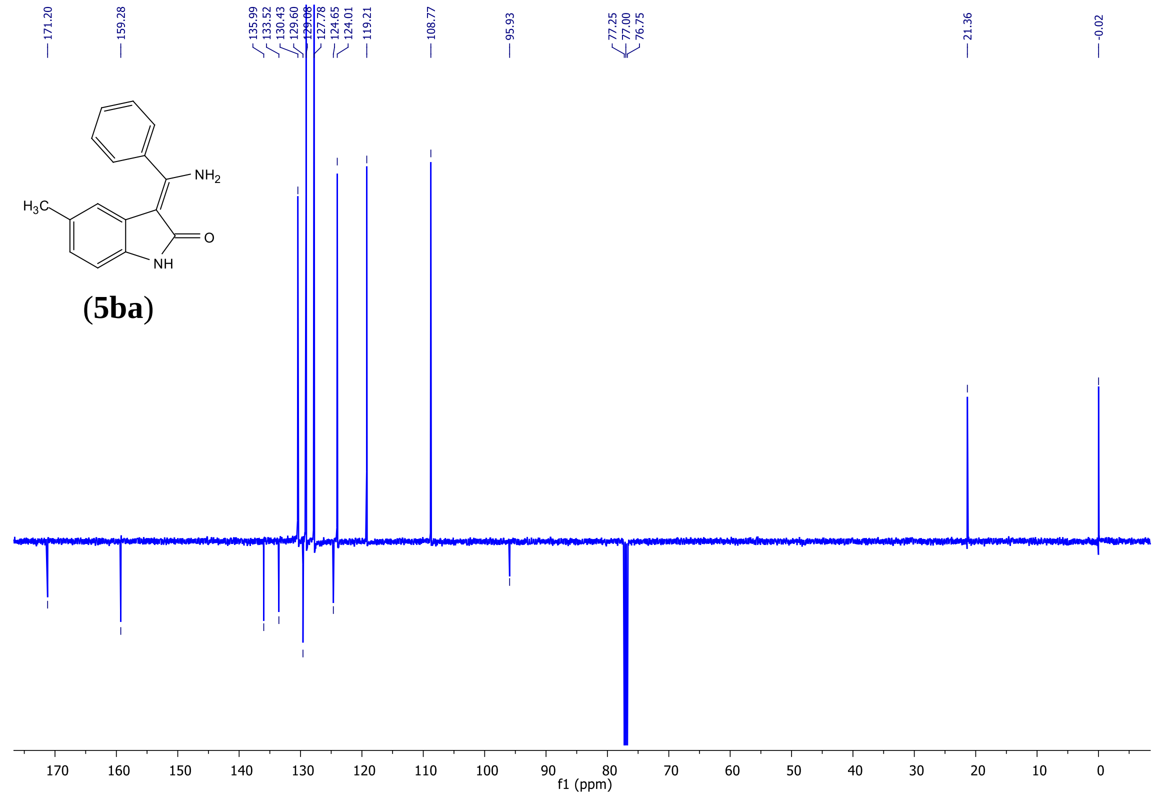
(5ba)
NH2
H3C
O
NH
171.20
159.28
135.99
133.52
130.43
129.60
129.08
127.78
124.65
124.01
119.21
108.77
95.93
77.25
77.00
76.75
21.36
-0.02
f1 (ppm)

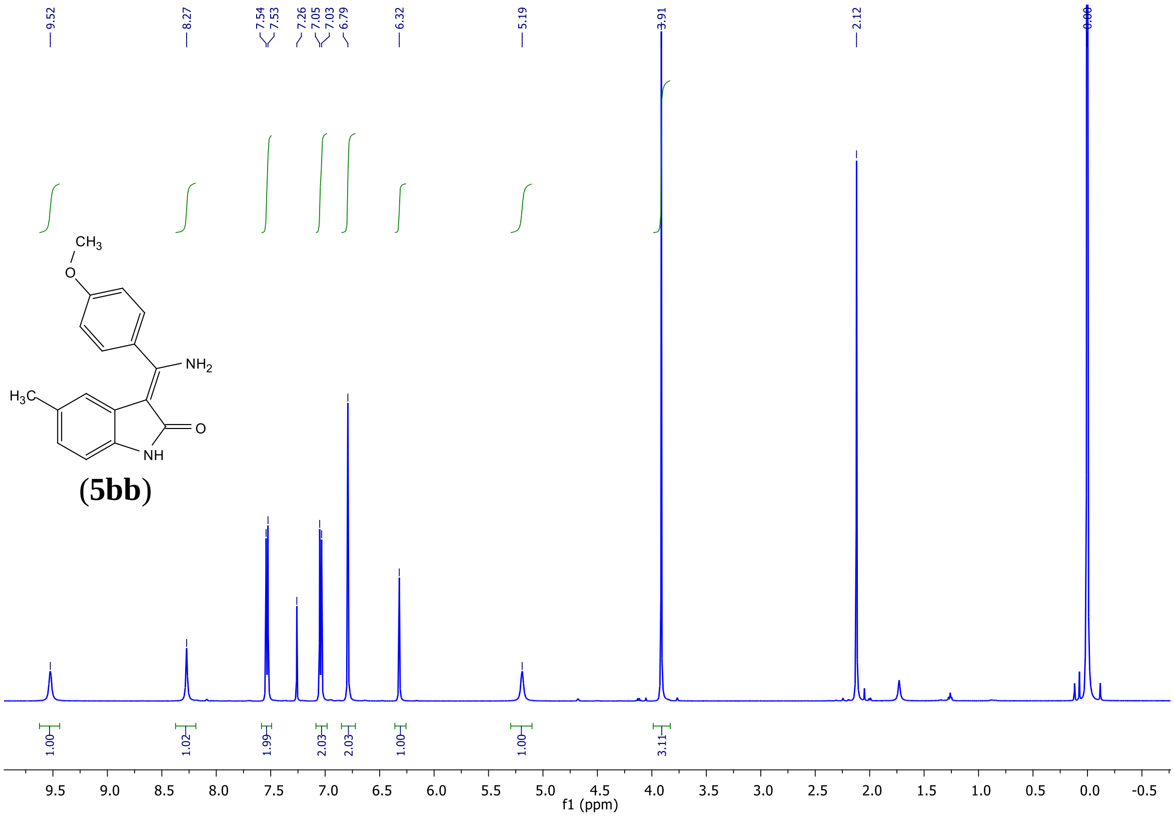
9.52
8.27
7.54
7.53
7.26
7.05
7.03
6.79
6.32
5.19
3.91
2.12
0.00
CH3
O
NH2
H3C
O
NH
(5bb)
1.00
1.02
1.99
2.03
2.03
1.00
1.00
3.11
f1 (ppm)

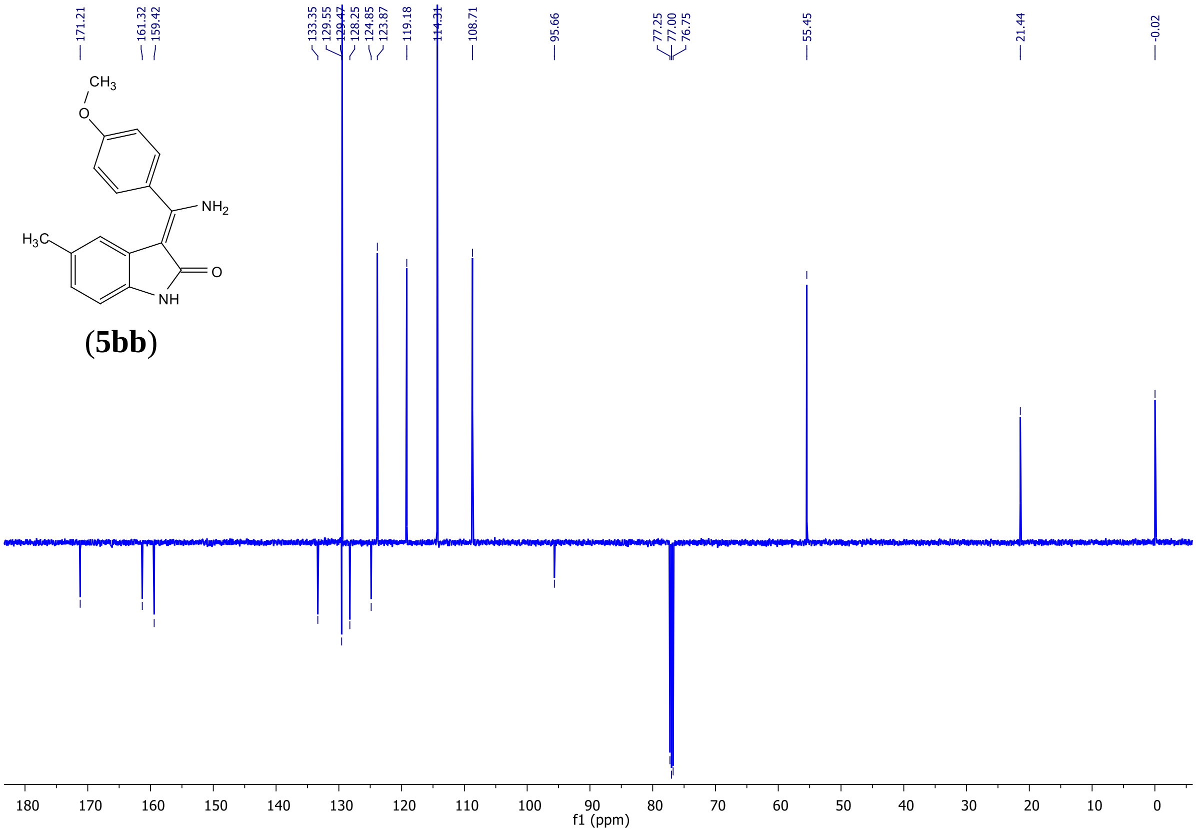
171.21
161.32
159.42
133.35
129.55
129.47
128.25
124.85
123.87
119.18
114.31
108.71
95.66
77.25
77.00
76.75
55.45
21.44
-0.02
CH3
O
NH2
H3C
O
NH
(5bb)
f1 (ppm)

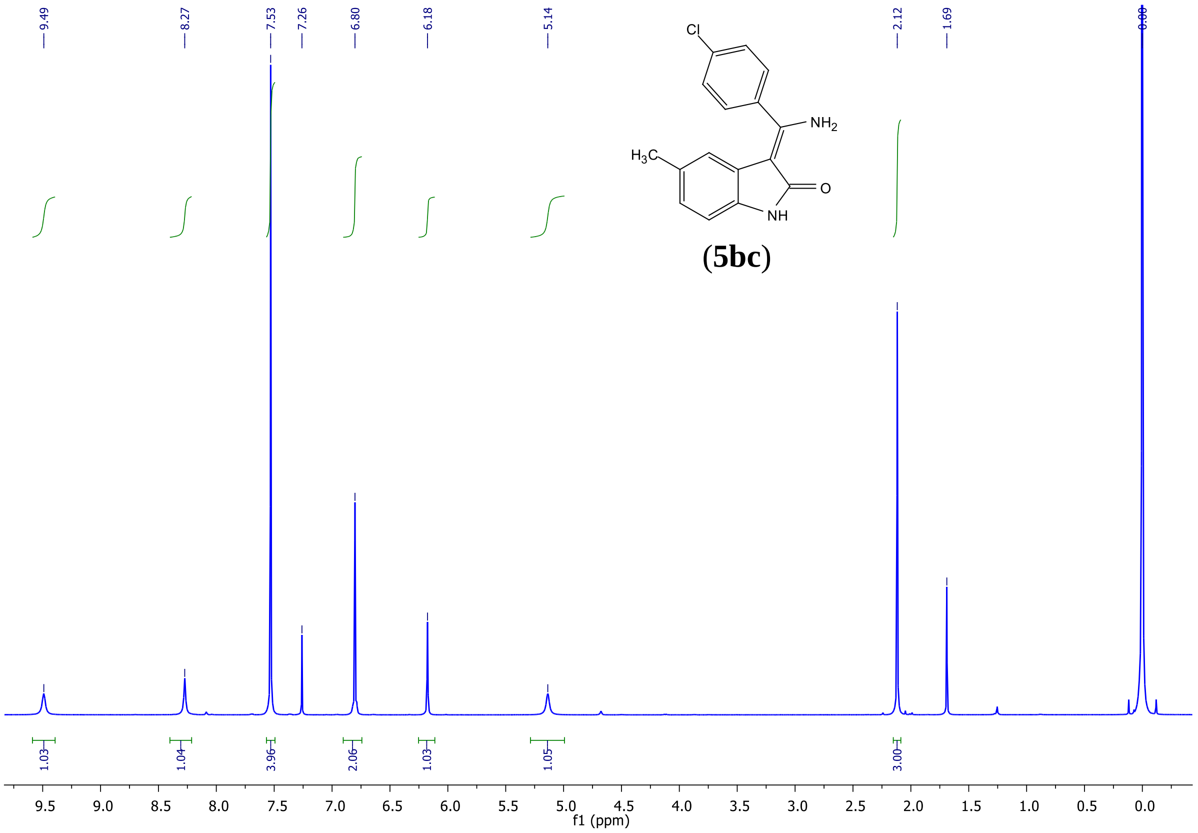

9.49
8.27
7.53
7.26
6.80
6.18
5.14
2.12
1.69
0.00
Cl
NH2
H3C
O
NH
(5bc)
1.03
1.04
3.96
2.06
1.03
1.05
3.00
f1 (ppm)

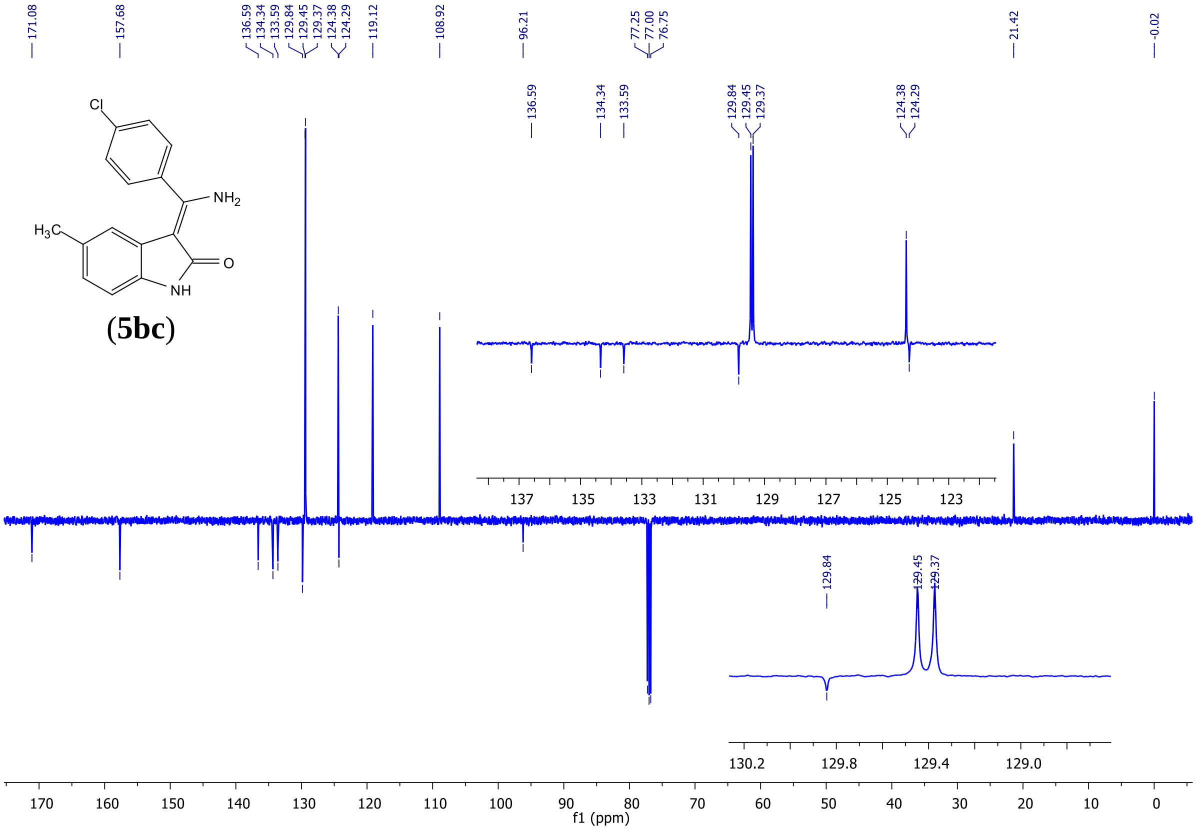

171.08
157.68
136.59
134.34
133.59
129.84
129.45
129.37
124.38
124.29
119.12
108.92
96.21
77.25
77.00
76.75
21.42
-0.02
Cl
NH2
H3C
O
NH
(5bc)
f1 (ppm)

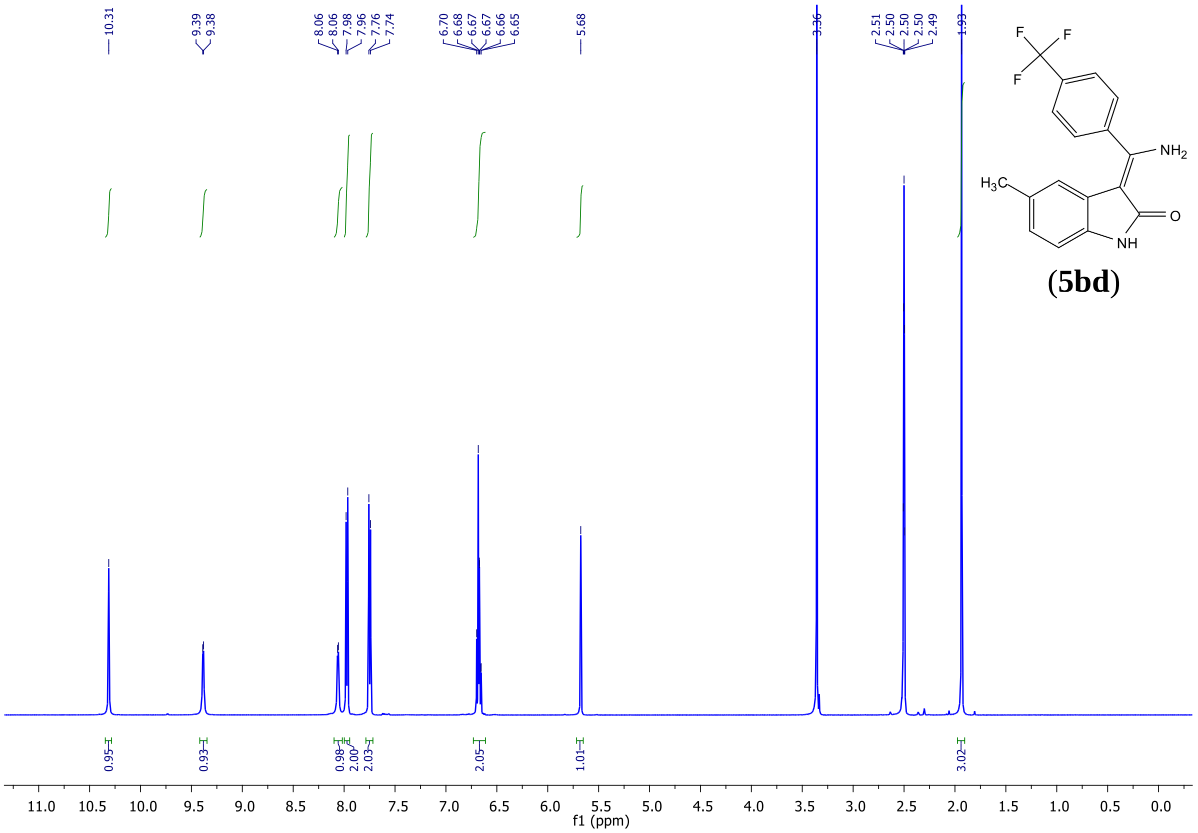
10.31
9.39
9.38
8.06
8.06
7.98
7.96
7.76
7.74
6.70
6.68
6.67
6.67
6.66
6.65
5.68
3.36
2.51
2.50
2.50
2.50
2.49
1.93
0.95
0.93
0.98
2.00
2.03
2.05
1.01
3.02
f1 (ppm)
F
F
F
NH_2
H_3C
O
NH

(5bd)

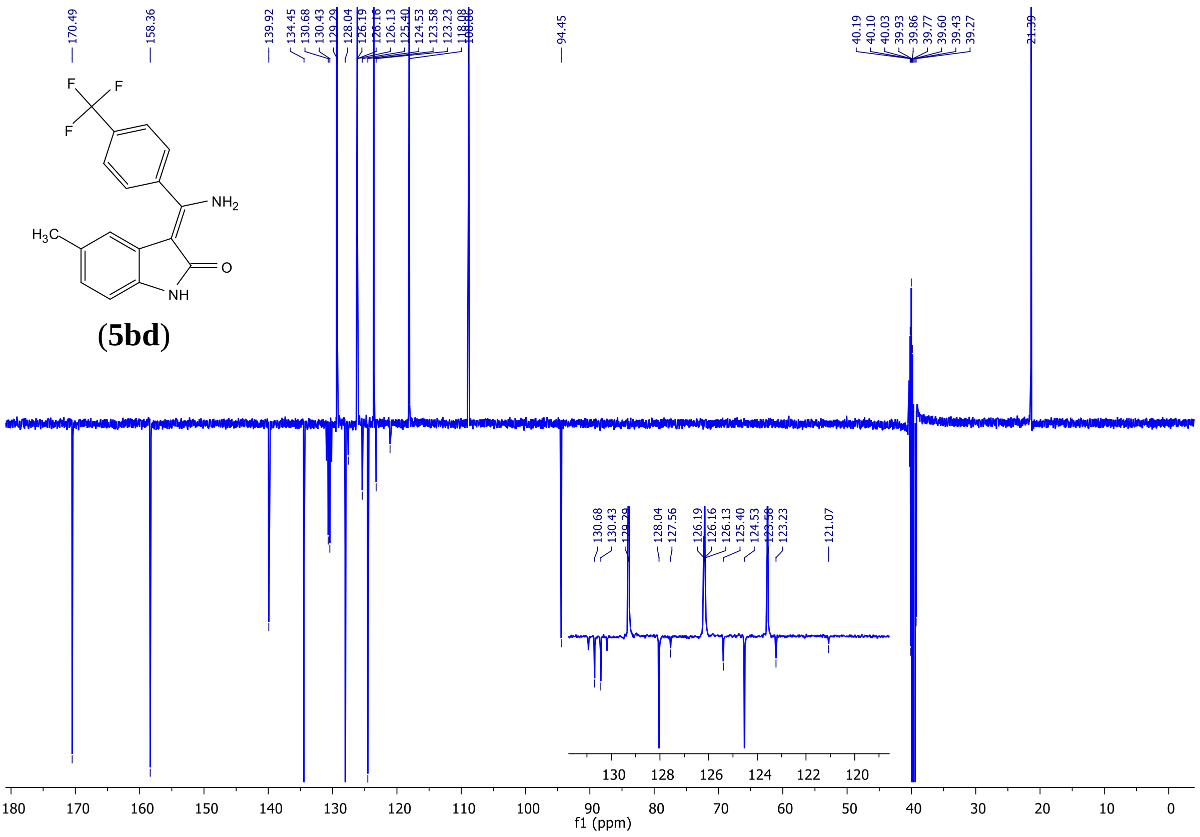
170.49
158.36
139.92
134.45
130.68
130.43
129.29
128.04
126.19
126.16
126.13
125.40
124.53
123.58
123.23
118.08
108.80
94.45
40.19
40.10
40.03
39.93
39.86
39.77
39.60
39.43
39.27
21.39
130.68
130.43
129.29
128.04
127.56
126.19
126.16
126.13
125.40
124.53
123.58
123.23
121.07
f1 (ppm)
F
F
F
NH_2
H_3C
O
NH

(5bd)

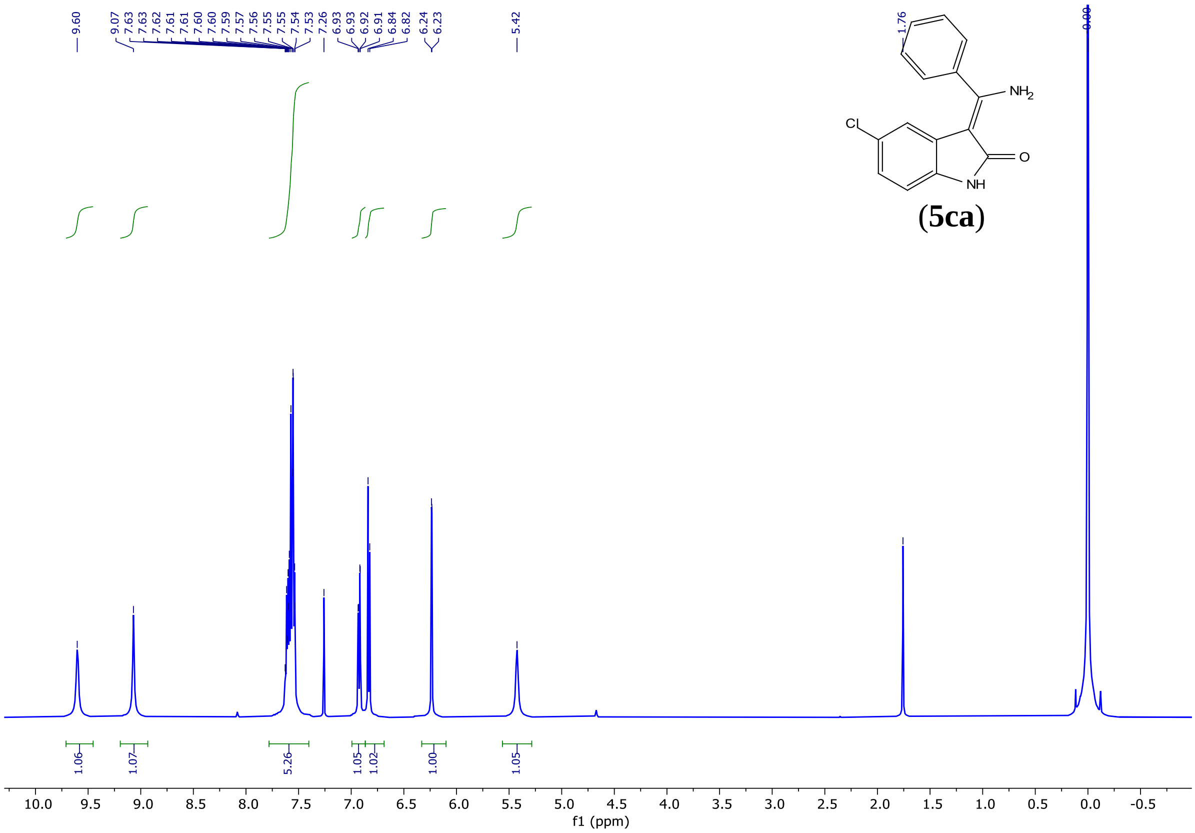
9.60
9.07
7.63
7.63
7.62
7.61
7.61
7.60
7.59
7.57
7.56
7.55
7.55
7.54
7.53
7.26
6.93
6.93
6.92
6.91
6.84
6.82
6.24
6.23
5.42
1.76
0.00
NH2
Cl
O
NH
(5ca)
1.06
1.07
5.26
1.05
1.02
1.00
1.05
f1 (ppm)

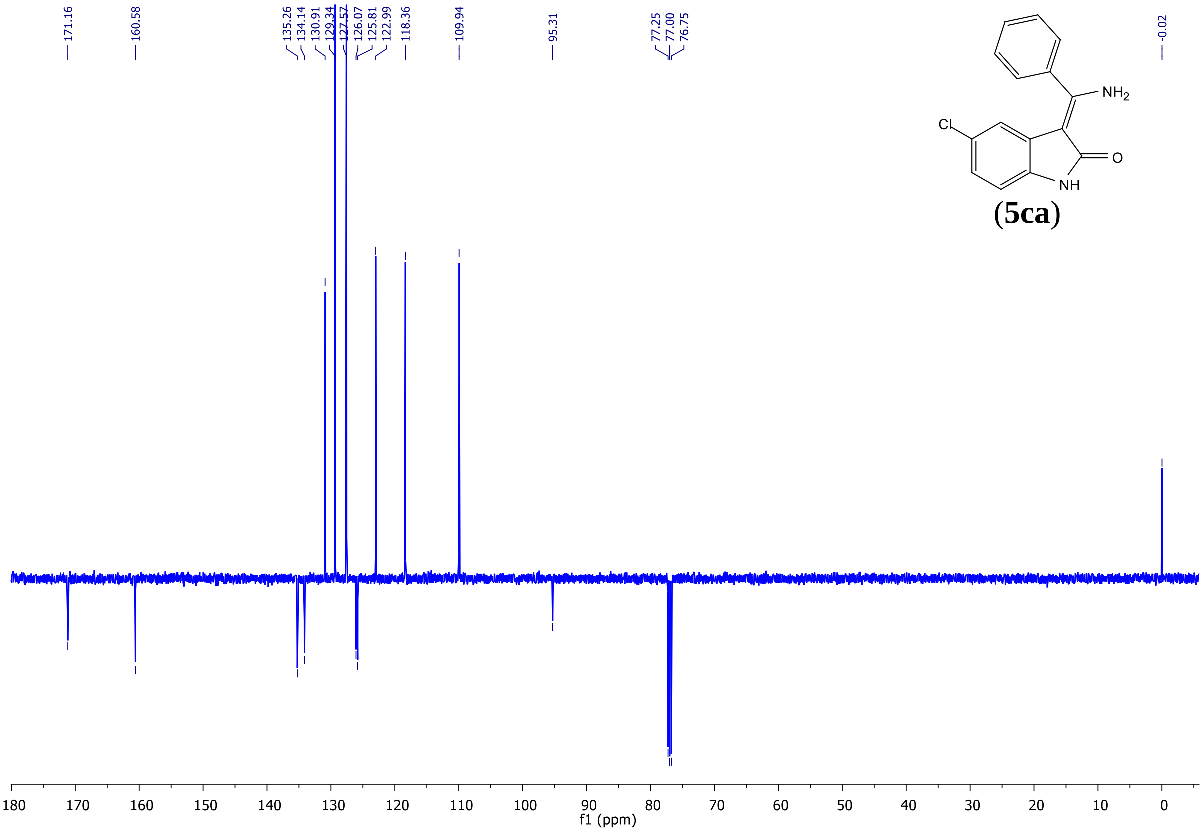
171.16
160.58
135.26
134.14
130.91
129.34
127.52
126.07
125.81
122.99
118.36
109.94
95.31
77.25
77.00
76.75
-0.02
NH2
Cl
O
NH
(5ca)
f1 (ppm)

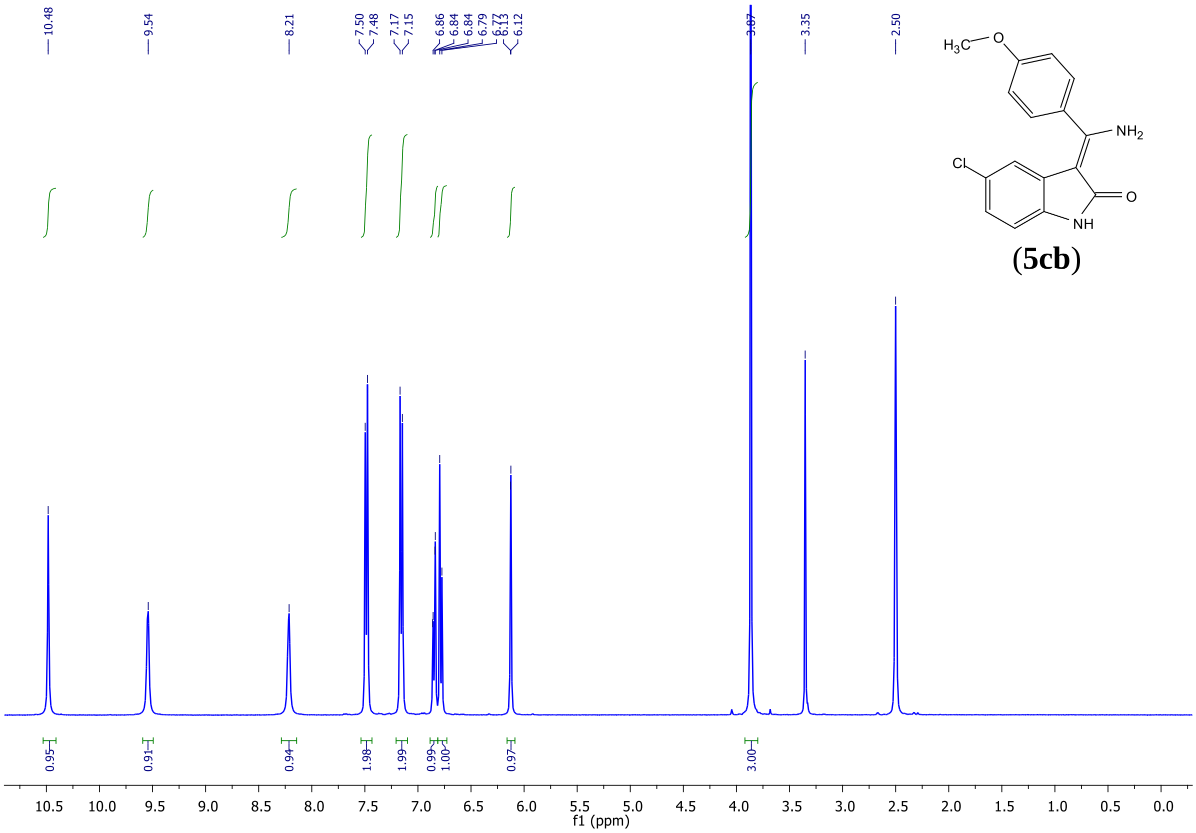
10.48
9.54
8.21
7.50
7.48
7.17
7.15
6.86
6.84
6.84
6.79
6.77
6.13
6.12
3.87
3.35
2.50
0.95
0.91
0.94
1.98
1.99
0.99
1.00
0.97
3.00
10.5 10.0 9.5 9.0 8.5 8.0 7.5 7.0 6.5 6.0 5.5 5.0 4.5 4.0 3.5 3.0 2.5 2.0 1.5 1.0 0.5 0.0
f1 (ppm)
H3C
O
NH2
Cl
O
NH
(5cb)

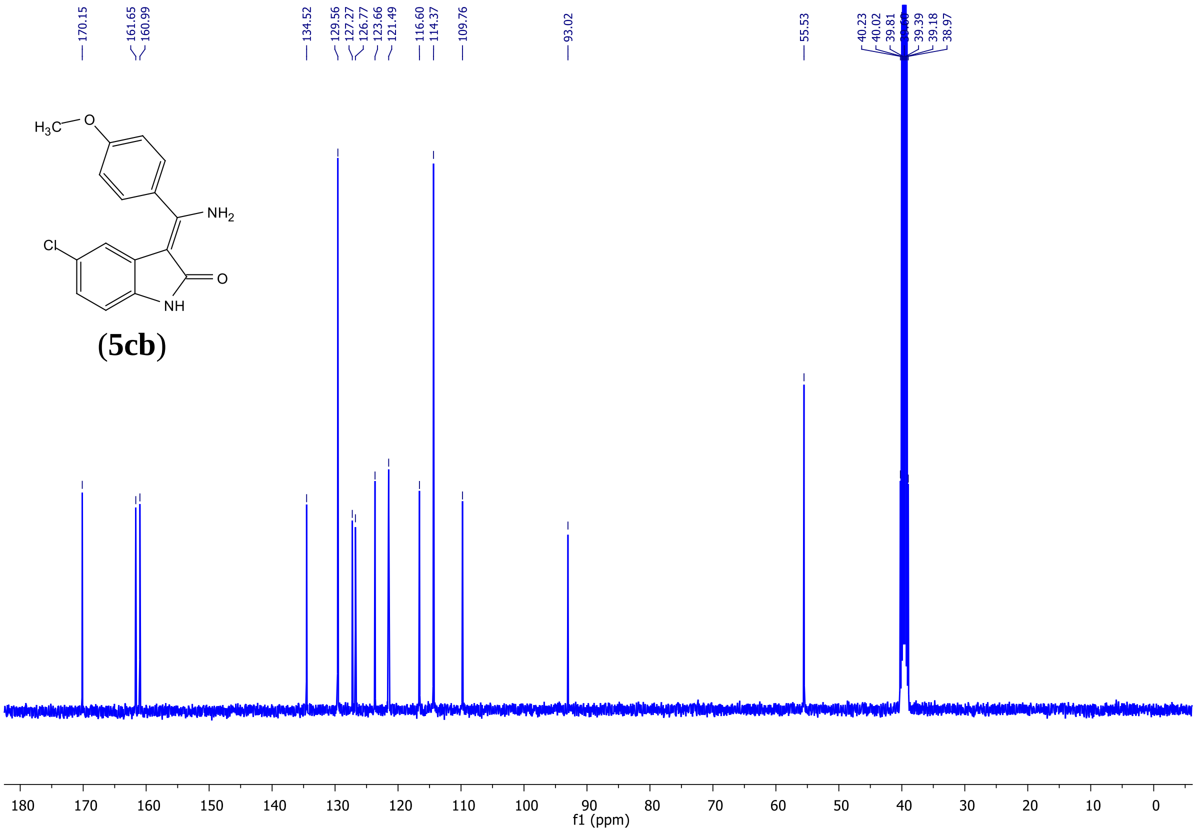
170.15
161.65
160.99
134.52
129.56
127.27
126.77
123.66
121.49
116.60
114.37
109.76
93.02
55.53
40.23
40.02
39.81
39.39
39.18
38.97
H3C
O
NH2
Cl
O
NH
(5cb)
180 170 160 150 140 130 120 110 100 90 80 70 60 50 40 30 20 10 0
f1 (ppm)

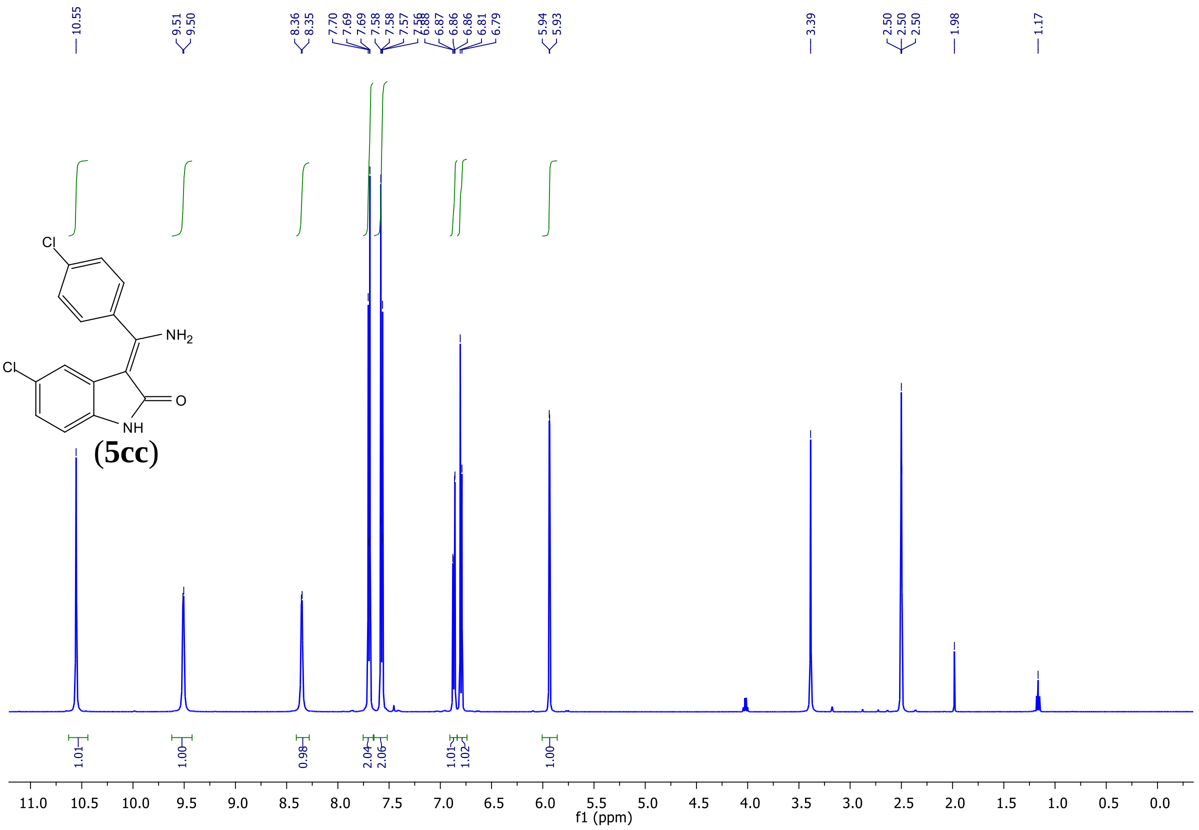
10.55
9.51
9.50
8.36
8.35
7.70
7.69
7.69
7.58
7.58
7.57
7.56
6.88
6.87
6.86
6.86
6.81
6.79
5.94
5.93
3.39
2.50
2.50
2.50
1.98
1.17
Cl
NH2
Cl
O
NH
(5cc)
1.01
1.00
0.98
2.04
2.06
1.01
1.02
1.00
f1 (ppm)

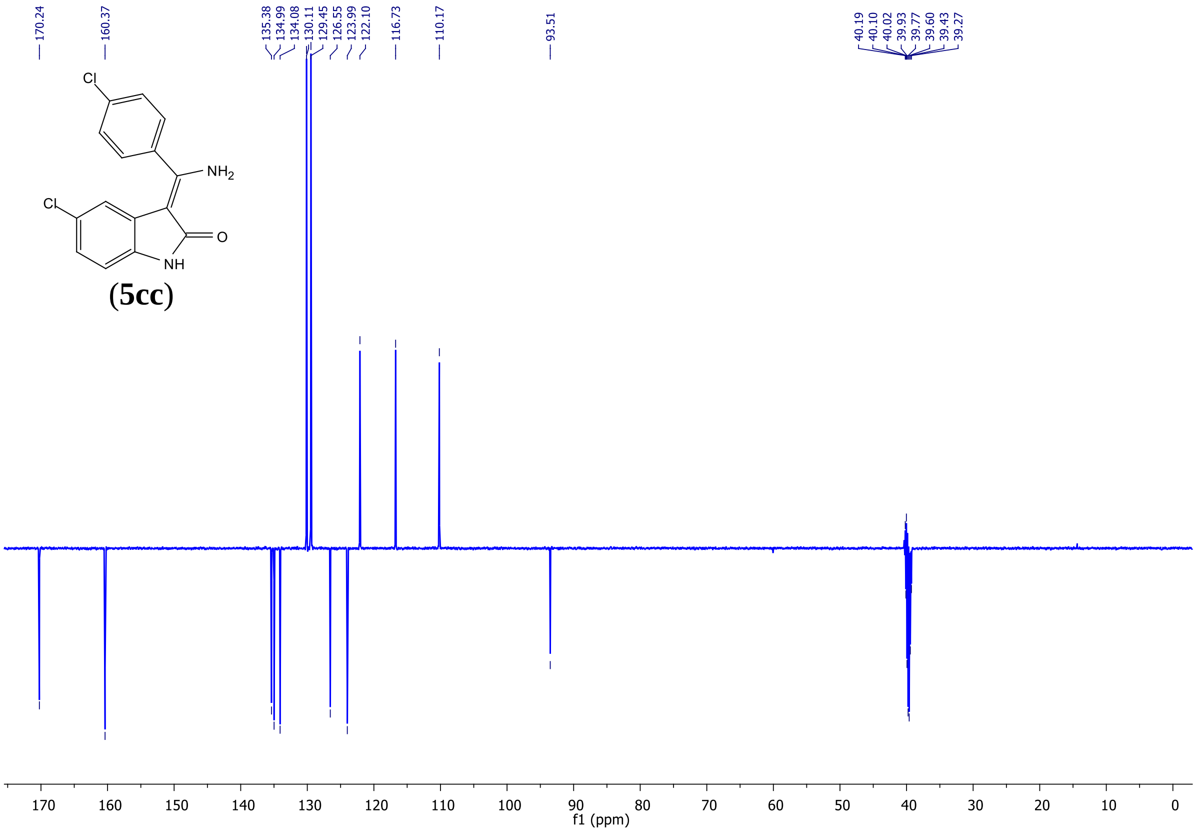
170.24
160.37
135.38
134.99
134.08
130.11
129.45
126.55
123.99
122.10
116.73
110.17
93.51
40.19
40.10
40.02
39.93
39.77
39.60
39.43
39.27
Cl
NH2
Cl
O
NH
(5cc)
f1 (ppm)

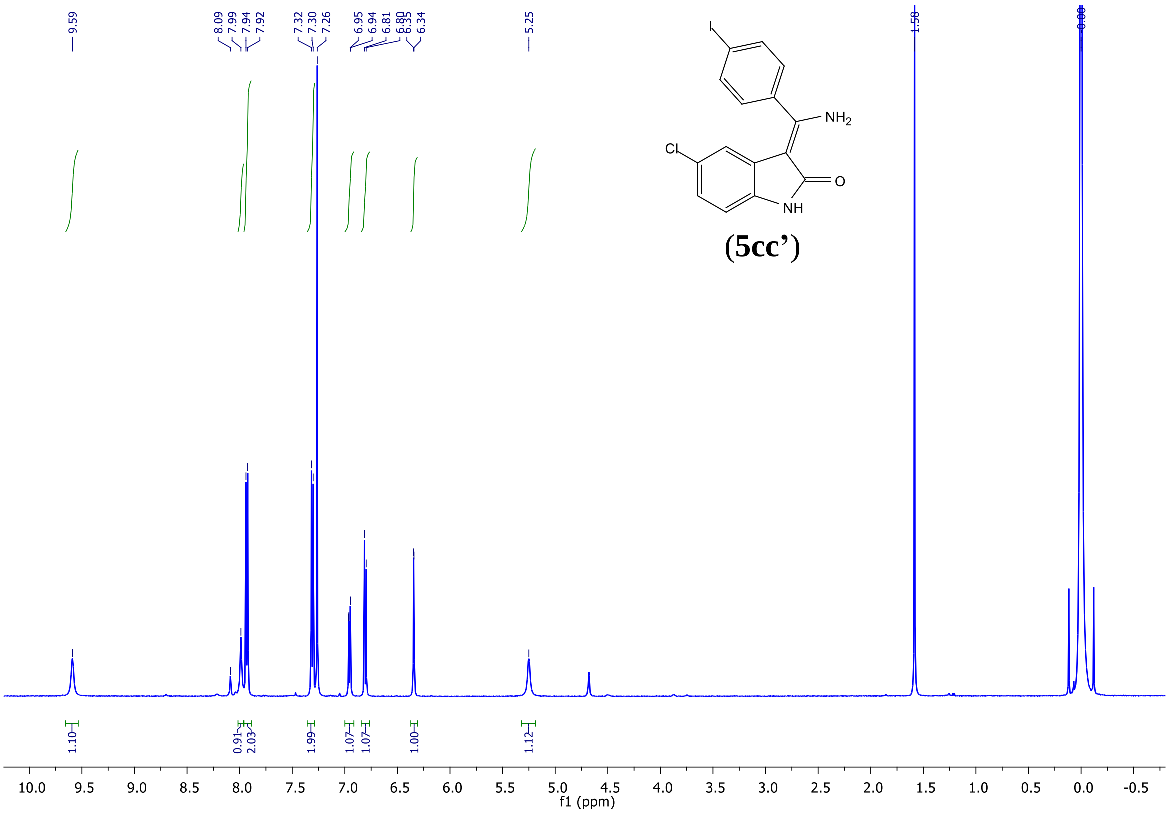
I
NH2
Cl
O
NH
(5cc')
f1 (ppm)

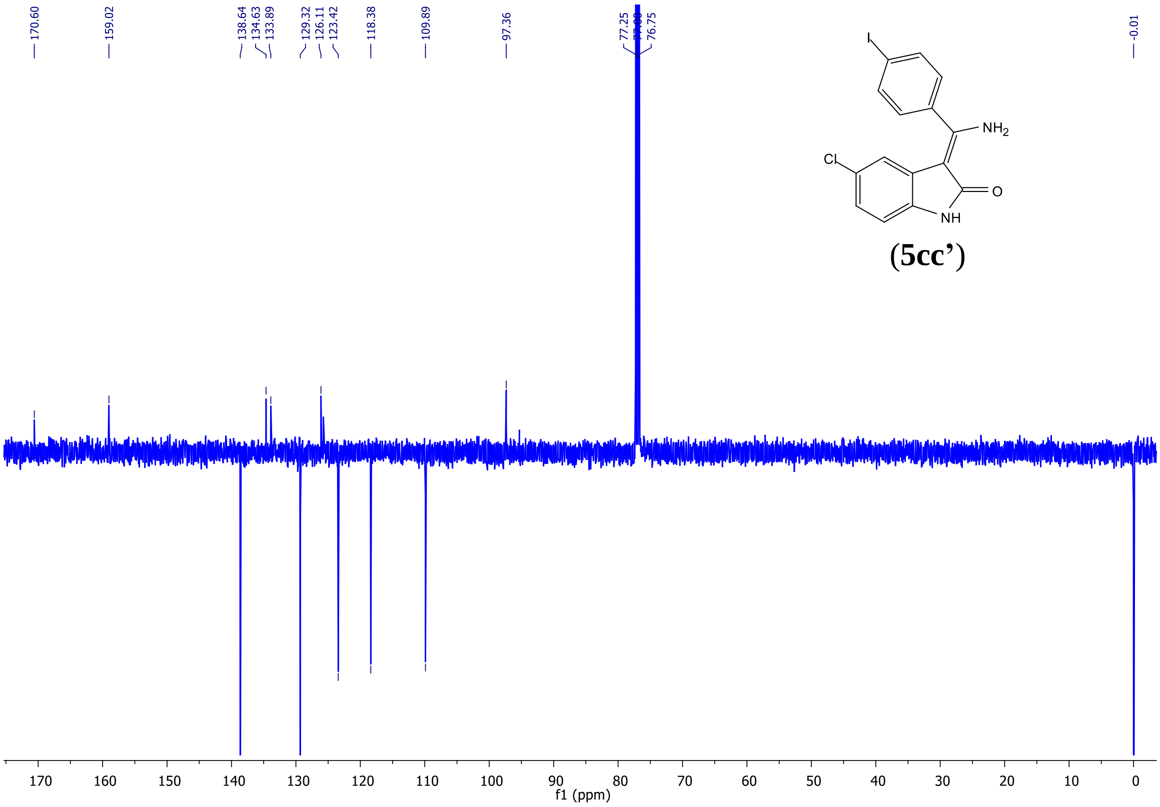
I
NH2
Cl
O
NH
(5cc')
f1 (ppm)

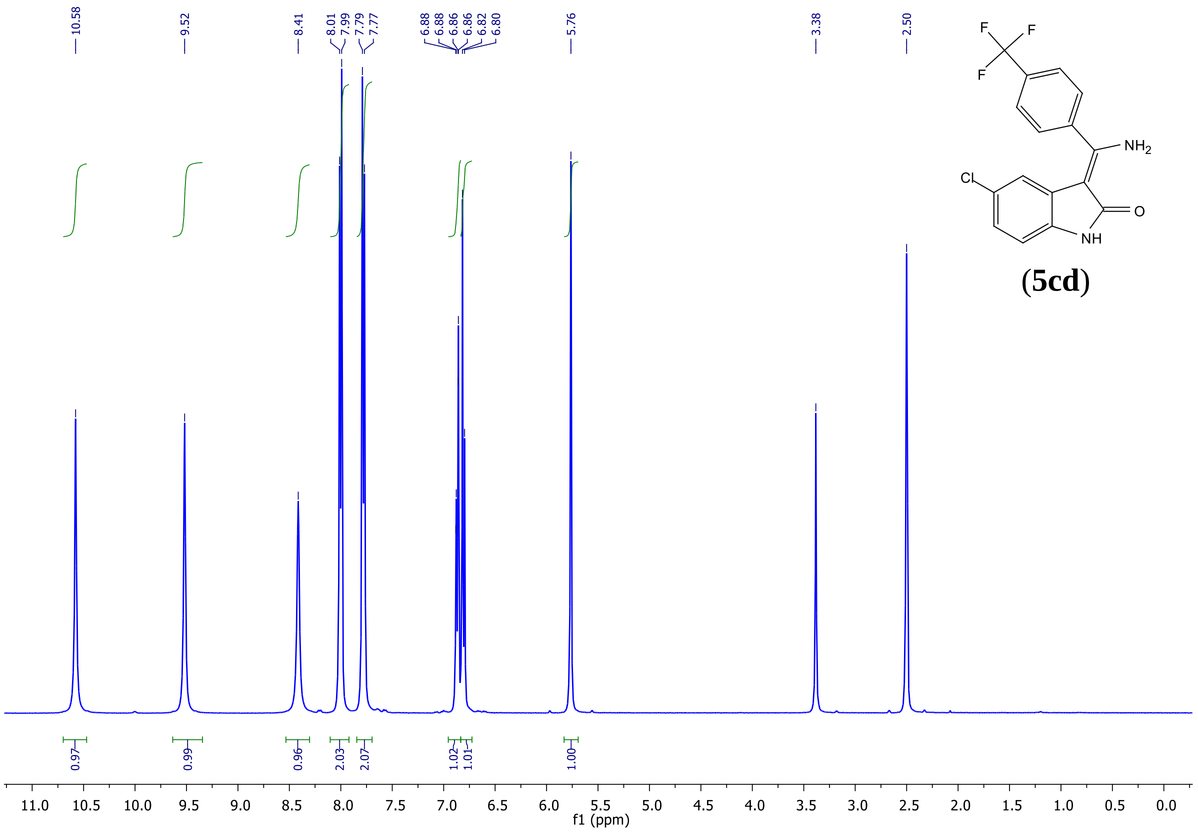
(5cd)
f1 (ppm)

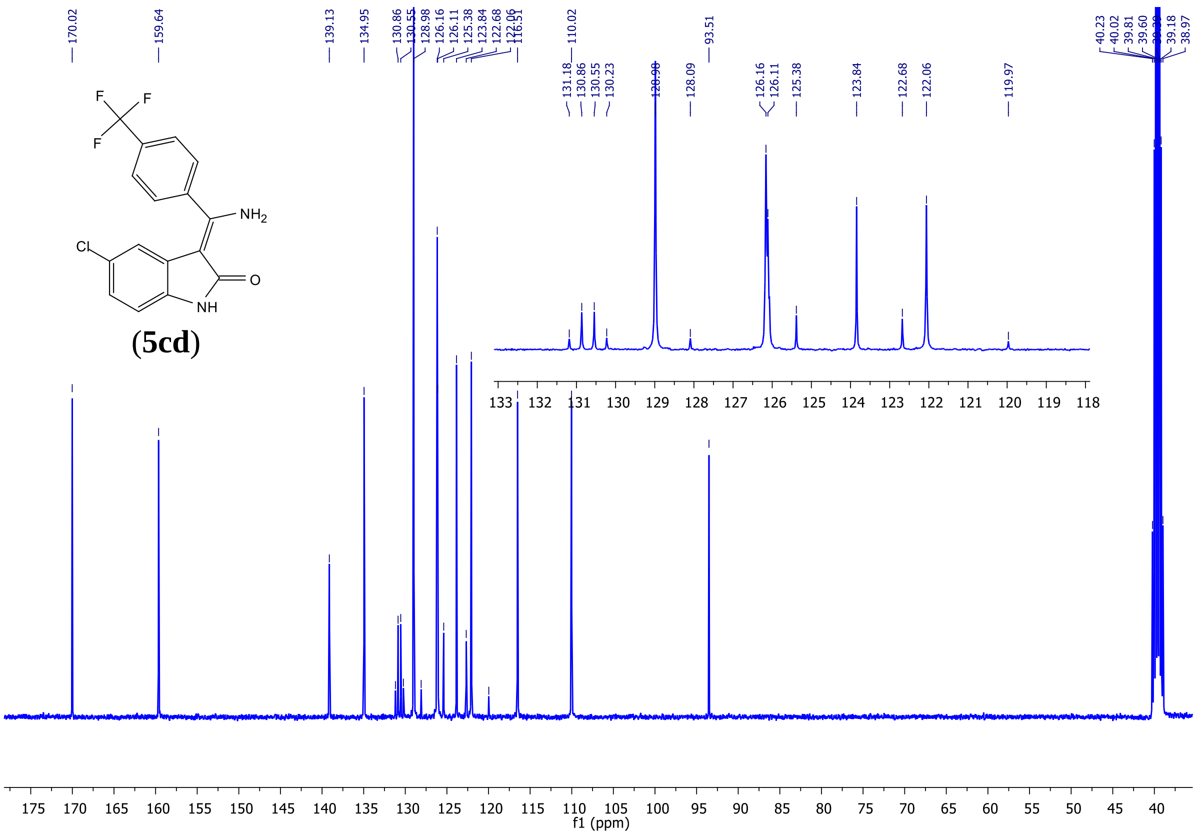
(5cd)
f1 (ppm)

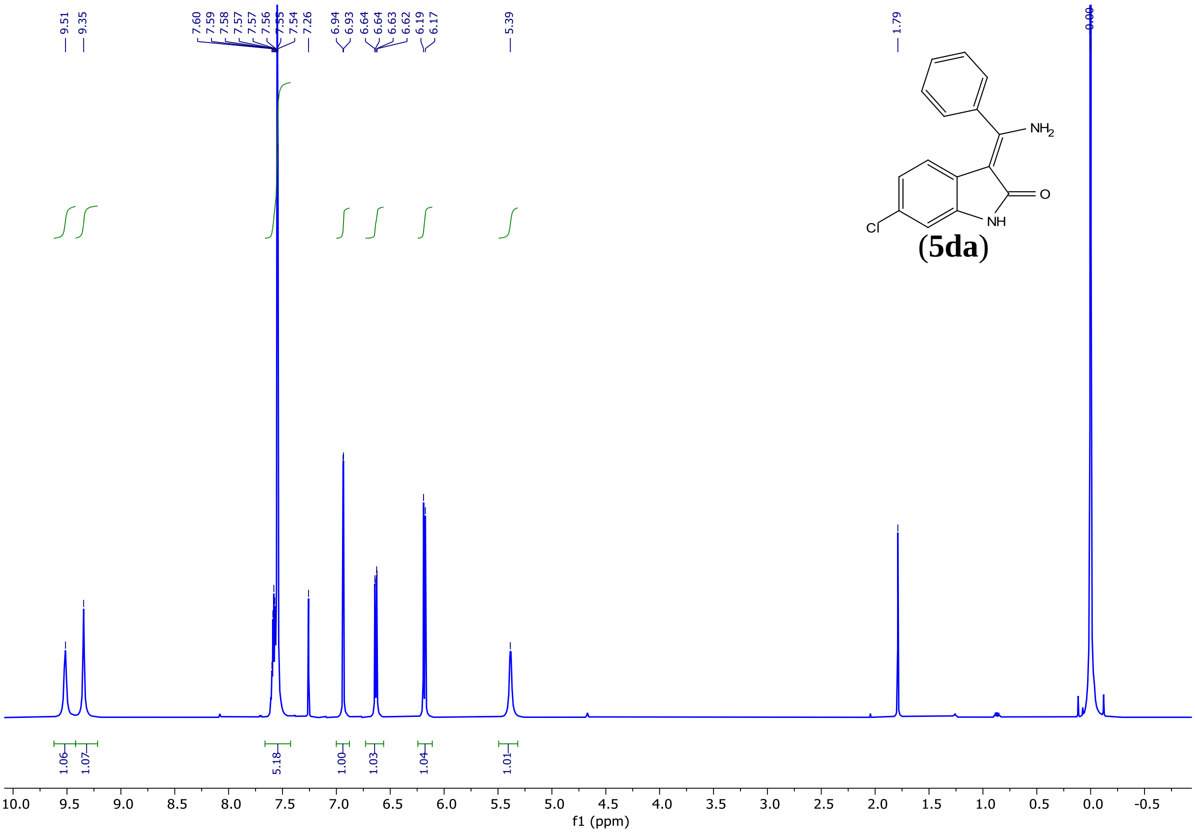

(5da)

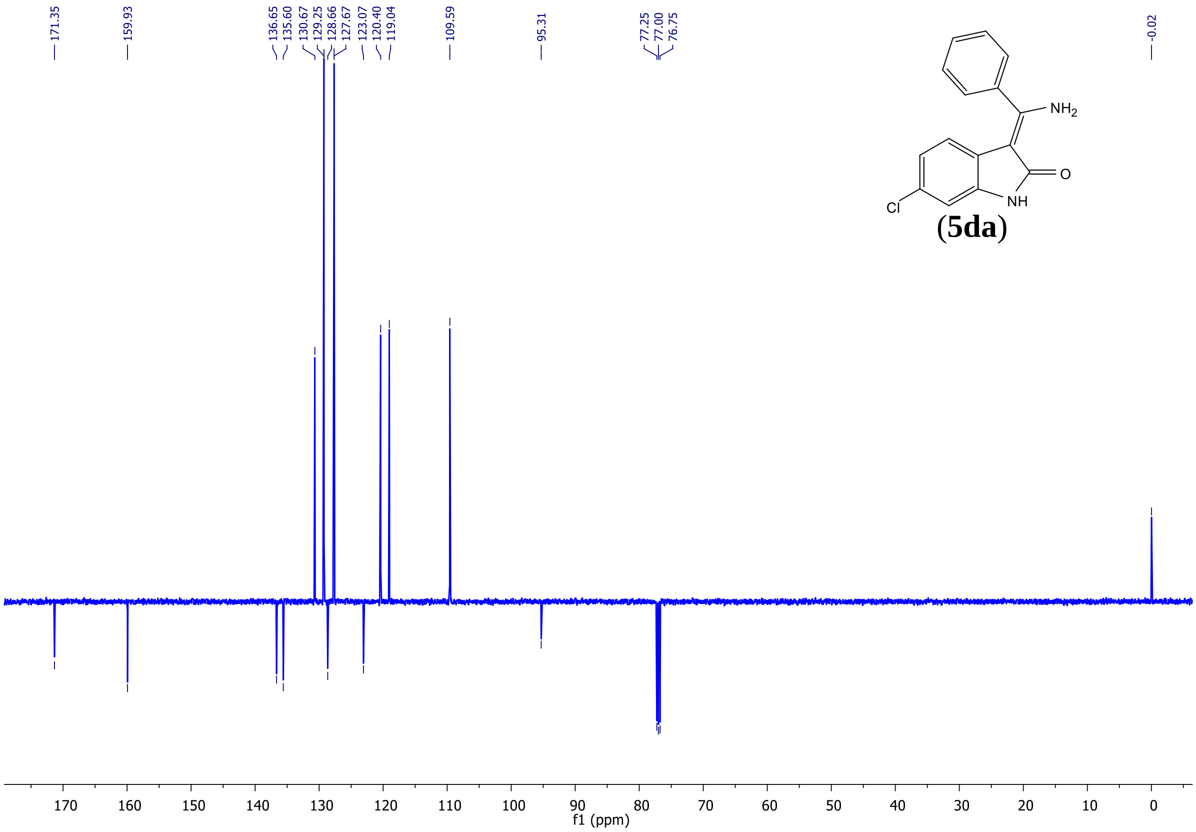

(5da)

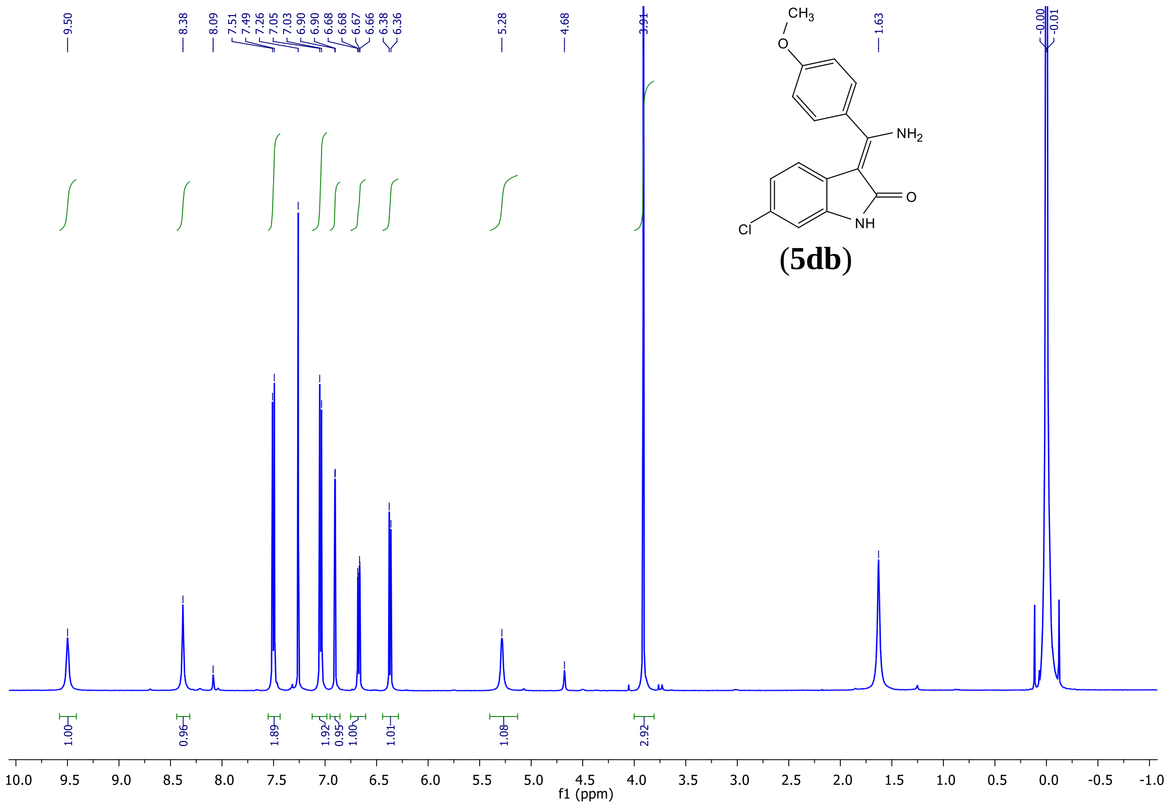
9.50
8.38
8.09
7.51
7.49
7.26
7.05
7.03
6.90
6.90
6.68
6.68
6.67
6.66
6.38
6.36
5.28
4.68
3.91
1.63
0.00
-0.01
CH3
O
NH2
O
Cl
NH
1.00
0.96
1.89
1.92
0.95
1.00
1.01
1.08
2.92
f1 (ppm)

(5db)

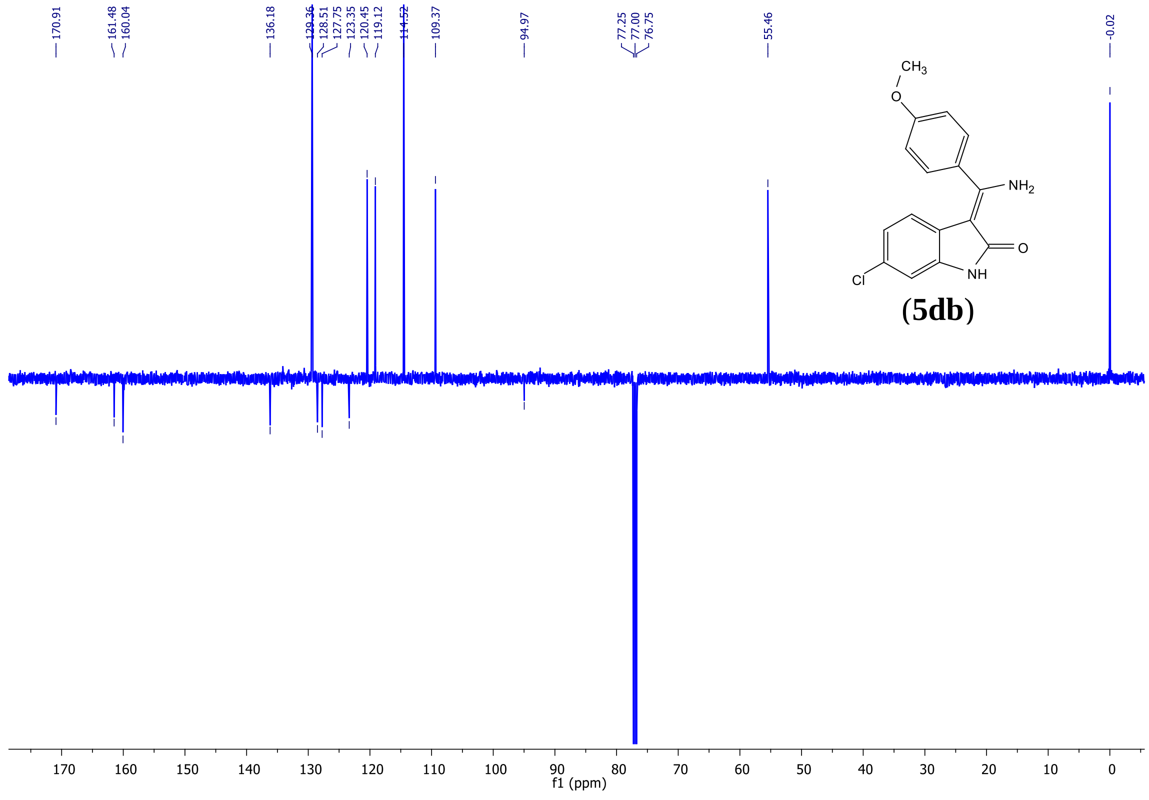
170.91
161.48
160.04
136.18
129.36
128.51
127.75
123.35
120.45
119.12
114.52
109.37
94.97
77.25
77.00
76.75
55.46
-0.02
CH3
O
NH2
O
Cl
NH
f1 (ppm)

(5db)

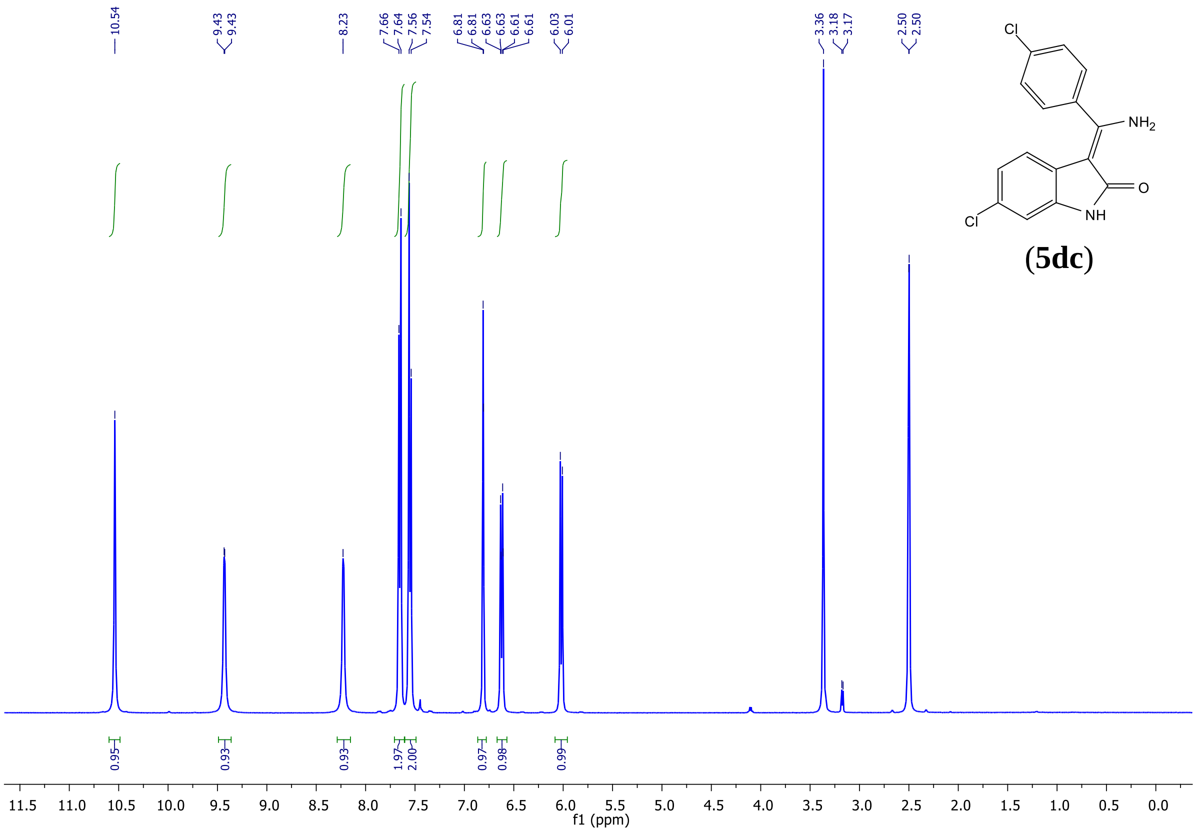
10.54
9.43
9.43
8.23
7.66
7.64
7.56
7.54
6.81
6.81
6.63
6.63
6.61
6.61
6.03
6.01
3.36
3.18
3.17
2.50
2.50
Cl
NH2
O
Cl
NH
(5dc)
0.95
0.93
0.93
1.97
2.00
0.97
0.98
0.99
f1 (ppm)

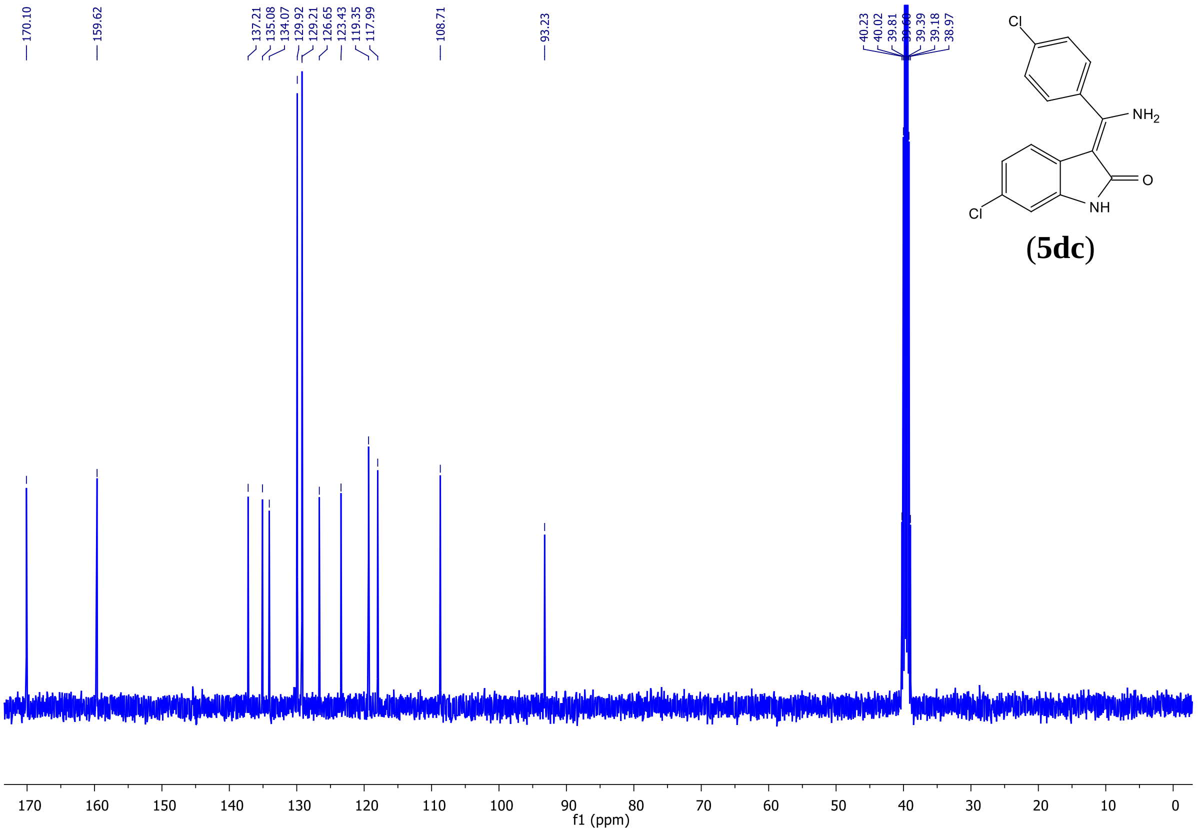
170.10
159.62
137.21
135.08
134.07
129.92
129.21
126.65
123.43
119.35
117.99
108.71
93.23
40.23
40.02
39.81
39.39
39.18
38.97
Cl
NH2
O
Cl
NH
(5dc)
f1 (ppm)

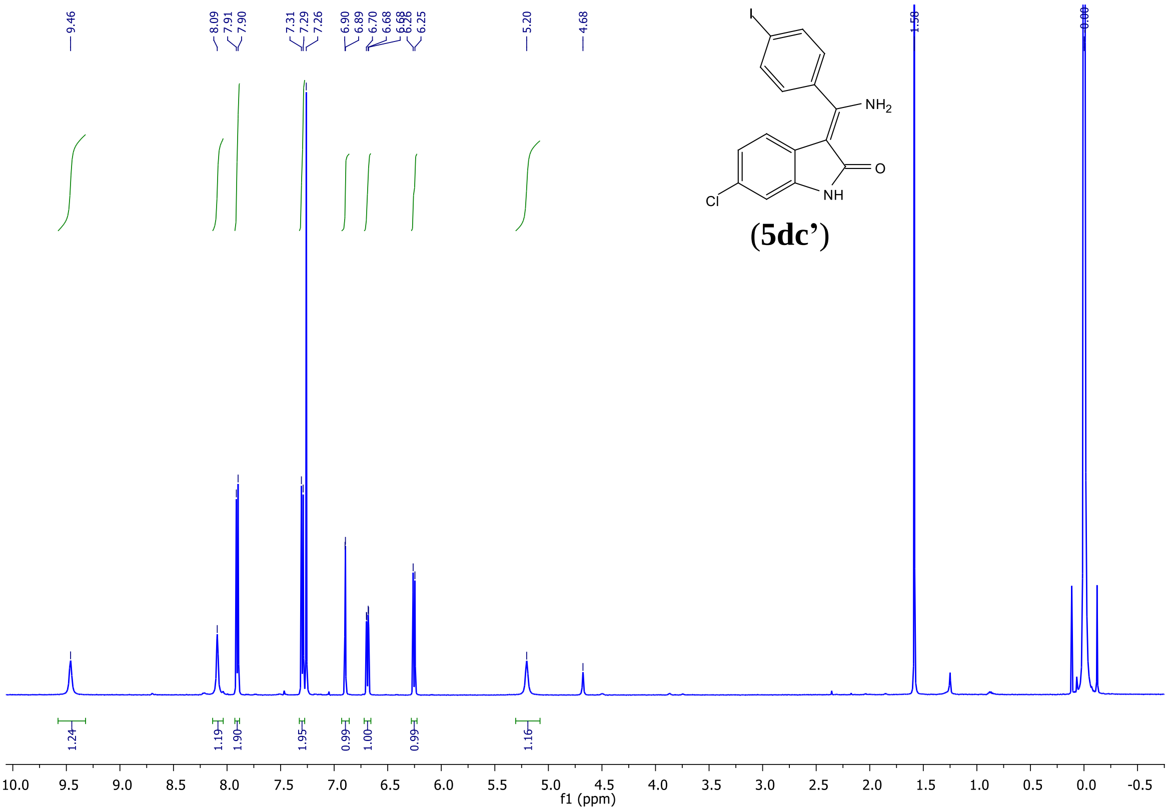
9.46
8.09
7.91
7.90
7.31
7.29
7.26
6.90
6.89
6.70
6.68
6.25
5.20
4.68
1.58
0.00
NH2
O
NH
Cl
I
(5dc')
1.24
1.19
1.90
1.95
0.99
1.00
0.99
1.16
10.0 9.5 9.0 8.5 8.0 7.5 7.0 6.5 6.0 5.5 5.0 4.5 4.0 3.5 3.0 2.5 2.0 1.5 1.0 0.5 0.0 -0.5
f1 (ppm)

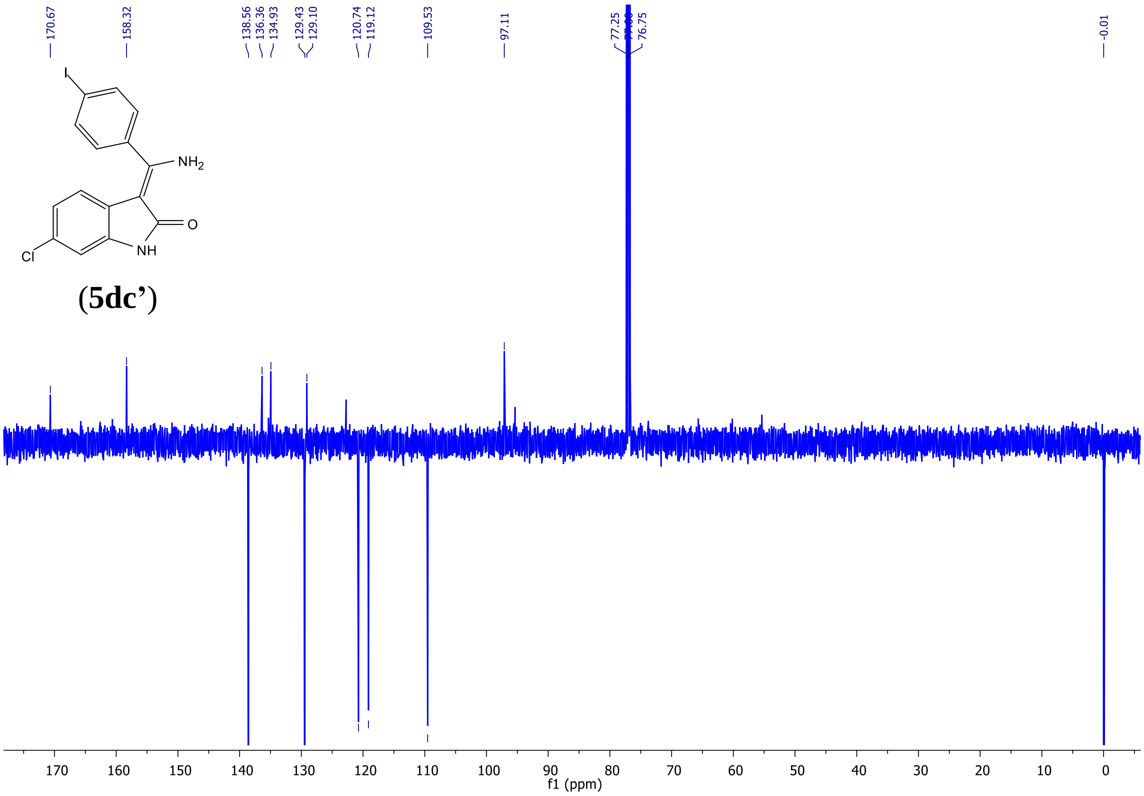
170.67
158.32
138.56
136.36
134.93
129.43
129.10
120.74
119.12
109.53
97.11
77.25
76.75
-0.01
NH2
O
NH
Cl
I
(5dc')
170 160 150 140 130 120 110 100 90 80 70 60 50 40 30 20 10 0
f1 (ppm)

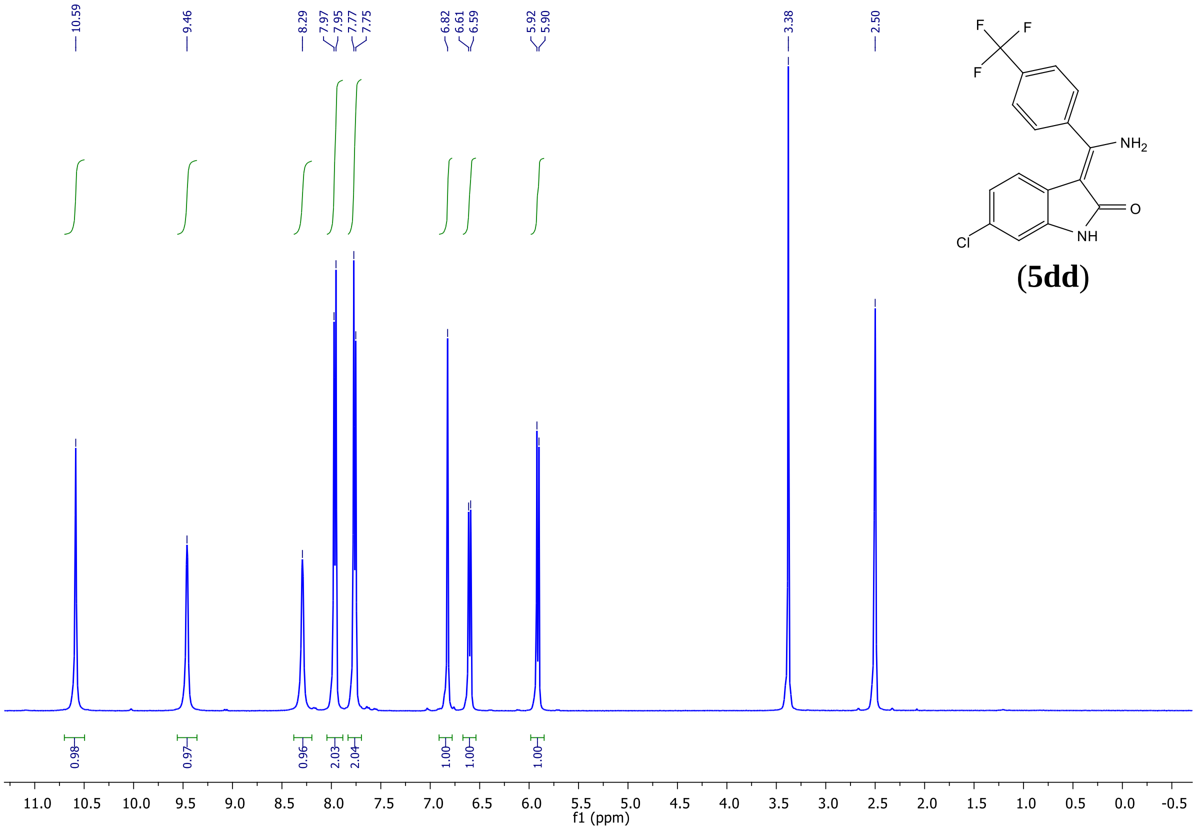
10.59
9.46
8.29
7.97
7.95
7.77
7.75
6.82
6.61
6.59
5.92
5.90
3.38
2.50
0.98
0.97
0.96
2.03
2.04
1.00
1.00
1.00
f1 (ppm)
F
F
F
NH2
O
NH
Cl
(5dd)

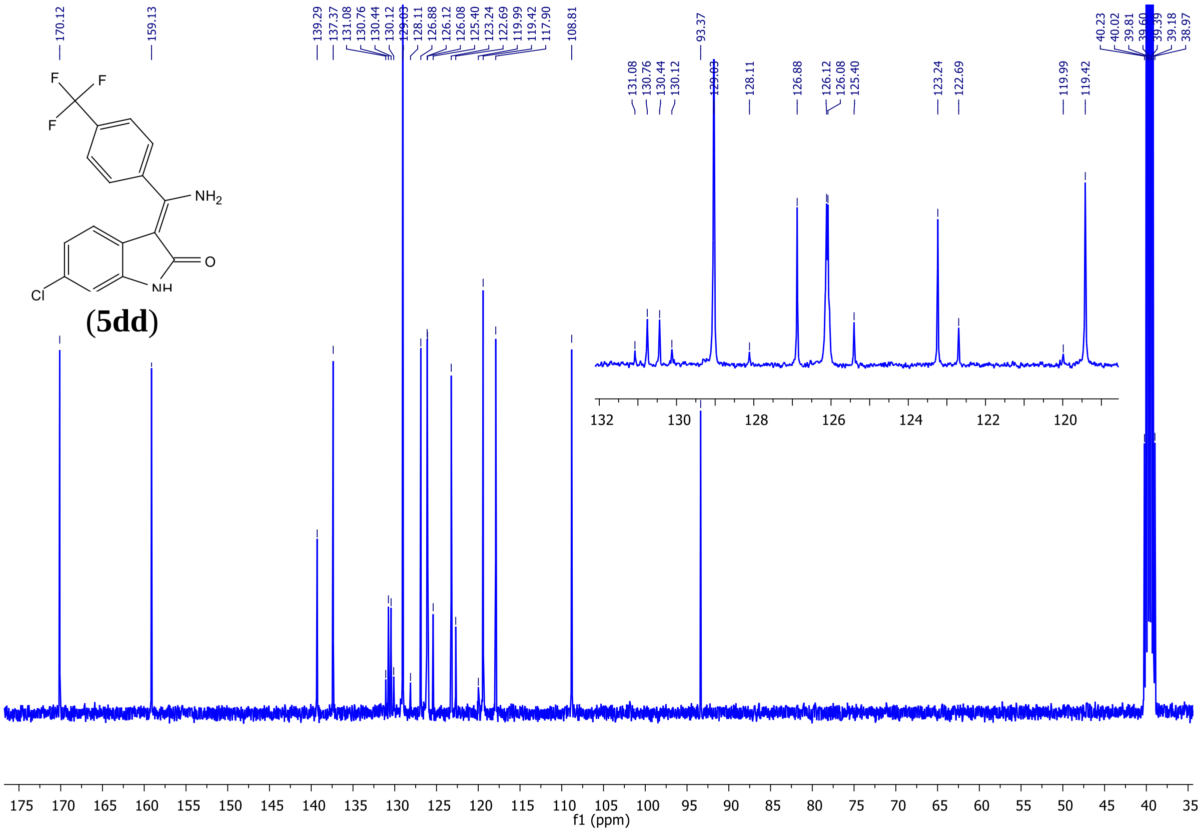
170.12
159.13
139.29
137.37
131.08
130.76
130.44
130.12
129.03
128.11
126.88
126.12
126.08
125.40
123.24
122.69
119.99
119.42
117.90
108.81
93.37
40.23
40.02
39.81
39.60
39.39
39.18
38.97
f1 (ppm)
F
F
F
NH2
O
NH
Cl
(5dd)

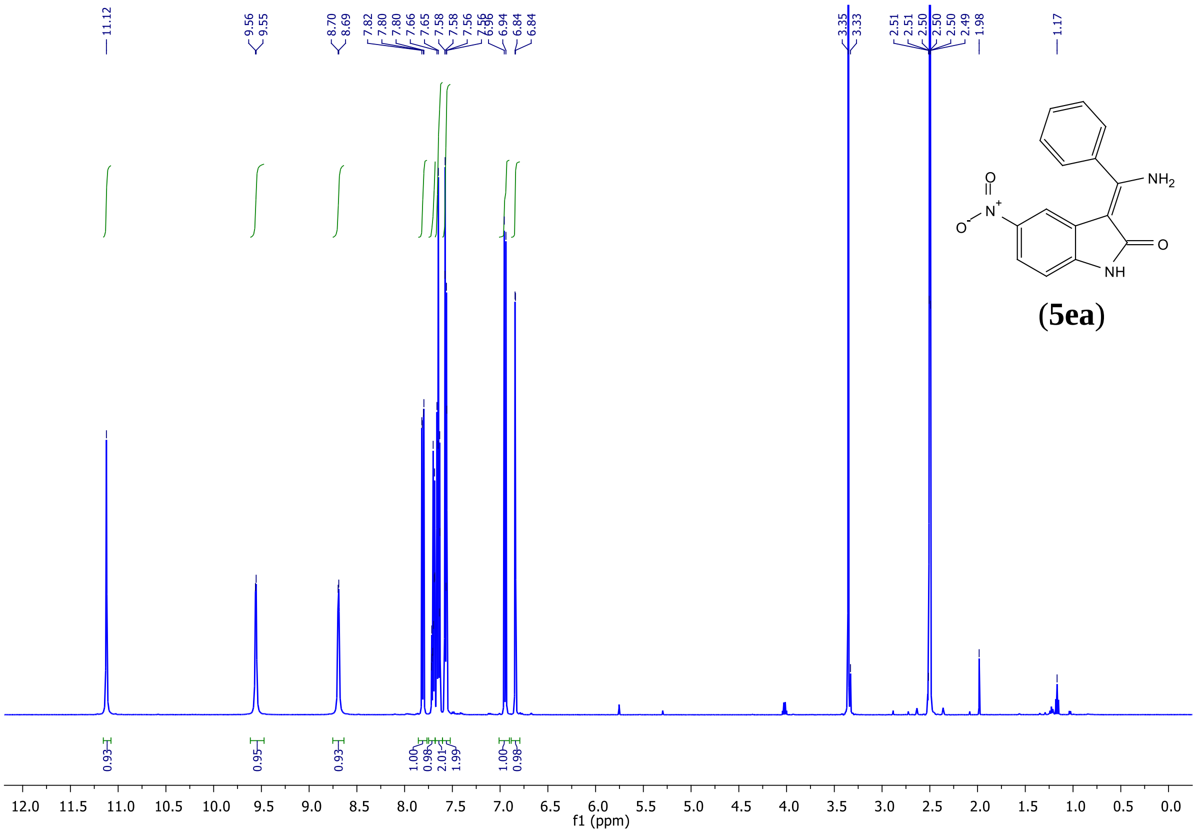

11.12
9.56
9.55
8.70
8.69
7.82
7.80
7.80
7.66
7.65
7.58
7.58
7.56
6.94
6.84
6.84
3.35
3.33
2.51
2.51
2.50
2.50
2.50
2.49
1.98
1.17
NH2
O
N+
O-
NH
O
(5ea)
0.93
0.95
0.93
1.00
0.98
2.01
1.99
1.00
0.98
12.0
11.5
11.0
10.5
10.0
9.5
9.0
8.5
8.0
7.5
7.0
6.5
6.0
5.5
5.0
4.5
4.0
3.5
3.0
2.5
2.0
1.5
1.0
0.5
0.0
f1 (ppm)

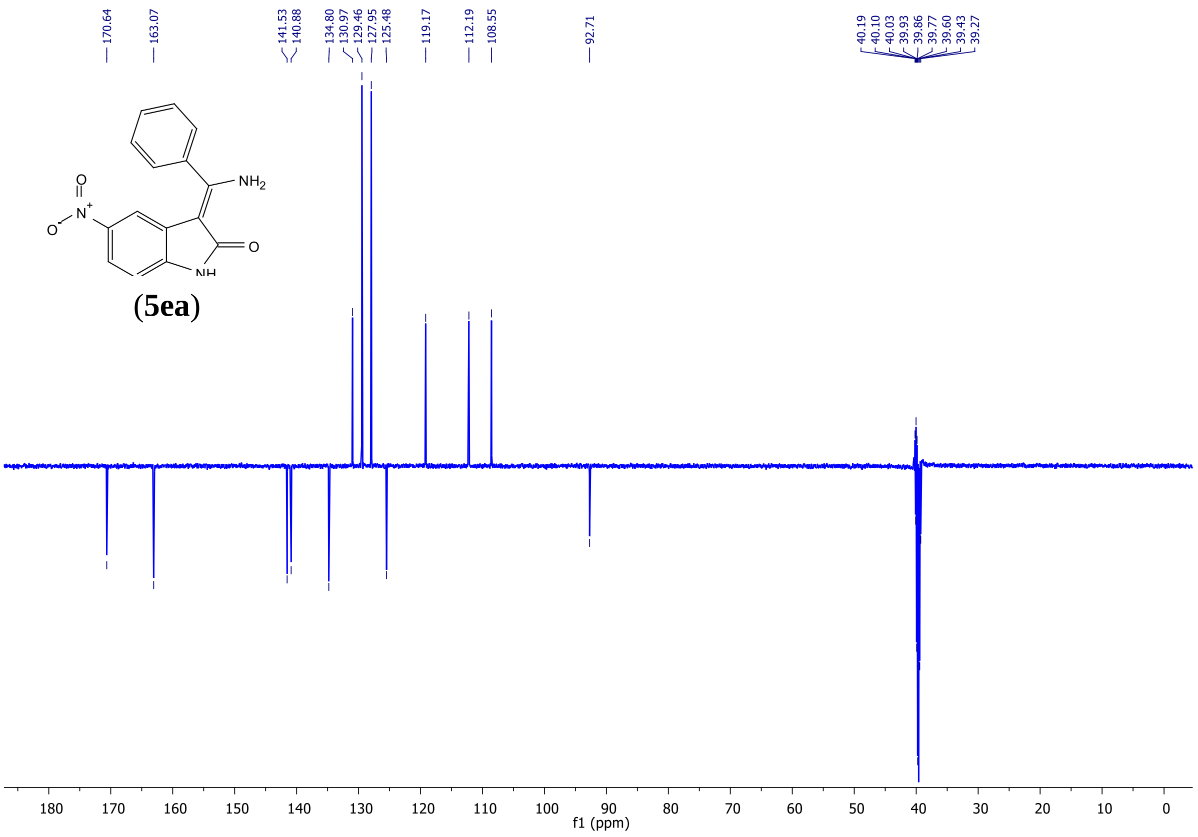

170.64
163.07
141.53
140.88
134.80
130.97
129.46
127.95
125.48
119.17
112.19
108.55
92.71
40.19
40.10
40.03
39.93
39.86
39.77
39.60
39.43
39.27
NH2
O
N+
O-
O
NH
(5ea)
180
170
160
150
140
130
120
110
100
90
80
70
60
50
40
30
20
10
0
f1 (ppm)

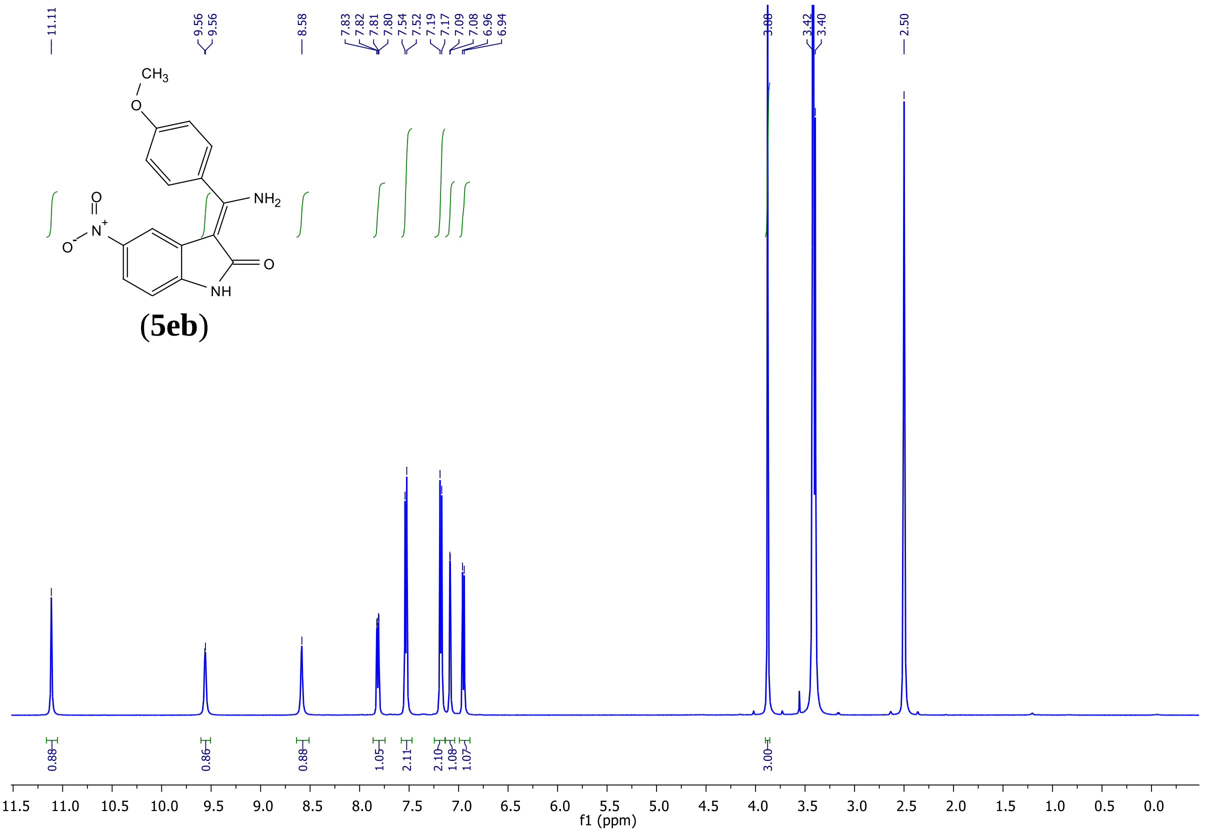
11.11
9.56
9.56
8.58
7.83
7.82
7.81
7.80
7.54
7.52
7.19
7.17
7.09
7.08
6.96
6.94
3.88
3.42
3.40
2.50
CH3
O
NH2
O
N+
O-
O
NH
(5eb)
0.88
0.86
0.88
1.05
2.11
2.10
1.08
1.07
3.00
11.5 11.0 10.5 10.0 9.5 9.0 8.5 8.0 7.5 7.0 6.5 6.0 5.5 5.0 4.5 4.0 3.5 3.0 2.5 2.0 1.5 1.0 0.5 0.0
f1 (ppm)

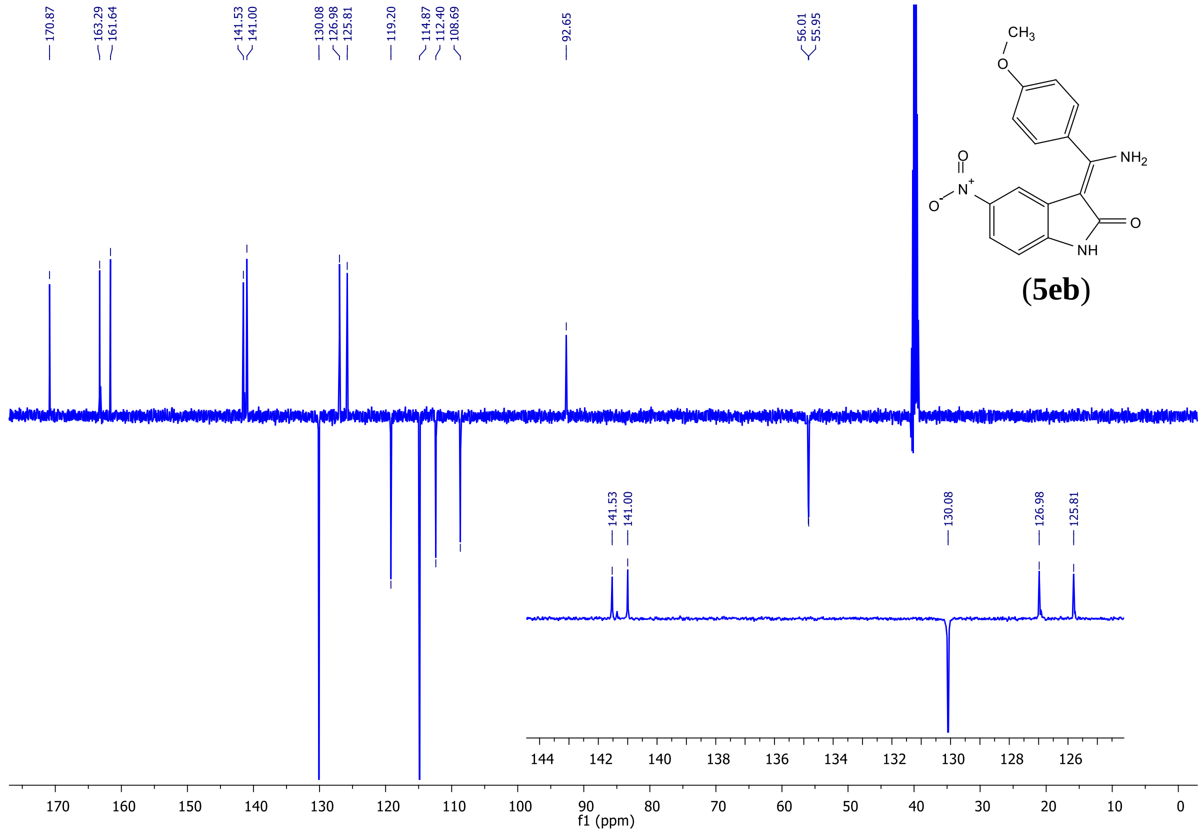
170.87
163.29
161.64
141.53
141.00
130.08
126.98
125.81
119.20
114.87
112.40
108.69
92.65
56.01
55.95
CH3
O
NH2
O
N+
O-
O
NH
(5eb)
141.53
141.00
130.08
126.98
125.81
144 142 140 138 136 134 132 130 128 126
170 160 150 140 130 120 110 100 90 80 70 60 50 40 30 20 10 0
f1 (ppm)

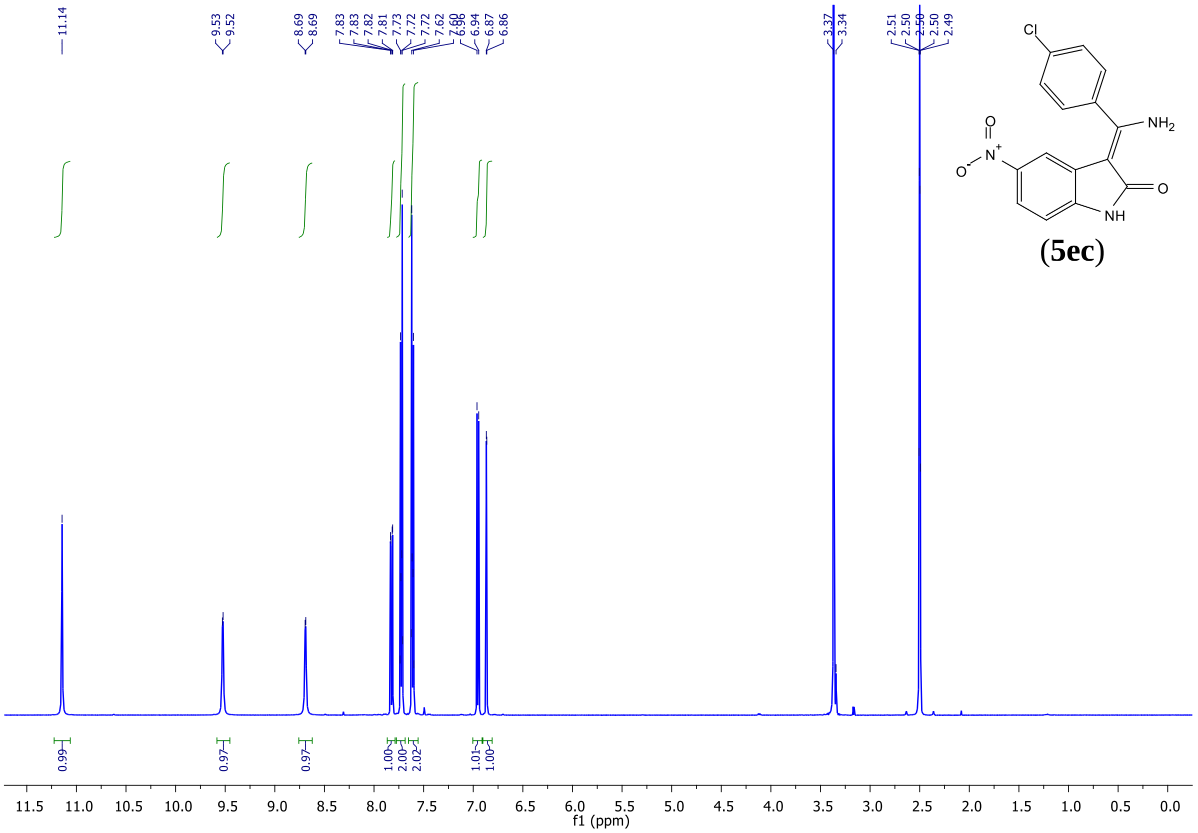

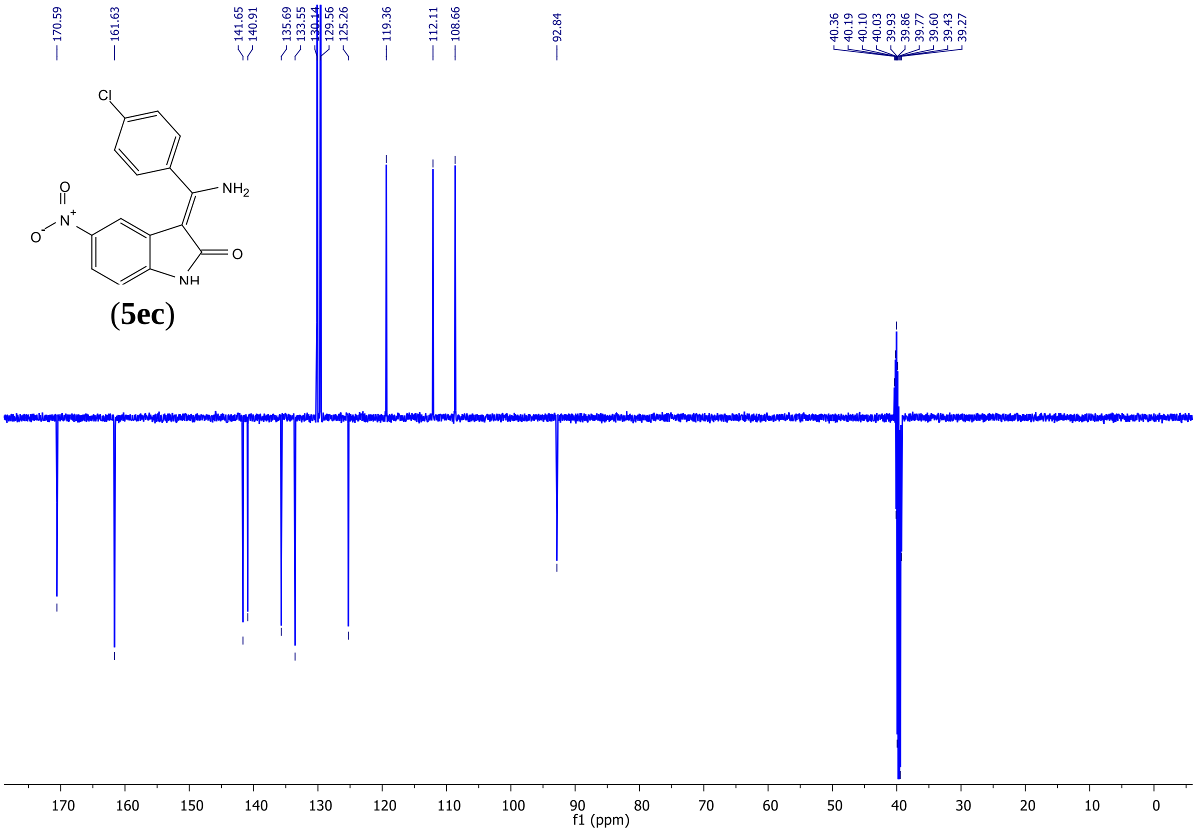

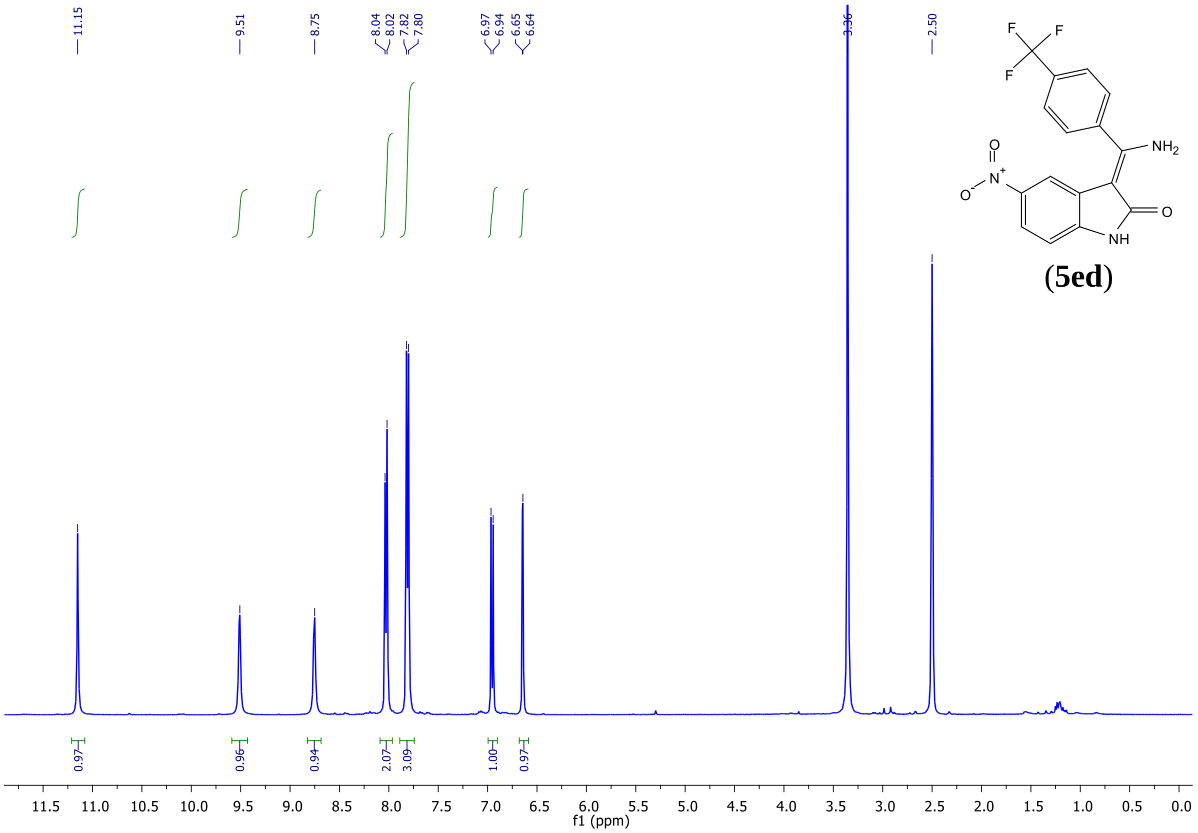
11.15
9.51
8.75
8.04
8.02
7.82
7.80
6.97
6.94
6.65
6.64
3.36
2.50
NH2
NH
O
(5ed)
0.97
0.96
0.94
2.07
3.09
1.00
0.97
f1 (ppm)

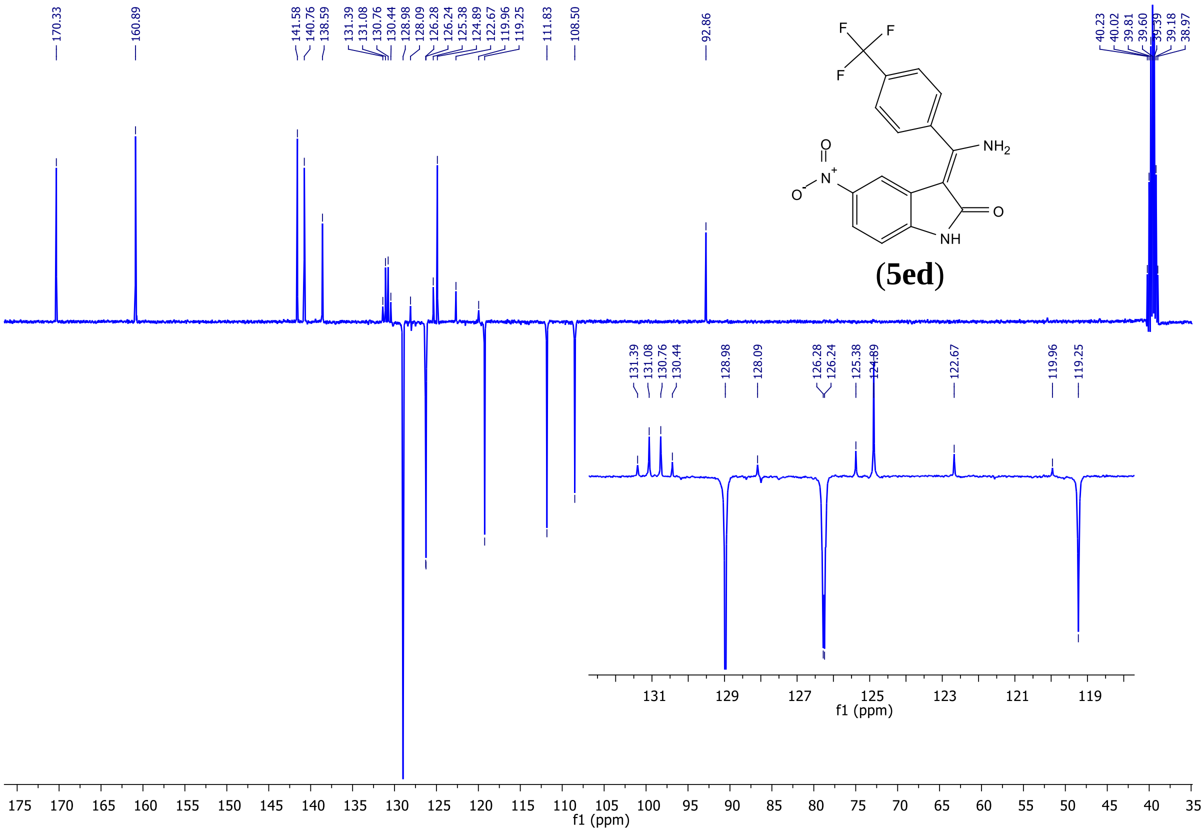
170.33
160.89
141.58
140.76
138.59
131.39
131.08
130.76
130.44
128.98
128.09
126.28
126.24
125.38
124.89
122.67
119.96
119.25
111.83
108.50
92.86
40.23
40.02
39.81
39.60
39.39
39.18
38.97
NH2
NH
O
(5ed)
f1 (ppm)

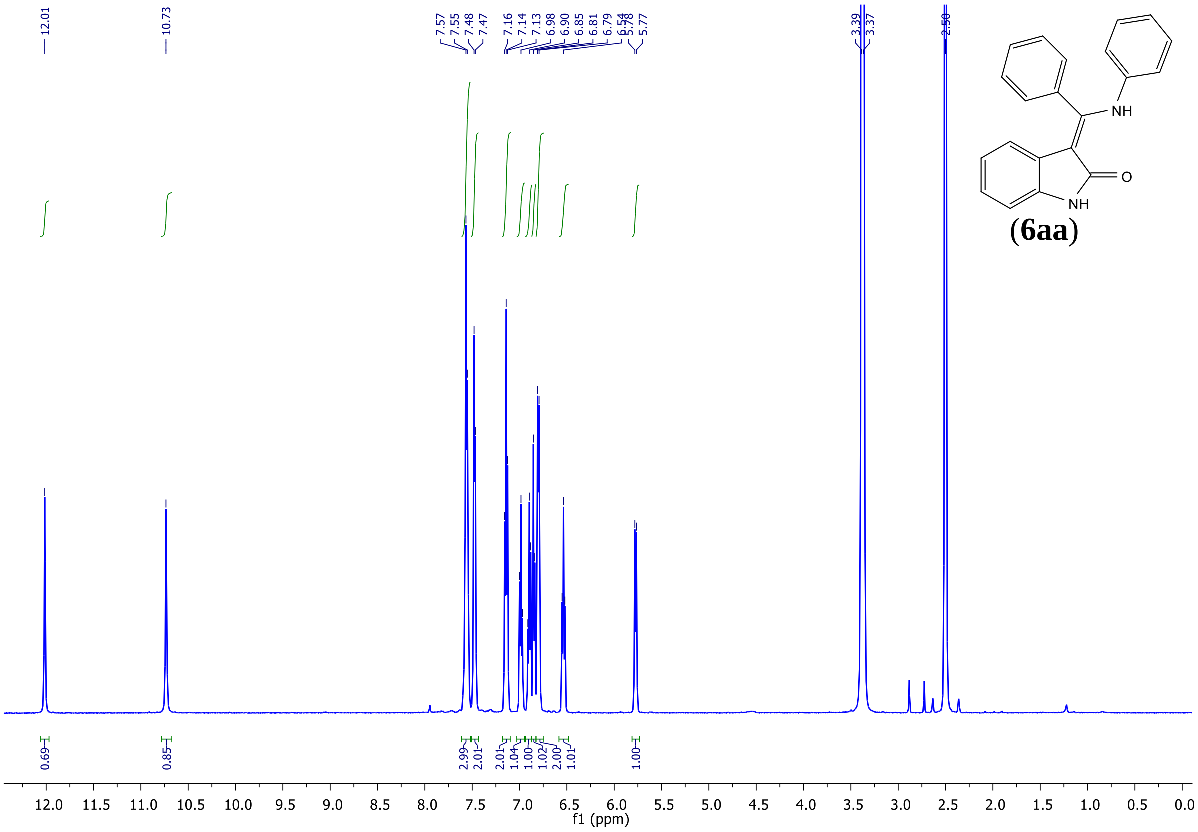
12.01
10.73
7.57
7.55
7.48
7.47
7.16
7.14
7.13
6.98
6.90
6.85
6.81
6.79
6.54
5.78
5.77
3.39
3.37
2.50
NH
O
NH
(6aa)
0.69
0.85
2.99
2.01
2.01
1.04
1.00
1.02
2.00
1.01
1.00
f1 (ppm)

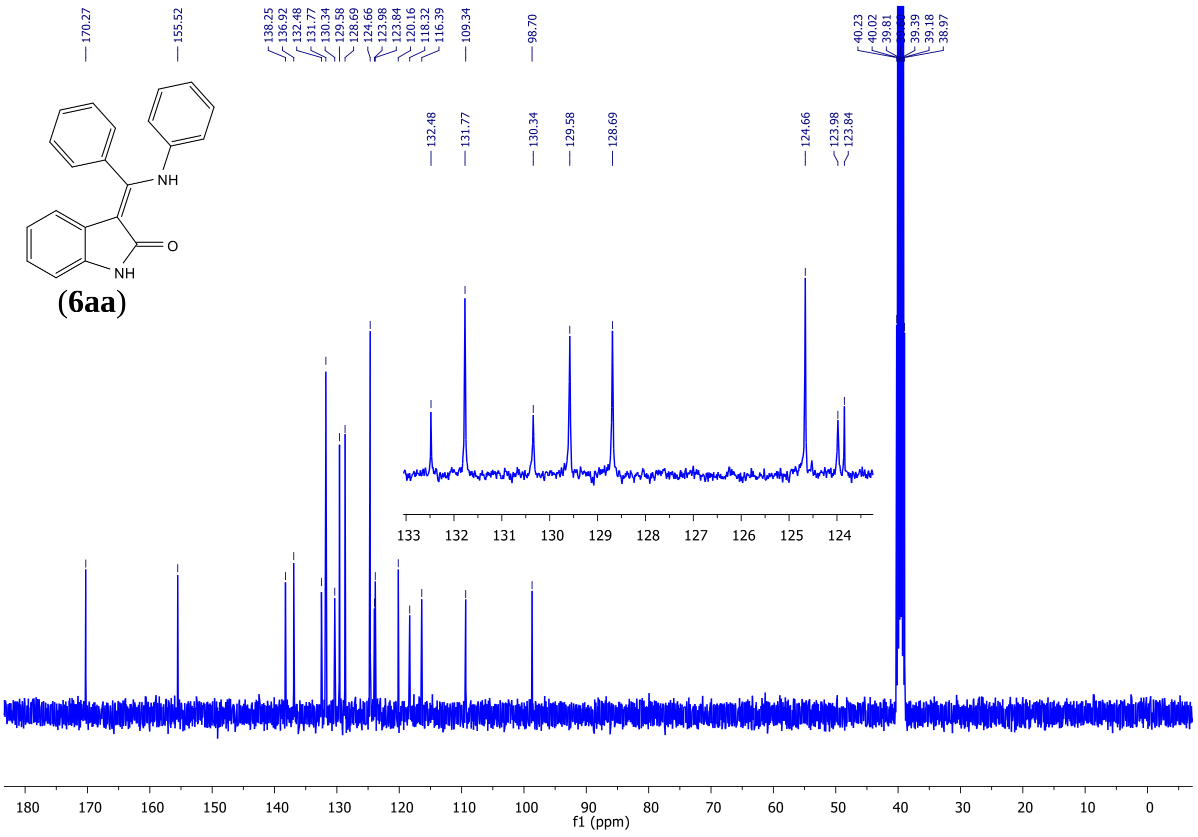
170.27
155.52
138.25
136.92
132.48
131.77
130.34
129.58
128.69
124.66
123.98
123.84
120.16
118.32
116.39
109.34
98.70
40.23
40.02
39.81
39.39
39.18
38.97
NH
O
NH
(6aa)
f1 (ppm)

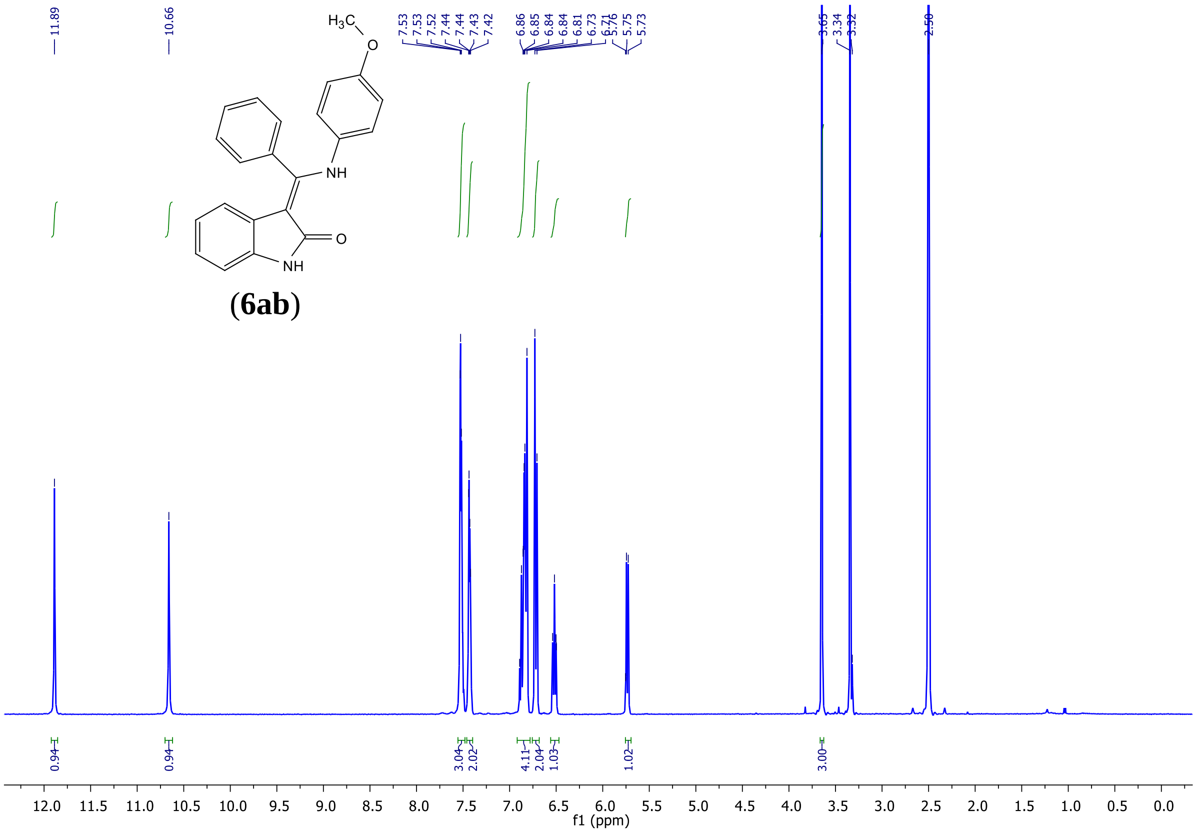
11.89
10.66
7.53
7.53
7.52
7.44
7.43
7.42
6.86
6.85
6.84
6.84
6.81
6.73
6.71
5.76
5.75
5.73
3.65
3.34
3.32
2.50
0.94
0.94
3.04
2.02
4.11
2.04
1.03
1.02
3.00
f1 (ppm)

(6ab)

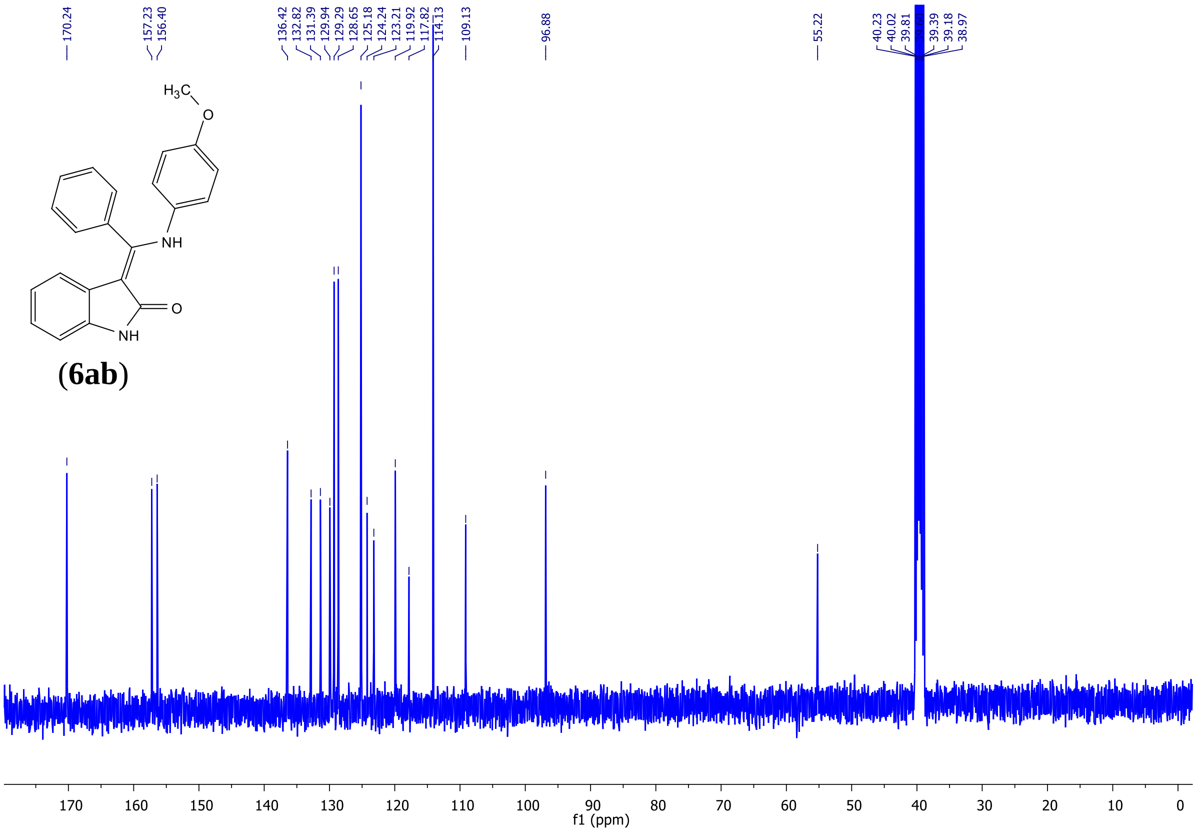
170.24
157.23
156.40
136.42
132.82
131.39
129.94
129.29
128.65
125.18
124.24
123.21
119.92
117.82
114.13
109.13
96.88
55.22
40.23
40.02
39.81
39.39
39.18
38.97
f1 (ppm)

(6ab)

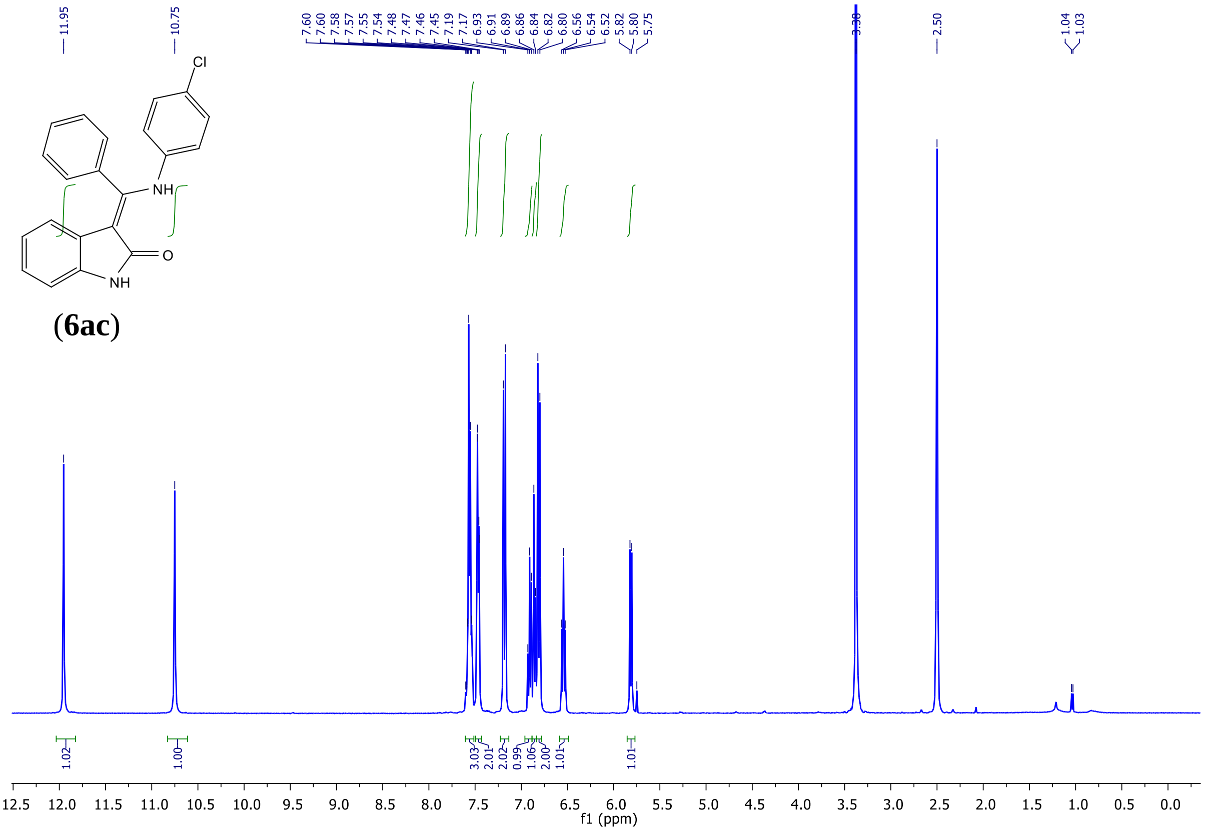
Cl
NH
O
NH
(6ac)
f1 (ppm)

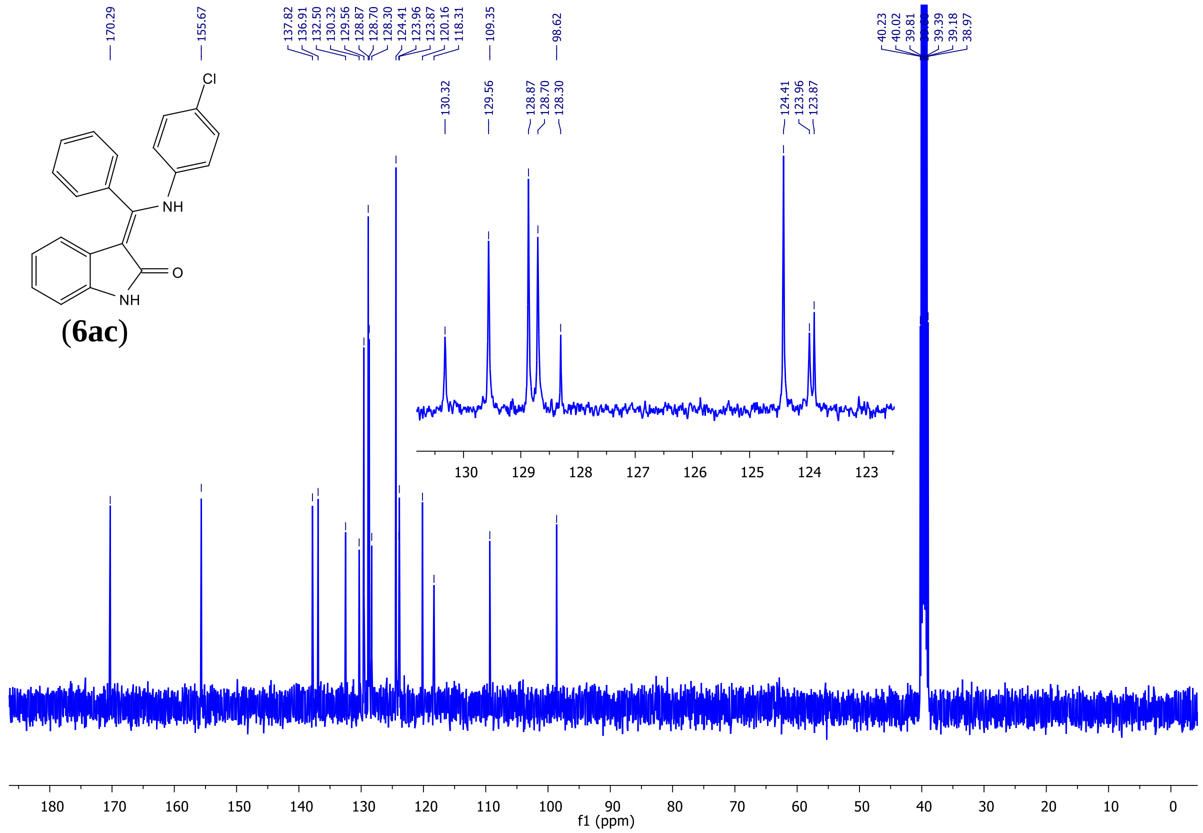
Cl
NH
O
NH
(6ac)
f1 (ppm)

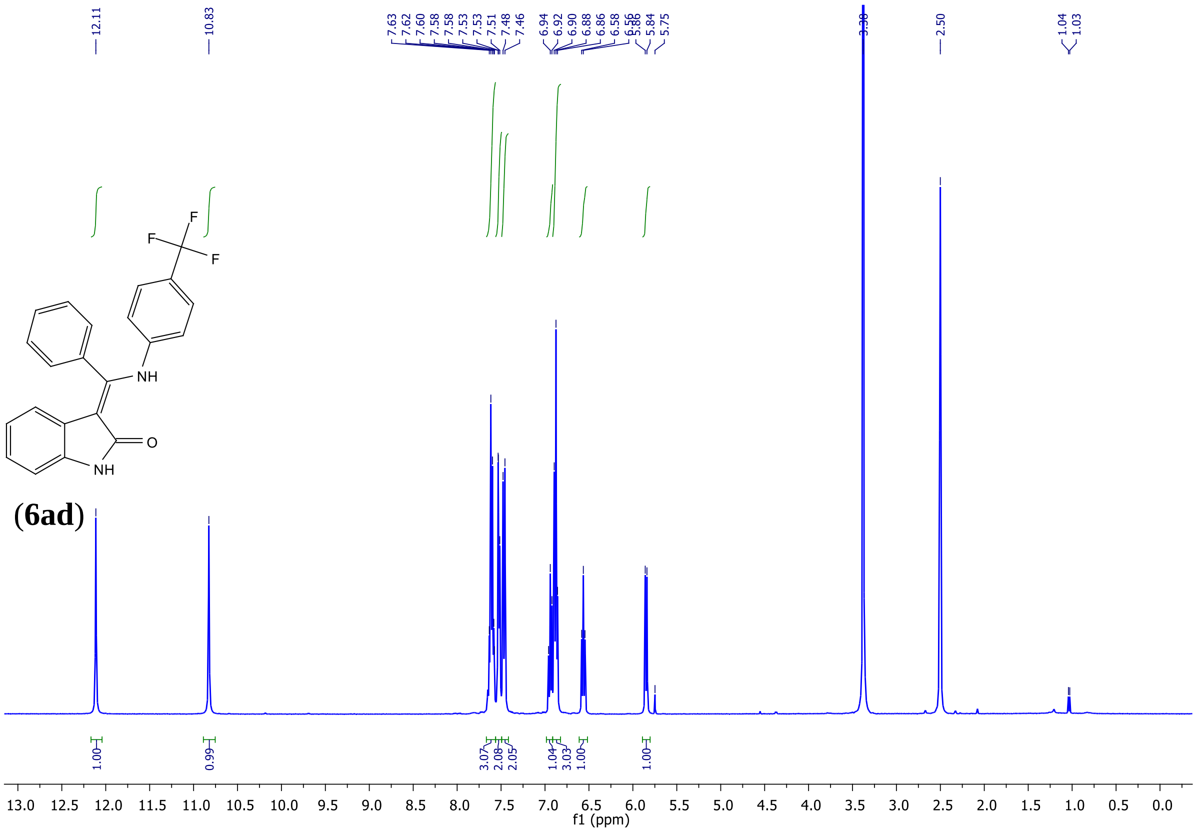

(6ad)

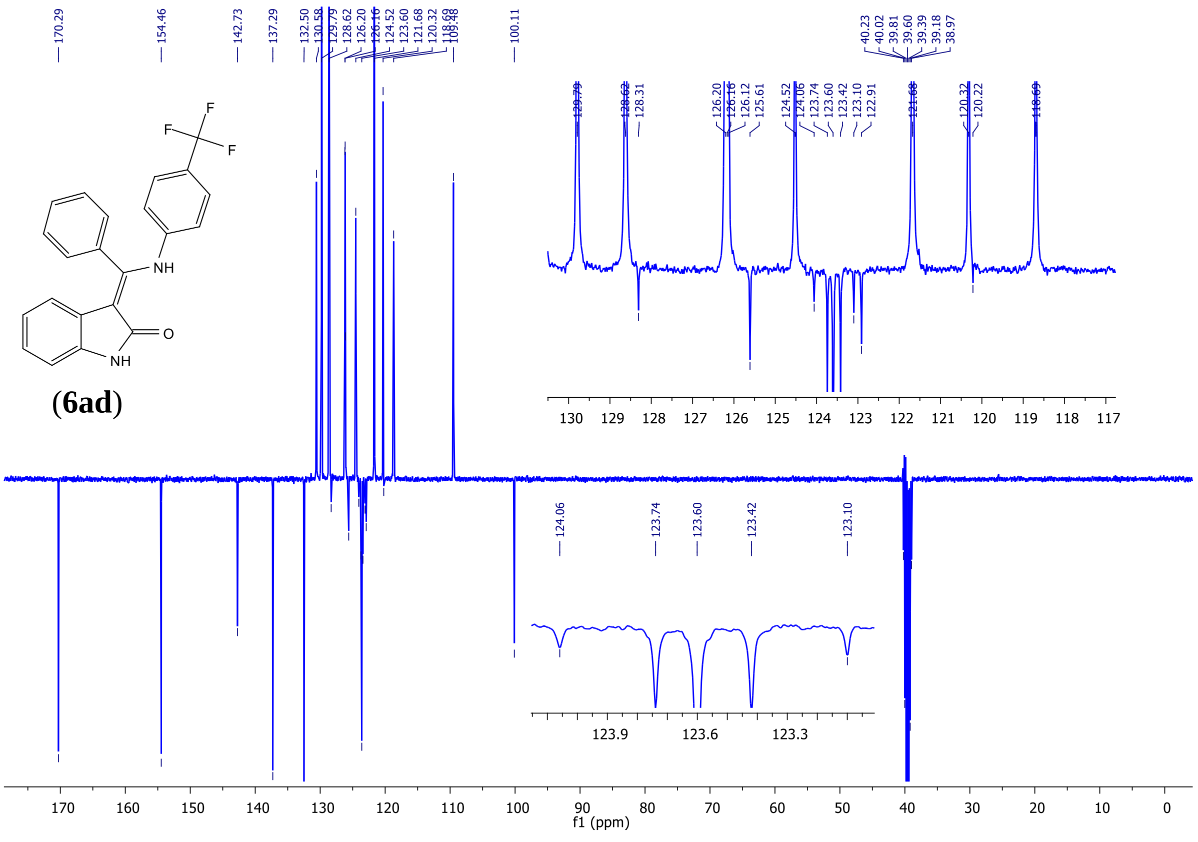

(6ad)

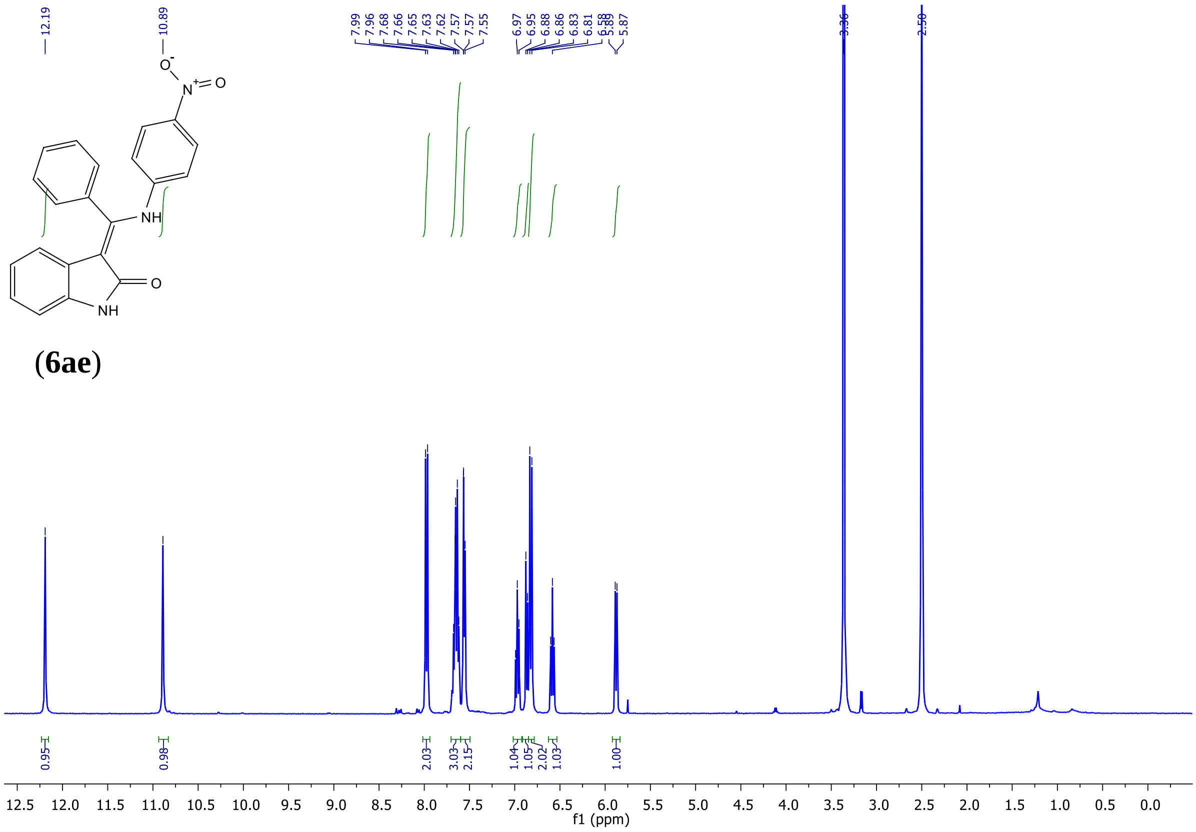
(6ae)
f1 (ppm)

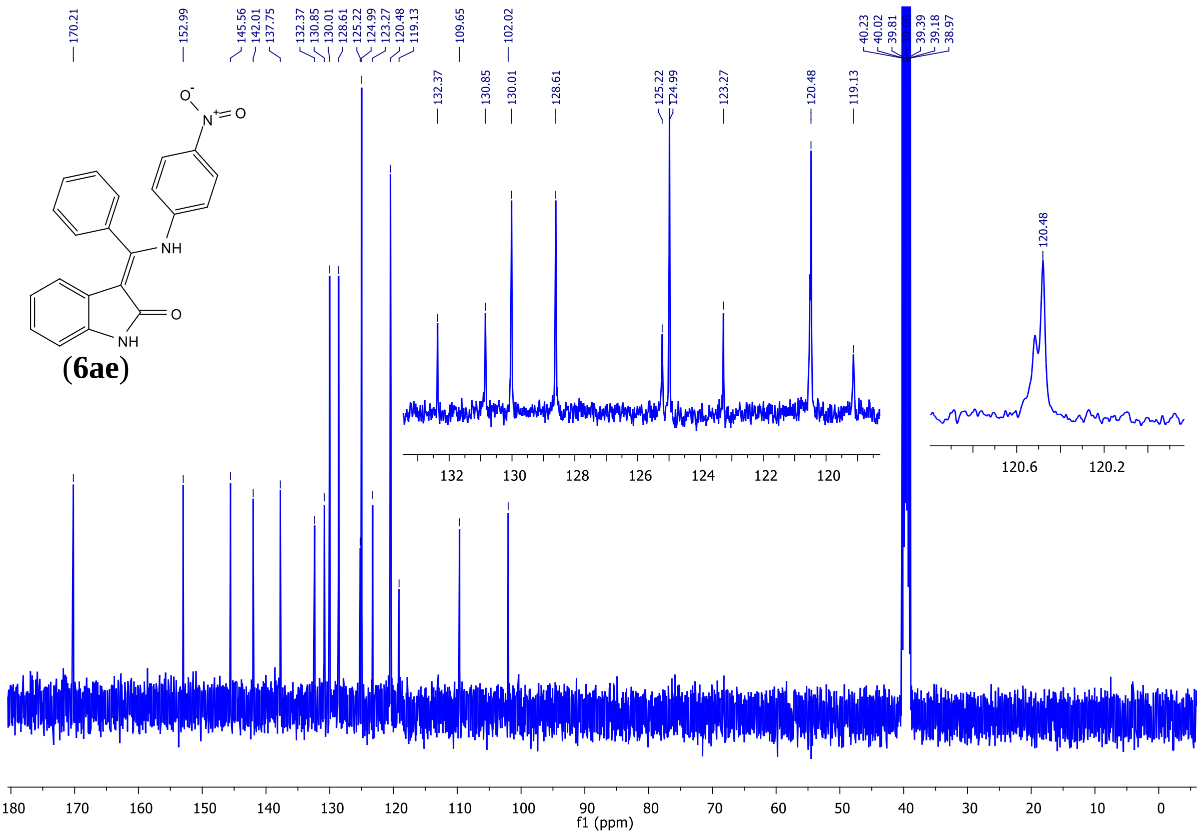
(6ae)
f1 (ppm)

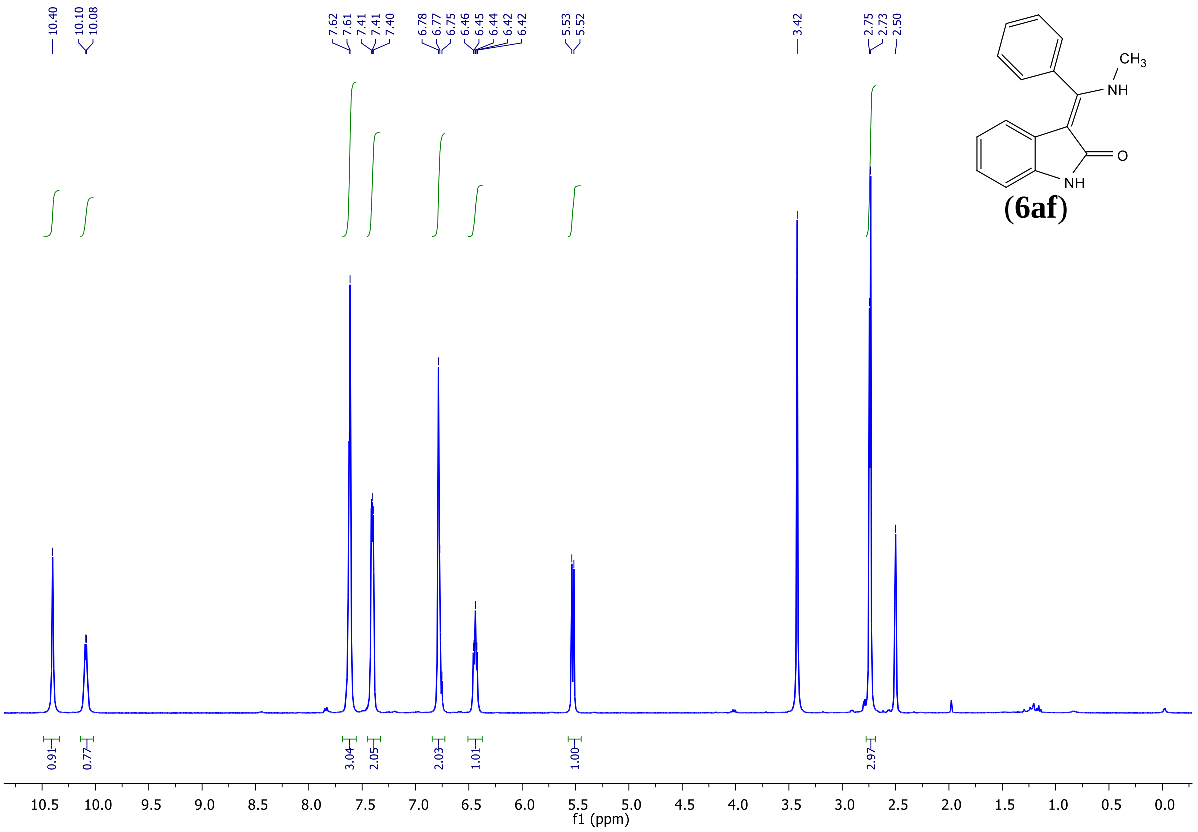
10.40
10.10
10.08
7.62
7.61
7.41
7.41
7.40
6.78
6.77
6.75
6.46
6.45
6.44
6.42
6.42
5.53
5.52
3.42
2.75
2.73
2.50
CH3
NH
O
NH
(6af)
0.91
0.77
3.04
2.05
2.03
1.01
1.00
2.97
10.5 10.0 9.5 9.0 8.5 8.0 7.5 7.0 6.5 6.0 5.5 5.0 4.5 4.0 3.5 3.0 2.5 2.0 1.5 1.0 0.5 0.0
f1 (ppm)

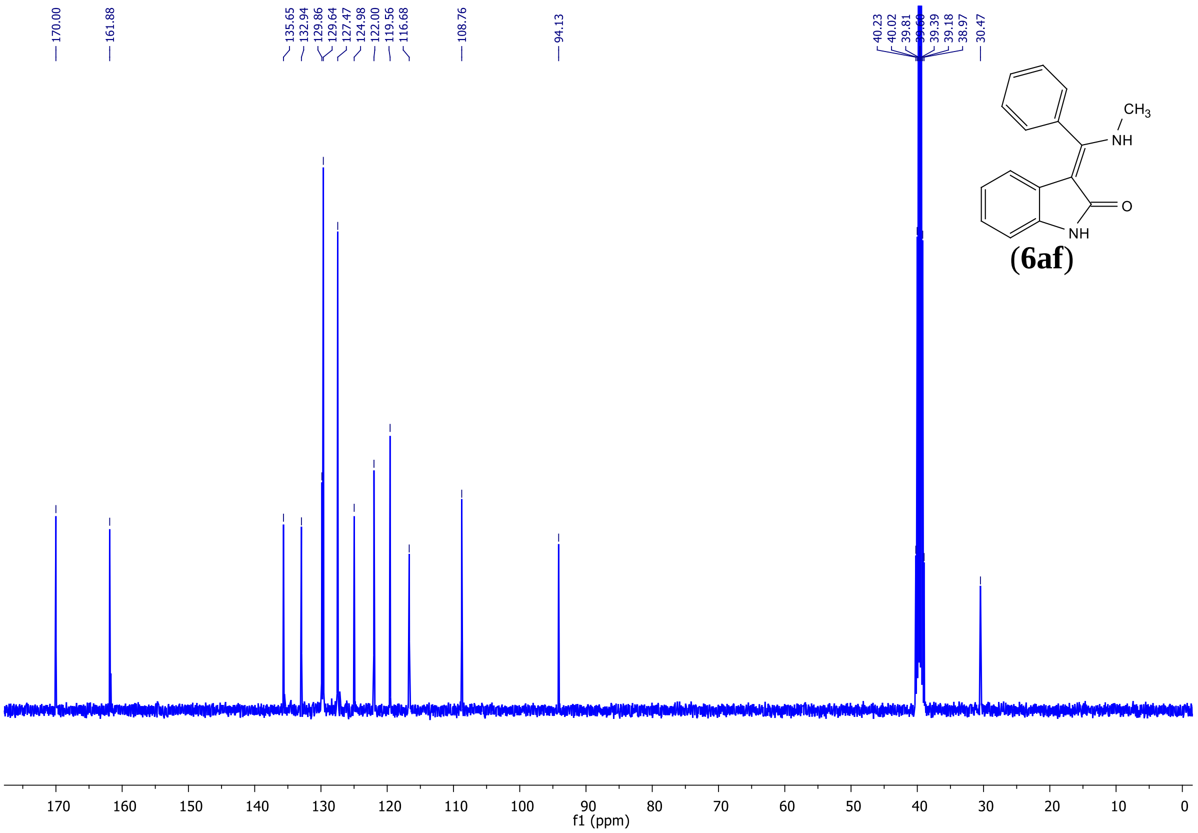
170.00
161.88
135.65
132.94
129.86
129.64
127.47
124.98
122.00
119.56
116.68
108.76
94.13
40.23
40.02
39.81
39.39
39.18
38.97
30.47
CH3
NH
O
NH
(6af)
170 160 150 140 130 120 110 100 90 80 70 60 50 40 30 20 10 0
f1 (ppm)

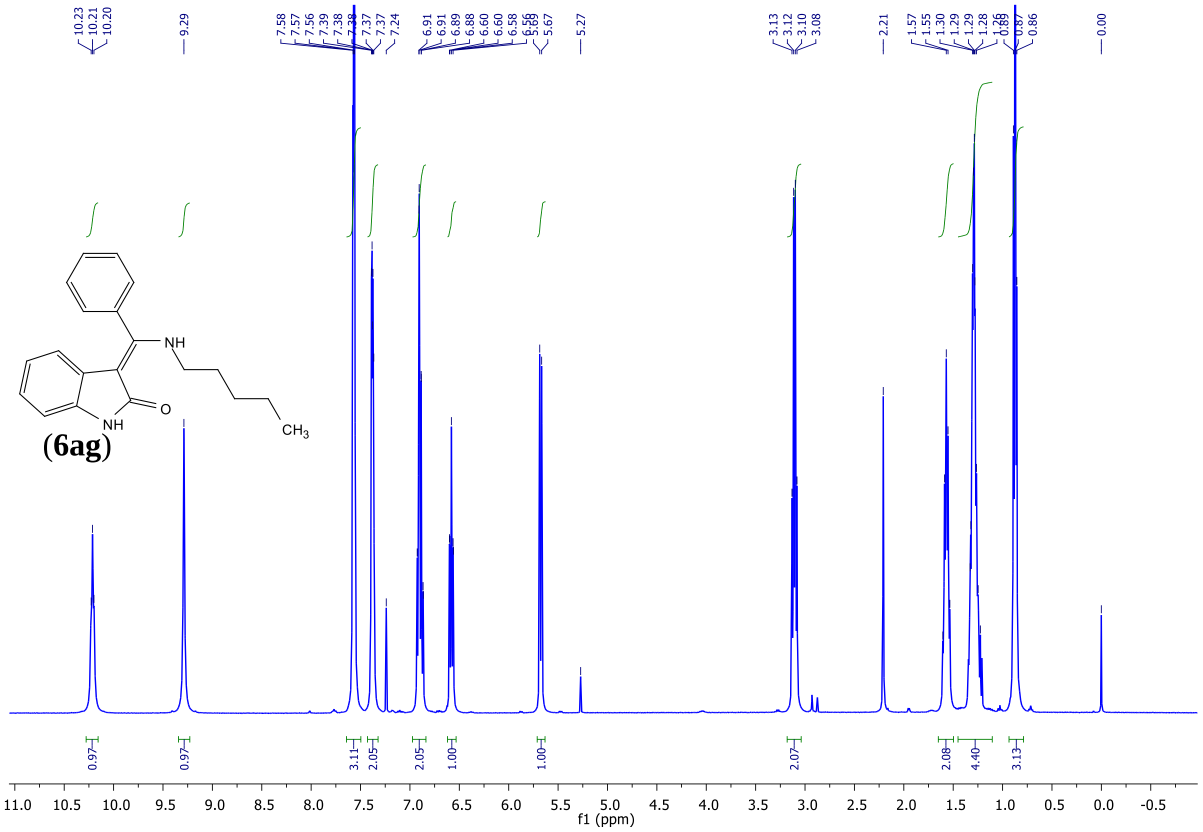

(6ag)

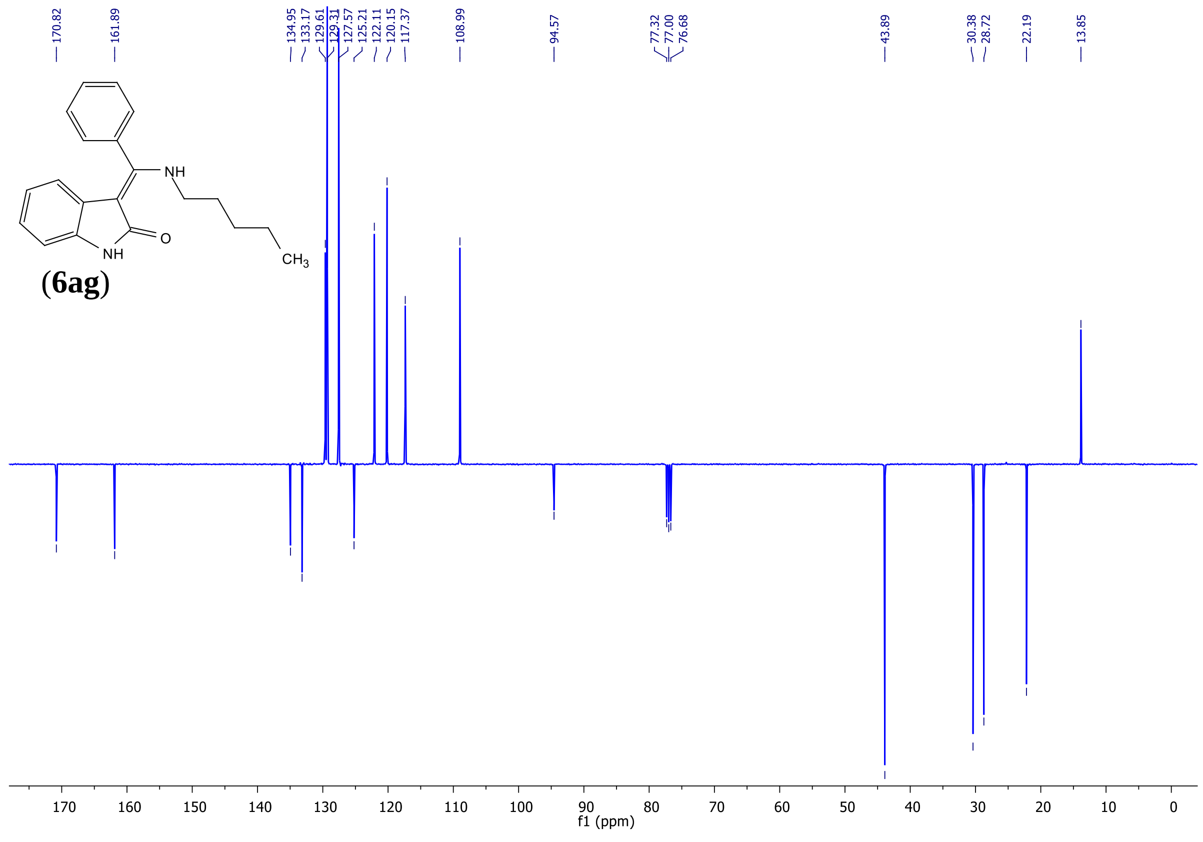

(6ag)

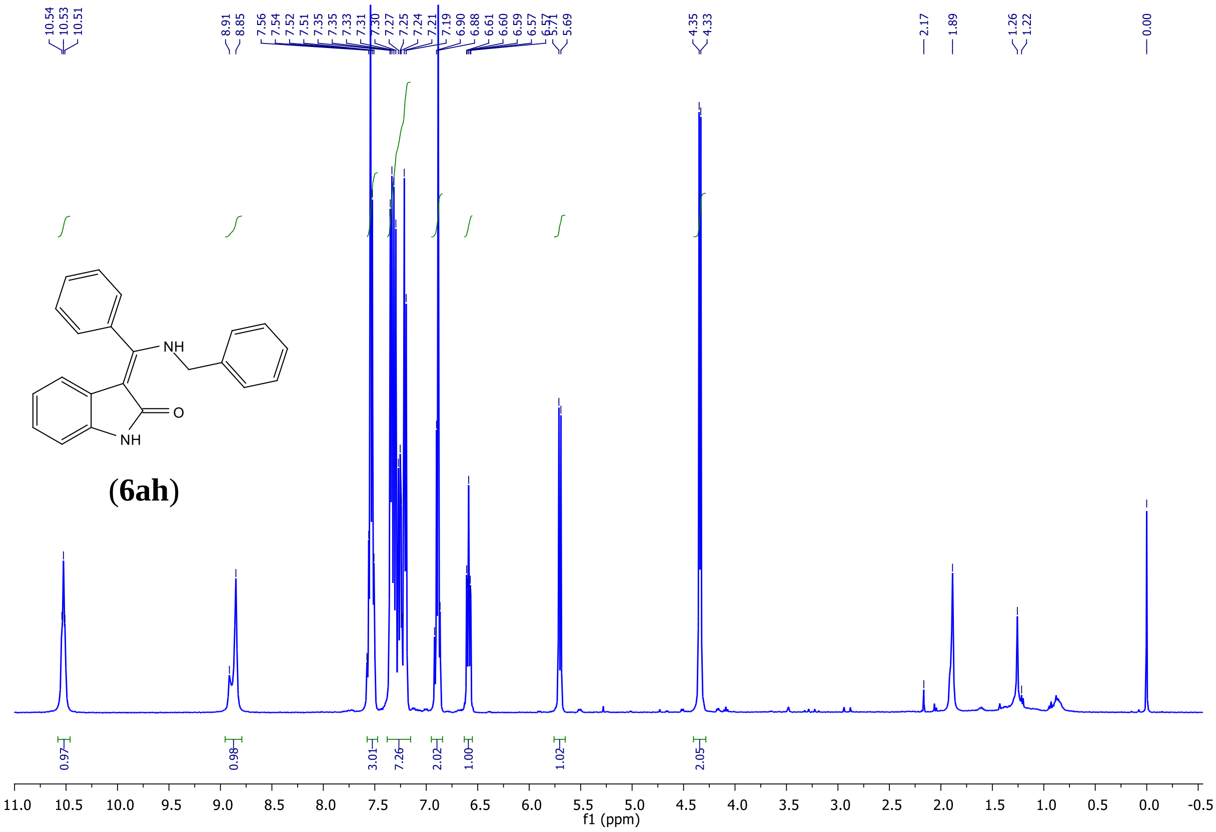

(6ah)

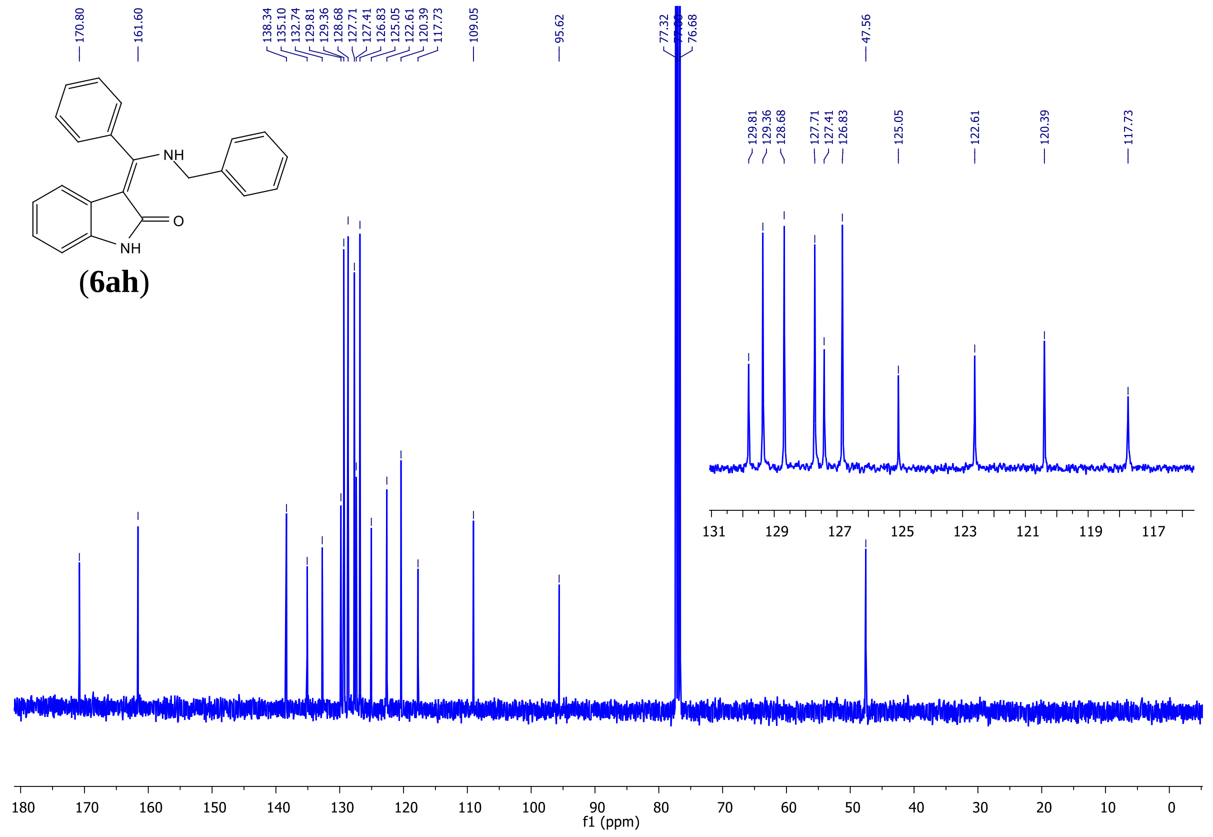

(6ah)

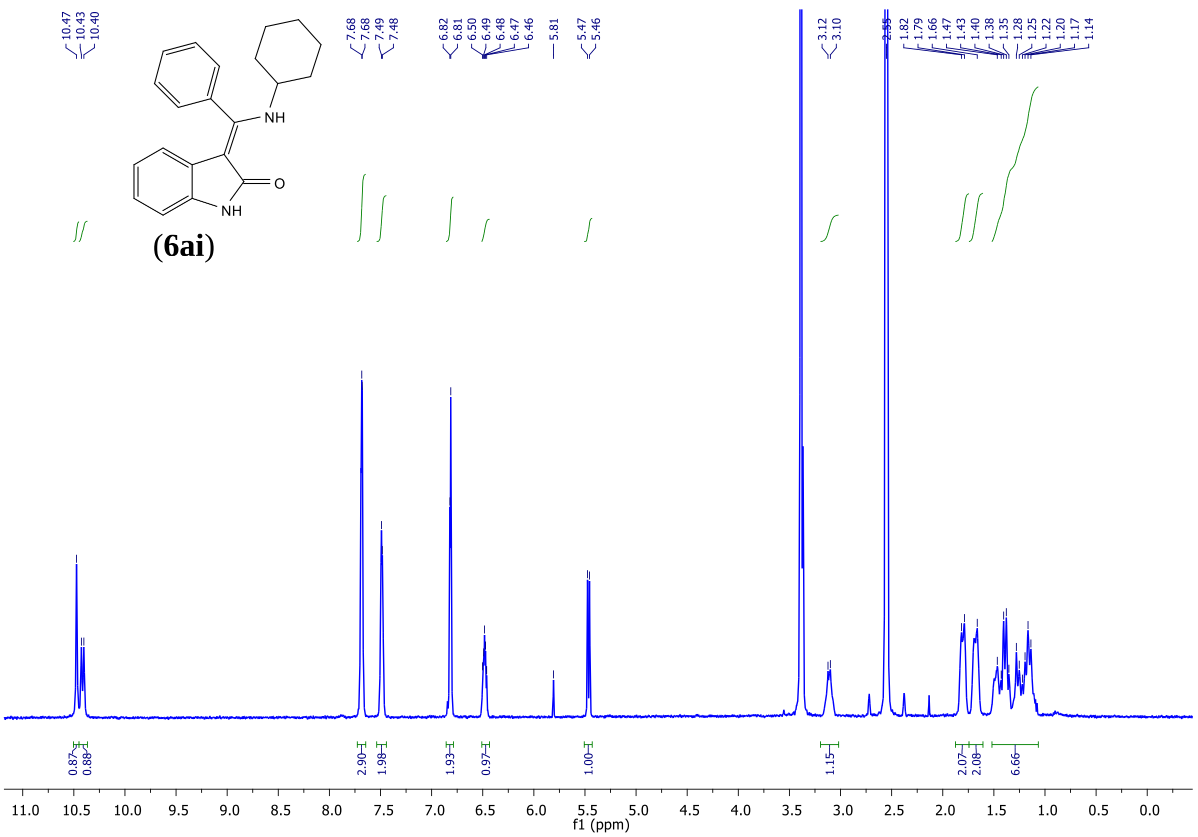

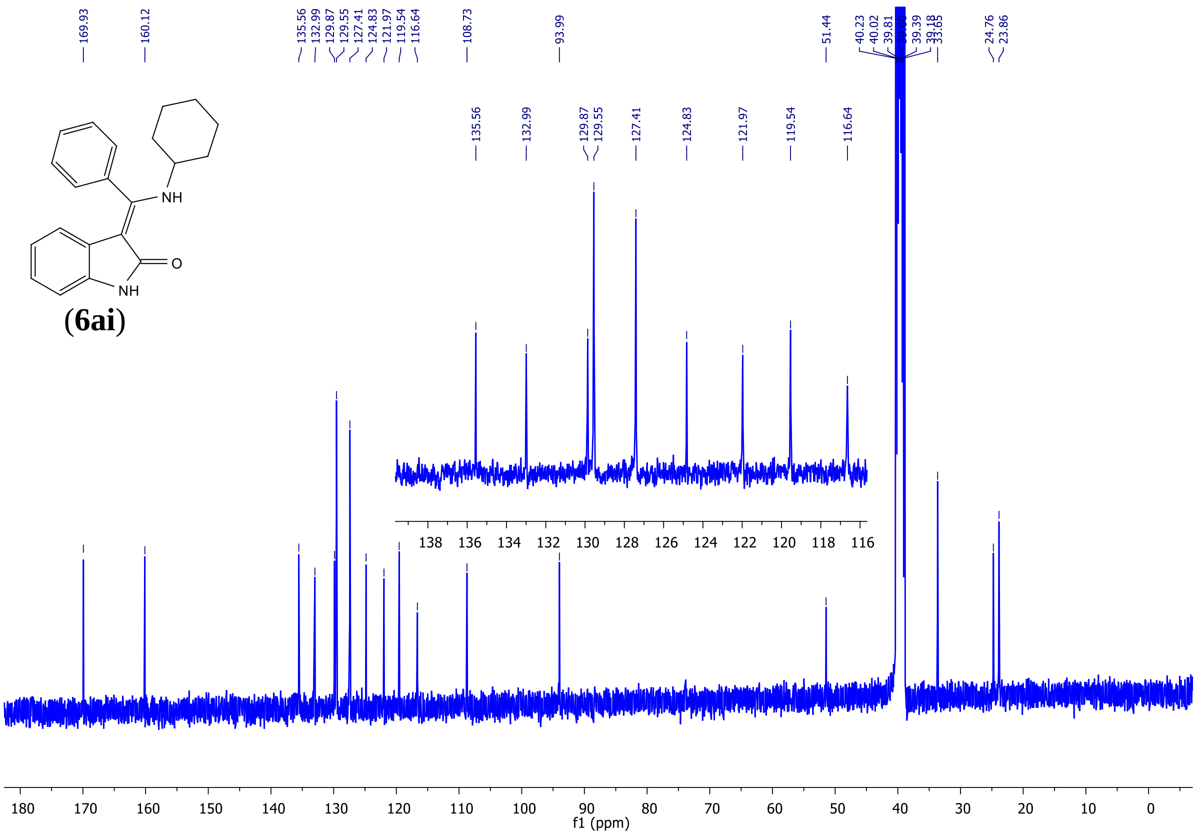

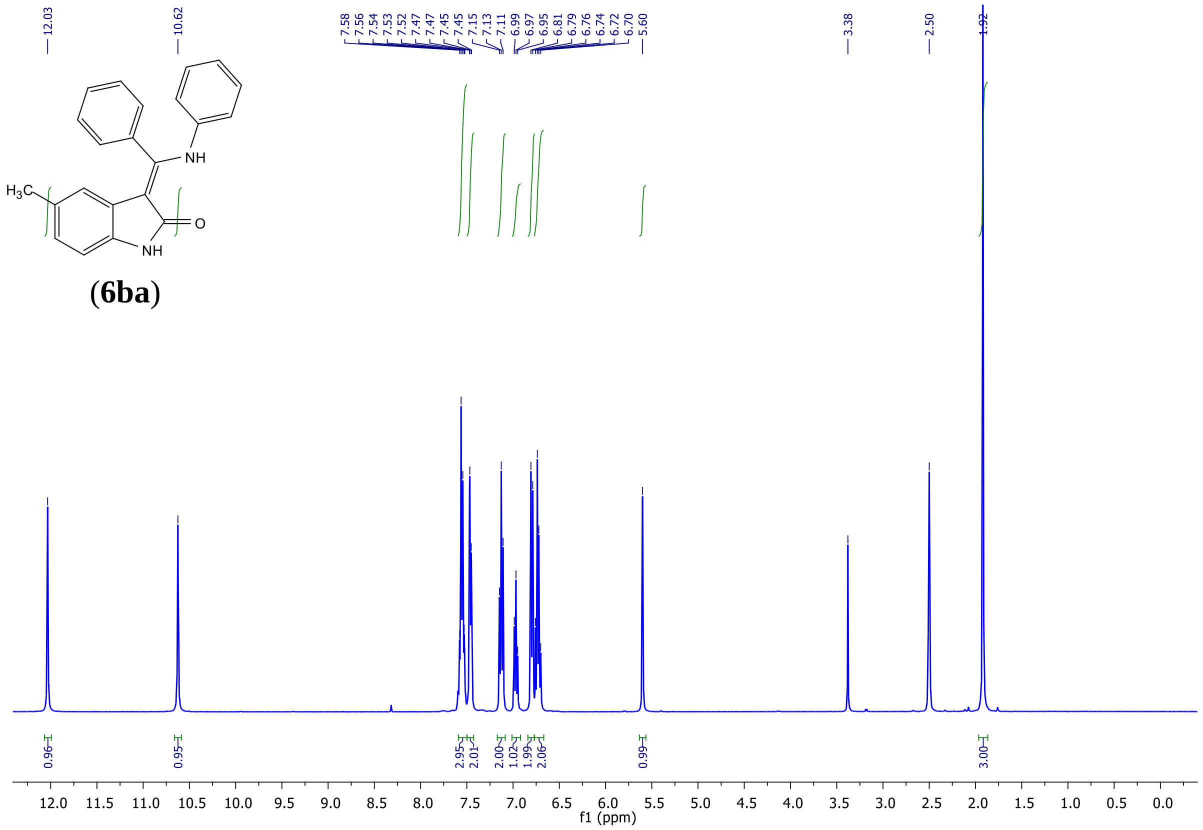
12.03
10.62
7.58
7.56
7.54
7.53
7.52
7.47
7.47
7.45
7.45
7.15
7.13
7.11
6.99
6.97
6.95
6.81
6.79
6.76
6.74
6.72
6.70
5.60
3.38
2.50
1.92
H_3C
NH
O
NH
0.96
0.95
2.95
2.01
2.00
1.02
1.99
2.06
0.99
3.00
12.0 11.5 11.0 10.5 10.0 9.5 9.0 8.5 8.0 7.5 7.0 6.5 6.0 5.5 5.0 4.5 4.0 3.5 3.0 2.5 2.0 1.5 1.0 0.5 0.0
f1 (ppm)

(6ba)

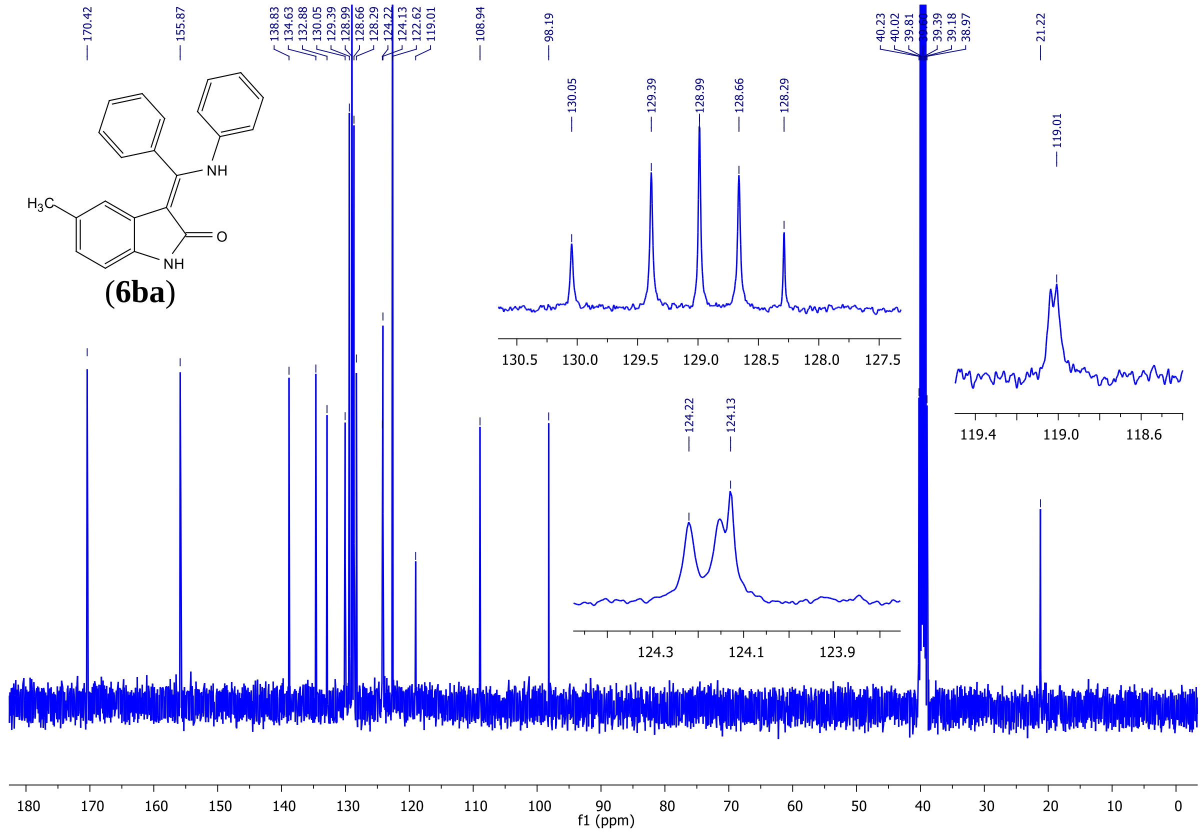
170.42
155.87
138.83
134.63
132.88
130.05
129.39
128.99
128.66
128.29
124.22
124.13
122.62
119.01
108.94
98.19
40.23
40.02
39.81
39.39
39.18
38.97
21.22
H_3C
NH
O
NH
130.05
129.39
128.99
128.66
128.29
130.5 130.0 129.5 129.0 128.5 128.0 127.5
119.01
119.4 119.0 118.6
124.22
124.13
124.3 124.1 123.9
180 170 160 150 140 130 120 110 100 90 80 70 60 50 40 30 20 10 0
f1 (ppm)

(6ba)

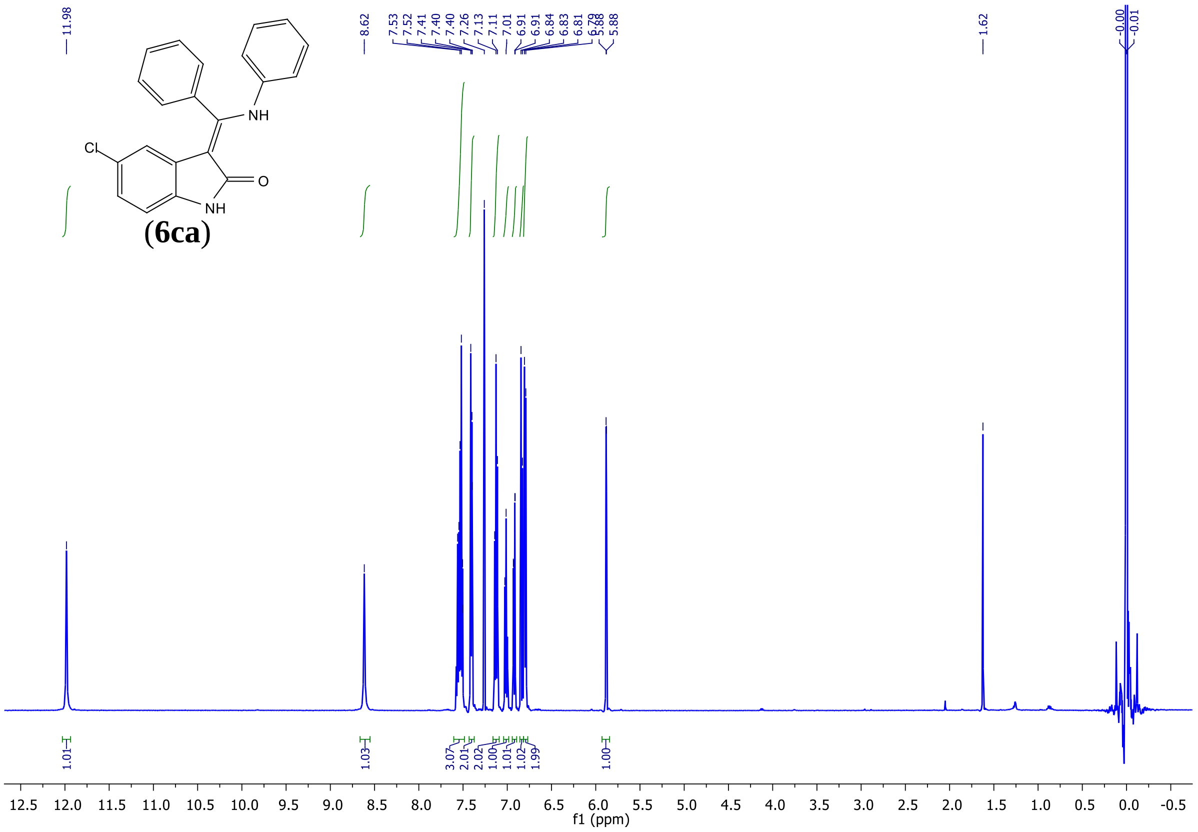

(6ca)

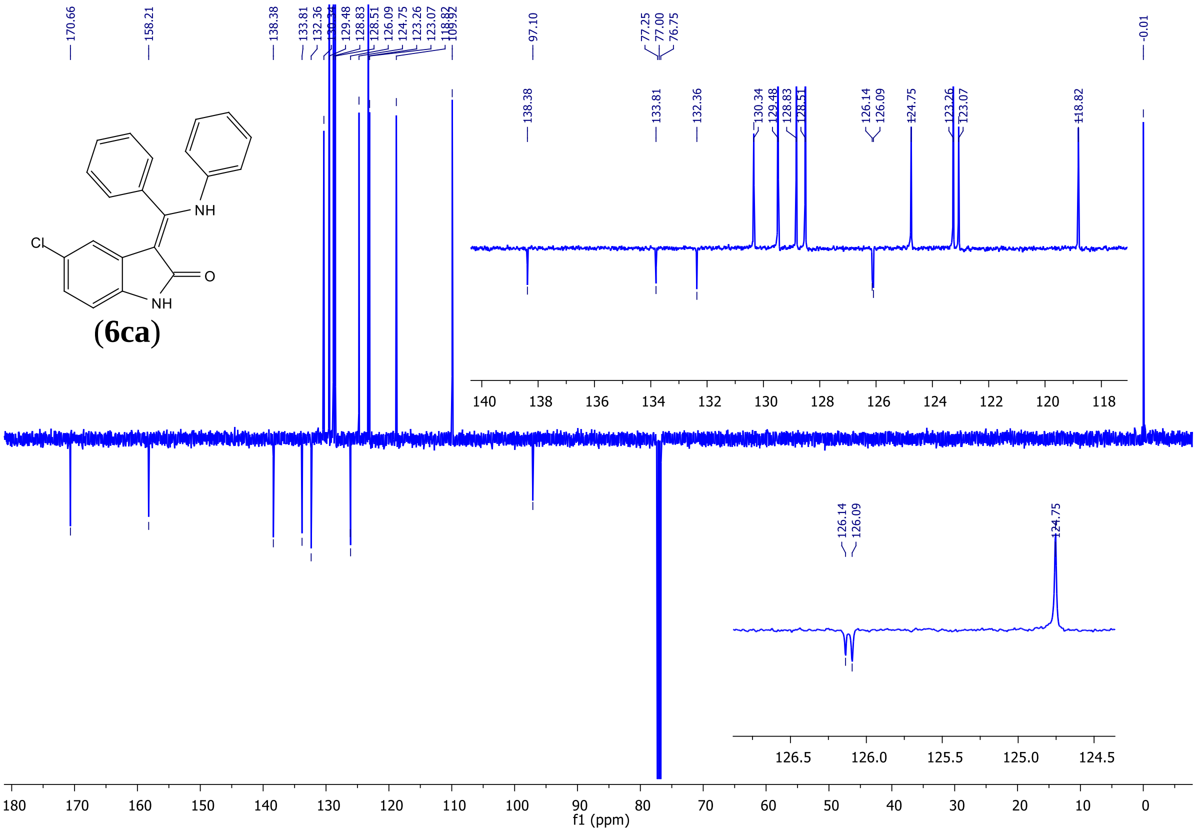

(6ca)

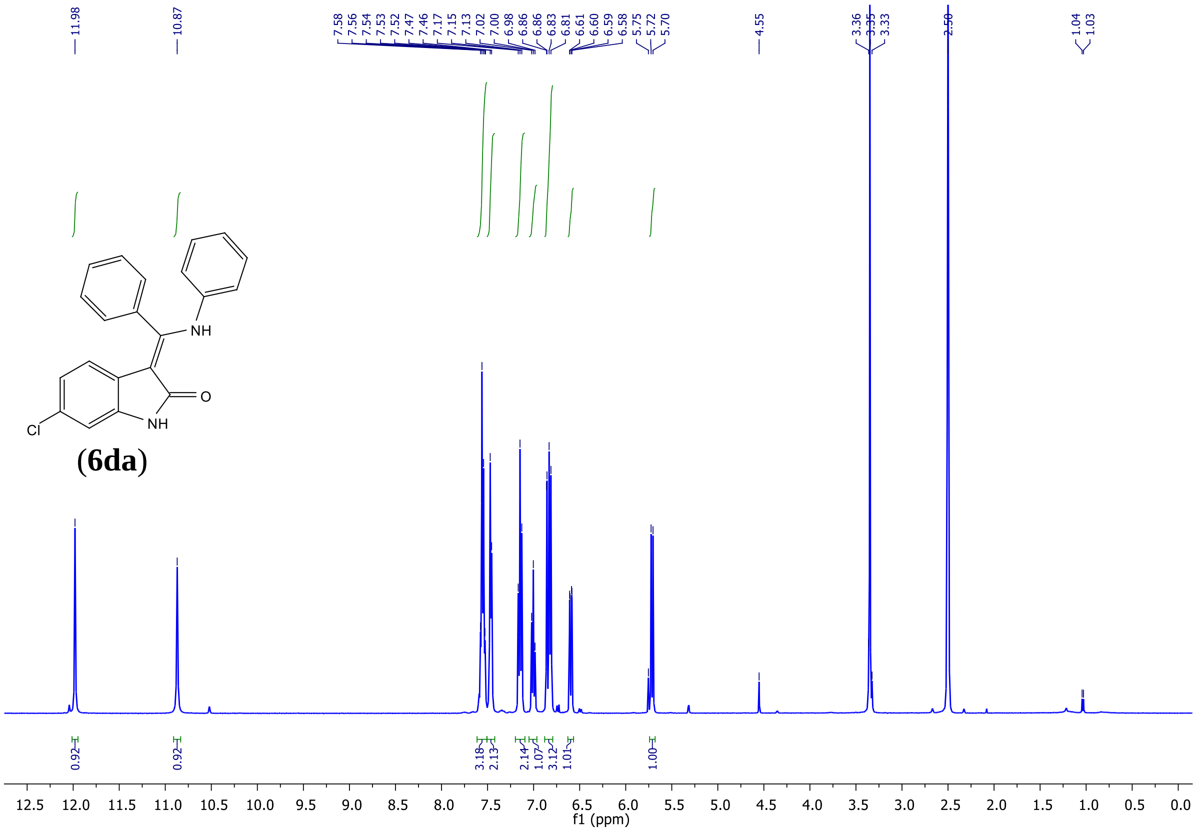
11.98
10.87
7.58 7.56 7.54 7.53 7.52 7.47 7.46 7.17 7.15 7.13 7.02 7.00 6.98 6.86 6.86 6.83 6.81 6.61 6.60 6.59 6.58 5.75 5.72 5.70
4.55
3.36 3.35 3.33
2.50
1.04 1.03
NH
O
NH
Cl
(6da)
0.92
0.92
3.18 2.13 2.14 1.07 3.12 1.01
1.00
12.5 12.0 11.5 11.0 10.5 10.0 9.5 9.0 8.5 8.0 7.5 7.0 6.5 6.0 5.5 5.0 4.5 4.0 3.5 3.0 2.5 2.0 1.5 1.0 0.5 0.0
f1 (ppm)

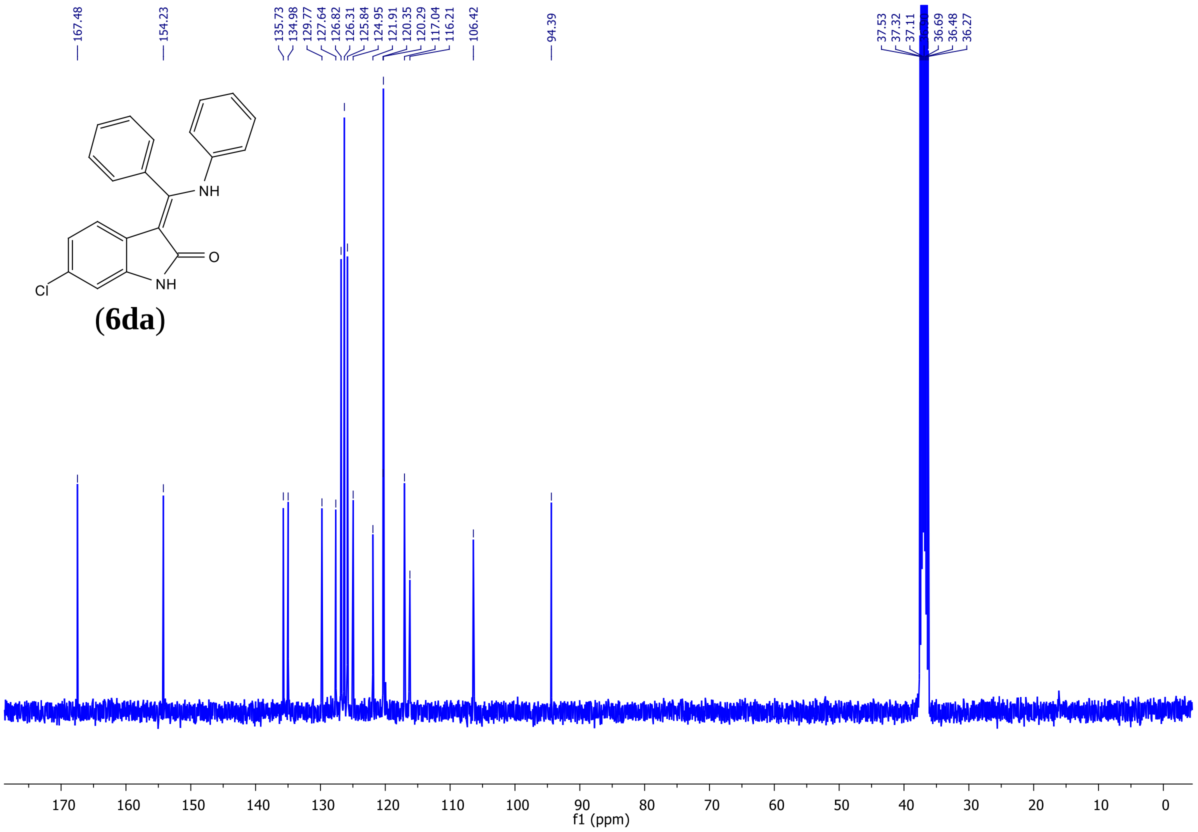
167.48
154.23
135.73 134.98 129.77 127.64 126.82 126.31 125.84 124.95 121.91 120.35 120.29 117.04 116.21
106.42
94.39
37.53 37.32 37.11 36.69 36.48 36.27
NH
O
NH
Cl
(6da)
170 160 150 140 130 120 110 100 90 80 70 60 50 40 30 20 10 0
f1 (ppm)

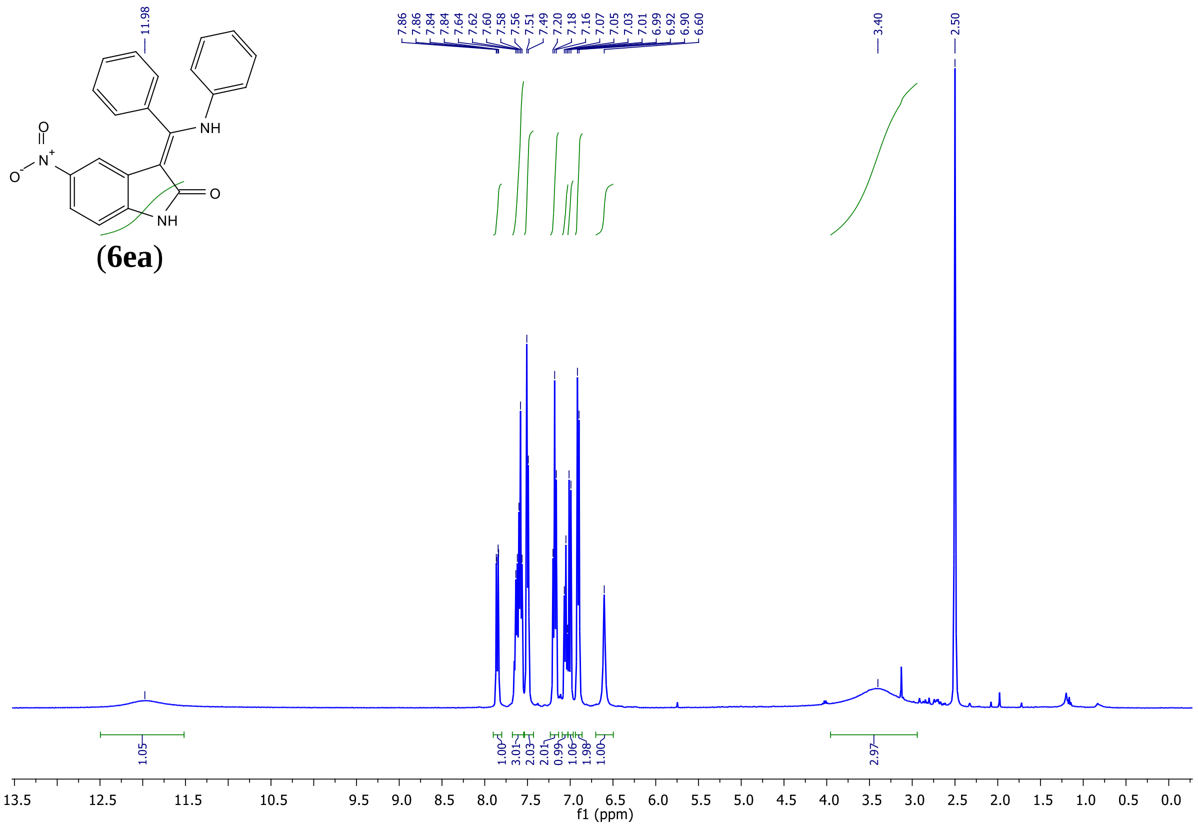

11.98
7.86 7.86 7.84 7.84 7.64 7.62 7.60 7.58 7.56 7.51 7.49 7.20 7.18 7.16 7.07 7.05 7.03 7.01 6.99 6.92 6.90 6.60
3.40
2.50
O
N+
O-
NH
O
NH
(6ea)
1.05
1.00 3.01 2.03 2.01 0.99 1.06 1.98 1.00
2.97
f1 (ppm)

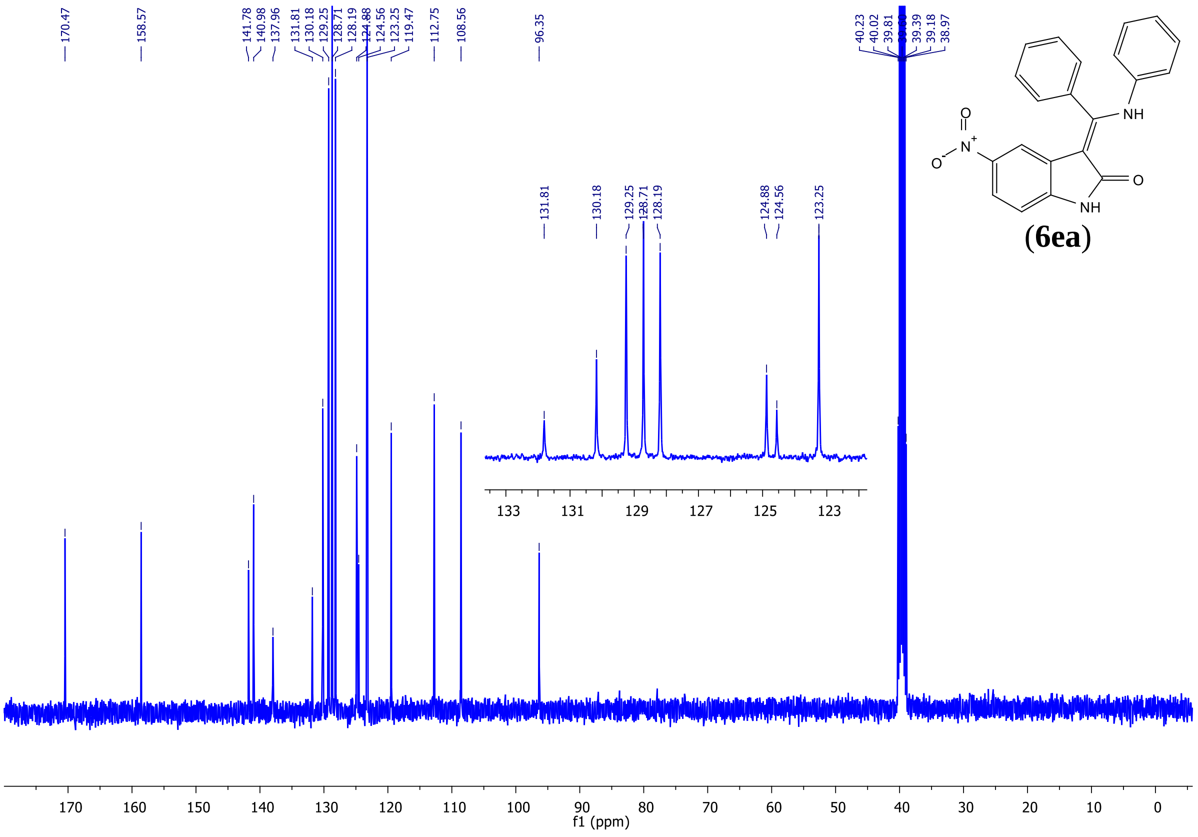

170.47
158.57
141.78 140.98 137.96 131.81 130.18 129.25 128.71 128.19 124.88 124.56 123.25 119.47 112.75 108.56
96.35
40.23 40.02 39.81 39.39 39.18 38.97
131.81 130.18 129.25 128.71 128.19 124.88 124.56 123.25
O
N+
O-
NH
O
NH
(6ea)
f1 (ppm)

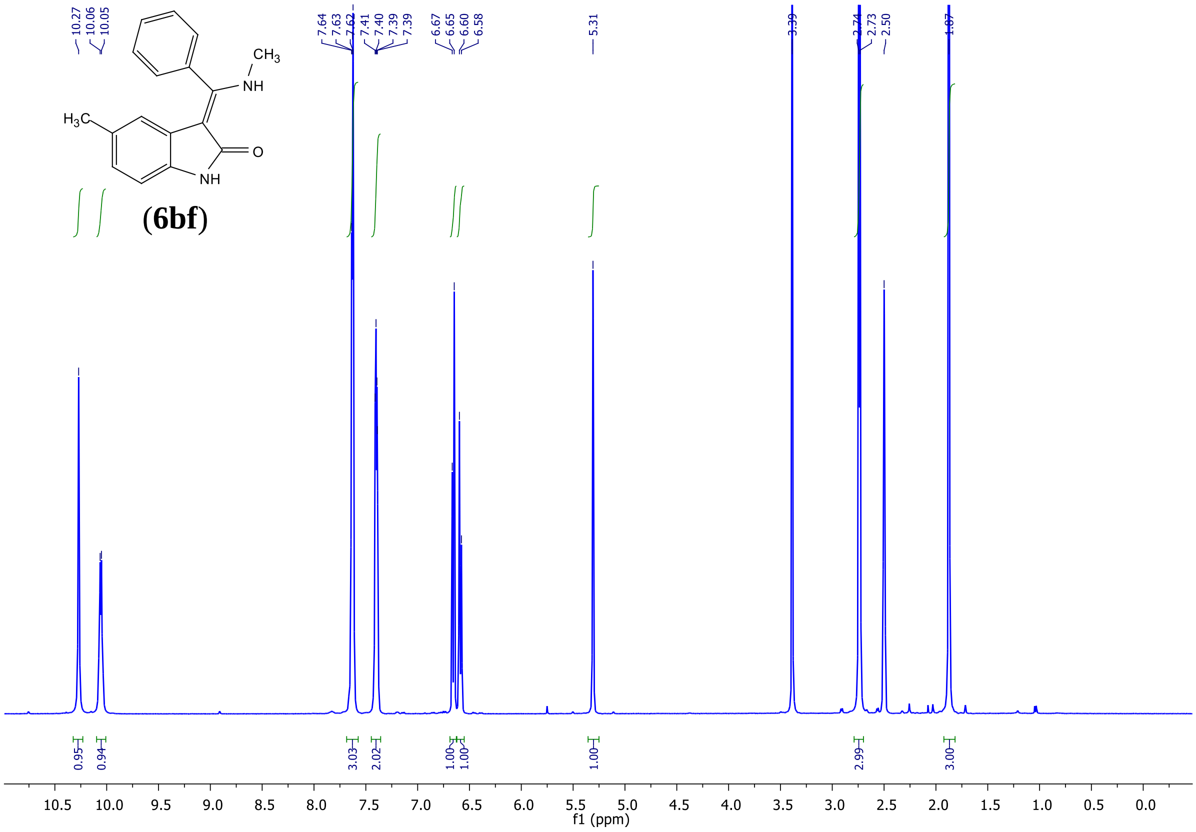

(6bf)

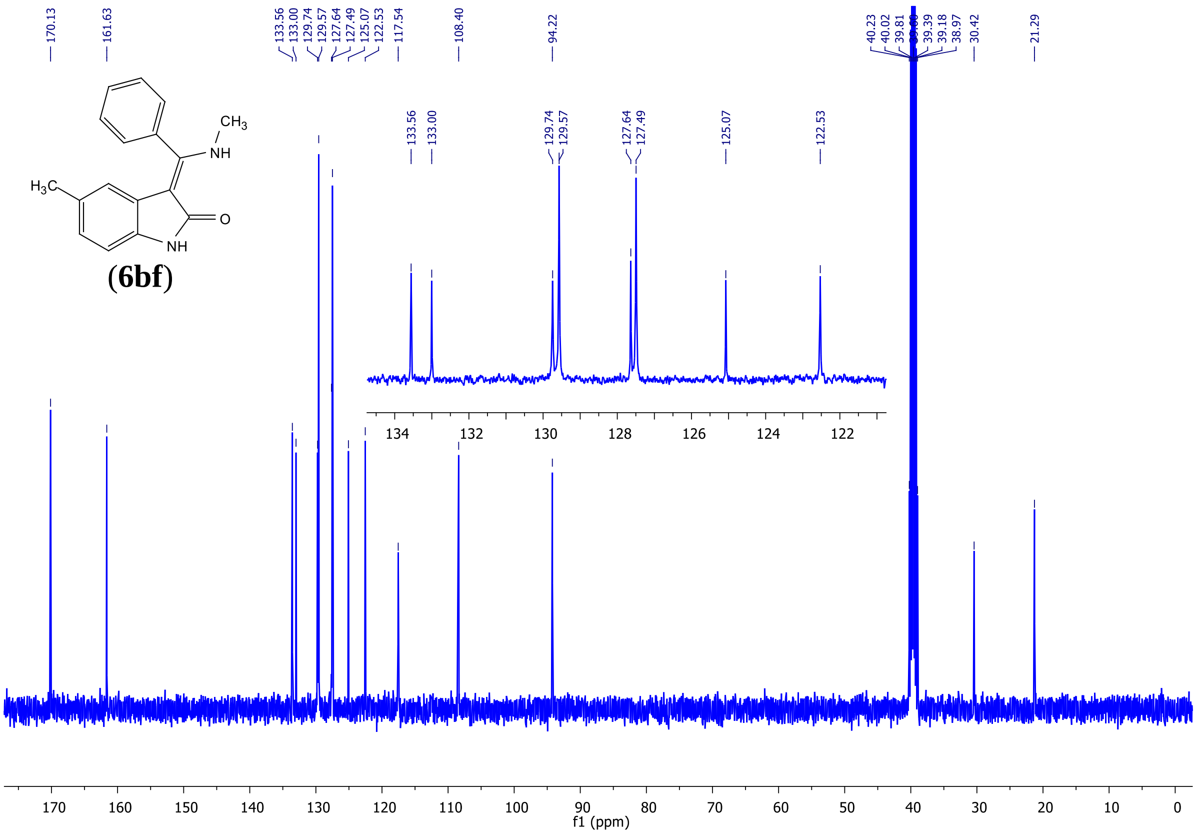

(6bf)

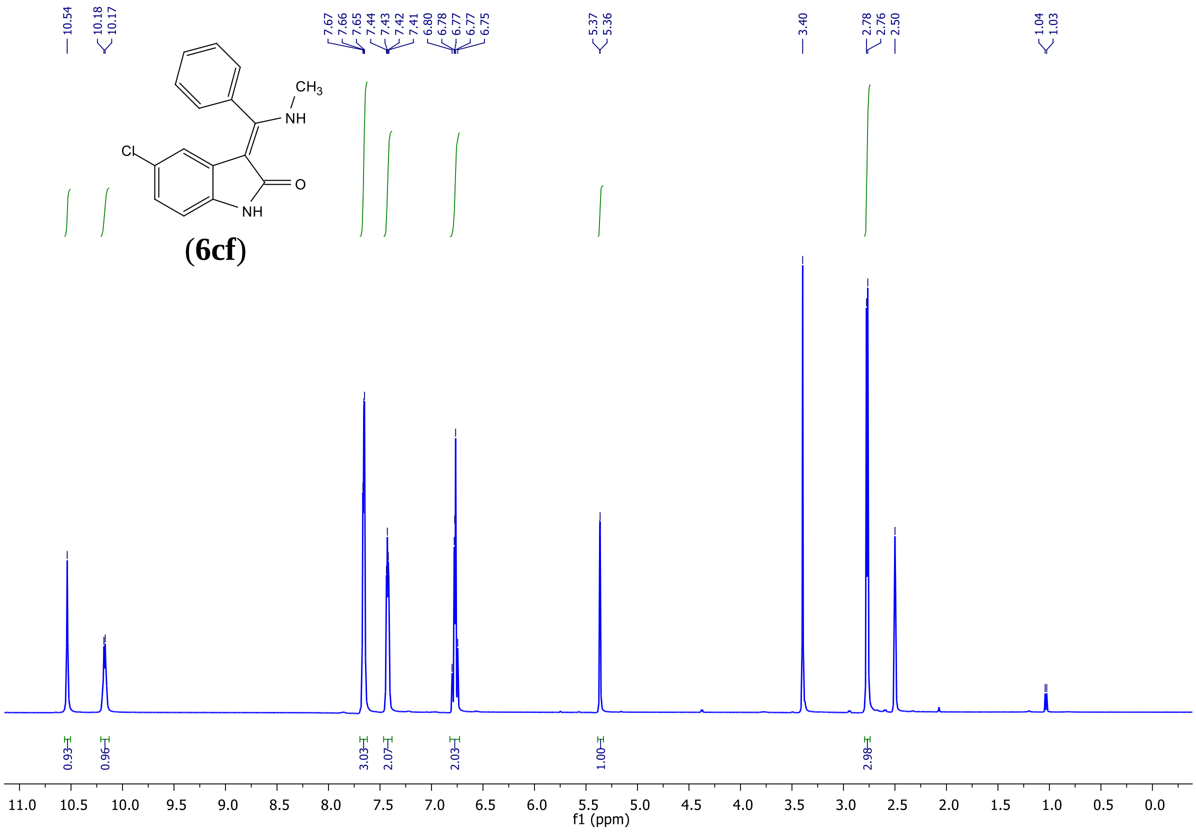
10.54
10.18
10.17
7.67
7.66
7.65
7.44
7.43
7.42
7.41
6.80
6.78
6.77
6.77
6.75
5.37
5.36
3.40
2.78
2.76
2.50
1.04
1.03
CH3
NH
Cl
O
NH
0.93
0.96
3.03
2.07
2.03
1.00
2.98
f1 (ppm)

(6cf)

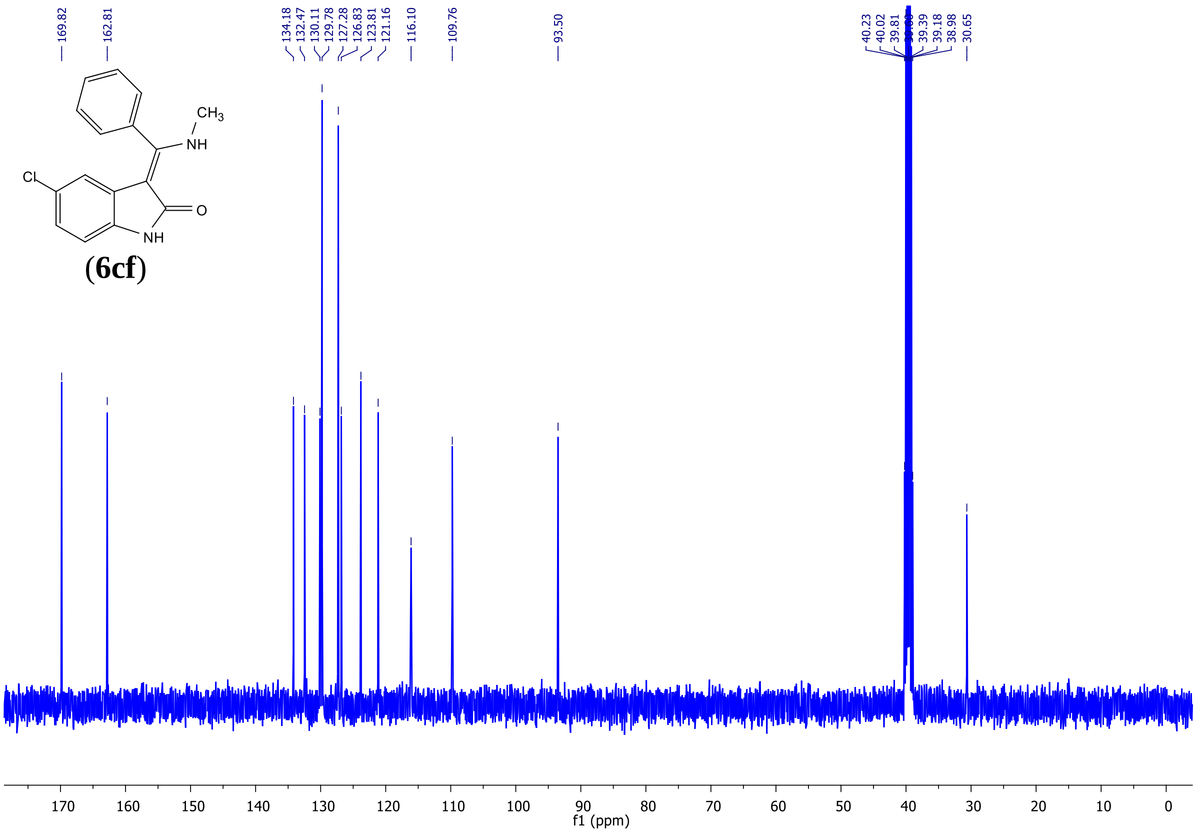
169.82
162.81
134.18
132.47
130.11
129.78
127.28
126.83
123.81
121.16
116.10
109.76
93.50
40.23
40.02
39.81
39.39
39.18
38.98
30.65
CH3
NH
Cl
O
NH
f1 (ppm)

(6cf)

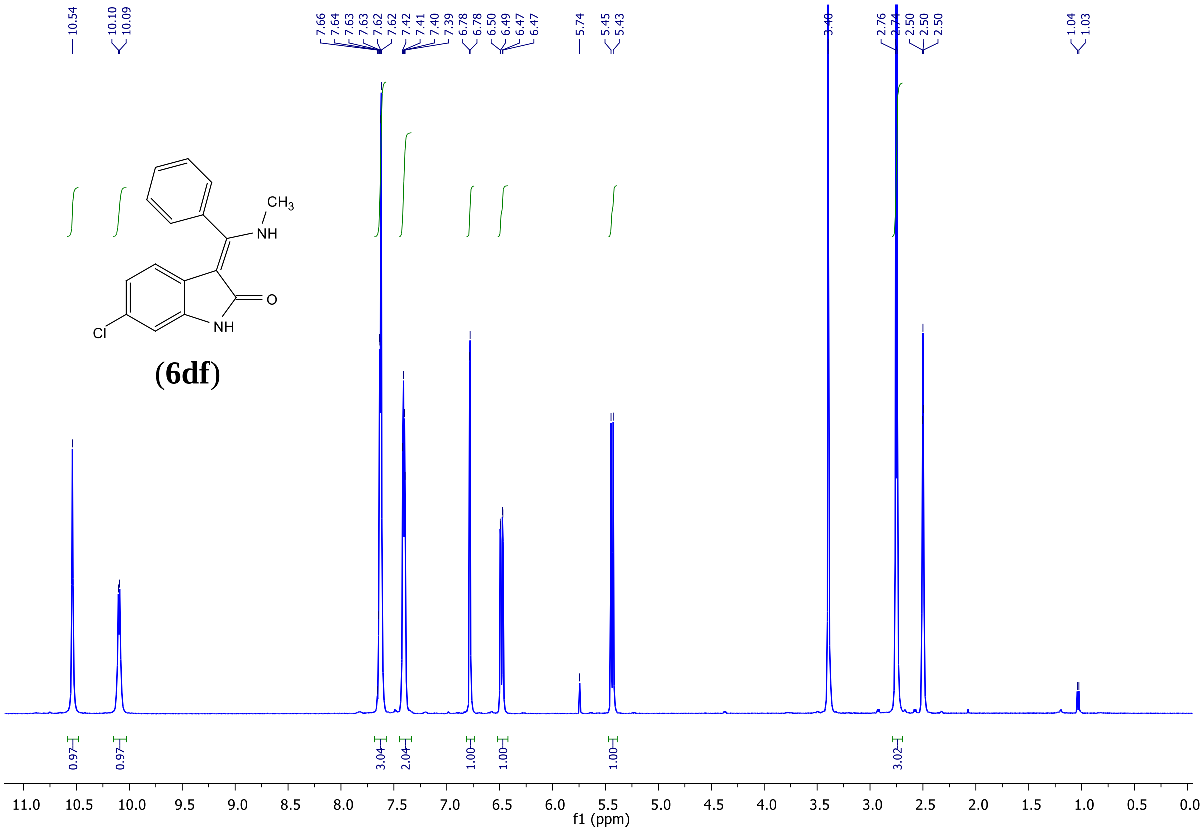

10.54
10.10
10.09
7.66
7.64
7.63
7.62
7.62
7.42
7.41
7.40
7.39
6.78
6.78
6.50
6.49
6.47
6.47
5.74
5.45
5.43
3.40
2.76
2.74
2.50
2.50
2.50
1.04
1.03
CH3
NH
O
NH
Cl
0.97
0.97
3.04
2.04
1.00
1.00
1.00
3.02
f1 (ppm)

(6df)

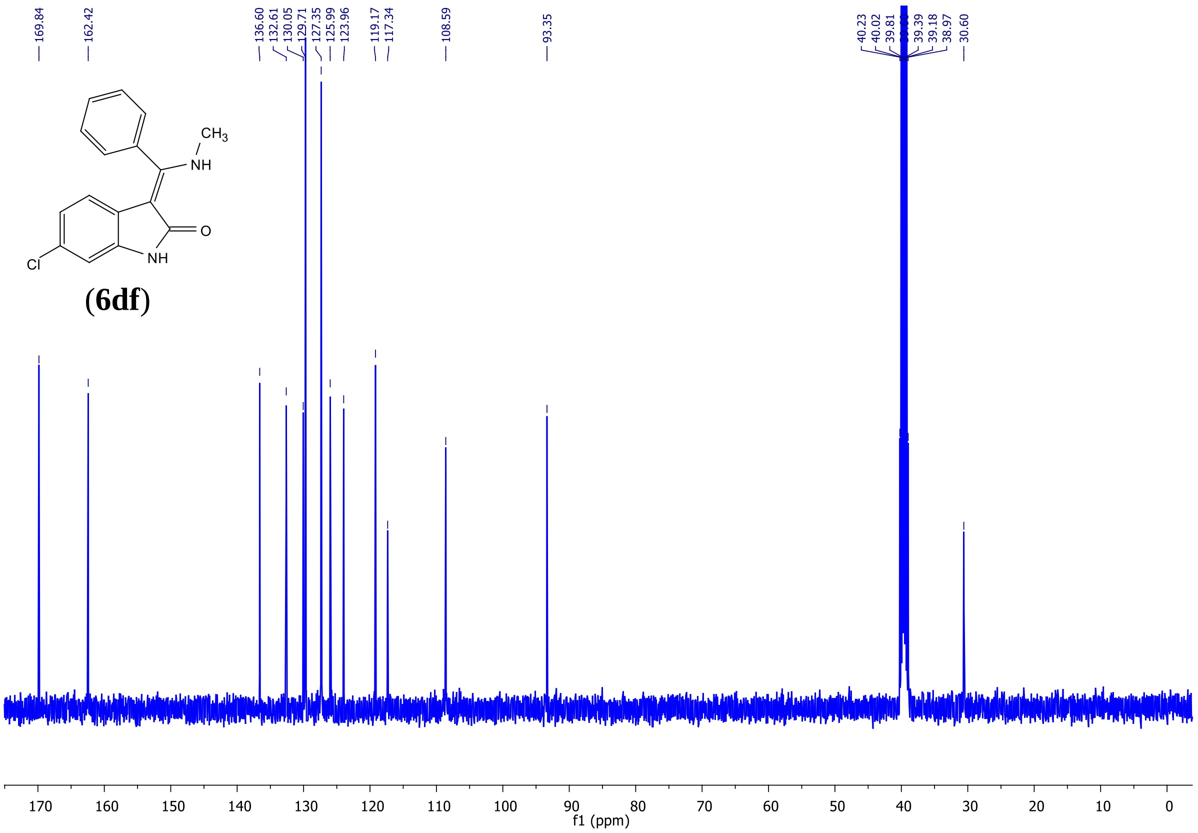

169.84
162.42
136.60
132.61
130.05
129.71
127.35
125.99
123.96
119.17
117.34
108.59
93.35
40.23
40.02
39.81
39.39
39.18
38.97
30.60
CH3
NH
O
NH
Cl
f1 (ppm)

(6df)

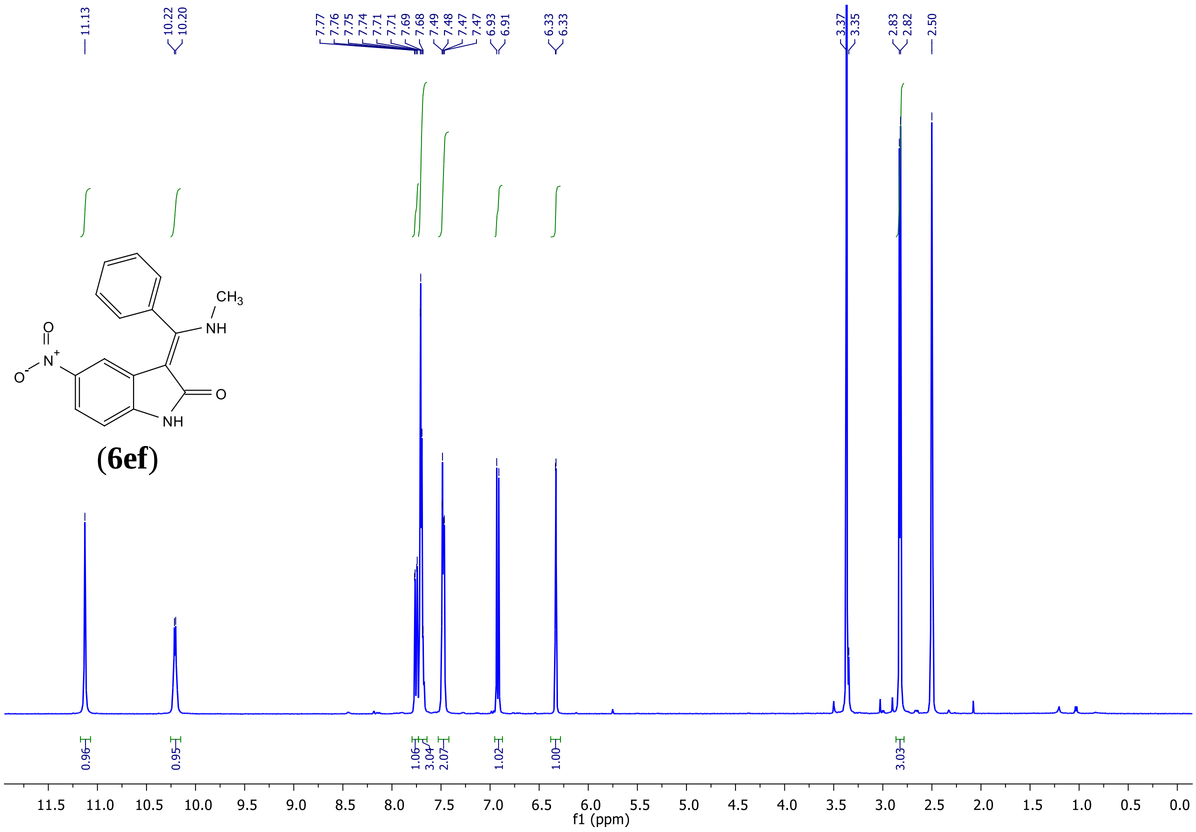

11.13
10.22
10.20
7.77
7.76
7.75
7.74
7.71
7.71
7.69
7.68
7.49
7.48
7.47
7.47
6.93
6.91
6.33
6.33
3.37
3.35
2.83
2.82
2.50
CH3
NH
O
N+
O-
O
NH
(6ef)
0.96
0.95
1.06
3.04
2.07
1.02
1.00
3.03
11.5
11.0
10.5
10.0
9.5
9.0
8.5
8.0
7.5
7.0
6.5
6.0
5.5
5.0
4.5
4.0
3.5
3.0
2.5
2.0
1.5
1.0
0.5
0.0
f1 (ppm)

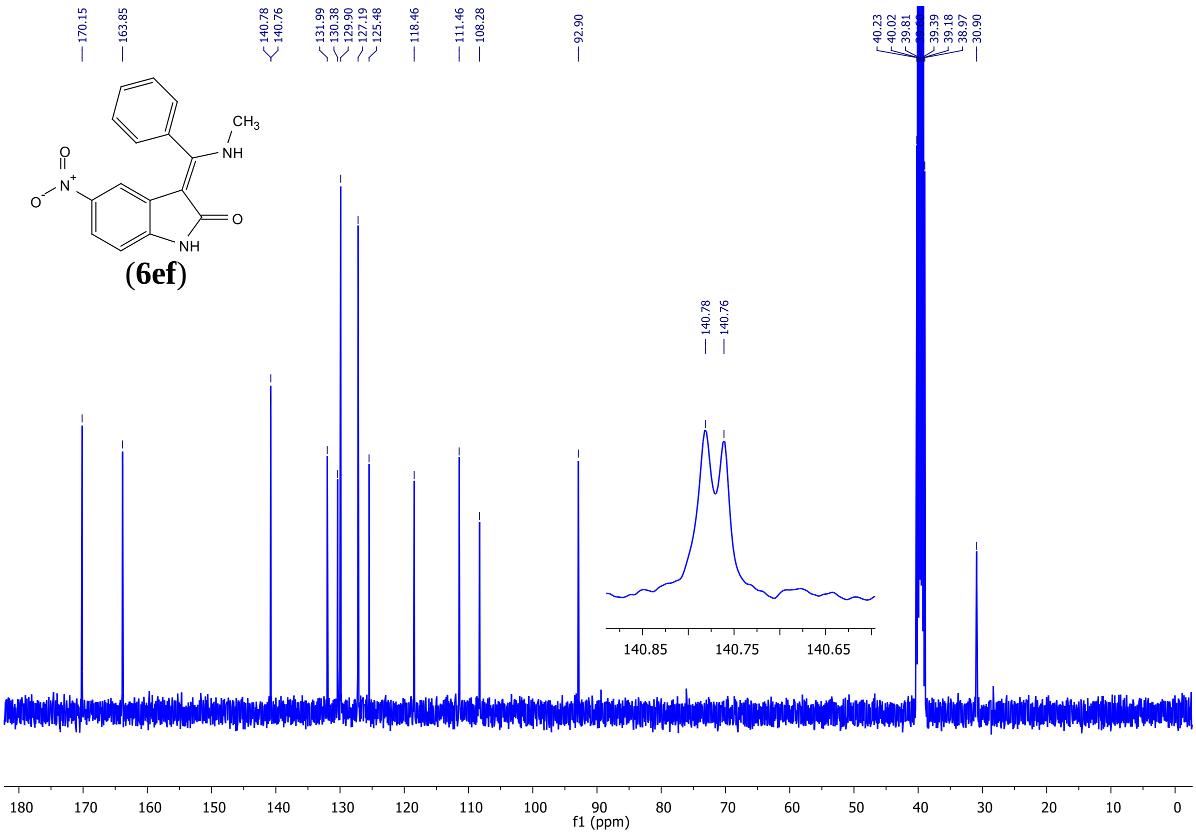

170.15
163.85
140.78
140.76
131.99
130.38
129.90
127.19
125.48
118.46
111.46
108.28
92.90
40.23
40.02
39.81
39.39
39.18
38.97
30.90
CH3
NH
O
N+
O-
O
NH
(6ef)
140.78
140.76
140.85
140.75
140.65
180
170
160
150
140
130
120
110
100
90
80
70
60
50
40
30
20
10
0
f1 (ppm)

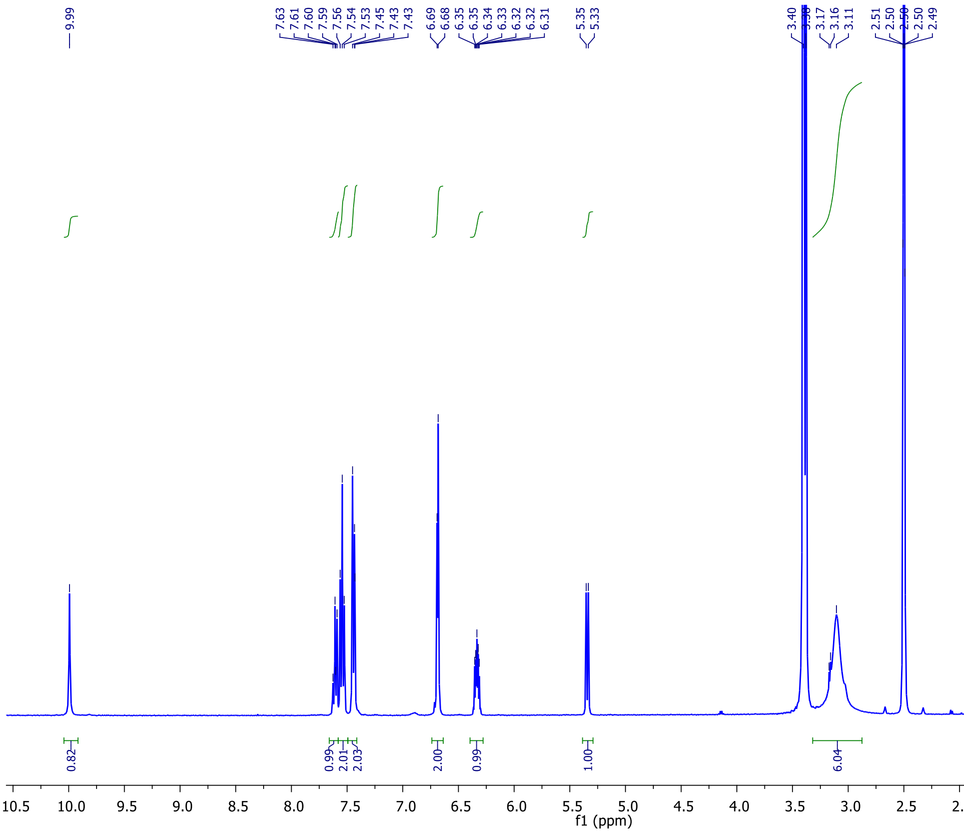
9.99
7.63 7.61 7.60 7.59 7.56 7.54 7.53 7.45 7.43 7.43
6.69 6.68
6.35 6.35 6.34 6.33 6.32 6.32 6.31
5.35 5.33
3.40 3.17 3.16 3.11
2.51 2.50 2.50 2.49
0.82 0.99 2.01 2.03 2.00 0.99 1.00 6.04
10.5 10.0 9.5 9.0 8.5 8.0 7.5 7.0 6.5 6.0 5.5 5.0 4.5 4.0 3.5 3.0 2.5 2.0 1.5 1.0 0.5 0.0
f1 (ppm)

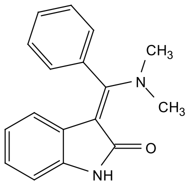
CH3
N
CH3
O
NH

(7aa)

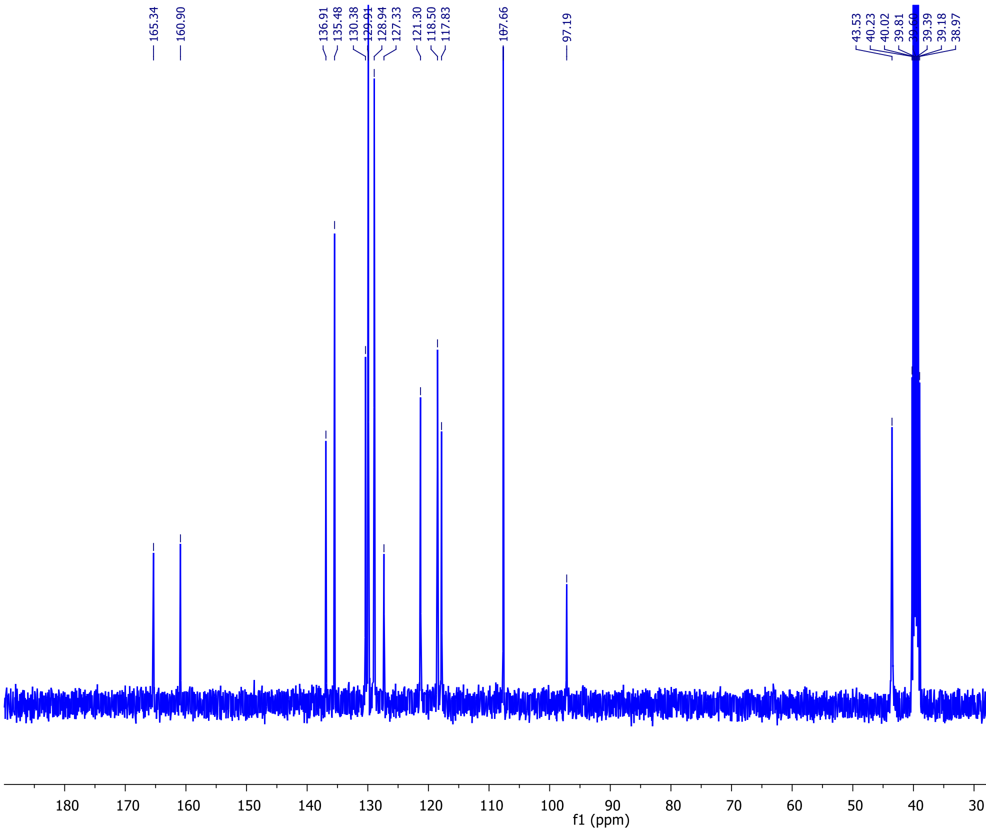
165.34
160.90
136.91 135.48 130.38 128.94 127.33
121.30 118.50 117.83
107.66
97.19
43.53 40.23 40.02 39.81 39.39 39.18 38.97
180 170 160 150 140 130 120 110 100 90 80 70 60 50 40 30 20 10 0
f1 (ppm)

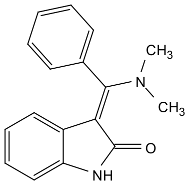
CH3
N
CH3
O
NH

(7aa)

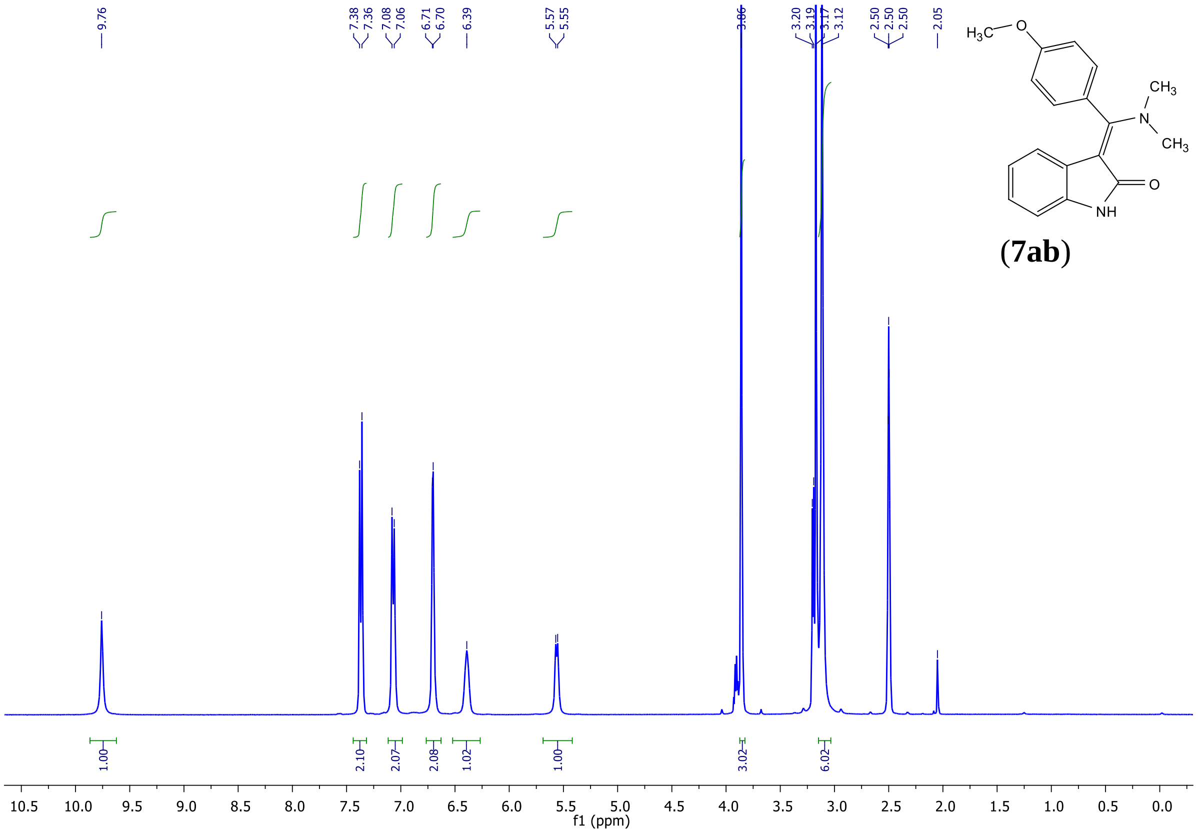
(7ab)
f1 (ppm)

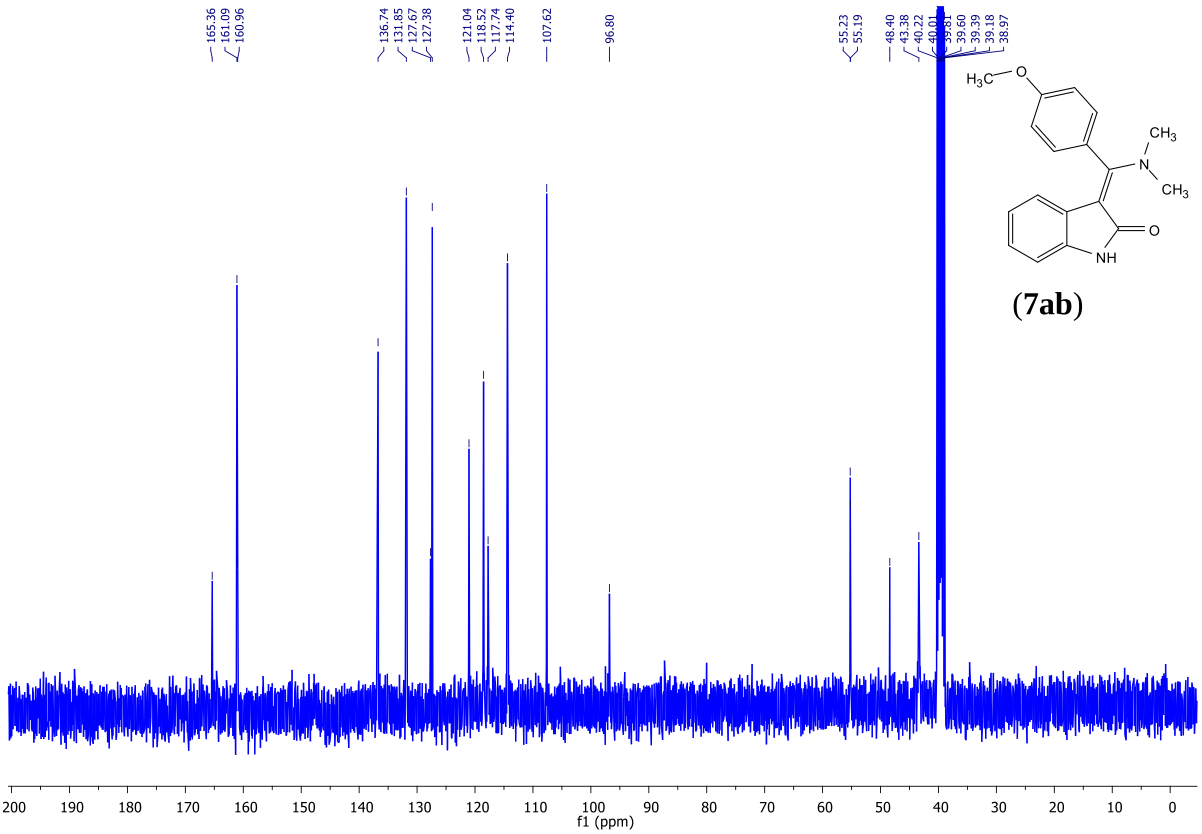
(7ab)
f1 (ppm)

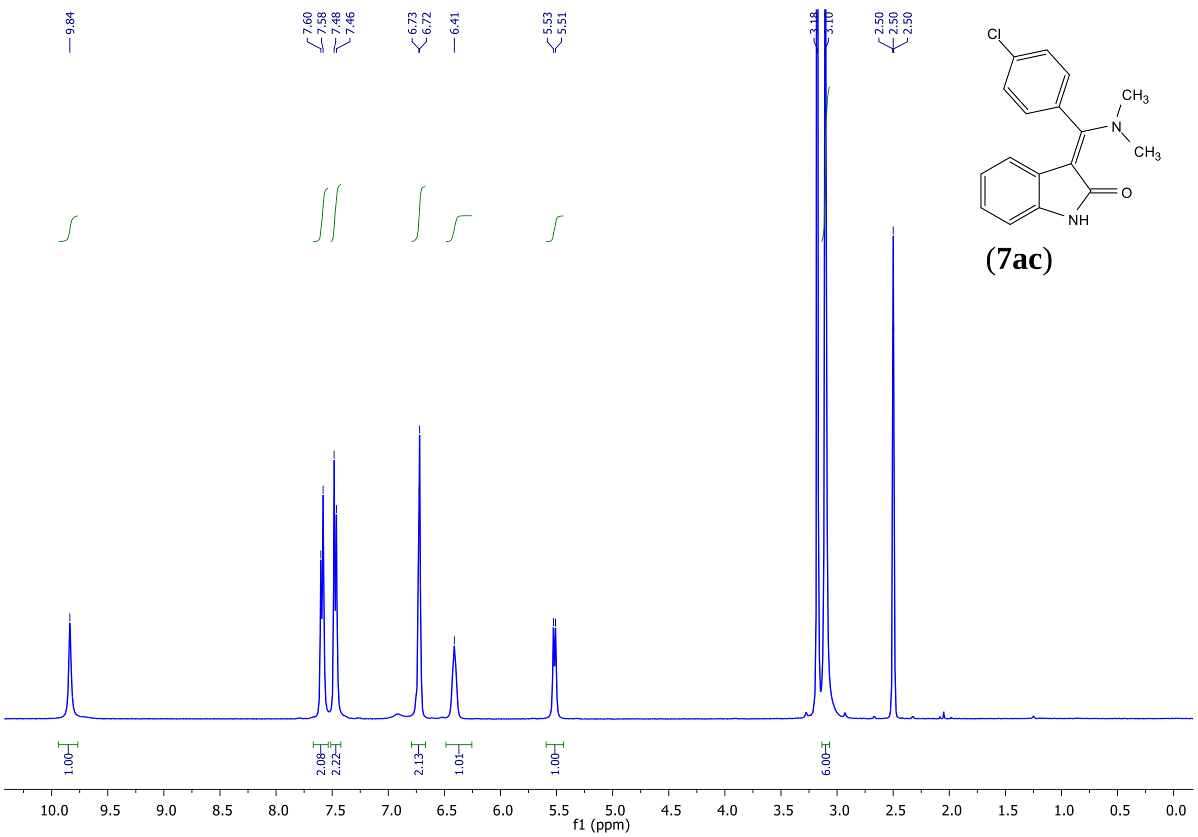
9.84
7.60
7.58
7.48
7.46
6.73
6.72
6.41
5.53
5.51
3.10
2.50
2.50
2.50
1.00
2.08
2.22
2.13
1.01
1.00
6.00
f1 (ppm)
Cl
CH3
N
CH3
O
NH
(7ac)

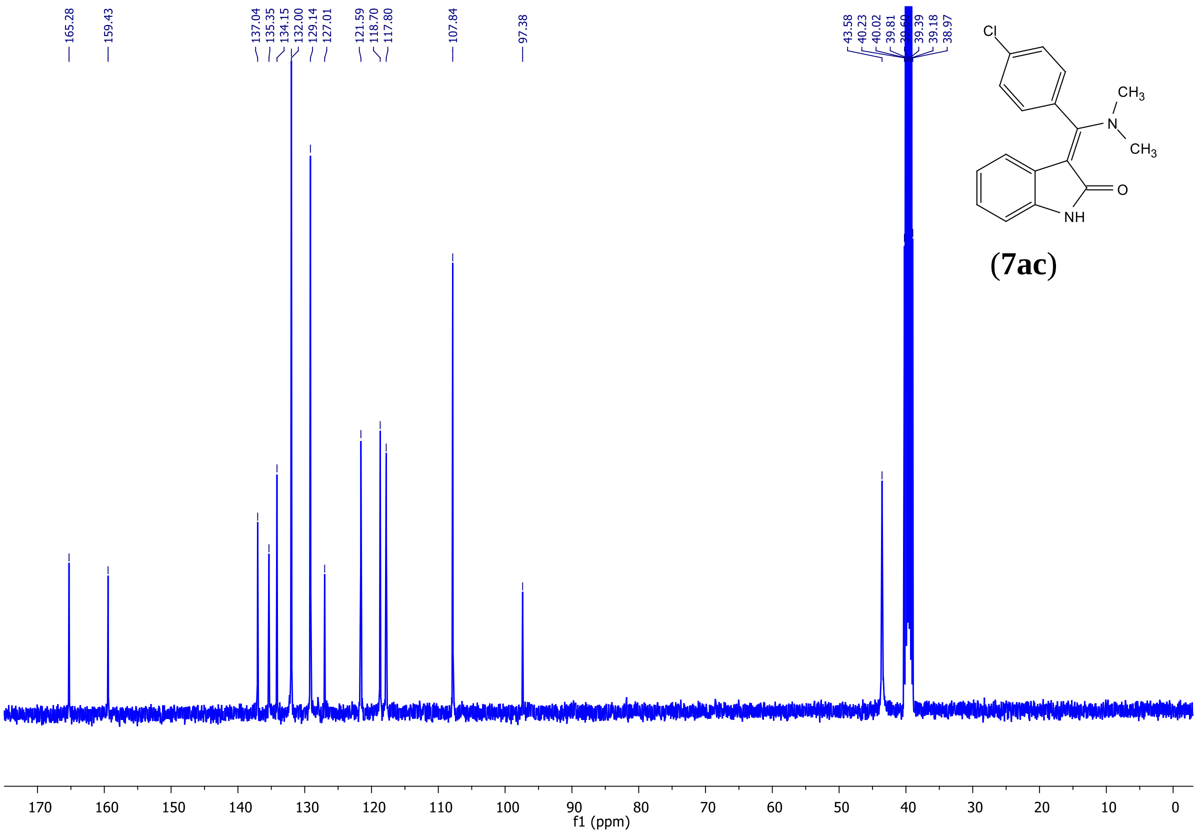
165.28
159.43
137.04
135.35
134.15
132.00
129.14
127.01
121.59
118.70
117.80
107.84
97.38
43.58
40.23
40.02
39.81
39.39
39.18
38.97
f1 (ppm)
Cl
CH3
N
CH3
O
NH
(7ac)

At 60 °C.

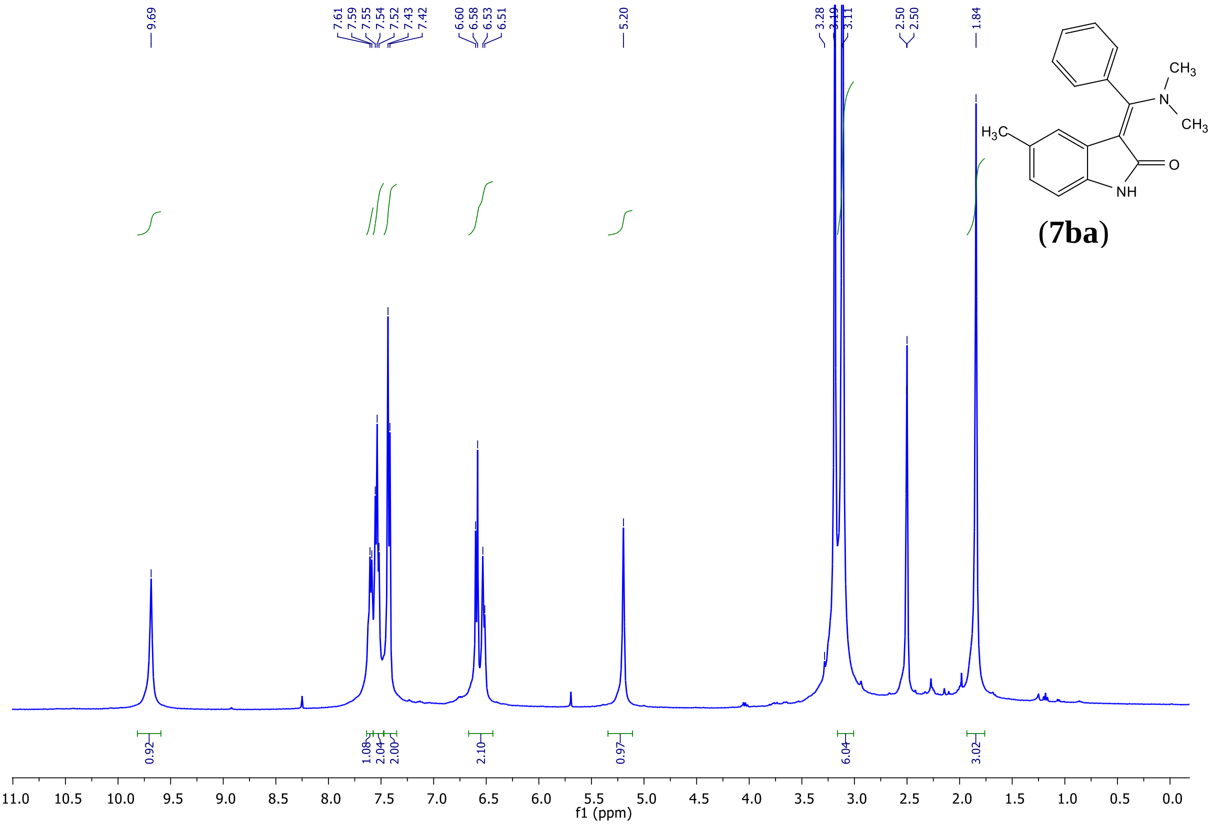

At 60 °C.

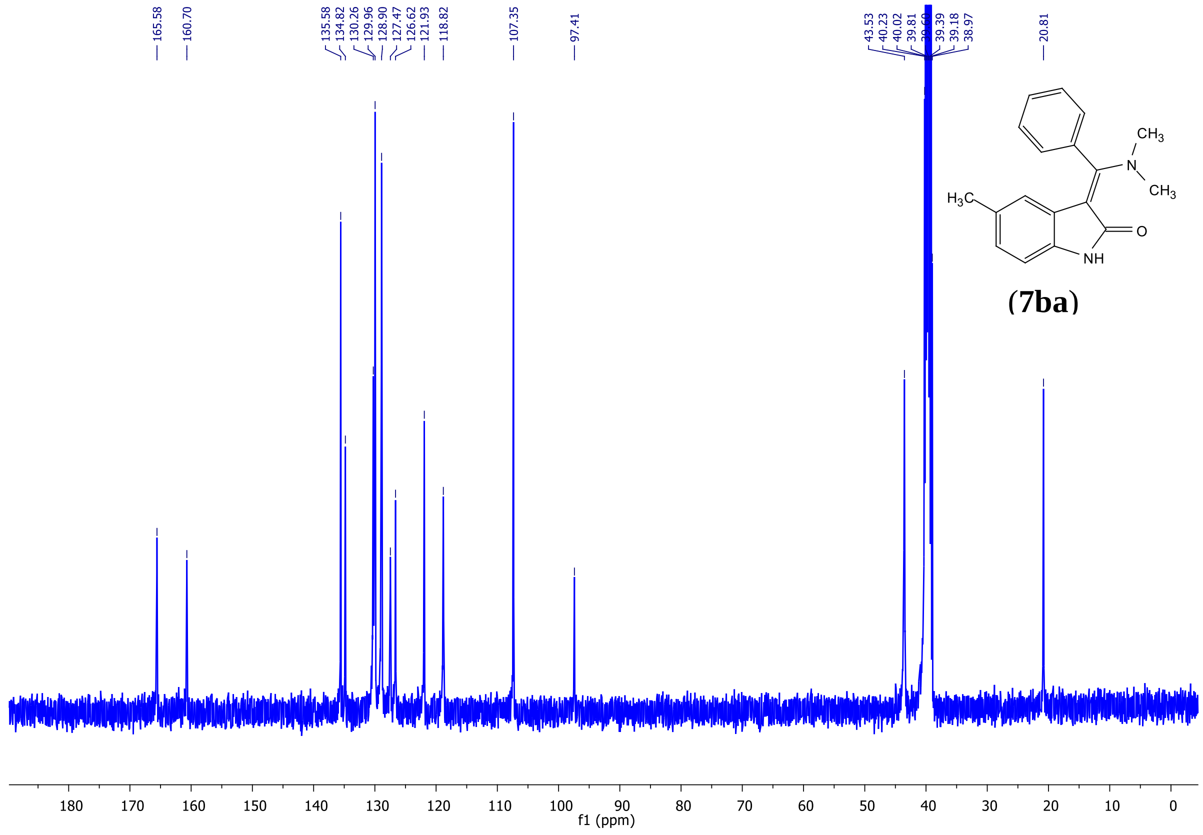

At 60 °C.

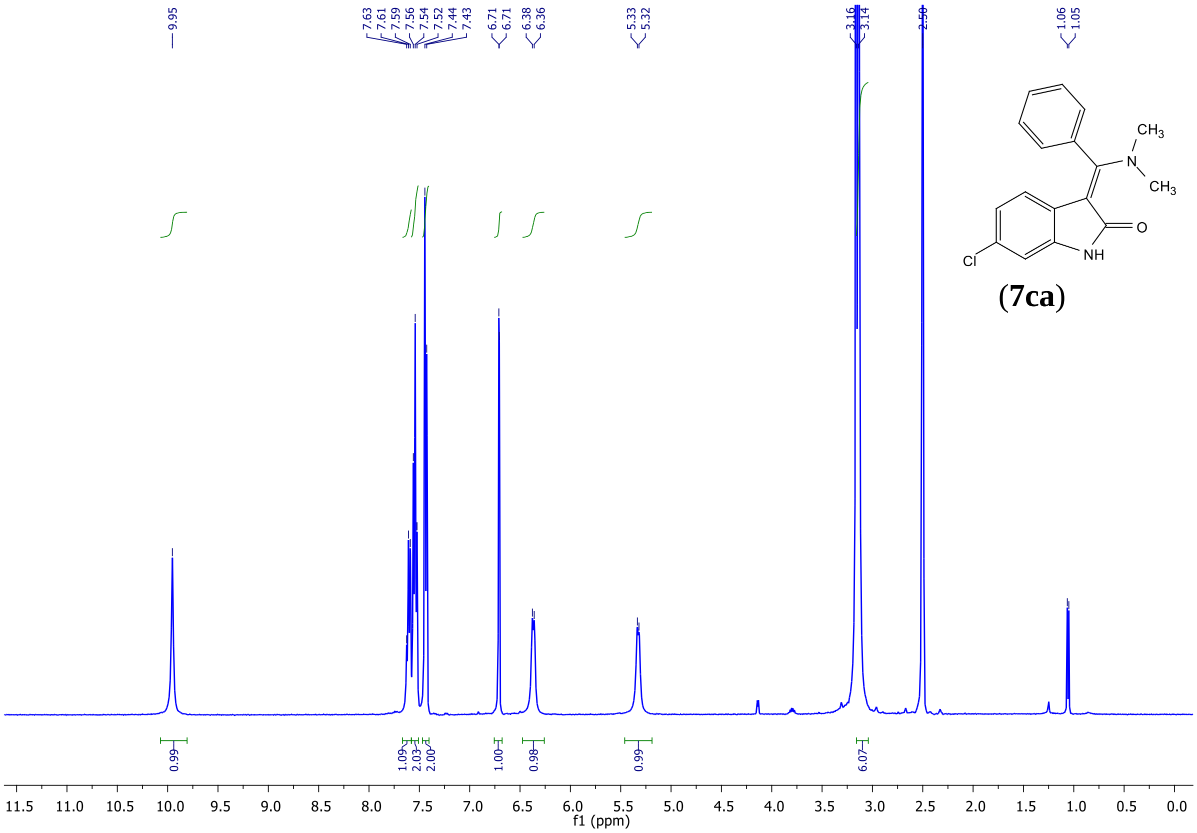
9.95
7.63
7.61
7.59
7.56
7.54
7.52
7.44
7.43
6.71
6.71
6.38
6.36
5.33
5.32
3.16
3.14
2.50
1.06
1.05
0.99
1.09
2.03
2.00
1.00
0.98
0.99
6.07
11.5 11.0 10.5 10.0 9.5 9.0 8.5 8.0 7.5 7.0 6.5 6.0 5.5 5.0 4.5 4.0 3.5 3.0 2.5 2.0 1.5 1.0 0.5 0.0
f1 (ppm)
CH3
N
CH3
O
NH
Cl

(7ca)

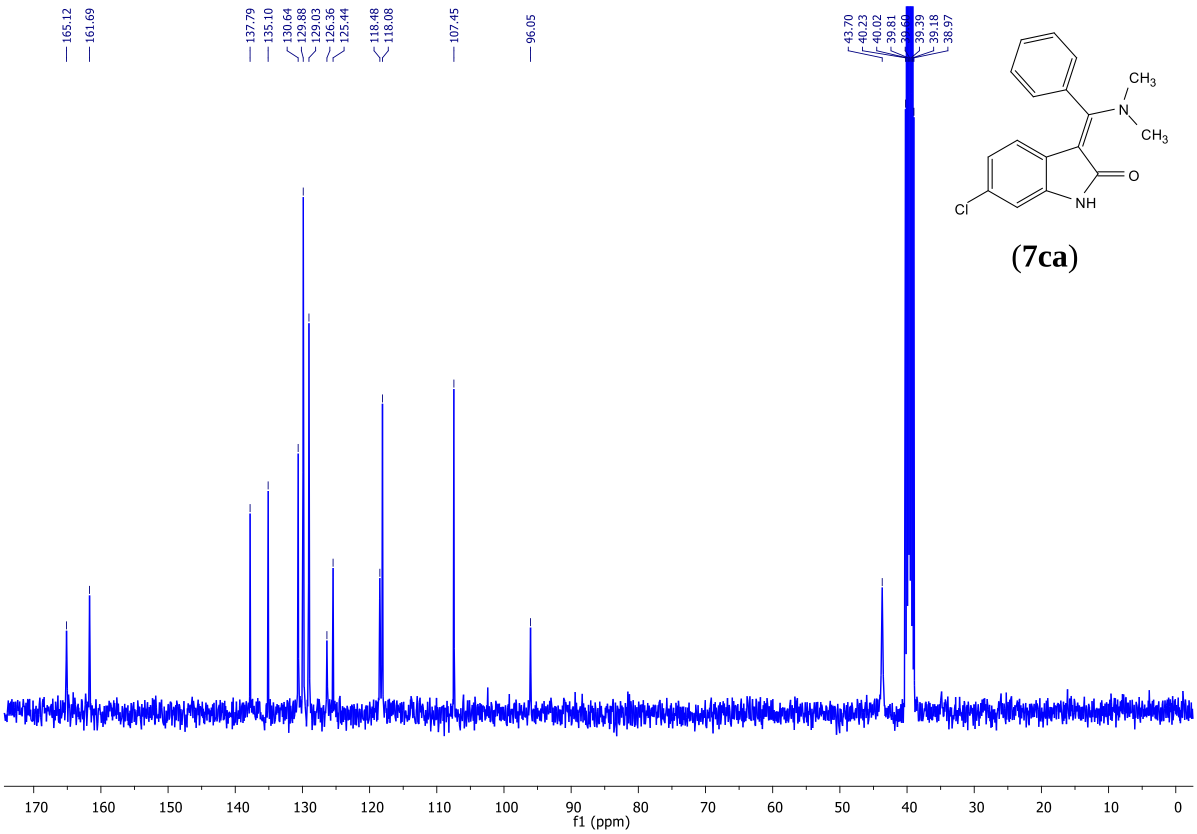
165.12
161.69
137.79
135.10
130.64
129.88
129.03
126.36
125.44
118.48
118.08
107.45
96.05
43.70
40.23
40.02
39.81
39.60
39.39
39.18
38.97
170 160 150 140 130 120 110 100 90 80 70 60 50 40 30 20 10 0
f1 (ppm)
CH3
N
CH3
O
NH
Cl

(7ca)

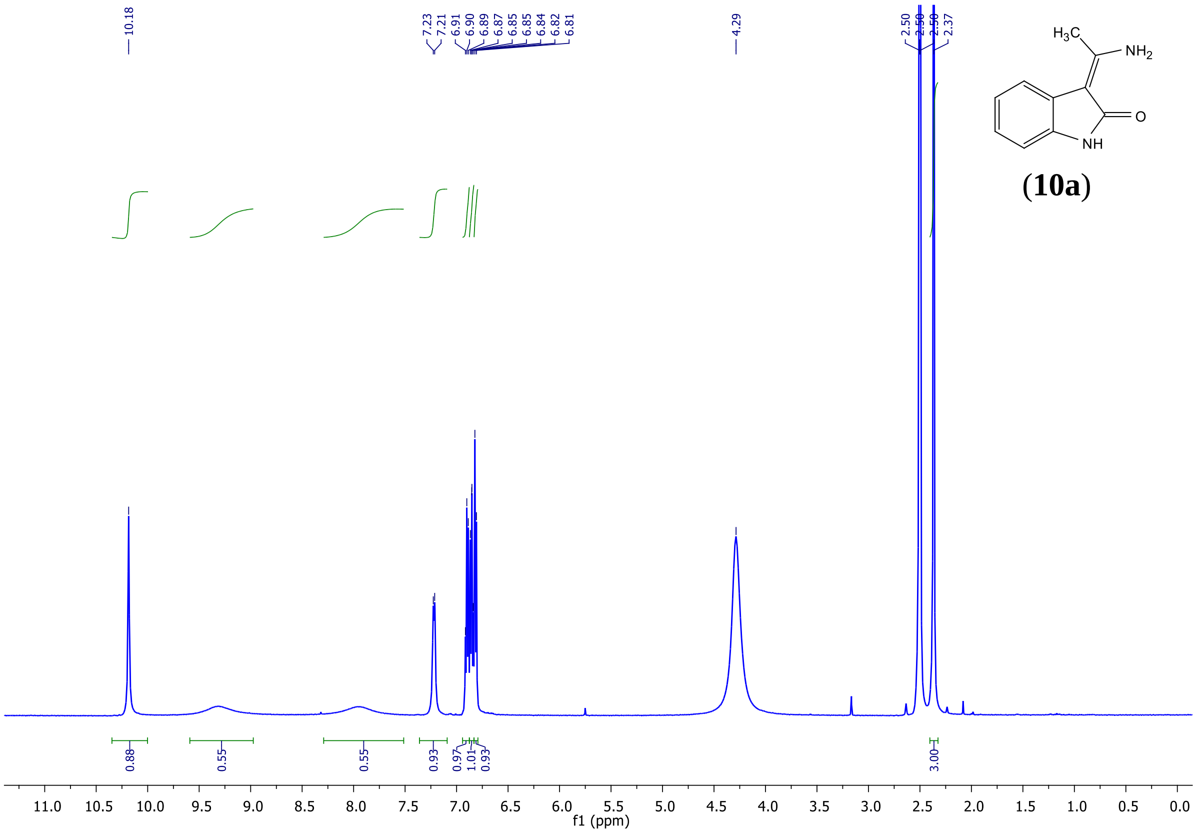
10.18
7.23
7.21
6.91
6.90
6.89
6.87
6.85
6.85
6.84
6.82
6.81
4.29
2.50
2.50
2.50
2.37
H3C
NH2
O
NH
(10a)
0.88
0.55
0.55
0.93
0.97
1.01
0.93
3.00
11.0 10.5 10.0 9.5 9.0 8.5 8.0 7.5 7.0 6.5 6.0 5.5 5.0 4.5 4.0 3.5 3.0 2.5 2.0 1.5 1.0 0.5 0.0
f1 (ppm)

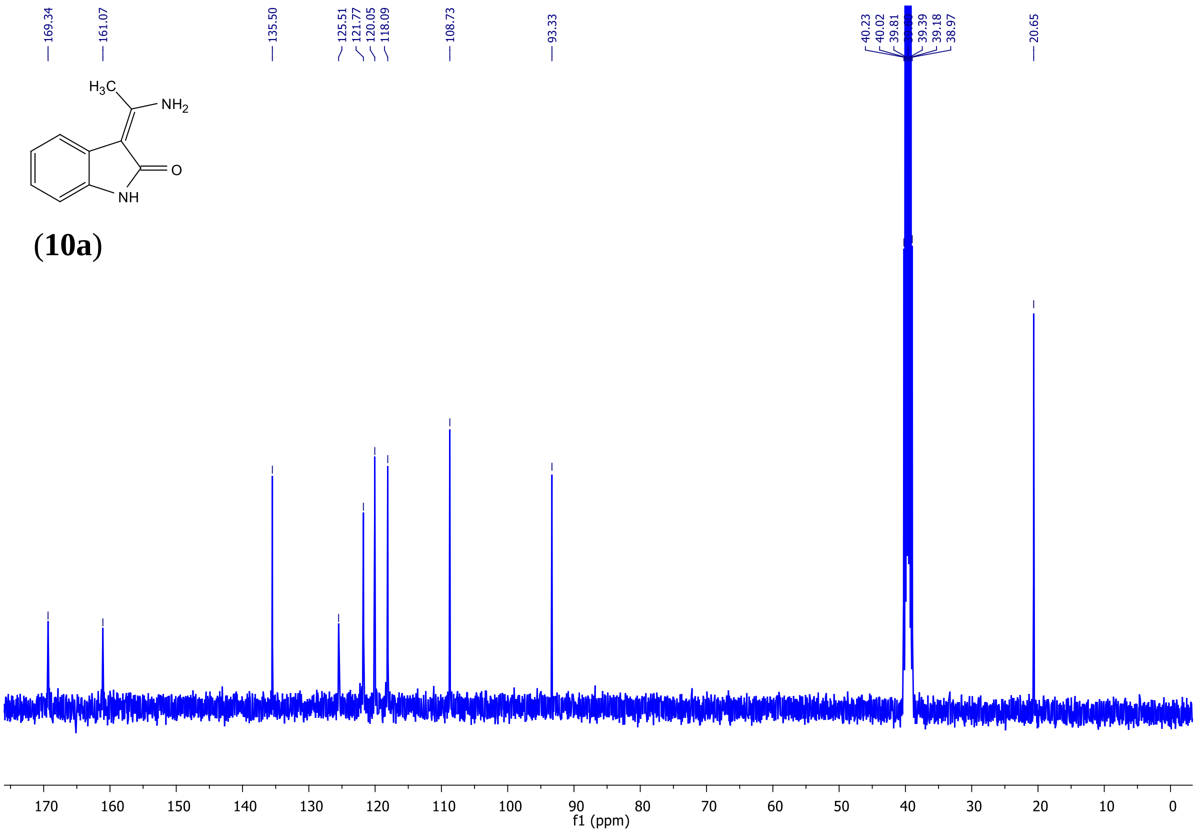
169.34
161.07
135.50
125.51
121.77
120.05
118.09
108.73
93.33
40.23
40.02
39.81
39.39
39.18
38.97
20.65
H3C
NH2
O
NH
(10a)
170 160 150 140 130 120 110 100 90 80 70 60 50 40 30 20 10 0
f1 (ppm)

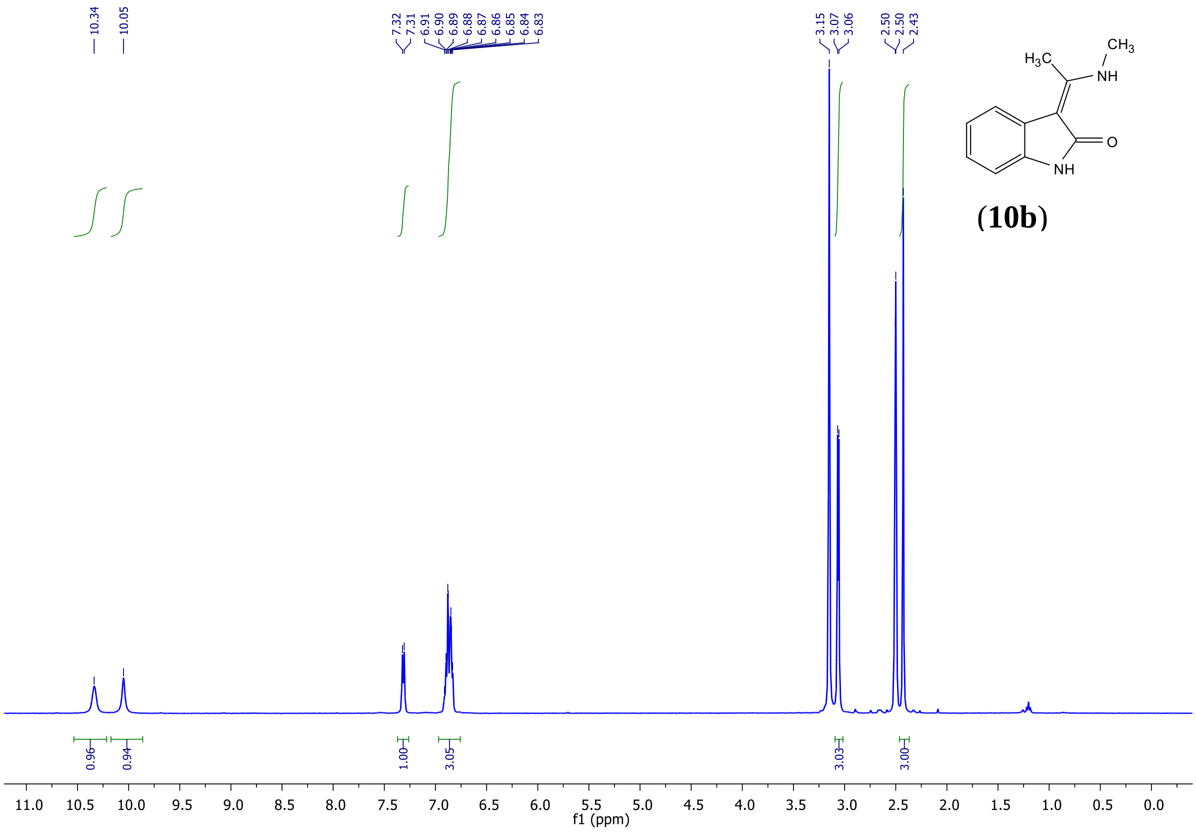

10.34
10.05
7.32
7.31
6.91
6.90
6.89
6.88
6.87
6.86
6.85
6.84
6.83
3.15
3.07
3.06
2.50
2.50
2.43
0.96
0.94
1.00
3.05
3.03
3.00
f1 (ppm)

(10b)

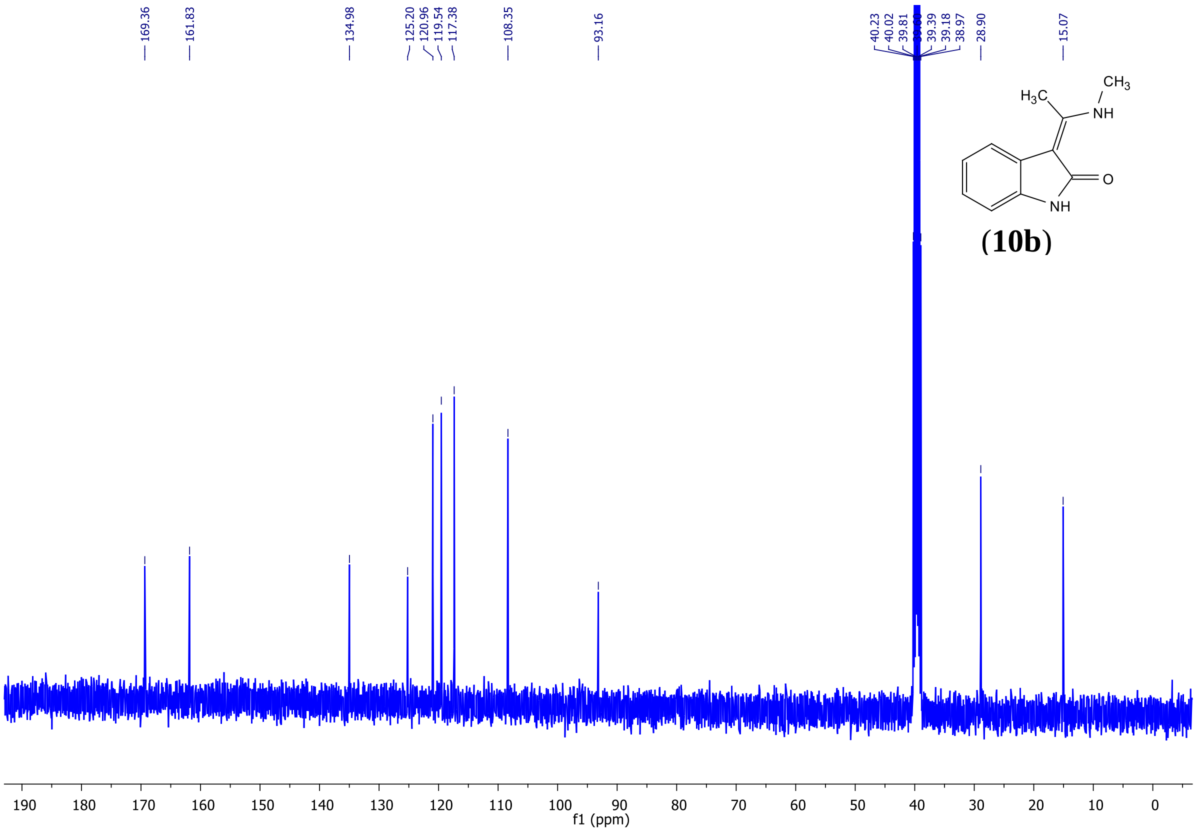

169.36
161.83
134.98
125.20
120.96
119.54
117.38
108.35
93.16
40.23
40.02
39.81
39.39
39.18
38.97
28.90
15.07
f1 (ppm)

(10b)

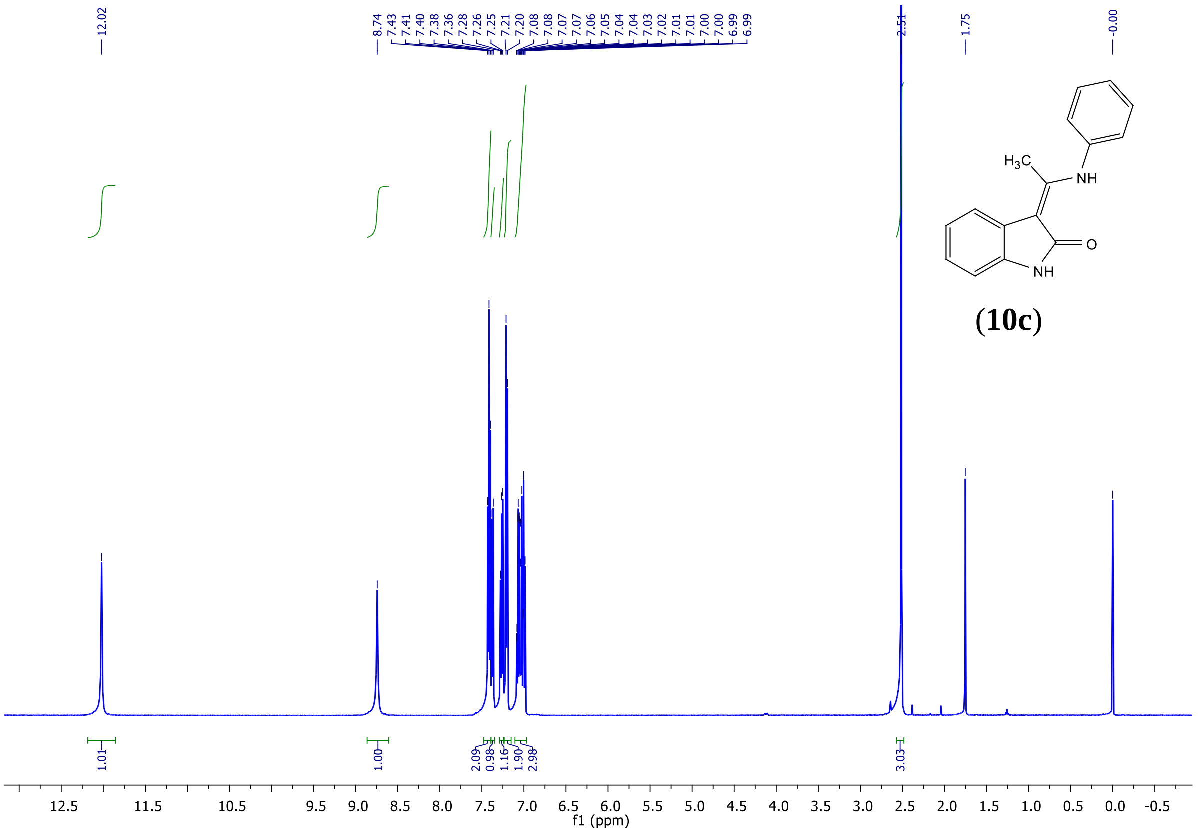

(10c)

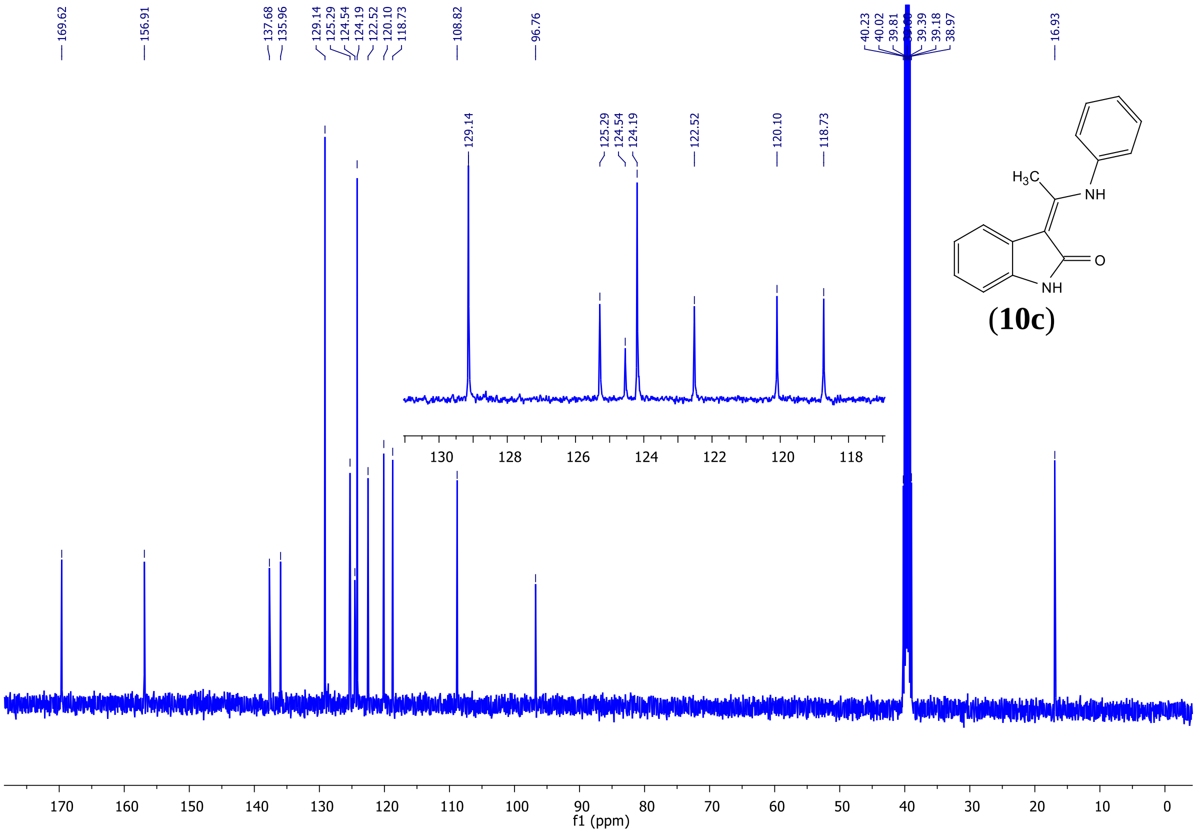

(10c)

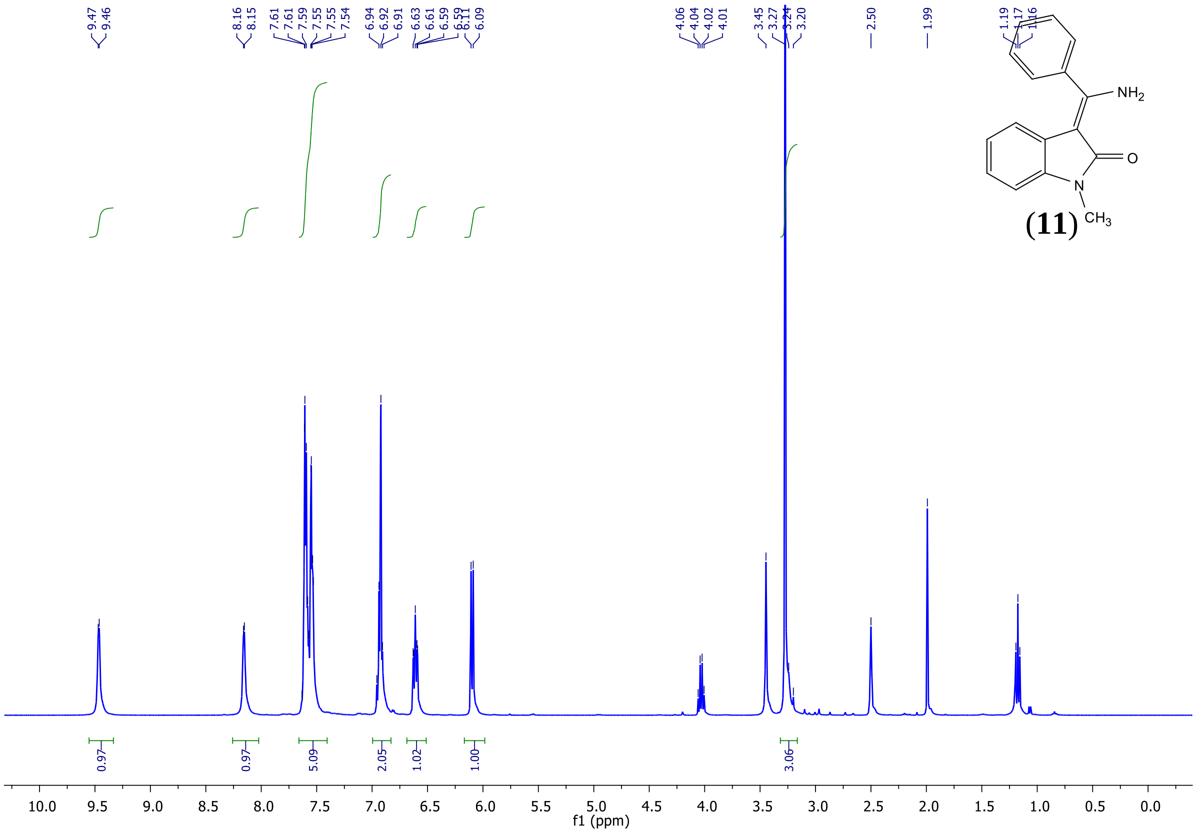

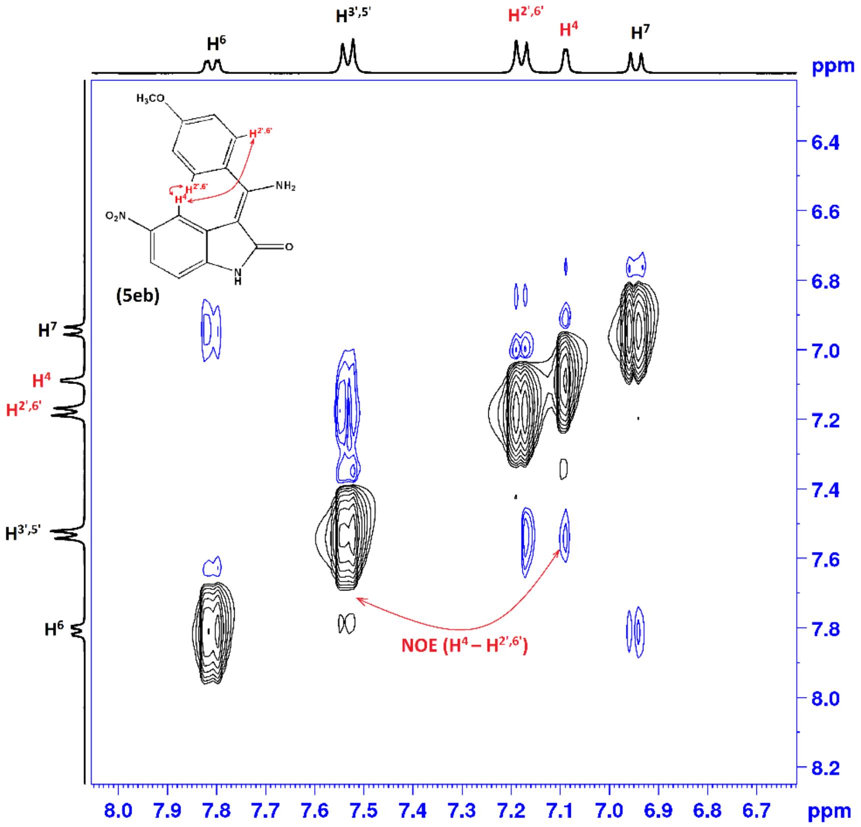

Current Data Parameters
NAME servis
EXPNO 6453
PROCNO 1

F2 - Acquisition Parameters
Date_ 20200924
Time 16.37
INSTRUM spect
PROBHD 5 mm PABBO BB-
PULPROG noesyph
TD 2048
SOLVENT DMSO
NS 128
DS 16
SWH 3780.242 Hz
FIDRES 1.845821 Hz
AQ 0.2708821 sec
RG 64
DW 132.267 usec
DE 6.50 usec
TE 297.9 K
D0 0.00011947 sec
D1 2.00000000 sec
D8 0.69999999 sec
IN0 0.00026440 sec

======== CHANNEL f1 ========
SFO1 400.1327130 MHz
NUC1 1H
P1 10.00 usec
PLW1 26.98999977 W

F1 - Acquisition parameters
TD 128
SFO1 400.1327 MHz
FIDRES 59.036066 Hz
SW 9.452 ppm
FnMODE States-TPPI

F2 - Processing parameters
SI 1024
SF 400.1300033 MHz
WDW QSINE
SSB 2
LB 0 Hz
GB 0
PC 1.00

F1 - Processing parameters
SI 1024
MC2 States-TPPI
SF 400.1300033 MHz
WDW QSINE
SSB 2
LB 0 Hz
GB 0

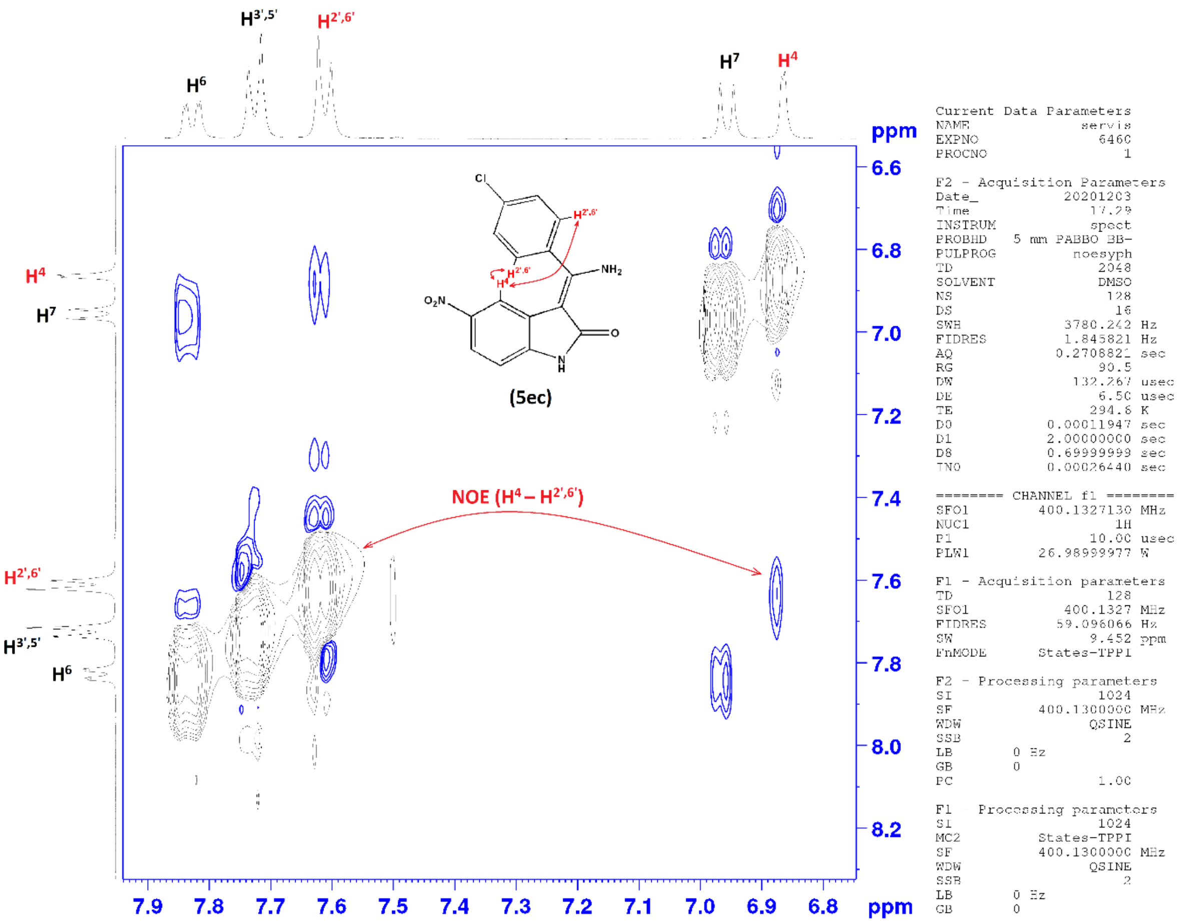
(5ec)
NOE (H4 – H2',6')
Current Data Parameters
NAME servis
EXPNO 6460
PROCNO 1
F2 - Acquisition Parameters
Date_ 20201203
Time 17.29
INSTRUM spect
PROBHD 5 mm PABBO BB-
PULPROG noesyph
TD 2048
SOLVENT DMSO
NS 128
DS 16
SWH 3780.242 Hz
FIDRES 1.845821 Hz
AQ 0.2708821 sec
RG 90.5
DW 132.267 usec
DE 6.50 usec
TE 294.8 K
D0 0.00011947 sec
D1 2.00000000 sec
D8 0.69999999 sec
IN0 0.00026440 sec
======== CHANNEL f1 ========
SFO1 400.1327130 MHz
NUC1 1H
P1 10.00 usec
PLW1 26.98999977 W
F1 - Acquisition parameters
TD 128
SFO1 400.1327 MHz
FIDRES 59.096066 Hz
SW 9.452 ppm
FnMODE States-TPPI
F2 - Processing parameters
SI 1024
SF 400.1300000 MHz
WDW QSINE
SSB 2
LB 0 Hz
GB 0
PC 1.00
F1 - Processing parameters
SI 1024
MC2 States-TPPI
SF 400.1300000 MHz
WDW QSINE
SSB 2
LB 0 Hz
GB 0
ppm

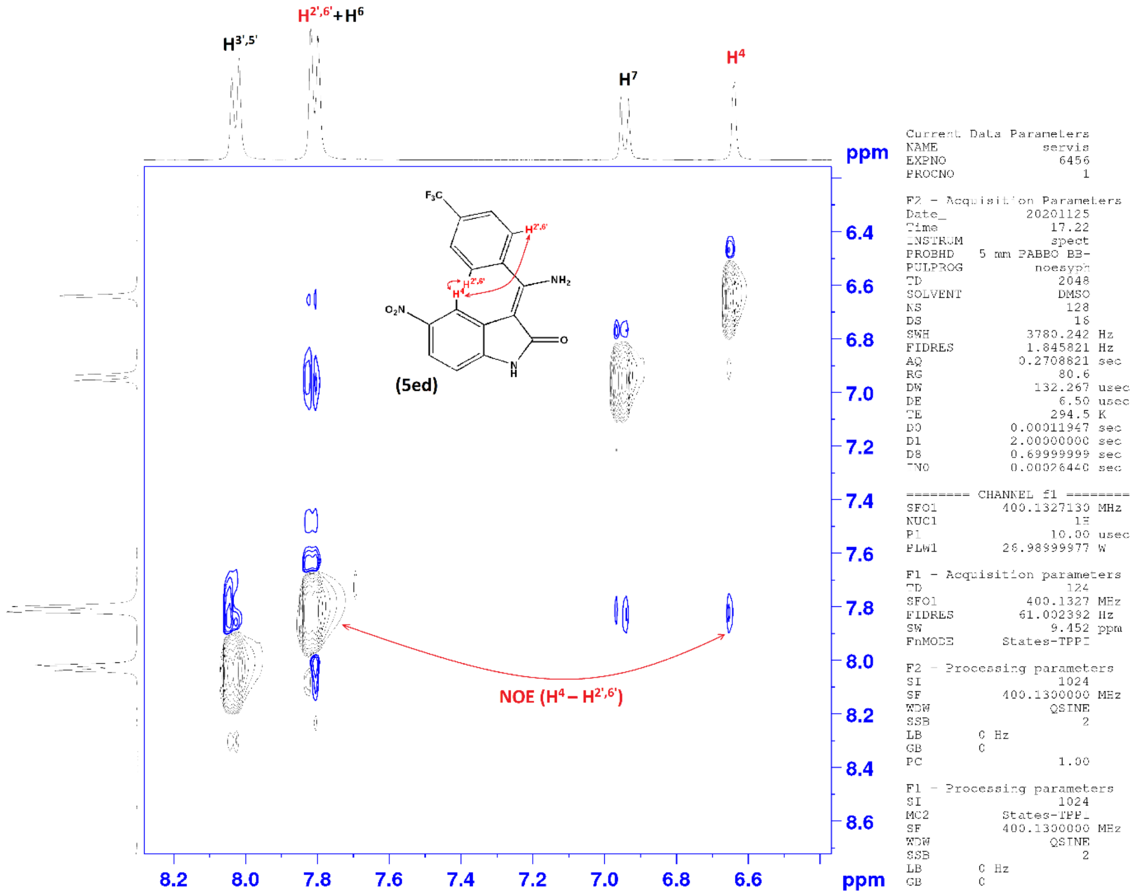
(5ed)
NOE (H4 – H2',6')
Current Data Parameters
NAME servis
EXPNO 6456
PROCNO 1
F2 - Acquisition Parameters
Date_ 20201125
Time 17.22
INSTRUM spect
PROBHD 5 mm PABBO BB-
PULPROG noesyph
TD 2048
SOLVENT DMSO
NS 128
DS 16
SWH 3780.242 Hz
FIDRES 1.845821 Hz
AQ 0.2708821 sec
RG 80.6
DW 132.267 usec
DE 6.50 usec
TE 294.5 K
D0 0.00011947 sec
D1 2.00000000 sec
D8 0.69999999 sec
IN0 0.00026440 sec
======== CHANNEL f1 ========
SFO1 400.1327130 MHz
NUC1 1H
P1 10.00 usec
PLW1 26.98999977 W
F1 - Acquisition parameters
TD 124
SFO1 400.1327 MHz
FIDRES 61.002392 Hz
SW 9.452 ppm
FnMODE States-TPPI
F2 - Processing parameters
SI 1024
SF 400.1300000 MHz
WDW QSINE
SSB 2
LB 0 Hz
GB 0
PC 1.00
F1 - Processing parameters
SI 1024
MC2 States-TPPI
SF 400.1300000 MHz
WDW QSINE
SSB 2
LB 0 Hz
GB 0
ppm

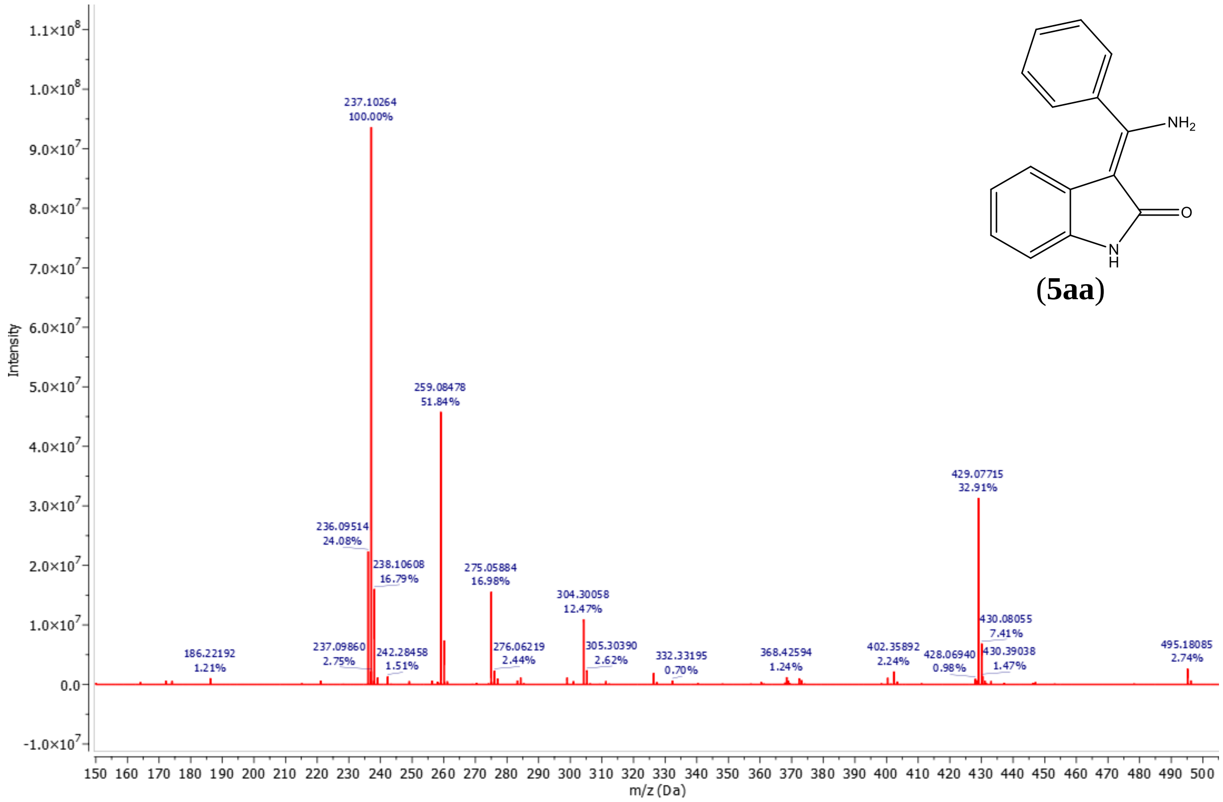
NH2
O
N
H
237.10264
100.00%
259.08478
51.84%
429.07715
32.91%
Intensity
m/z (Da)

(5aa)

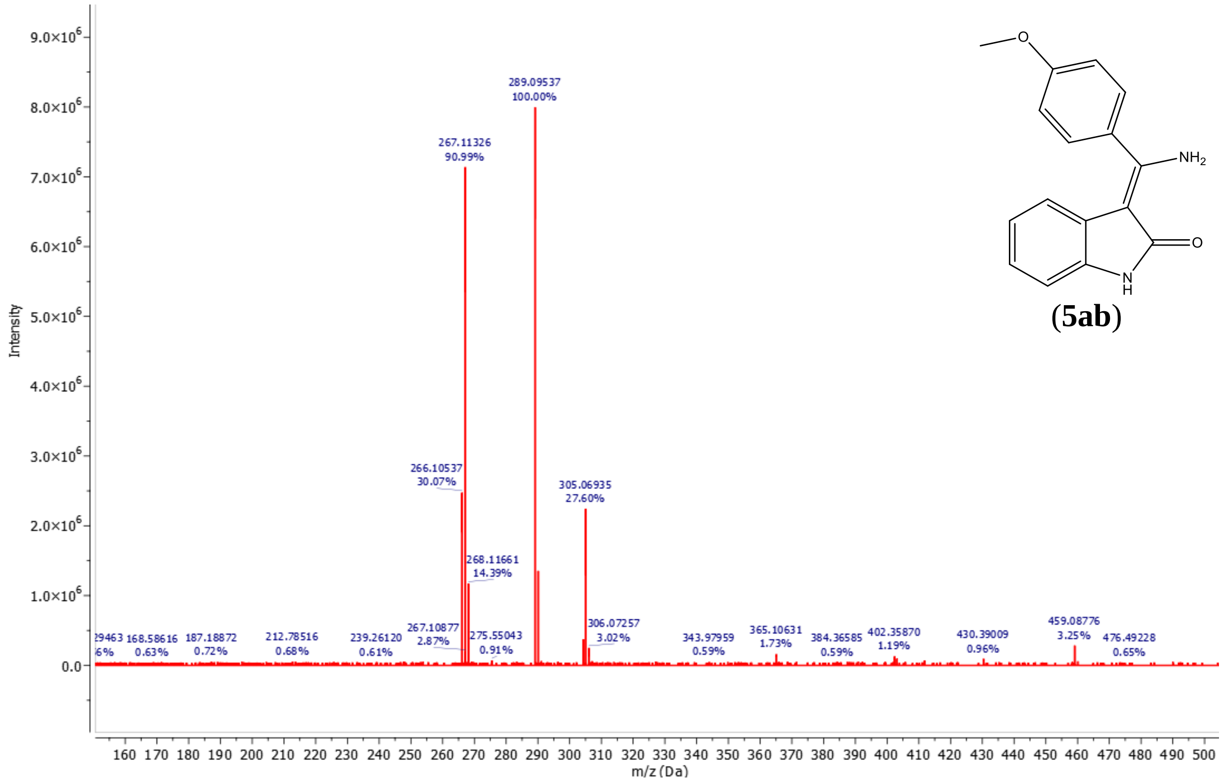
O
NH2
O
N
H
289.09537
100.00%
267.11326
90.99%
305.06935
27.60%
Intensity
m/z (Da)

(5ab)

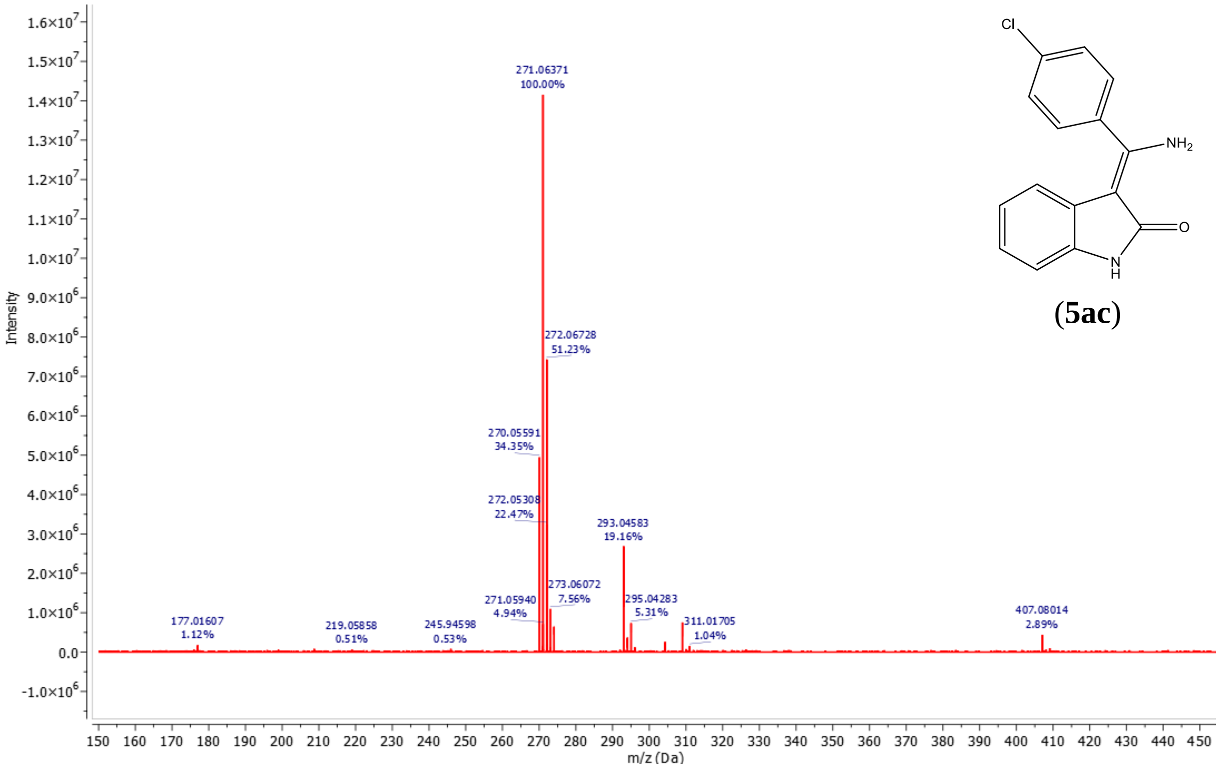
Intensity
m/z (Da)
271.06371
100.00%
272.06728
51.23%
270.05591
34.35%
272.05308
22.47%
293.04583
19.16%
273.06072
7.56%
271.05940
4.94%
295.04283
5.31%
311.01705
1.04%
177.01607
1.12%
219.05858
0.51%
245.94598
0.53%
407.08014
2.89%
Cl
NH2
O
N
H

(5ac)

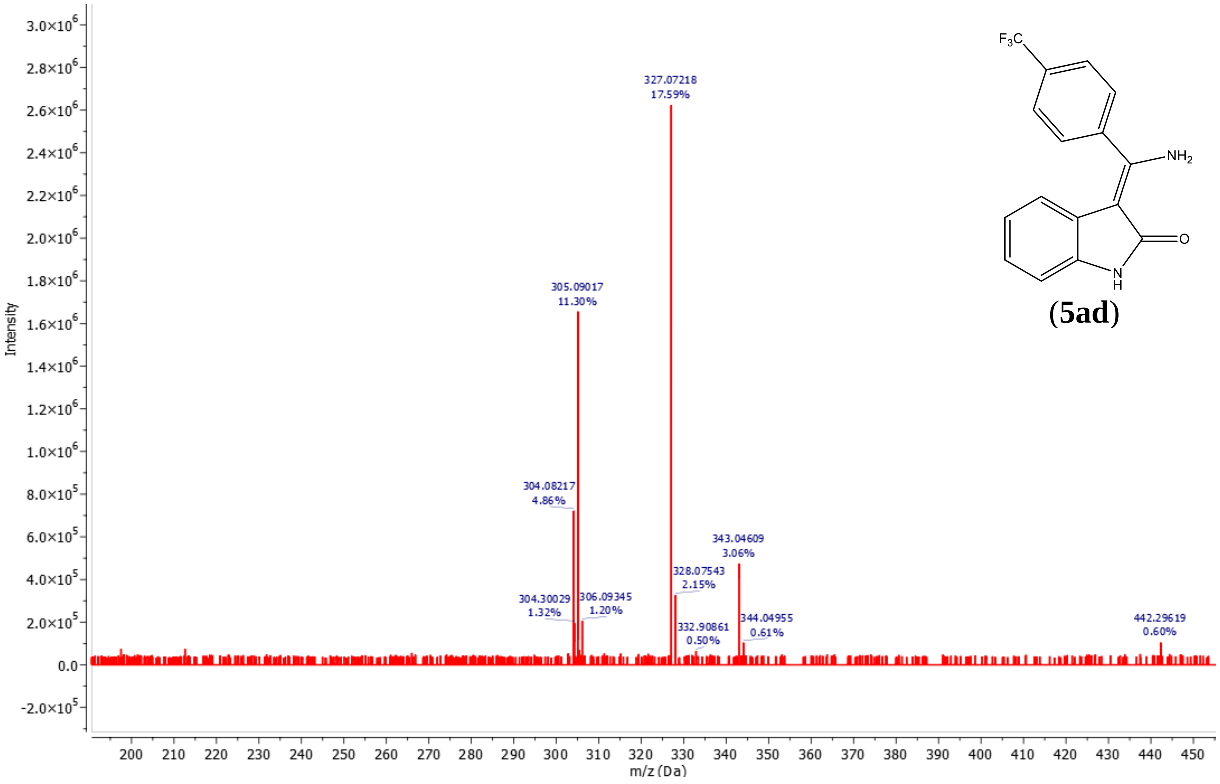
Intensity
m/z (Da)
327.07218
17.59%
305.09017
11.30%
304.08217
4.86%
343.04609
3.06%
328.07543
2.15%
304.30029
1.32%
306.09345
1.20%
332.90861
0.50%
344.04955
0.61%
442.29619
0.60%
F3C
NH2
O
N
H

(5ad)

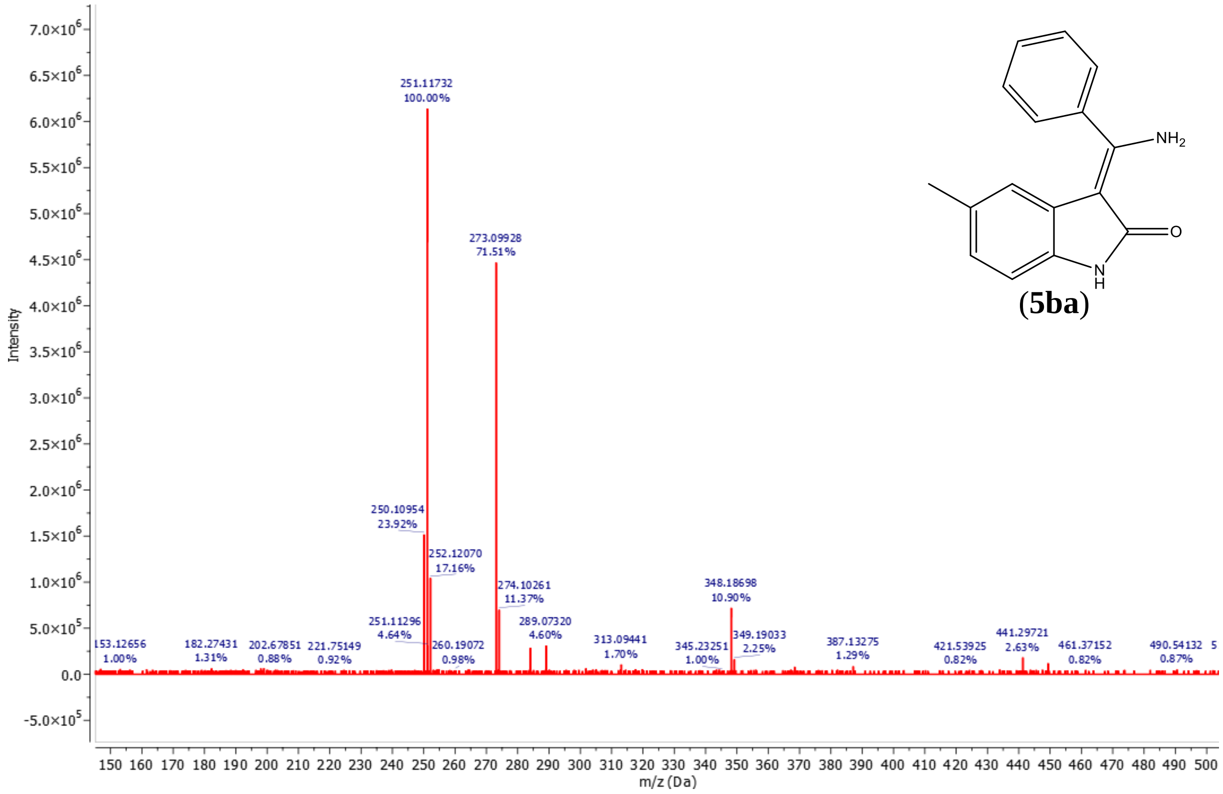
Intensity
m/z (Da)
251.11732
100.00%
273.09928
71.51%
250.10954
23.92%
252.12070
17.16%
274.10261
11.37%
348.18698
10.90%
251.11296
4.64%
289.07320
4.60%
NH2
O
N
H
(5ba)

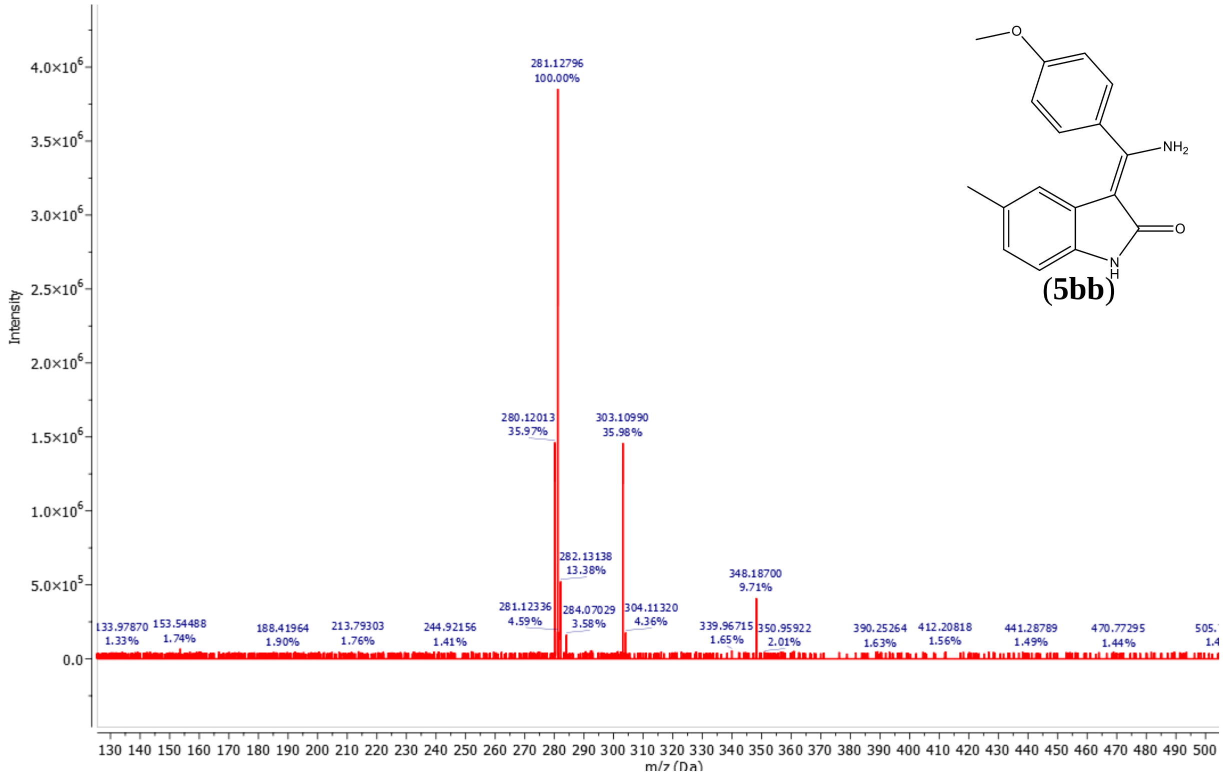
Intensity
m/z (Da)
281.12796
100.00%
280.12013
35.97%
303.10990
35.98%
282.13138
13.38%
348.18700
9.71%
281.12336
4.59%
284.07029
3.58%
304.11320
4.36%
O
NH2
O
N
H
(5bb)

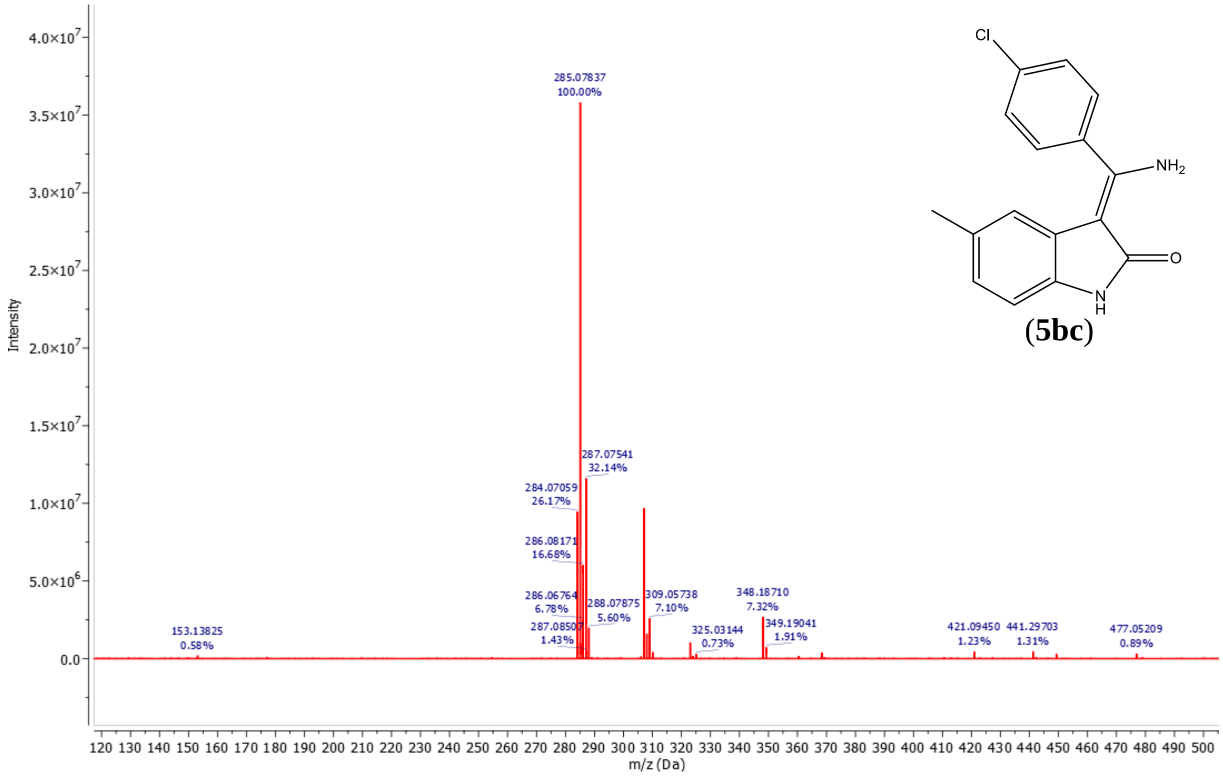
Intensity
m/z (Da)
285.07837 100.00%
287.07541 32.14%
284.07059 26.17%
286.08171 16.68%
286.06764 6.78%
287.08507 1.43%
288.07875 5.60%
309.05738 7.10%
325.03144 0.73%
348.18710 7.32%
349.19041 1.91%
153.13825 0.58%
421.09450 1.23%
441.29703 1.31%
477.05209 0.89%
Cl
NH2
O
N
H

(5bc)

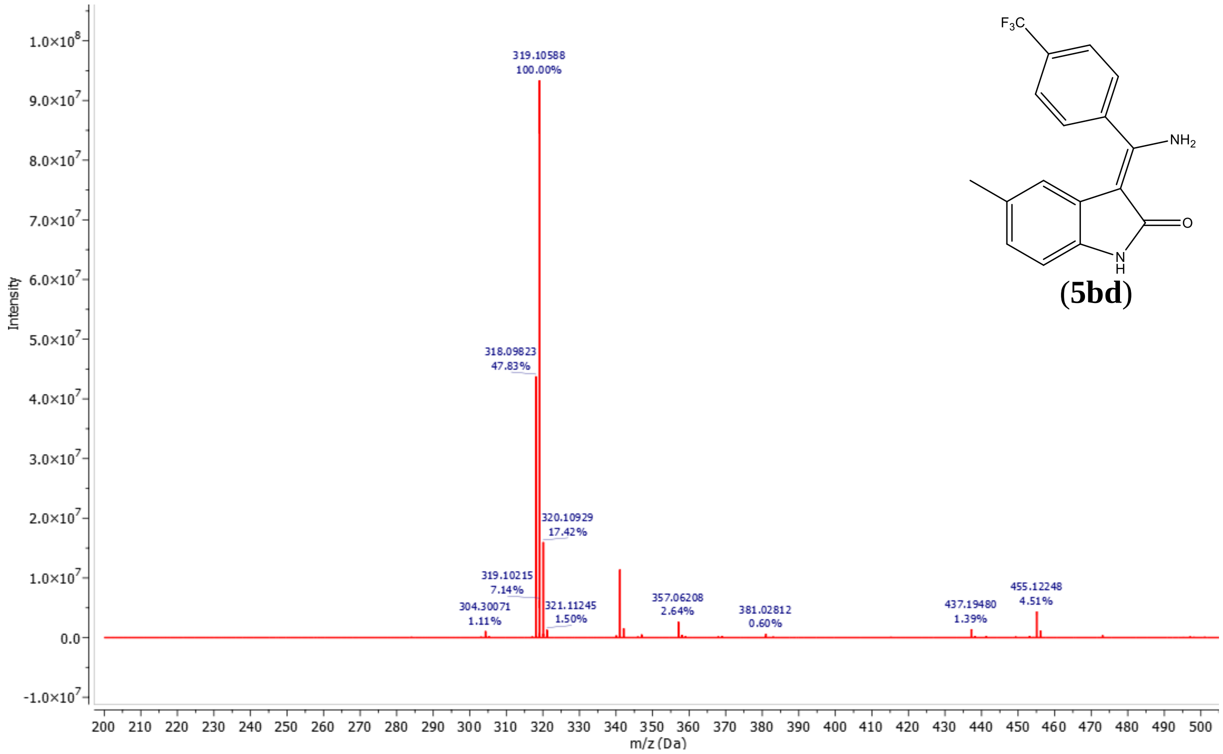
Intensity
m/z (Da)
319.10588 100.00%
318.09823 47.83%
320.10929 17.42%
319.10215 7.14%
304.30071 1.11%
321.11245 1.50%
357.06208 2.64%
381.02812 0.60%
437.19480 1.39%
455.12248 4.51%
F3C
NH2
O
N
H

(5bd)

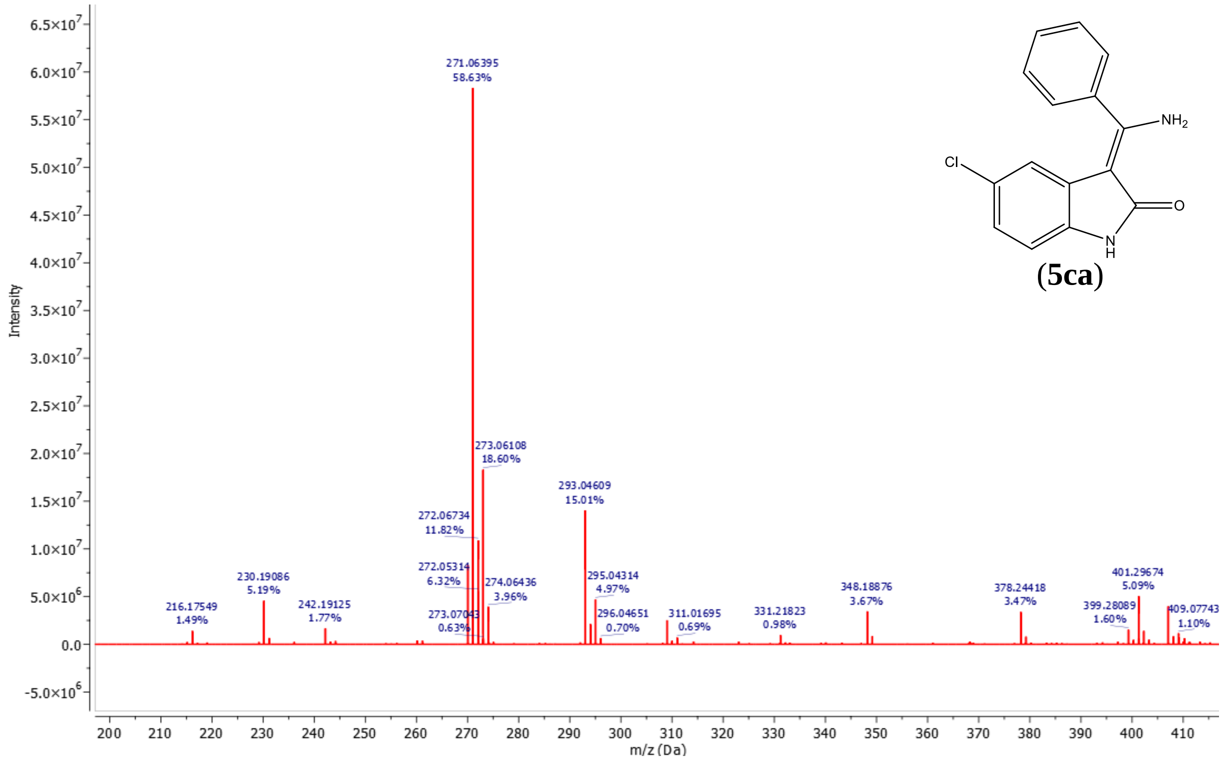

Intensity
m/z (Da)
271.06395
58.63%
273.06108
18.60%
293.04609
15.01%
272.06734
11.82%
272.05314
6.32%
274.06436
3.96%
295.04314
4.97%
296.04651
0.70%
273.07043
0.63%
230.19086
5.19%
216.17549
1.49%
242.19125
1.77%
311.01695
0.69%
331.21823
0.98%
348.18876
3.67%
378.24418
3.47%
401.29674
5.09%
399.28089
1.60%
409.07743
1.10%
Cl
NH2
O
N
H

(5ca)

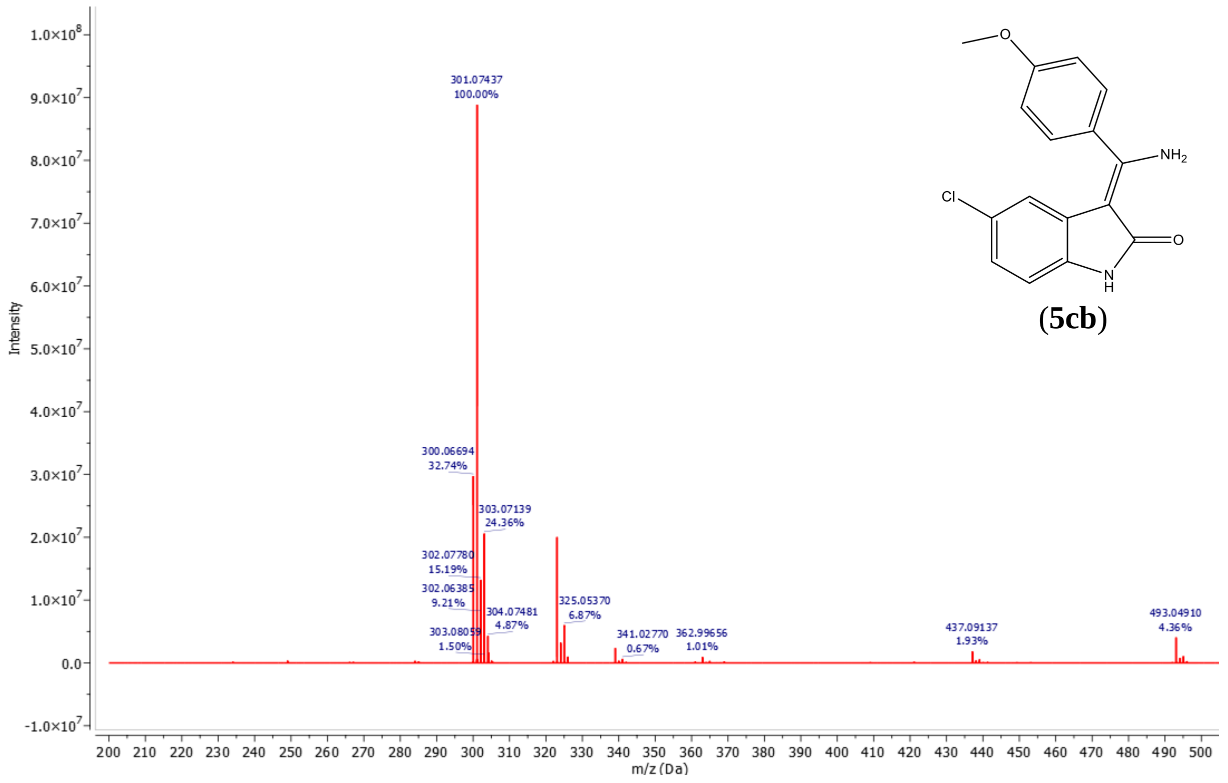

Intensity
m/z (Da)
301.07437
100.00%
300.06694
32.74%
303.07139
24.36%
302.07780
15.19%
302.06385
9.21%
304.07481
4.87%
303.08059
1.50%
325.05370
6.87%
341.02770
0.67%
362.99656
1.01%
437.09137
1.93%
493.04910
4.36%
O
Cl
NH2
O
N
H

(5cb)

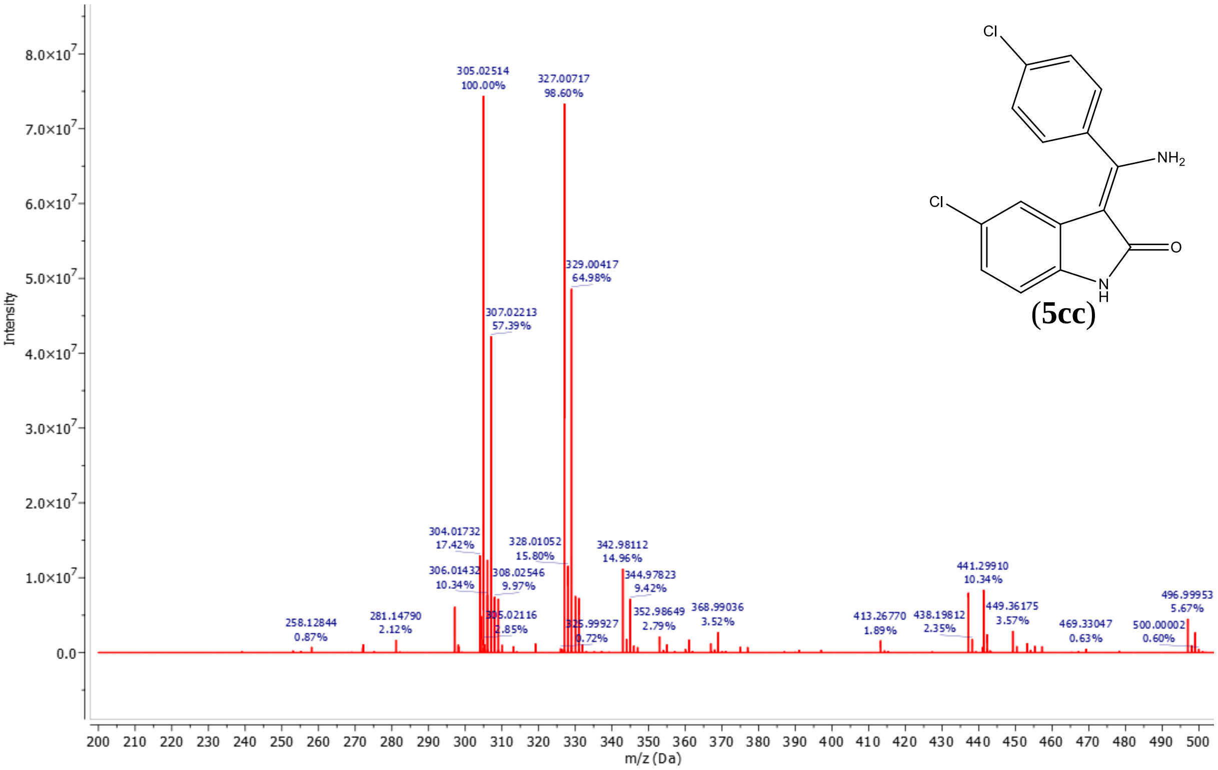

305.02514
100.00%
327.00717
98.60%
329.00417
64.98%
307.02213
57.39%
304.01732
17.42%
306.01432
10.34%
308.02546
9.97%
305.02116
2.85%
328.01052
15.80%
325.99927
0.72%
342.98112
14.96%
344.97823
9.42%
352.98649
2.79%
368.99036
3.52%
258.12844
0.87%
281.14790
2.12%
413.26770
1.89%
438.19812
2.35%
441.29910
10.34%
449.36175
3.57%
469.33047
0.63%
496.99953
5.67%
500.00002
0.60%
Intensity
m/z (Da)
Cl
NH2
Cl
O
N
H
(5cc)

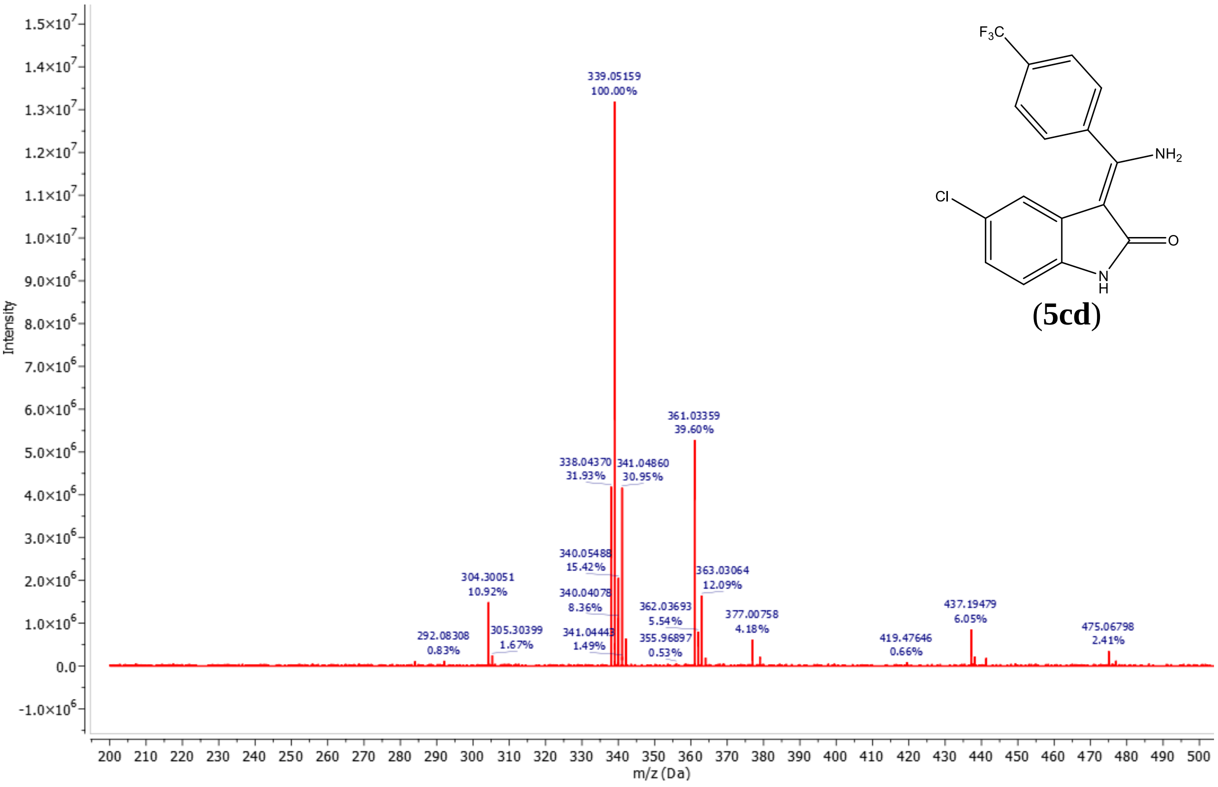

339.05159
100.00%
361.03359
39.60%
338.04370
31.93%
341.04860
30.95%
340.05488
15.42%
340.04078
8.36%
341.04443
1.49%
304.30051
10.92%
305.30399
1.67%
292.08308
0.83%
363.03064
12.09%
362.03693
5.54%
355.96897
0.53%
377.00758
4.18%
419.47646
0.66%
437.19479
6.05%
475.06798
2.41%
Intensity
m/z (Da)
F3C
NH2
Cl
O
N
H
(5cd)

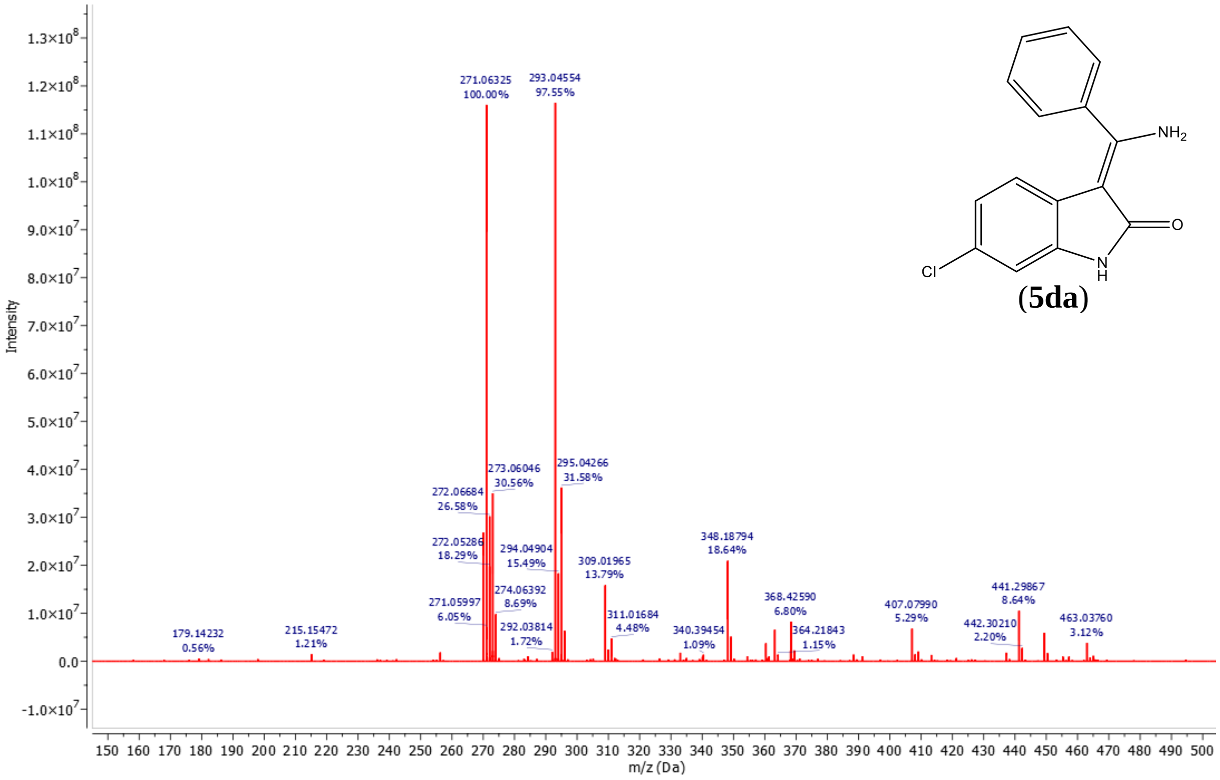
Intensity
m/z (Da)
271.06325
100.00%
293.04554
97.55%
NH2
O
Cl
N
H

(5da)

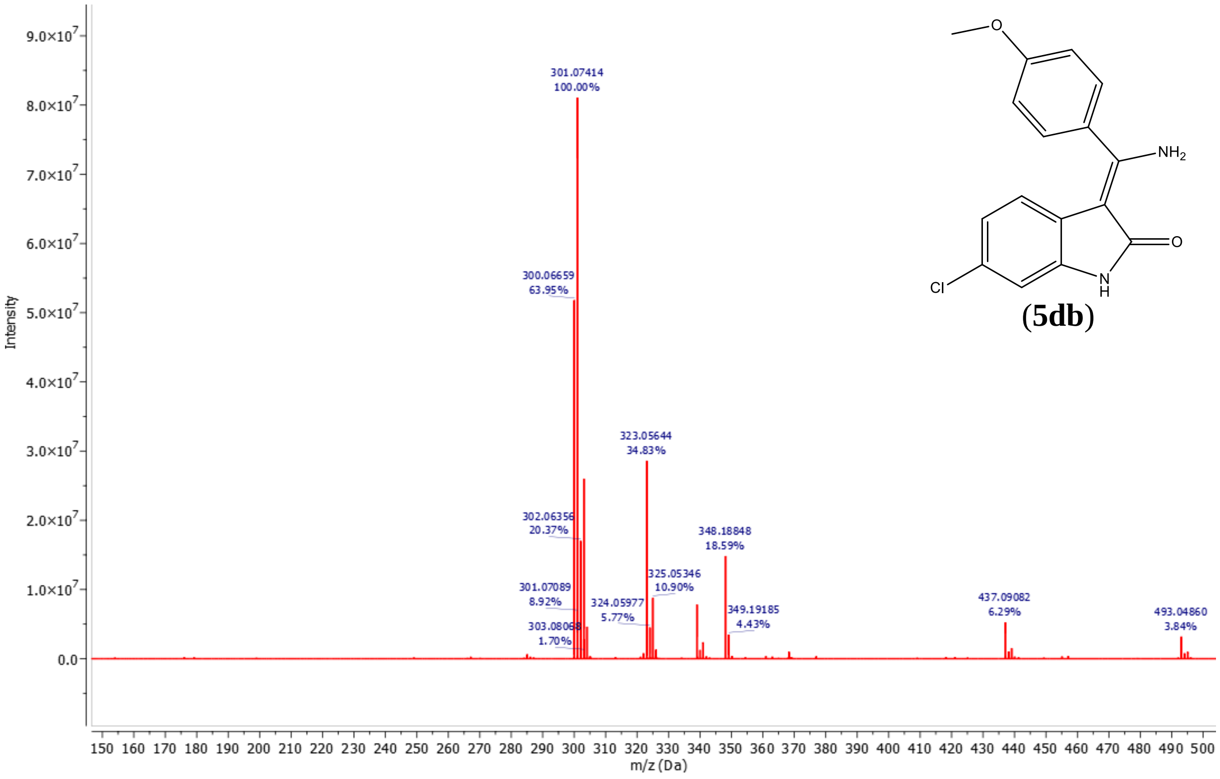
Intensity
m/z (Da)
301.07414
100.00%
O
NH2
O
Cl
N
H

(5db)

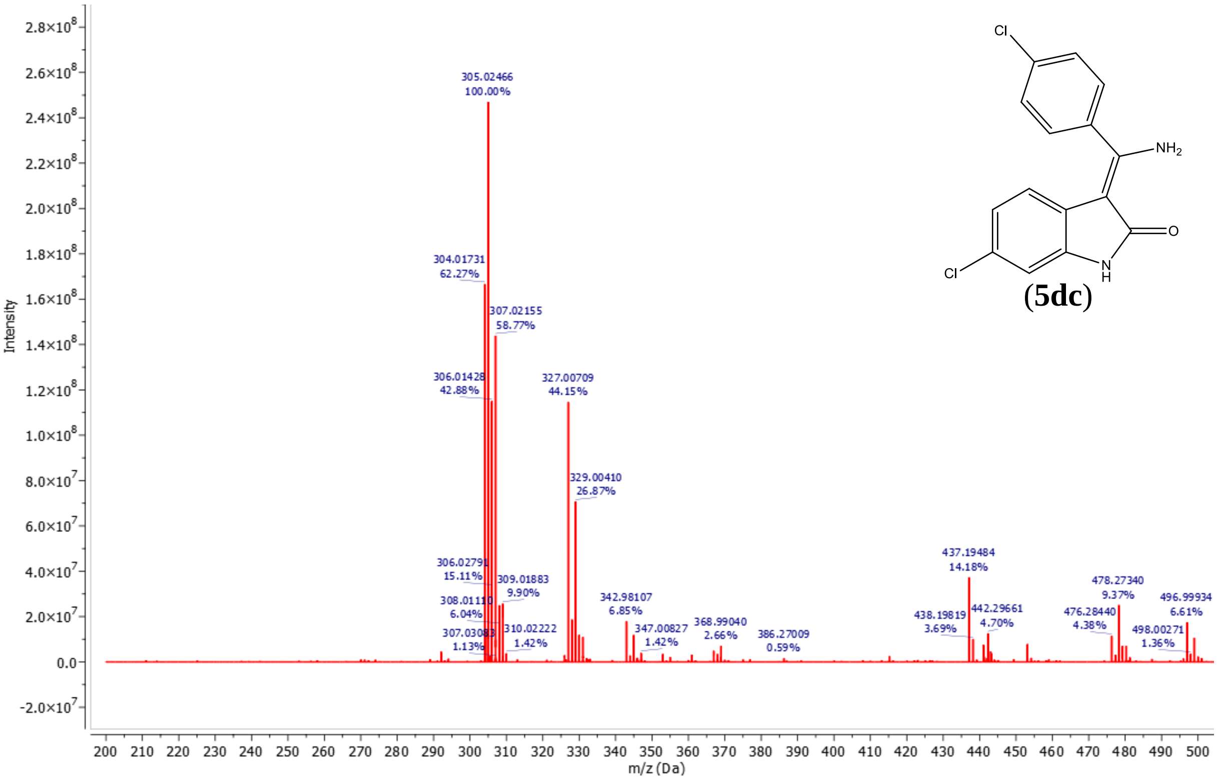

(5dc)

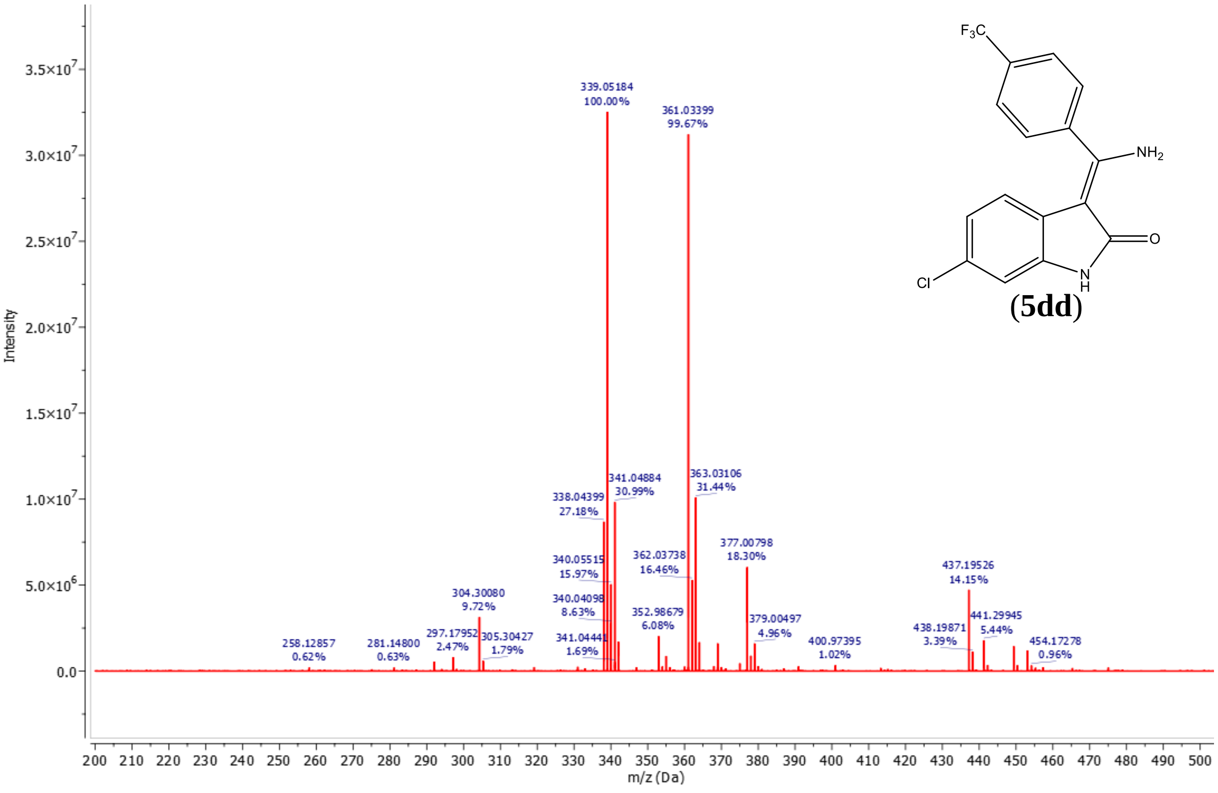

(5dd)

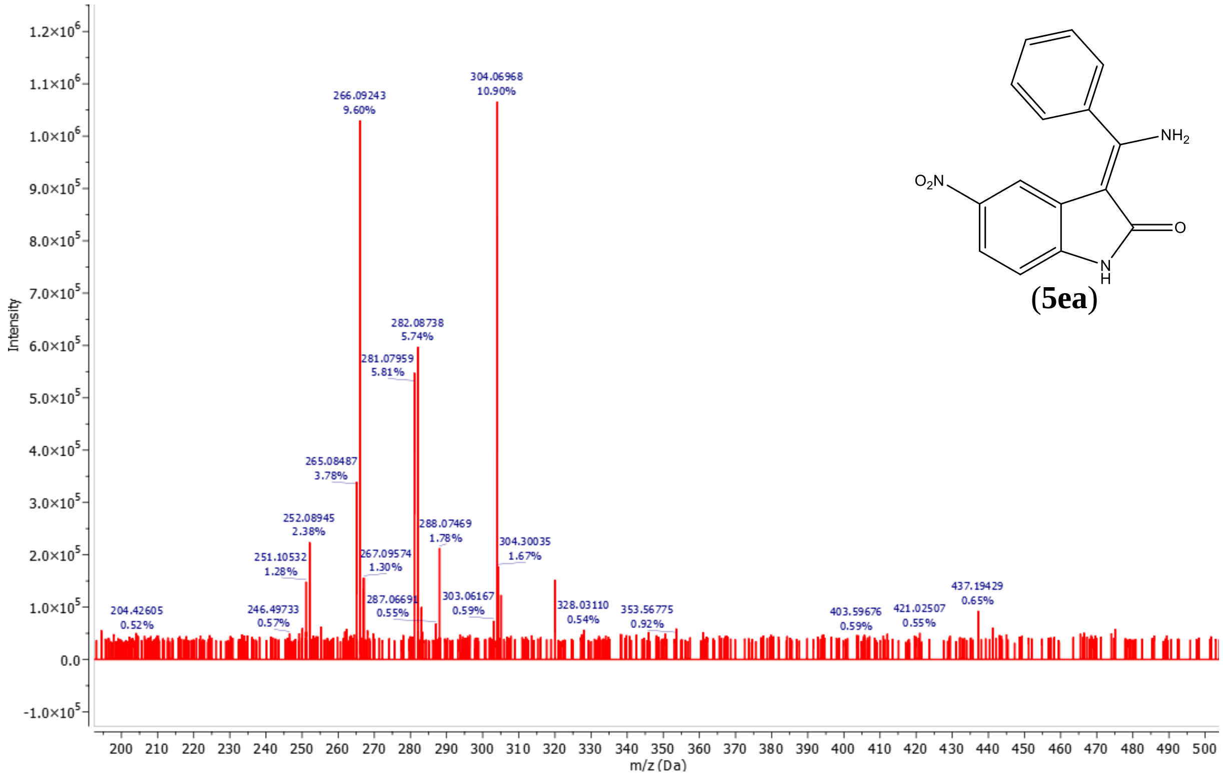

Intensity
m/z (Da)
266.09243 9.60%
304.06968 10.90%
282.08738 5.74%
281.07959 5.81%
265.08487 3.78%
252.08945 2.38%
251.10532 1.28%
267.09574 1.30%
288.07469 1.78%
304.30035 1.67%
204.42605 0.52%
246.49733 0.57%
287.06691 0.55%
303.06167 0.59%
328.03110 0.54%
353.56775 0.92%
403.59676 0.59%
421.02507 0.55%
437.19429 0.65%
NH_2
O_2N
O
N
H

(5ea)

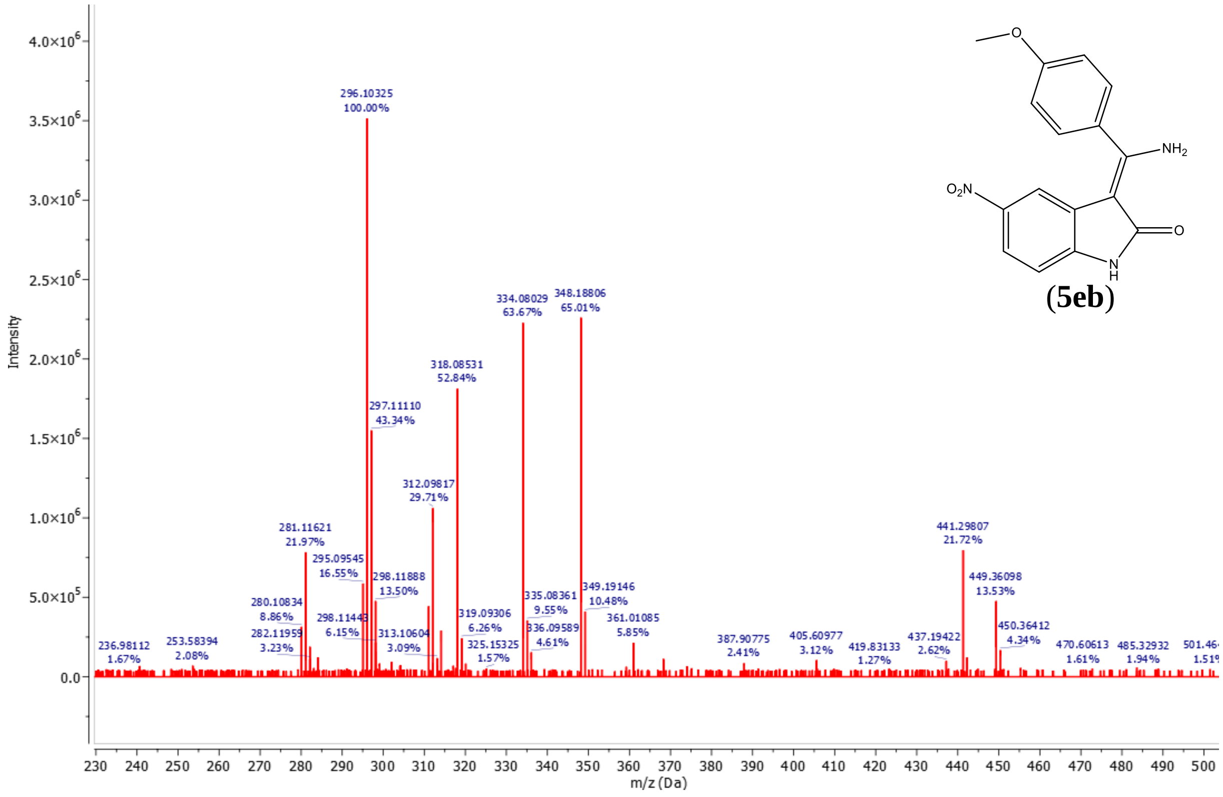

Intensity
m/z (Da)
296.10325 100.00%
334.08029 63.67%
348.18806 65.01%
318.08531 52.84%
297.11110 43.34%
312.09817 29.71%
281.11621 21.97%
295.09545 16.55%
298.11888 13.50%
280.10834 8.86%
298.11443 6.15%
282.11959 3.23%
313.10604 3.09%
319.09306 6.26%
325.15325 1.57%
335.08361 9.55%
336.09589 4.61%
349.19146 10.48%
361.01085 5.85%
236.98112 1.67%
253.58394 2.08%
387.90775 2.41%
405.60977 3.12%
419.83133 1.27%
437.19422 2.62%
441.29807 21.72%
449.36098 13.53%
450.36412 4.34%
470.60613 1.61%
485.32932 1.94%
O
NH_2
O_2N
O
N
H

(5eb)

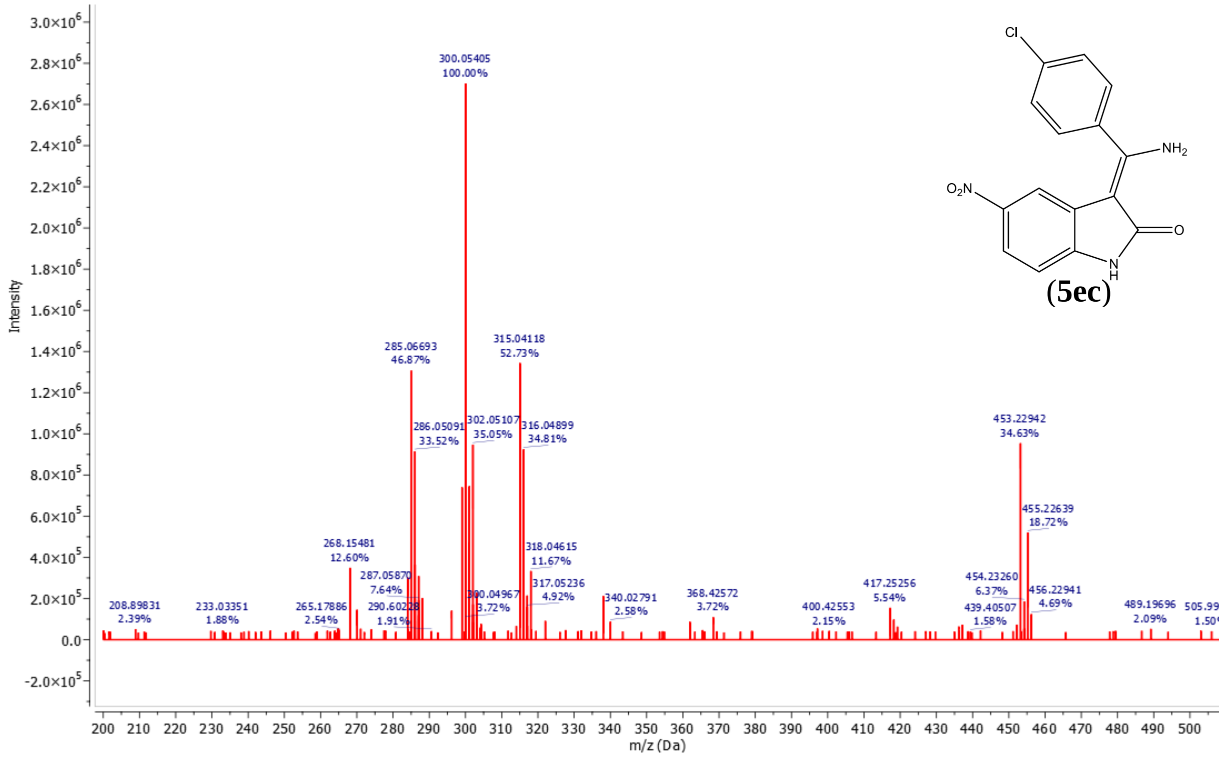

Intensity
m/z (Da)
300.05405
100.00%
315.04118
52.73%
285.06693
46.87%
286.05091
33.52%
302.05107
35.05%
316.04899
34.81%
453.22942
34.63%
455.22639
18.72%
268.15481
12.60%
318.04615
11.67%
287.05870
7.64%
317.05236
4.92%
454.23260
6.37%
417.25256
5.54%
456.22941
4.69%
368.42572
3.72%
340.02791
2.58%
208.89831
2.39%
233.03351
1.88%
265.17886
2.54%
290.60228
1.91%
400.42553
2.15%
439.40507
1.58%
489.19696
2.09%
Cl
NH_2
O_2N
O
N
H

(5ec)

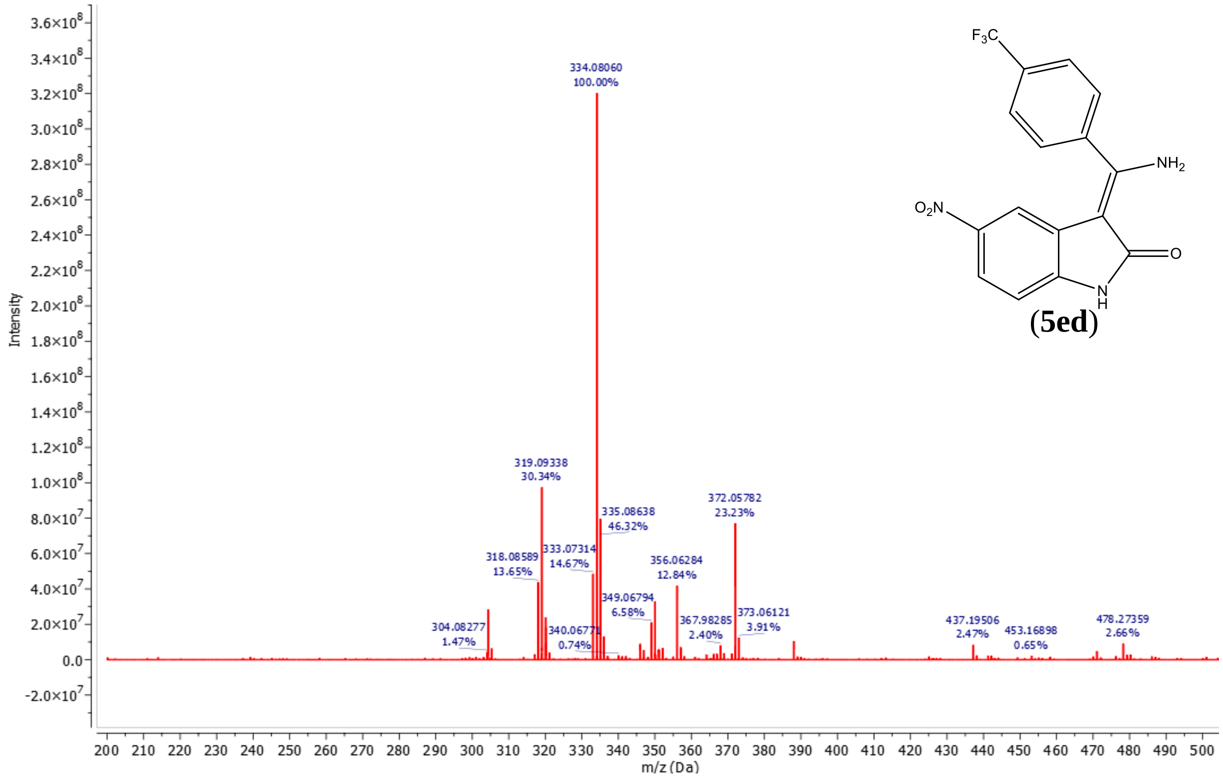

Intensity
m/z (Da)
334.08060
100.00%
319.09338
30.34%
335.08638
46.32%
372.05782
23.23%
333.07314
14.67%
318.08589
13.65%
356.06284
12.84%
349.06794
6.58%
304.08277
1.47%
340.06771
0.74%
367.98285
2.40%
373.06121
3.91%
437.19506
2.47%
453.16898
0.65%
478.27359
2.66%
F_3C
NH_2
O_2N
O
N
H

(5ed)

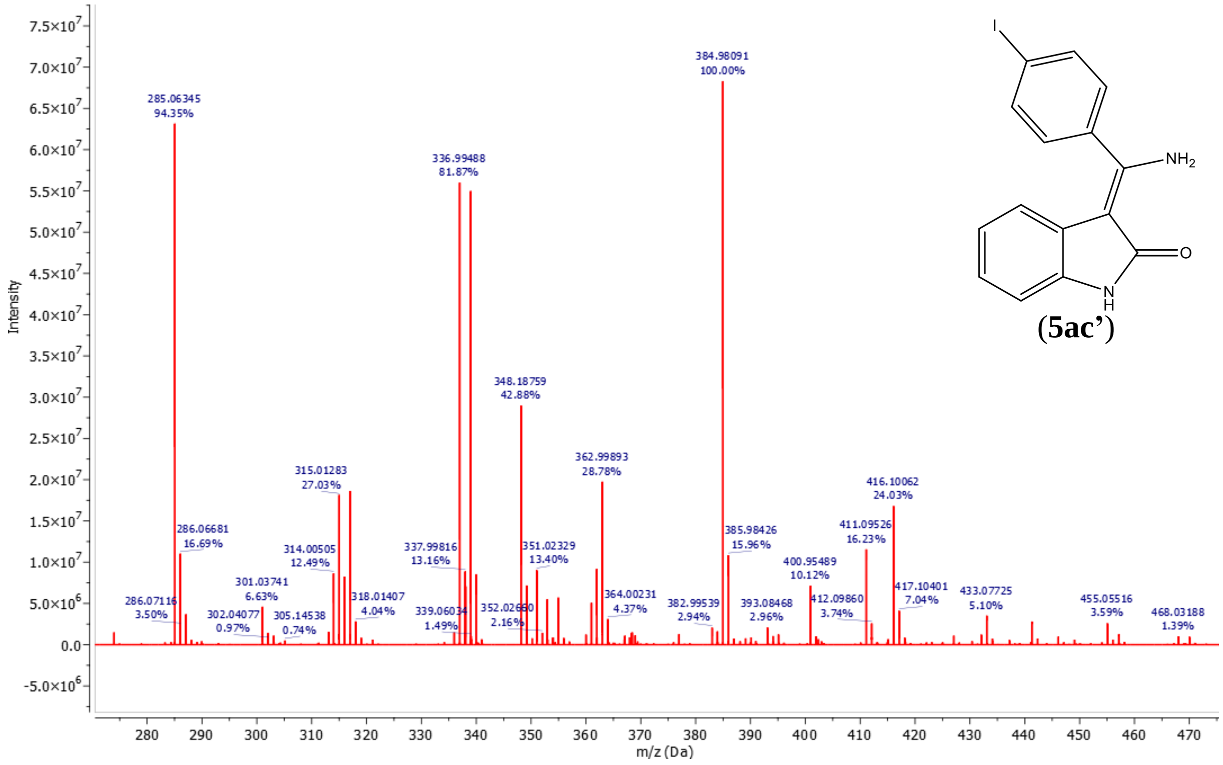

Intensity
m/z (Da)
285.06345
94.35%
384.98091
100.00%
336.99488
81.87%
348.18759
42.88%
362.99893
28.78%
315.01283
27.03%
416.10062
24.03%
286.06681
16.69%
314.00505
12.49%
337.99816
13.16%
351.02329
13.40%
385.98426
15.96%
411.09526
16.23%
400.95489
10.12%
301.03741
6.63%
286.07116
3.50%
302.04077
0.97%
305.14538
0.74%
318.01407
4.04%
339.06034
1.49%
352.02680
2.16%
364.00231
4.37%
382.99539
2.94%
393.08468
2.96%
412.09860
3.74%
417.10401
7.04%
433.07725
5.10%
455.05516
3.59%
468.03188
1.39%
I
NH_2
O
N
H

(5ac')

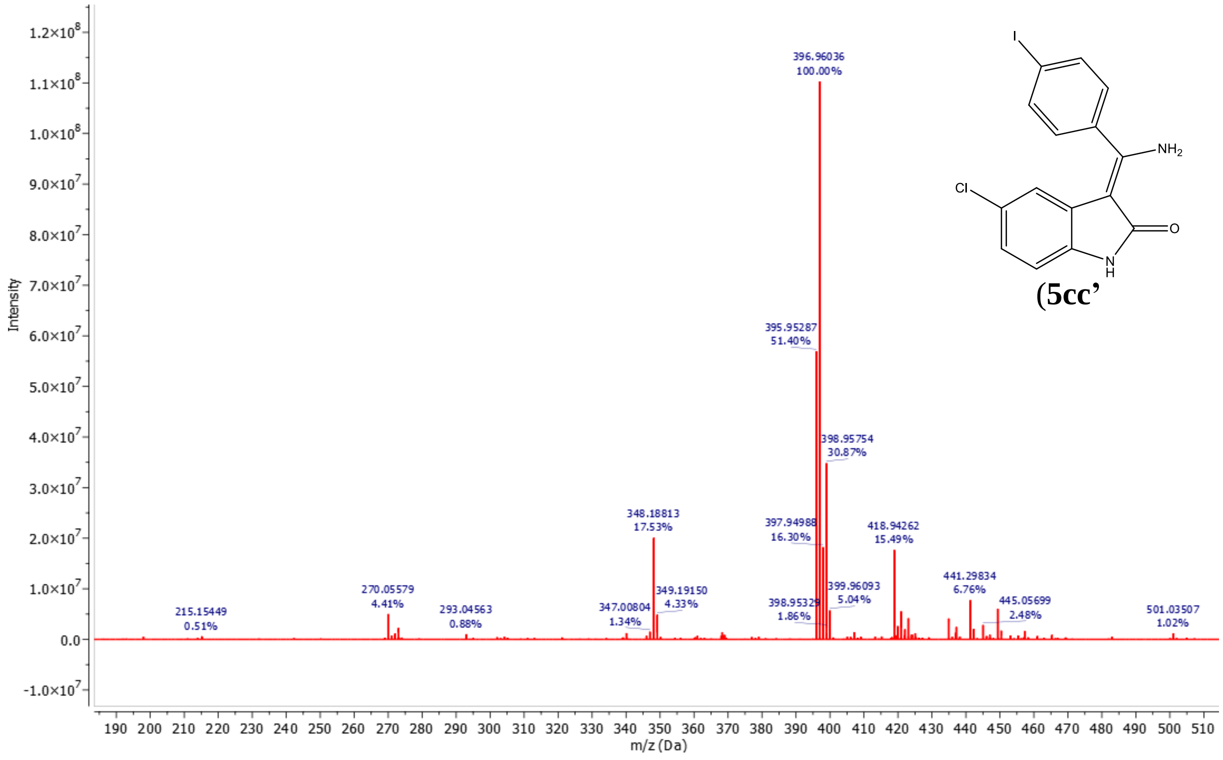

Intensity
m/z (Da)
396.96036
100.00%
395.95287
51.40%
398.95754
30.87%
348.18813
17.53%
397.94988
16.30%
418.94262
15.49%
441.29834
6.76%
270.05579
4.41%
349.19150
4.33%
399.96093
5.04%
445.05699
2.48%
347.00804
1.34%
398.95329
1.86%
293.04563
0.88%
215.15449
0.51%
501.03507
1.02%
I
NH_2
Cl
O
N
H

(5cc'

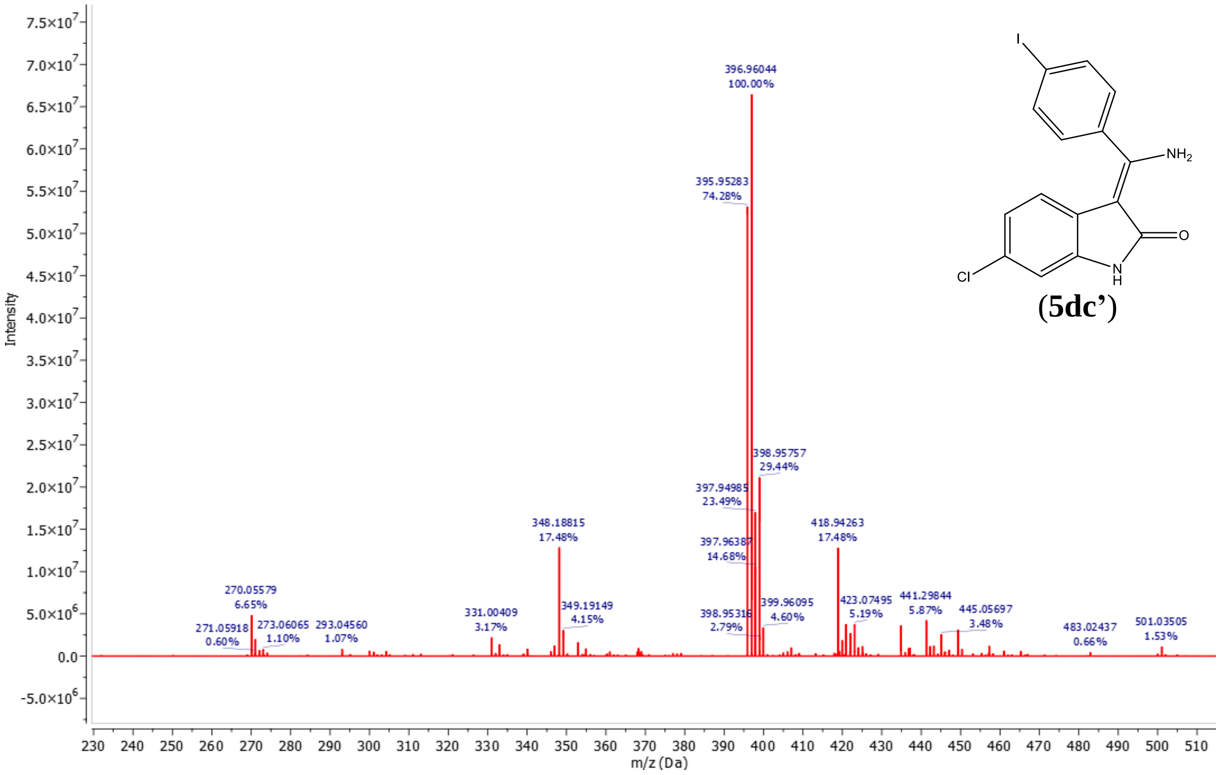
Intensity
m/z (Da)
396.96044
100.00%
395.95283
74.28%
398.95757
29.44%
397.94985
23.49%
348.18815
17.48%
418.94263
17.48%
397.96387
14.68%
270.05579
6.65%
441.29844
5.87%
423.07495
5.19%
399.96095
4.60%
349.19149
4.15%
445.05697
3.48%
331.00409
3.17%
398.95316
2.79%
501.03505
1.53%
273.06065
1.10%
293.04560
1.07%
483.02437
0.66%
271.05918
0.60%
I
NH2
O
Cl
N
H
(5dc')

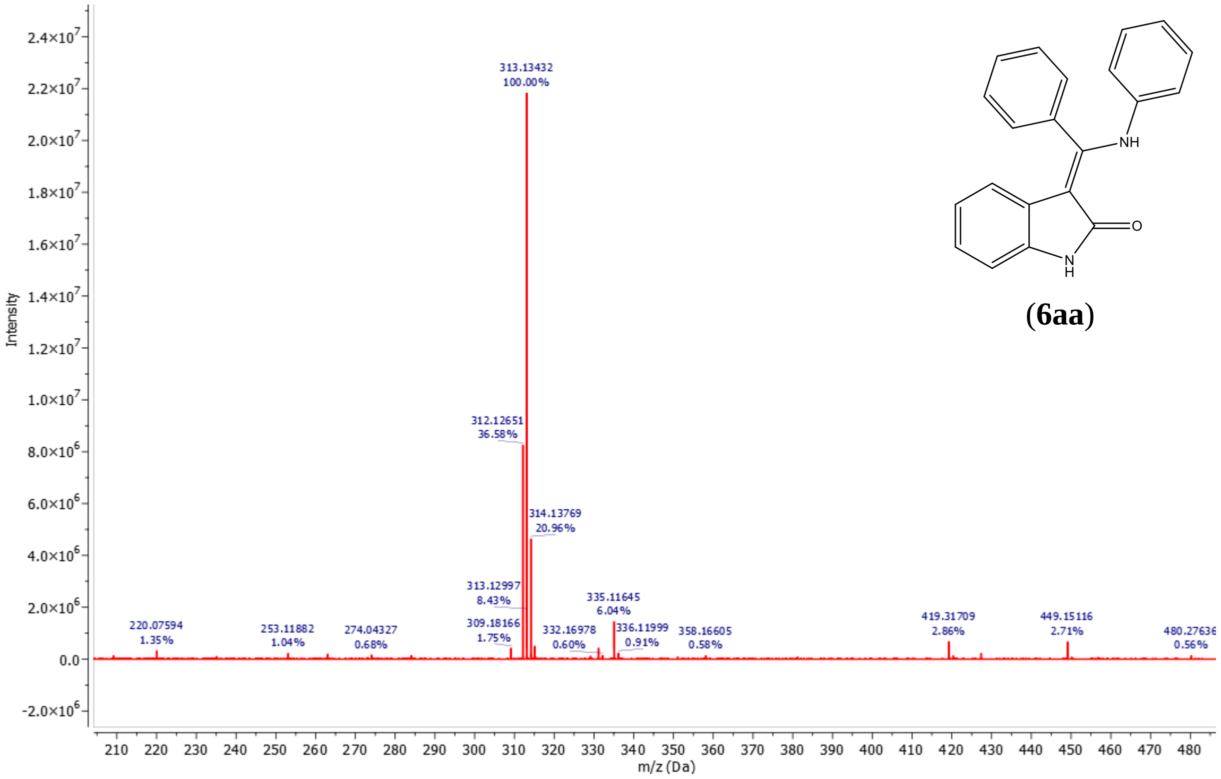
Intensity
m/z (Da)
313.13432
100.00%
312.12651
36.58%
314.13769
20.96%
313.12997
8.43%
335.11645
6.04%
419.31709
2.86%
449.15116
2.71%
309.18166
1.75%
220.07594
1.35%
253.11882
1.04%
336.11999
0.91%
274.04327
0.68%
332.16978
0.60%
358.16605
0.58%
480.27636
0.56%
NH
O
N
H
(6aa)

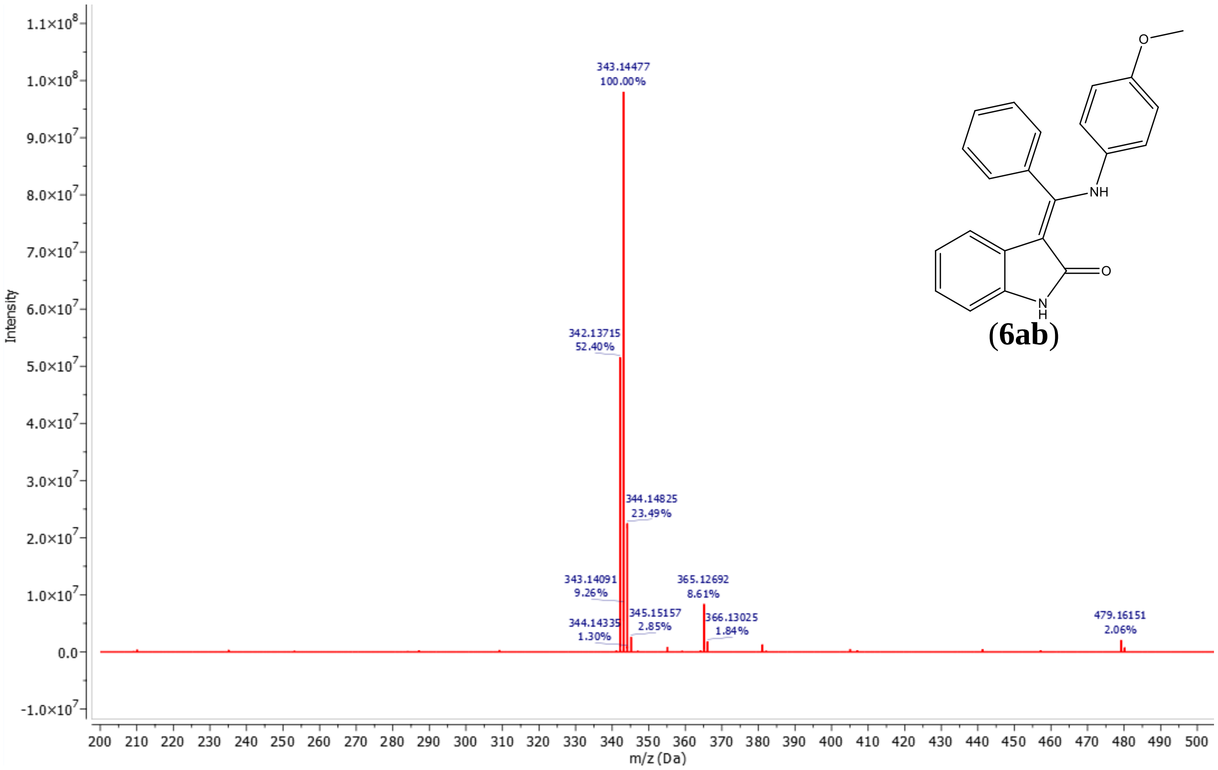
343.14477
100.00%
342.13715
52.40%
344.14825
23.49%
343.14091
9.26%
365.12692
8.61%
344.14335
1.30%
345.15157
2.85%
366.13025
1.84%
479.16151
2.06%
Intensity
m/z (Da)
NH
O
H

(6ab)

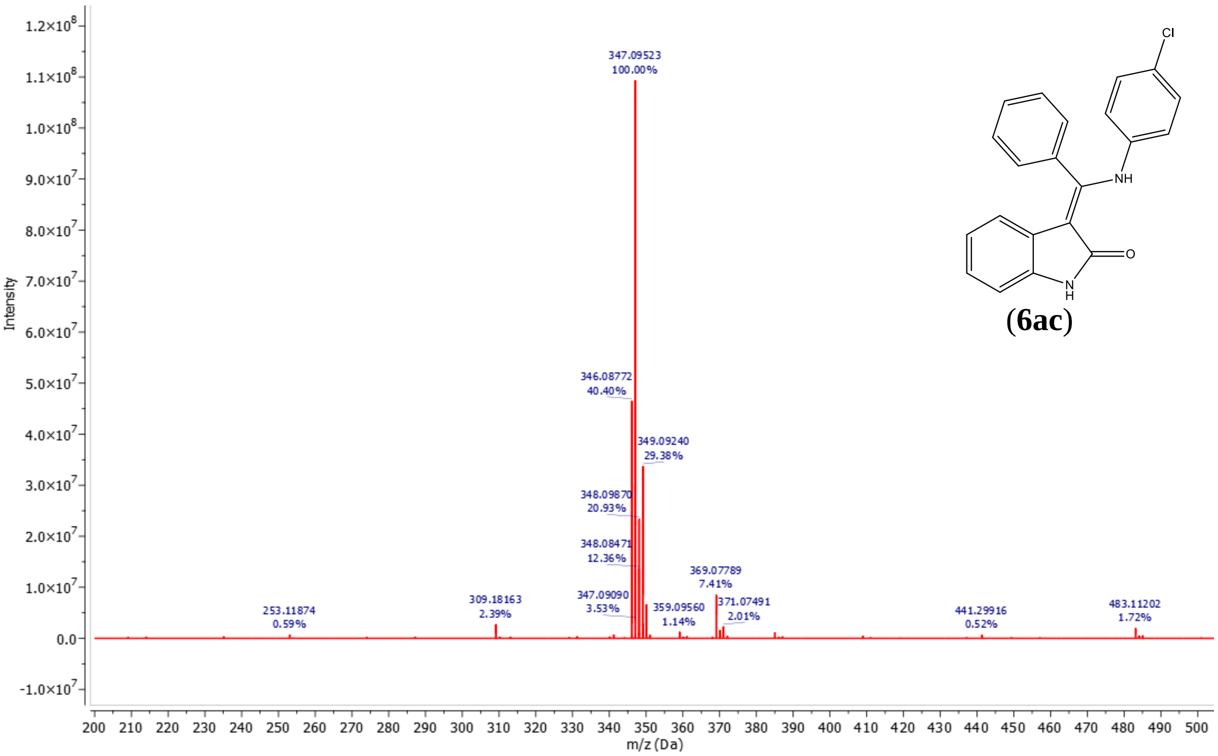
347.09523
100.00%
346.08772
40.40%
349.09240
29.38%
348.09870
20.93%
348.08471
12.36%
369.07789
7.41%
253.11874
0.59%
309.18163
2.39%
347.09090
3.53%
359.09560
1.14%
371.07491
2.01%
441.29916
0.52%
483.11202
1.72%
Intensity
m/z (Da)
Cl
NH
O
H

(6ac)

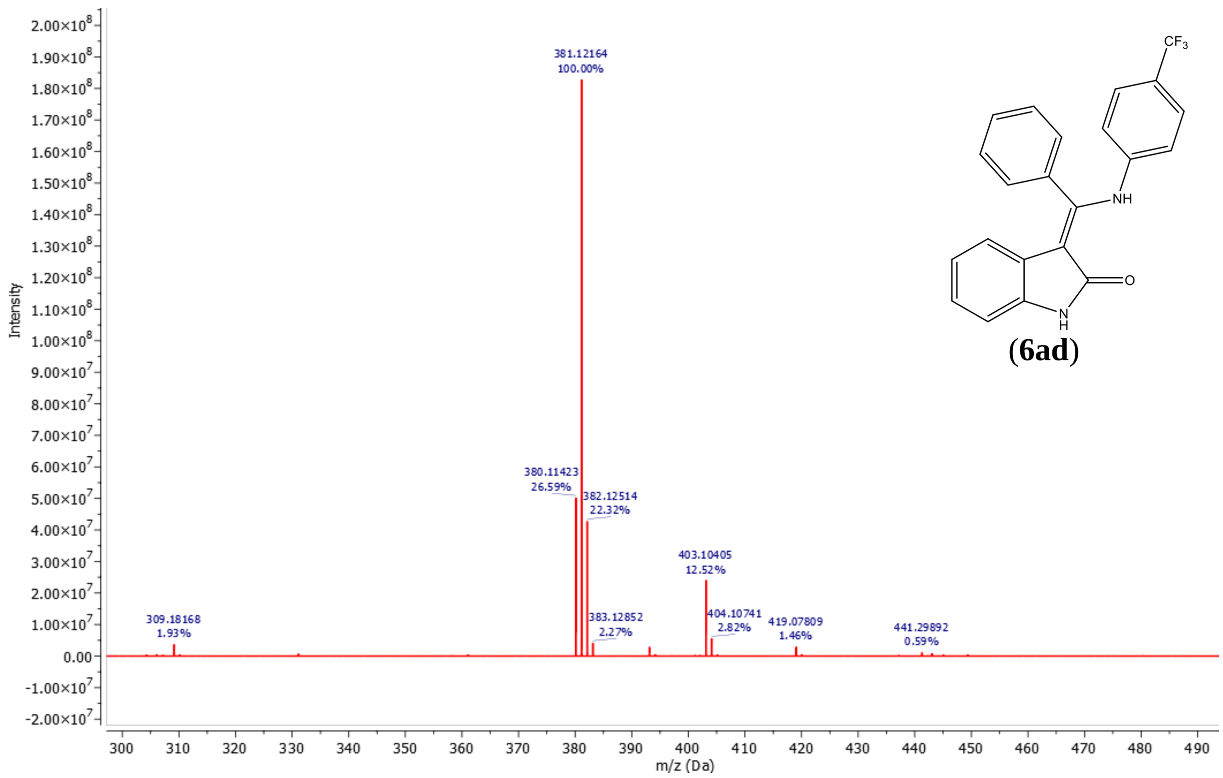
Intensity
m/z (Da)
381.12164 100.00%
380.11423 26.59%
382.12514 22.32%
403.10405 12.52%
383.12852 2.27%
404.10741 2.82%
419.07809 1.46%
441.29892 0.59%
309.18168 1.93%
CF3
NH
O
N H

(6ad)

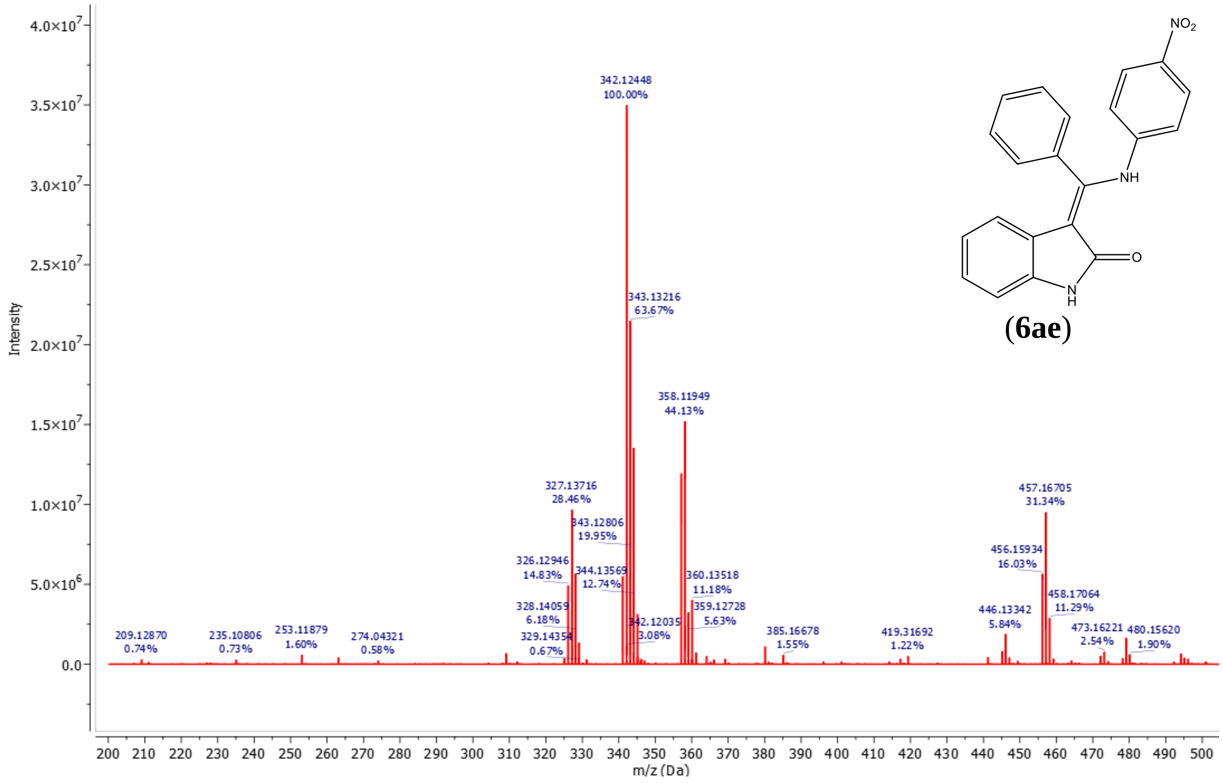
Intensity
m/z (Da)
342.12448 100.00%
343.13216 63.67%
358.11949 44.13%
457.16705 31.34%
327.13716 28.46%
343.12806 19.95%
456.15934 16.03%
326.12946 14.83%
344.13569 12.74%
360.13518 11.18%
458.17064 11.29%
328.14059 6.18%
359.12728 5.63%
446.13342 5.84%
329.14354 0.67%
342.12035 3.08%
385.16678 1.55%
419.31692 1.22%
473.16221 2.54%
480.15620 1.90%
209.12870 0.74%
235.10806 0.73%
253.11879 1.60%
274.04321 0.58%
NO2
NH
O
N H

(6ae)

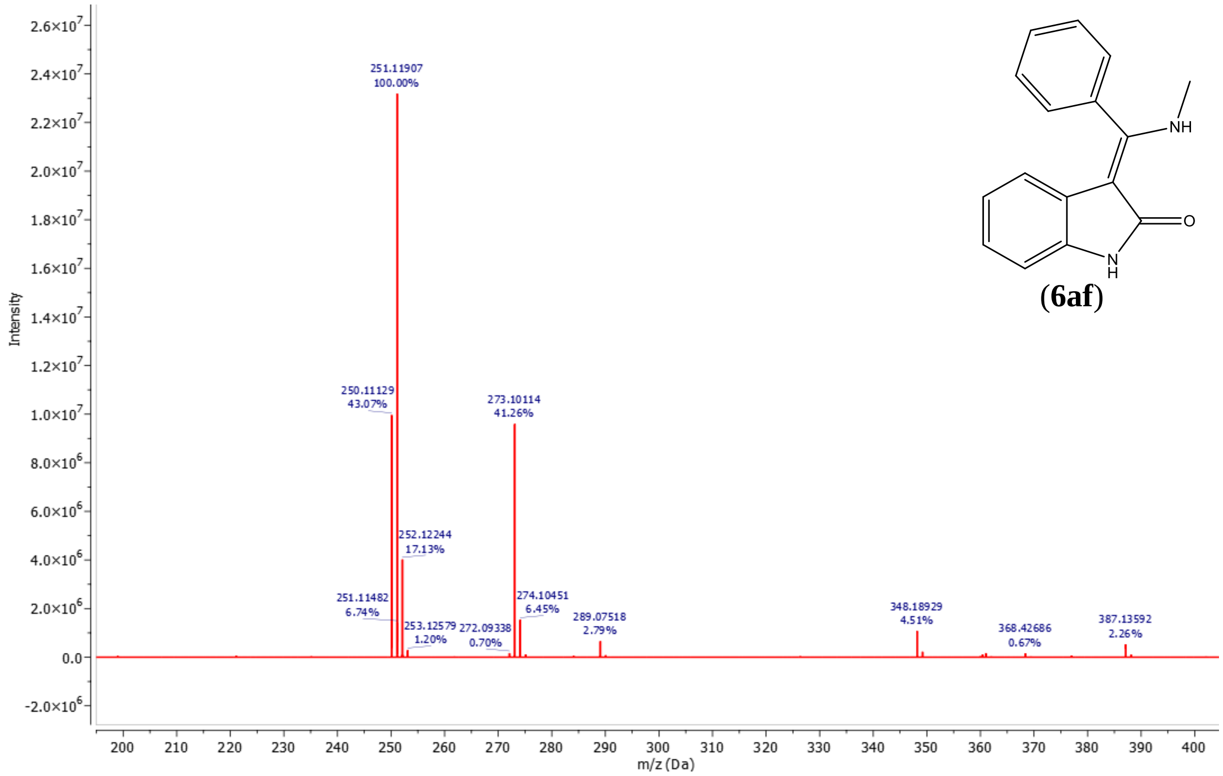
Intensity
m/z (Da)
251.11907
100.00%
250.11129
43.07%
273.10114
41.26%
252.12244
17.13%
251.11482
6.74%
274.10451
6.45%
253.12579
1.20%
272.09338
0.70%
289.07518
2.79%
348.18929
4.51%
368.42686
0.67%
387.13592
2.26%
NH
O
N
H

(6af)

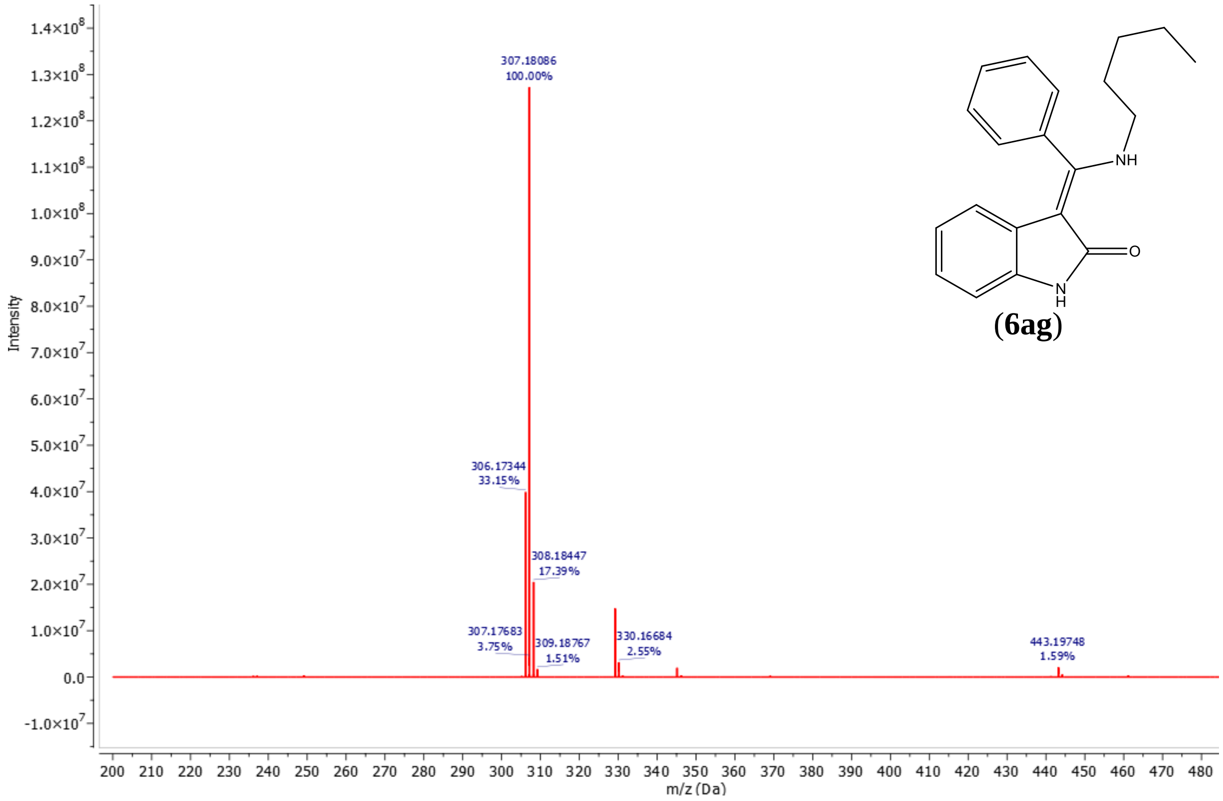
Intensity
m/z (Da)
307.18086
100.00%
306.17344
33.15%
308.18447
17.39%
307.17683
3.75%
309.18767
1.51%
330.16684
2.55%
443.19748
1.59%
NH
O
N
H

(6ag)

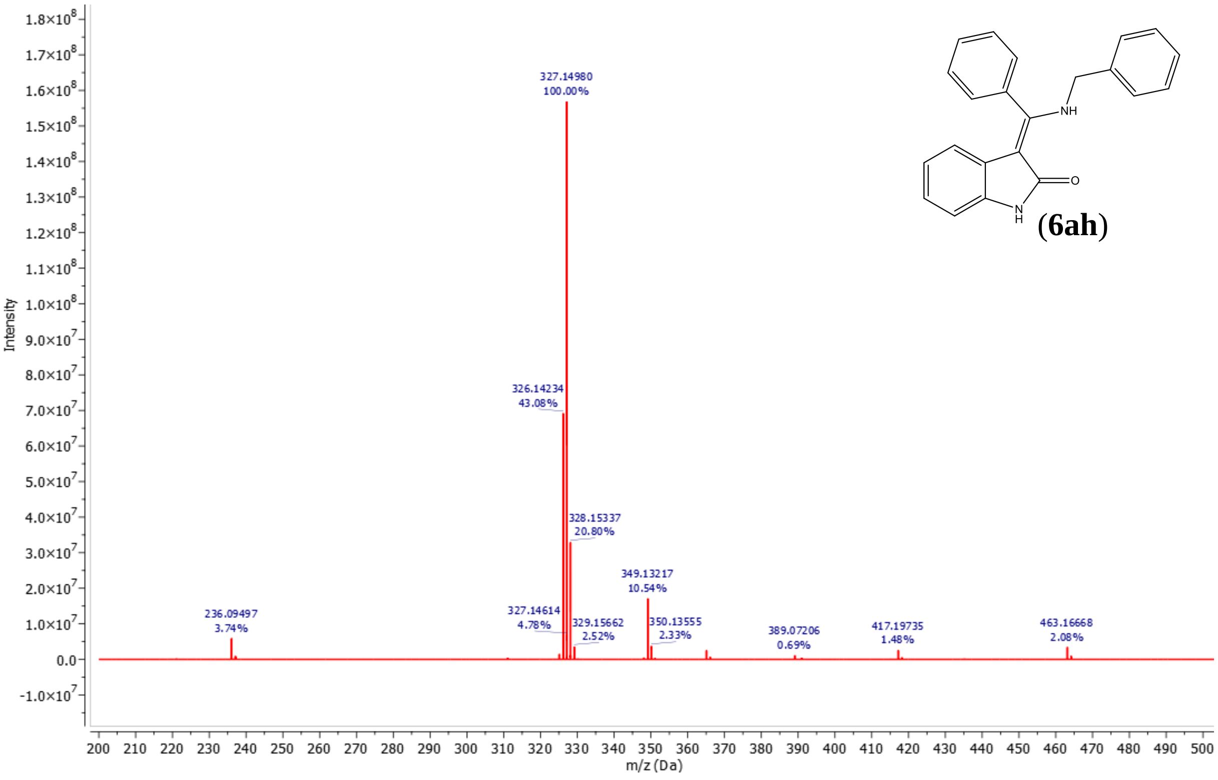
327.14980
100.00%
326.14234
43.08%
328.15337
20.80%
349.13217
10.54%
236.09497
3.74%
327.14614
4.78%
329.15662
2.52%
350.13555
2.33%
389.07206
0.69%
417.19735
1.48%
463.16668
2.08%
Intensity
m/z (Da)
NH
O
N
H

(6ah)

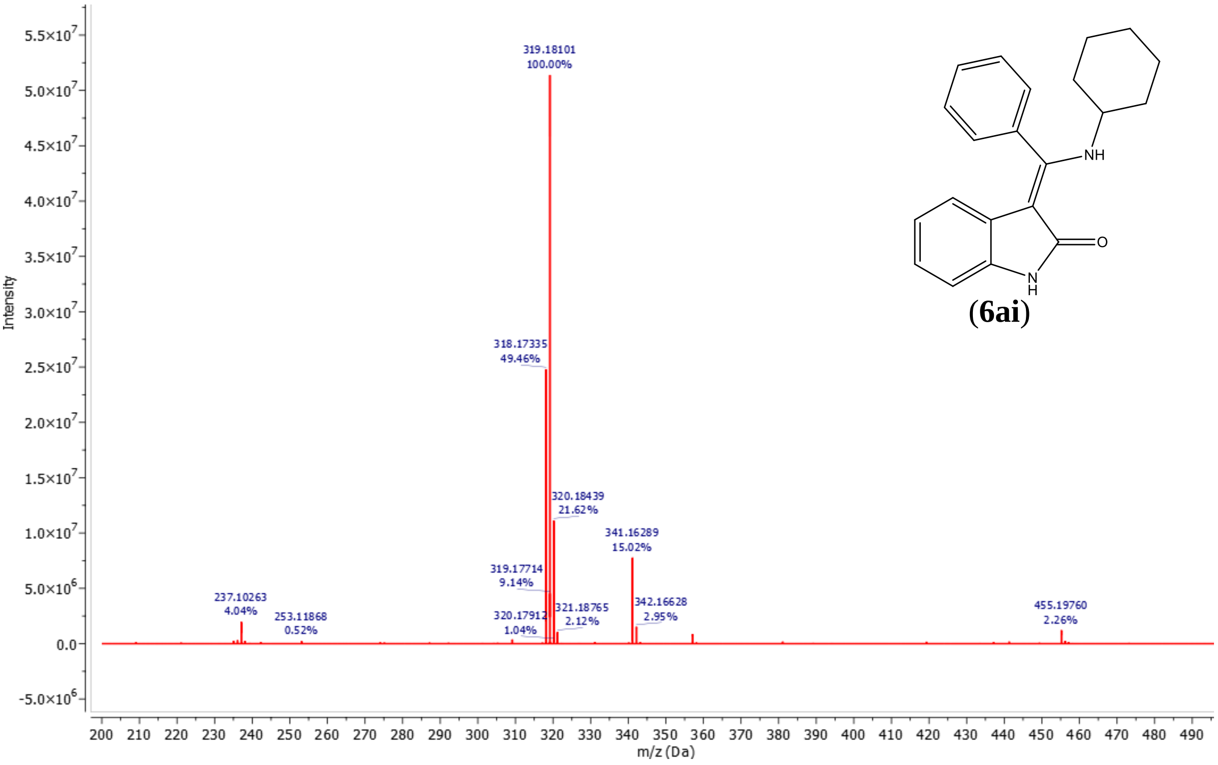
319.18101
100.00%
318.17335
49.46%
320.18439
21.62%
341.16289
15.02%
319.17714
9.14%
237.10263
4.04%
253.11868
0.52%
320.17912
1.04%
321.18765
2.12%
342.16628
2.95%
455.19760
2.26%
Intensity
m/z (Da)
NH
O
N
H

(6ai)

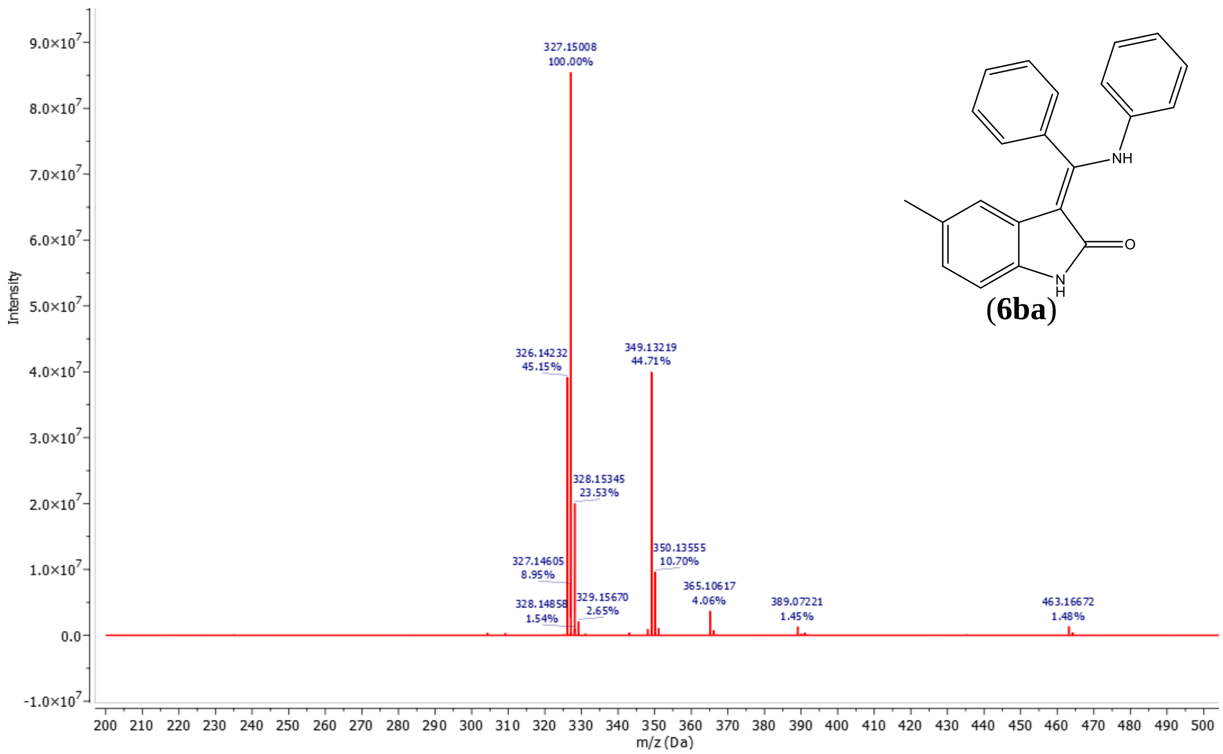
327.15008
100.00%
326.14232
45.15%
349.13219
44.71%
328.15345
23.53%
350.13555
10.70%
327.14605
8.95%
365.10617
4.06%
329.15670
2.65%
328.14858
1.54%
389.07221
1.45%
463.16672
1.48%
NH
O
N
H
(6ba)
Intensity
m/z (Da)

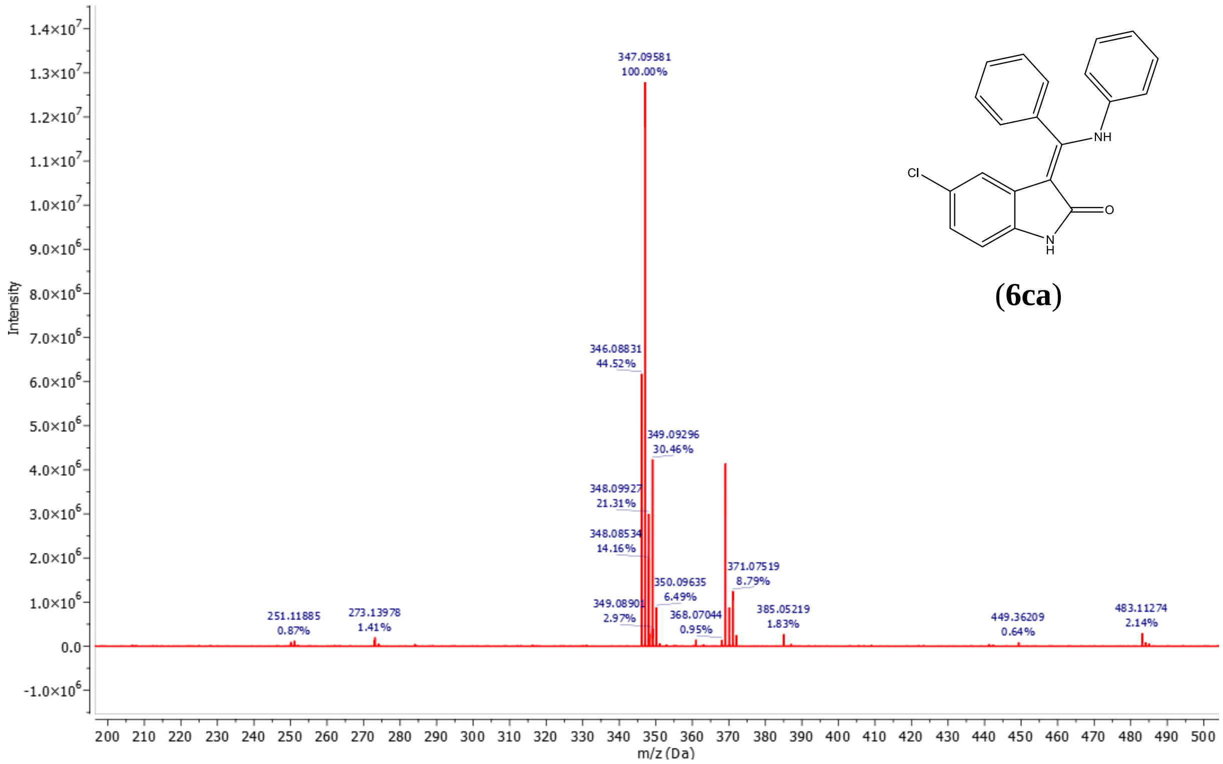
347.09581
100.00%
346.08831
44.52%
349.09296
30.46%
348.09927
21.31%
348.08534
14.16%
371.07519
8.79%
350.09635
6.49%
349.08901
2.97%
368.07044
0.95%
385.05219
1.83%
251.11885
0.87%
273.13978
1.41%
449.36209
0.64%
483.11274
2.14%
Cl
NH
O
N
H
(6ca)
Intensity
m/z (Da)

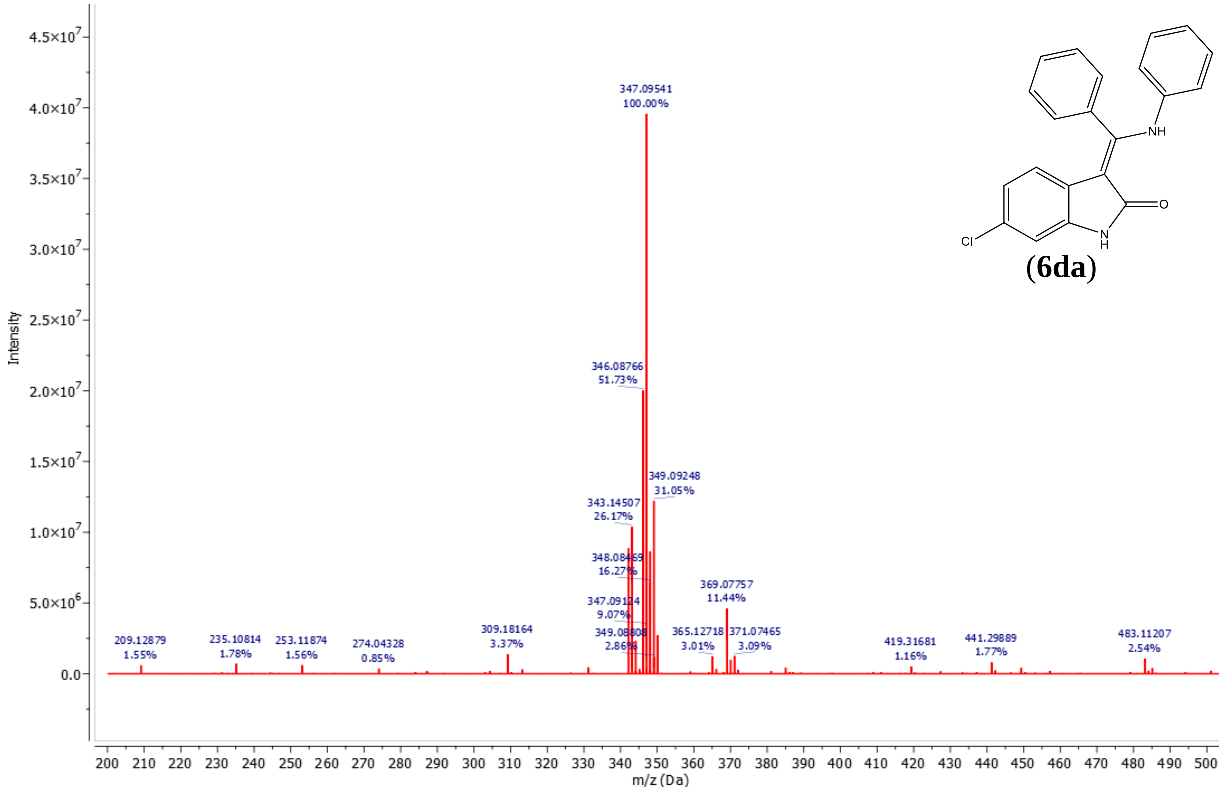
(6da)
NH
O
N
H
Cl
Intensity
m/z (Da)
347.09541
100.00%
346.08766
51.73%
349.09248
31.05%
343.14507
26.17%
348.08469
16.27%
347.09124
9.07%
349.08808
2.86%
369.07757
11.44%
365.12718
3.01%
371.07465
3.09%
209.12879
1.55%
235.10814
1.78%
253.11874
1.56%
274.04328
0.85%
309.18164
3.37%
419.31681
1.16%
441.29889
1.77%
483.11207
2.54%

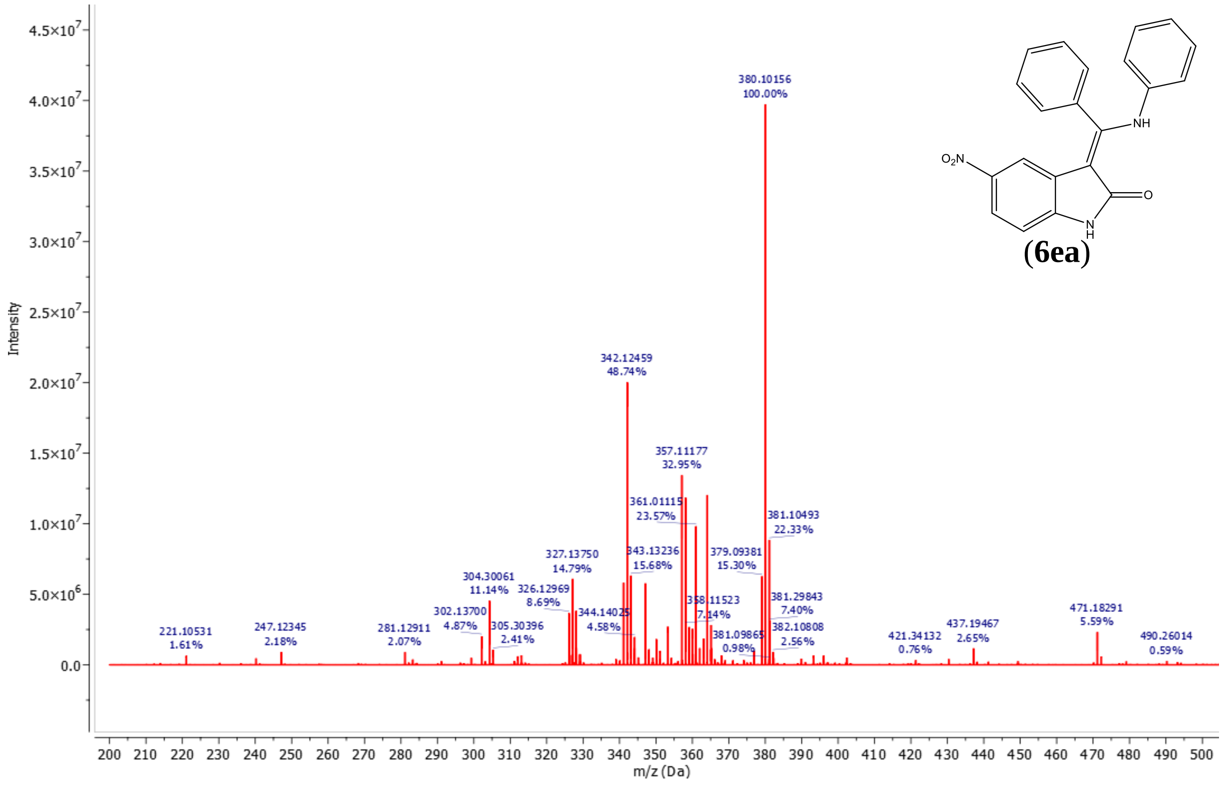
(6ea)
NH
O_2N
O
N
H
Intensity
m/z (Da)
380.10156
100.00%
342.12459
48.74%
357.11177
32.95%
361.01115
23.57%
381.10493
22.33%
343.13236
15.68%
379.09381
15.30%
327.13750
14.79%
304.30061
11.14%
326.12969
8.69%
381.29843
7.40%
358.11523
7.14%
302.13700
4.87%
344.14025
4.58%
305.30396
2.41%
381.09865
0.98%
382.10808
2.56%
221.10531
1.61%
247.12345
2.18%
281.12911
2.07%
421.34132
0.76%
437.19467
2.65%
471.18291
5.59%
490.26014
0.59%

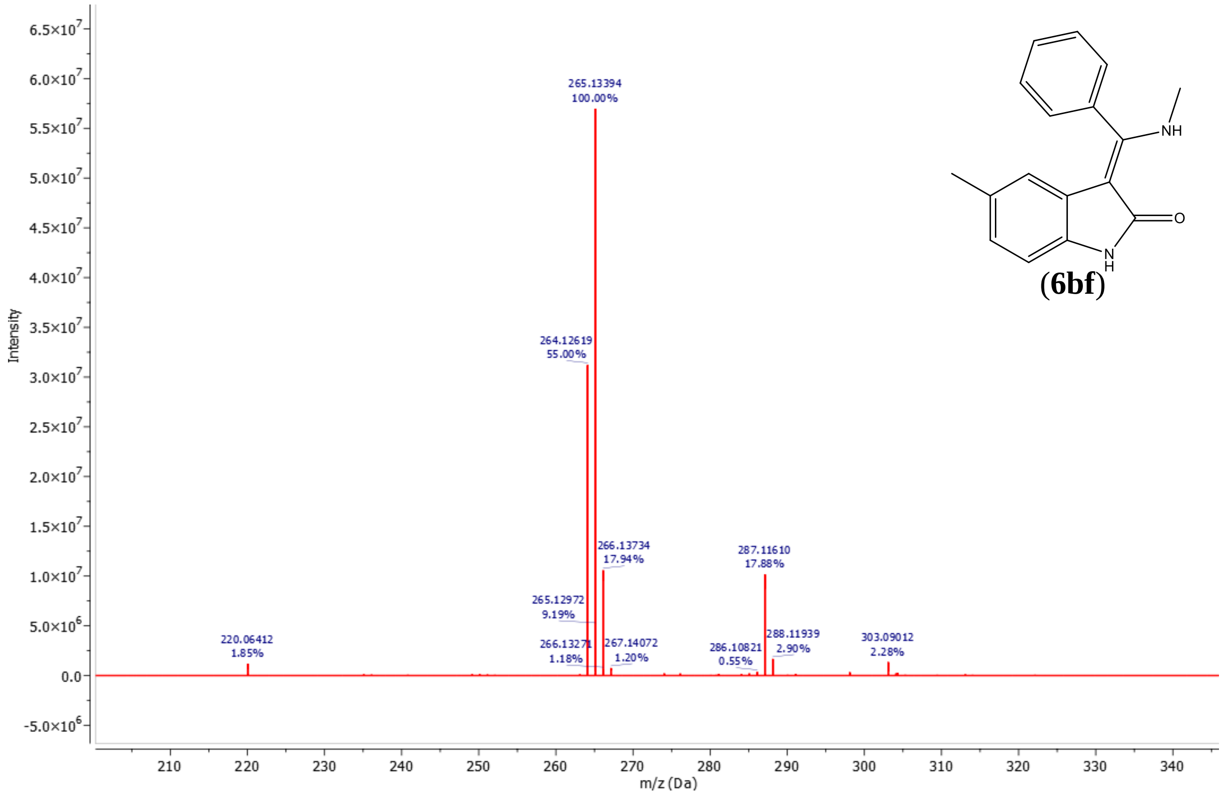
265.13394
100.00%
264.12619
55.00%
266.13734
17.94%
287.11610
17.88%
265.12972
9.19%
220.06412
1.85%
266.13271
1.18%
267.14072
1.20%
286.10821
0.55%
288.11939
2.90%
303.09012
2.28%
Intensity
m/z (Da)
NH
O
N
H

(6bf)

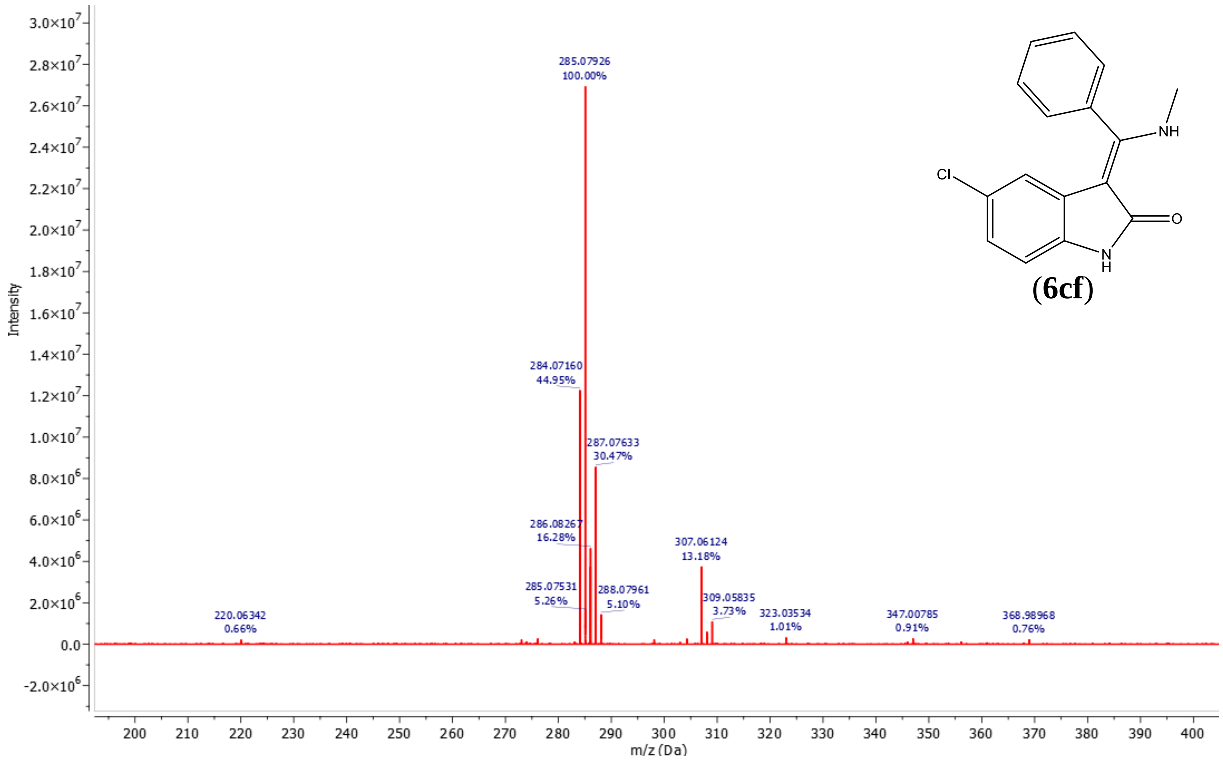
285.07926
100.00%
284.07160
44.95%
287.07633
30.47%
286.08267
16.28%
307.06124
13.18%
285.07531
5.26%
288.07961
5.10%
309.05835
3.73%
220.06342
0.66%
323.03534
1.01%
347.00785
0.91%
368.98968
0.76%
Intensity
m/z (Da)
Cl
NH
O
N
H

(6cf)

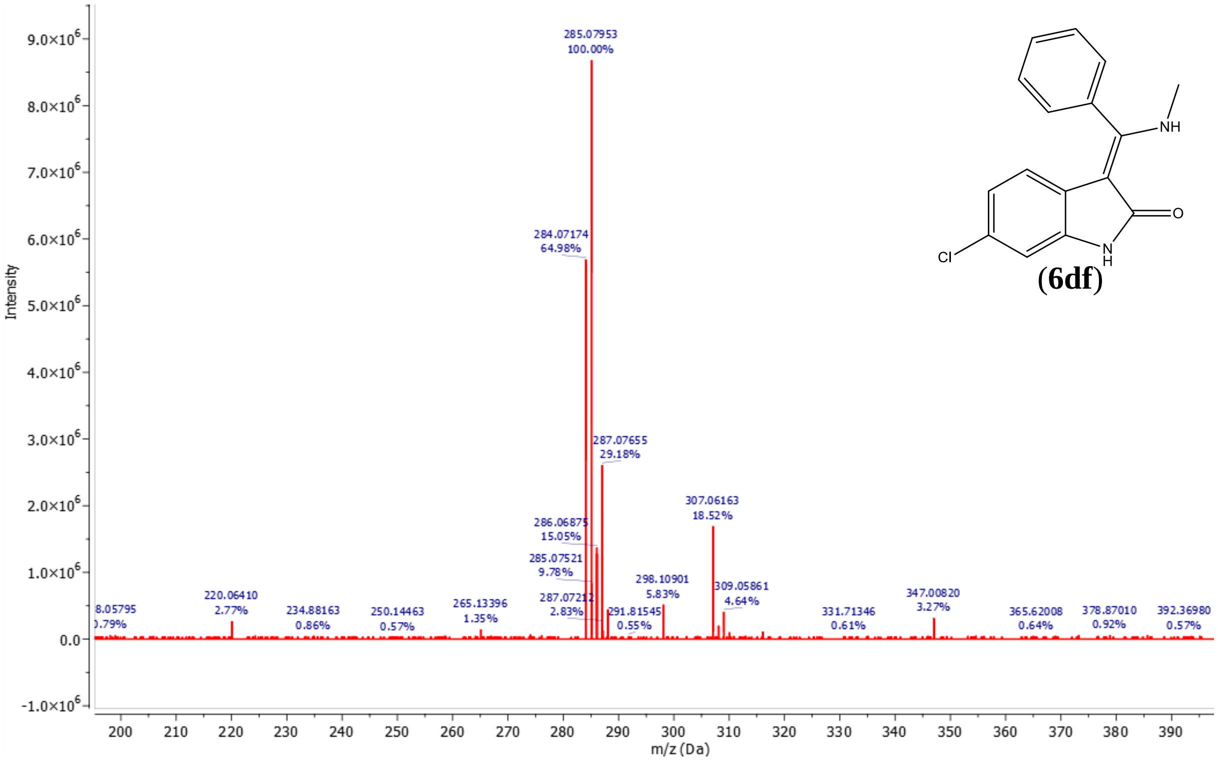
285.07953
100.00%
284.07174
64.98%
287.07655
29.18%
307.06163
18.52%
286.06875
15.05%
285.07521
9.78%
298.10901
5.83%
309.05861
4.64%
287.07212
2.83%
291.81545
0.55%
220.06410
2.77%
234.88163
0.86%
250.14463
0.57%
265.13396
1.35%
331.71346
0.61%
347.00820
3.27%
365.62008
0.64%
378.87010
0.92%
392.36980
0.57%
Intensity
m/z (Da)
NH
O
N
H
Cl
(6df)

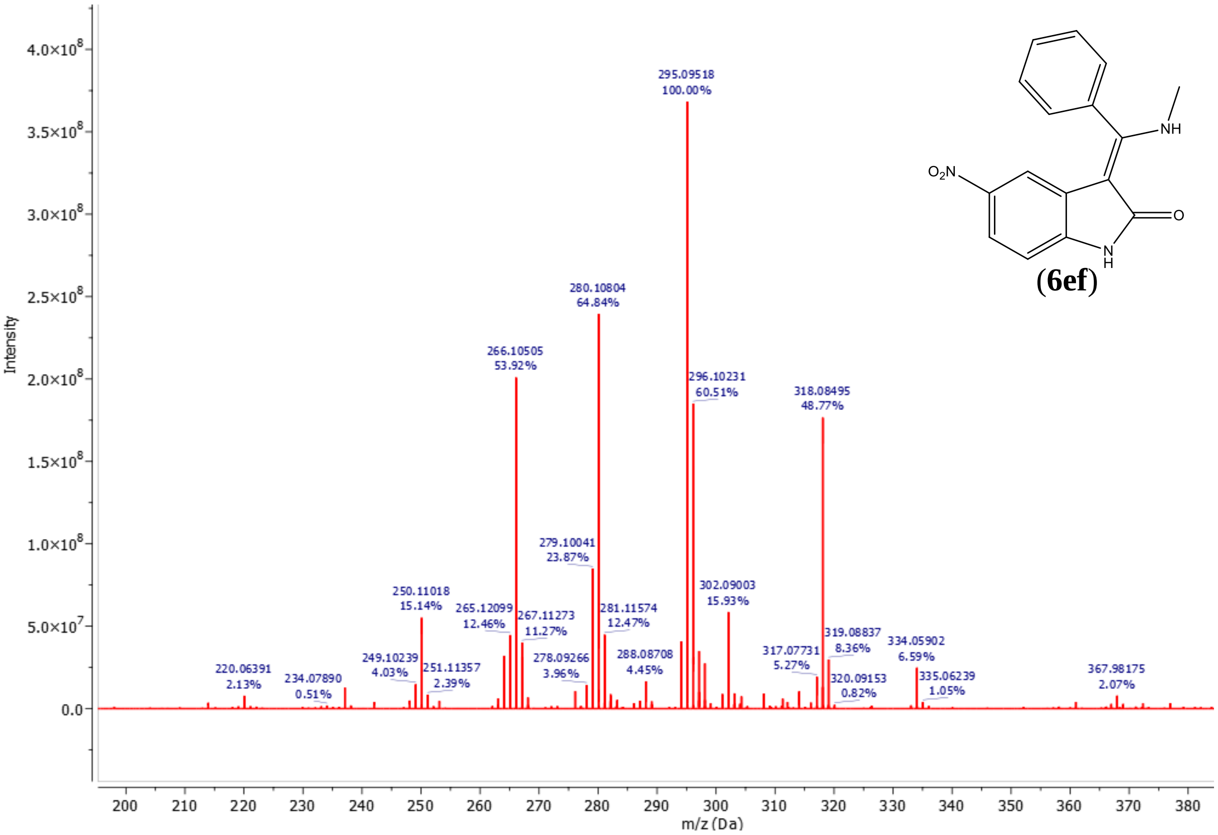
295.09518
100.00%
280.10804
64.84%
266.10505
53.92%
296.10231
60.51%
318.08495
48.77%
279.10041
23.87%
302.09003
15.93%
250.11018
15.14%
265.12099
12.46%
267.11273
11.27%
281.11574
12.47%
319.08837
8.36%
334.05902
6.59%
317.07731
5.27%
288.08708
4.45%
249.10239
4.03%
278.09266
3.96%
251.11357
2.39%
220.06391
2.13%
234.07890
0.51%
320.09153
0.82%
335.06239
1.05%
367.98175
2.07%
Intensity
m/z (Da)
NH
O_2N
O
N
H
(6ef)

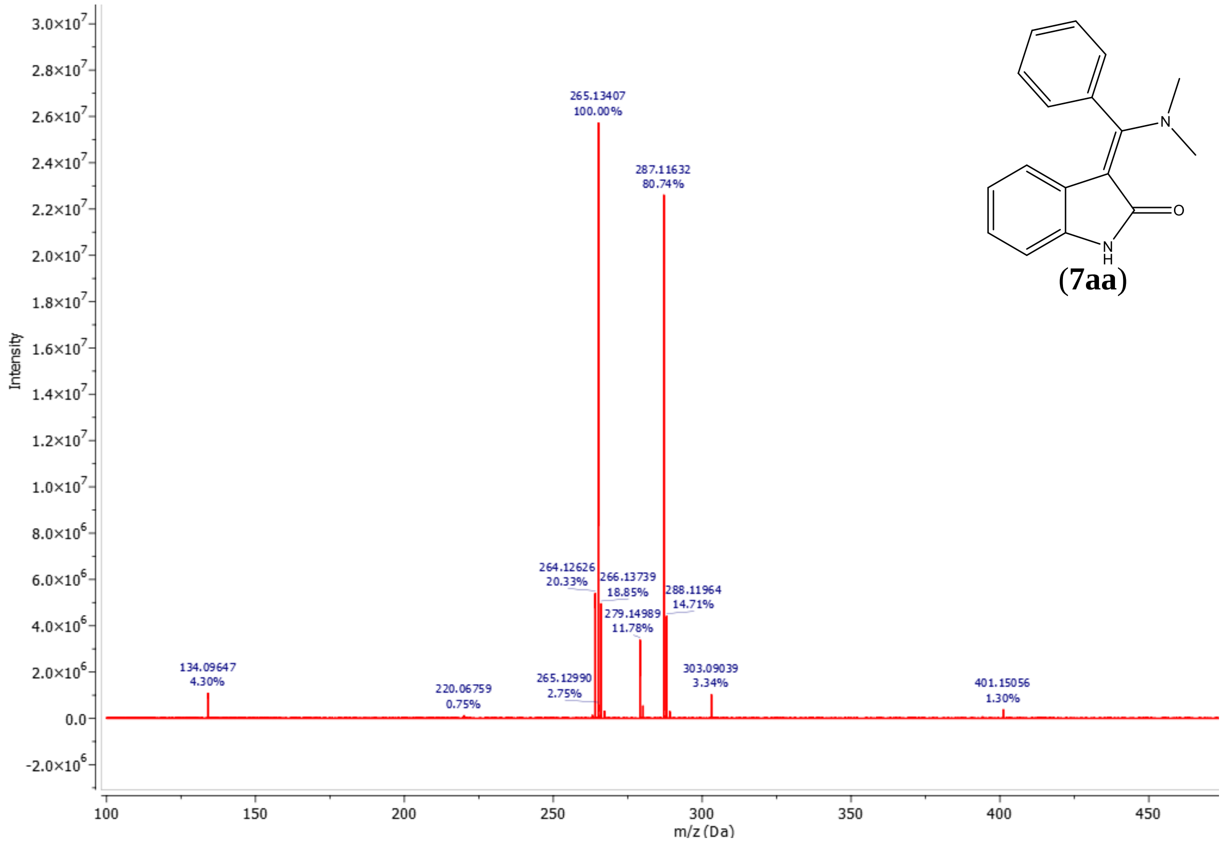
265.13407
100.00%
287.11632
80.74%
264.12626
20.33%
266.13739
18.85%
288.11964
14.71%
279.14989
11.78%
134.09647
4.30%
220.06759
0.75%
265.12990
2.75%
303.09039
3.34%
401.15056
1.30%
Intensity
m/z (Da)
N
O
H
(7aa)

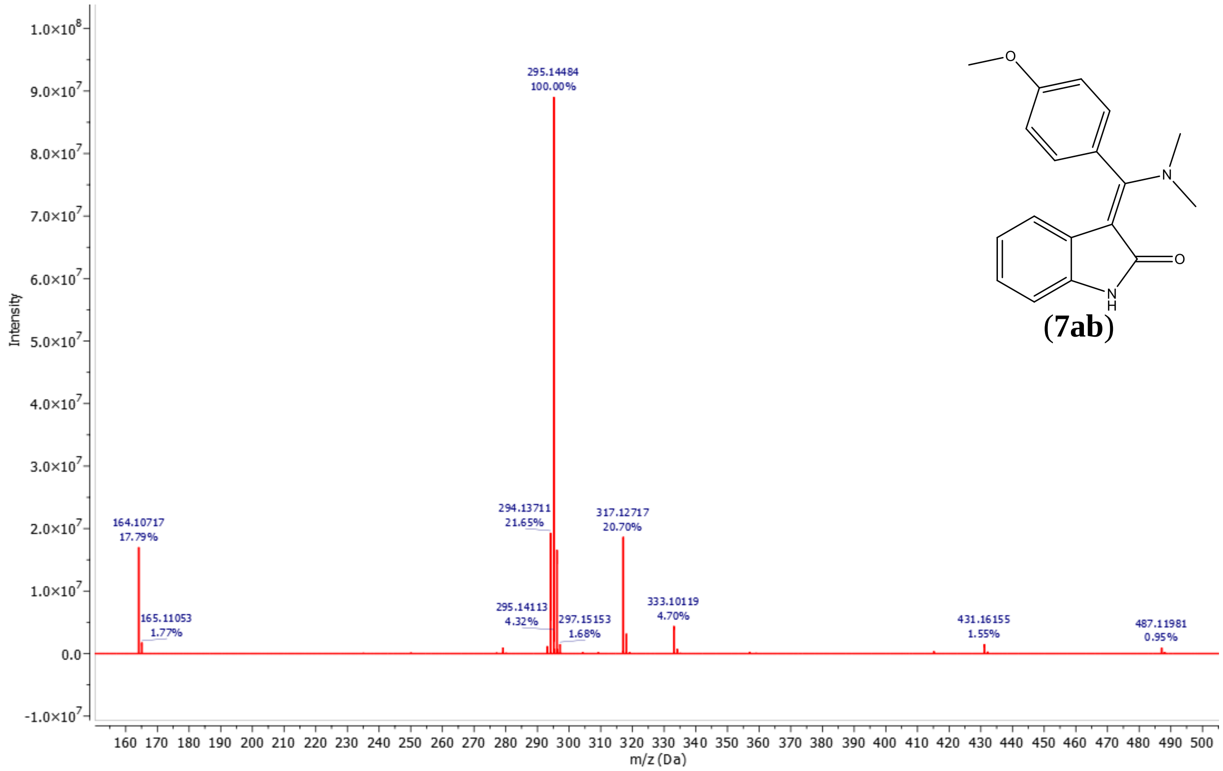
295.14484
100.00%
294.13711
21.65%
317.12717
20.70%
164.10717
17.79%
165.11053
1.77%
295.14113
4.32%
297.15153
1.68%
333.10119
4.70%
431.16155
1.55%
487.11981
0.95%
Intensity
m/z (Da)
O
N
O
H
(7ab)

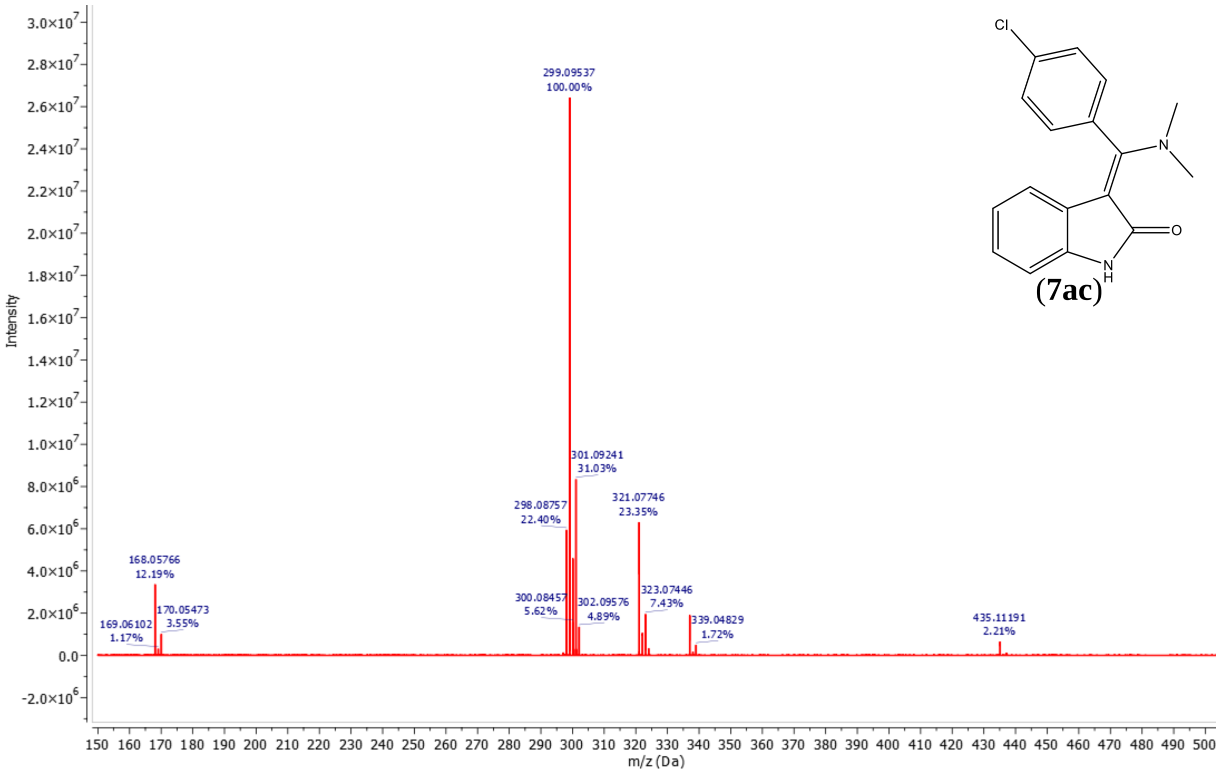

Intensity
m/z (Da)
299.09537
100.00%
301.09241
31.03%
298.08757
22.40%
321.07746
23.35%
168.05766
12.19%
170.05473
3.55%
169.06102
1.17%
300.08457
5.62%
302.09576
4.89%
323.07446
7.43%
339.04829
1.72%
435.11191
2.21%
Cl
N
O
N
H
(7ac)

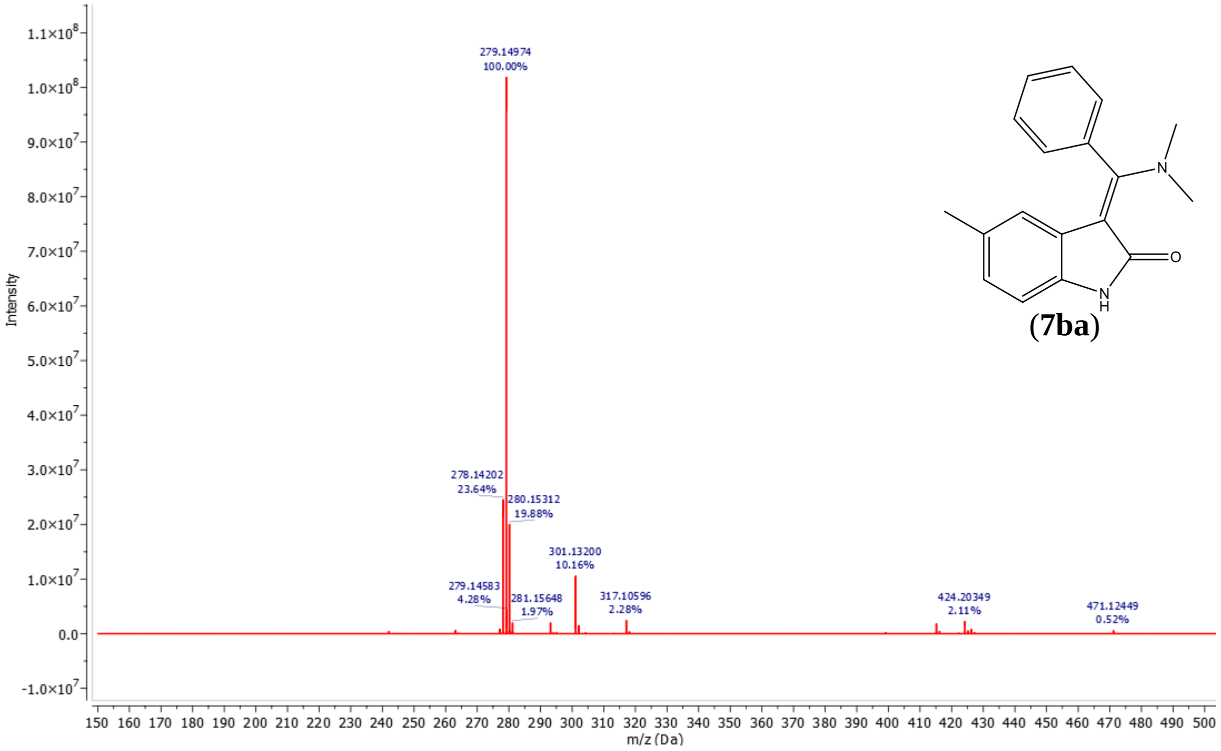

Intensity
m/z (Da)
279.14974
100.00%
278.14202
23.64%
280.15312
19.88%
301.13200
10.16%
279.14583
4.28%
281.15648
1.97%
317.10596
2.28%
424.20349
2.11%
471.12449
0.52%
N
O
N
H
(7ba)

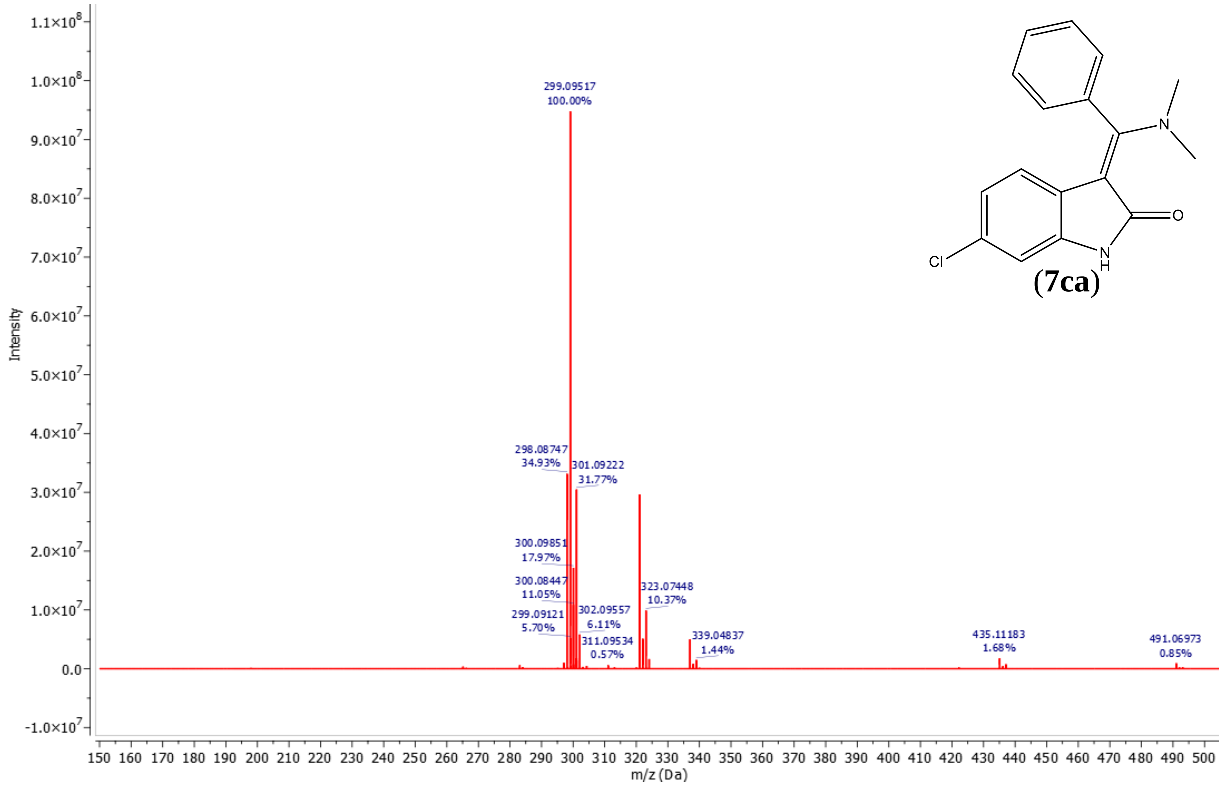

Intensity
m/z (Da)
299.09517
100.00%
298.08747
34.93%
301.09222
31.77%
300.09851
17.97%
300.08447
11.05%
323.07448
10.37%
302.09557
6.11%
299.09121
5.70%
311.09534
0.57%
339.04837
1.44%
435.11183
1.68%
491.06973
0.85%
N
O
Cl
N
H

(7ca)

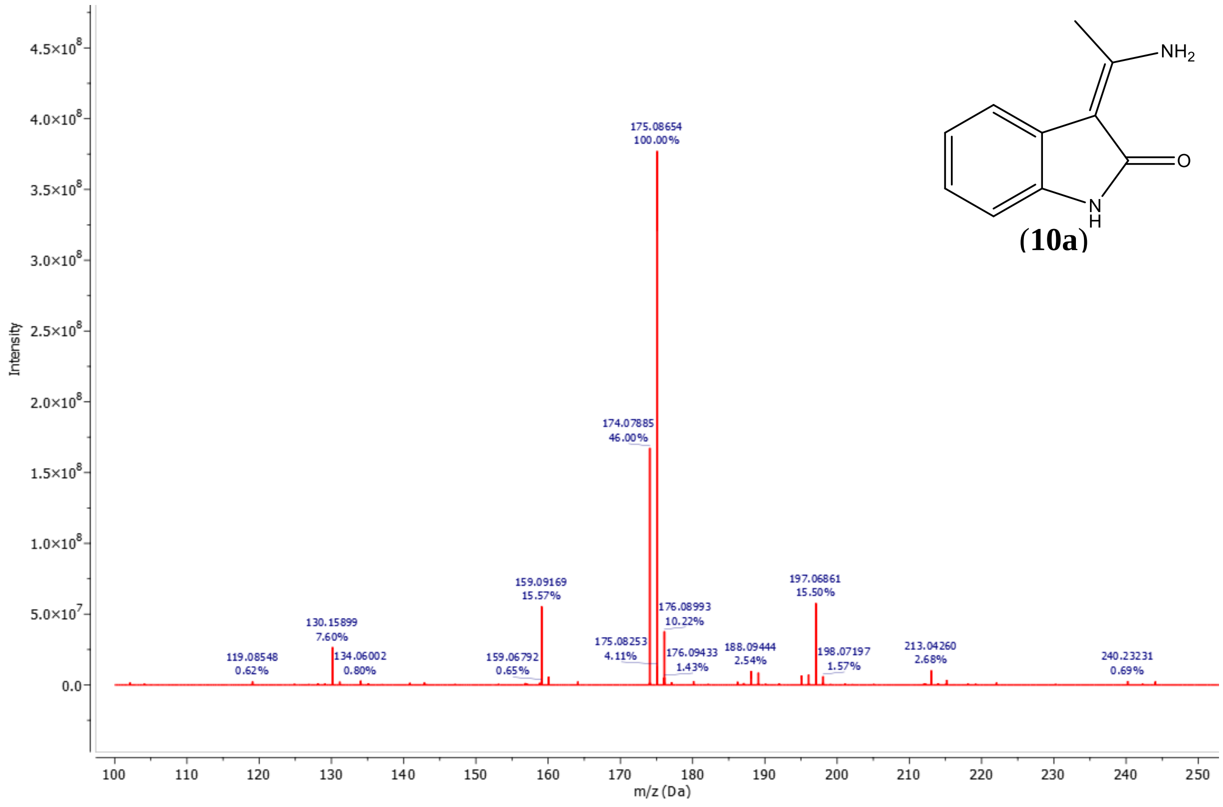

Intensity
m/z (Da)
175.08654
100.00%
174.07885
46.00%
159.09169
15.57%
197.06861
15.50%
176.08993
10.22%
130.15899
7.60%
175.08253
4.11%
188.09444
2.54%
119.08548
0.62%
134.06002
0.80%
159.06792
0.65%
176.09433
1.43%
198.07197
1.57%
213.04260
2.68%
240.23231
0.69%
NH_2
O
N
H

(10a)

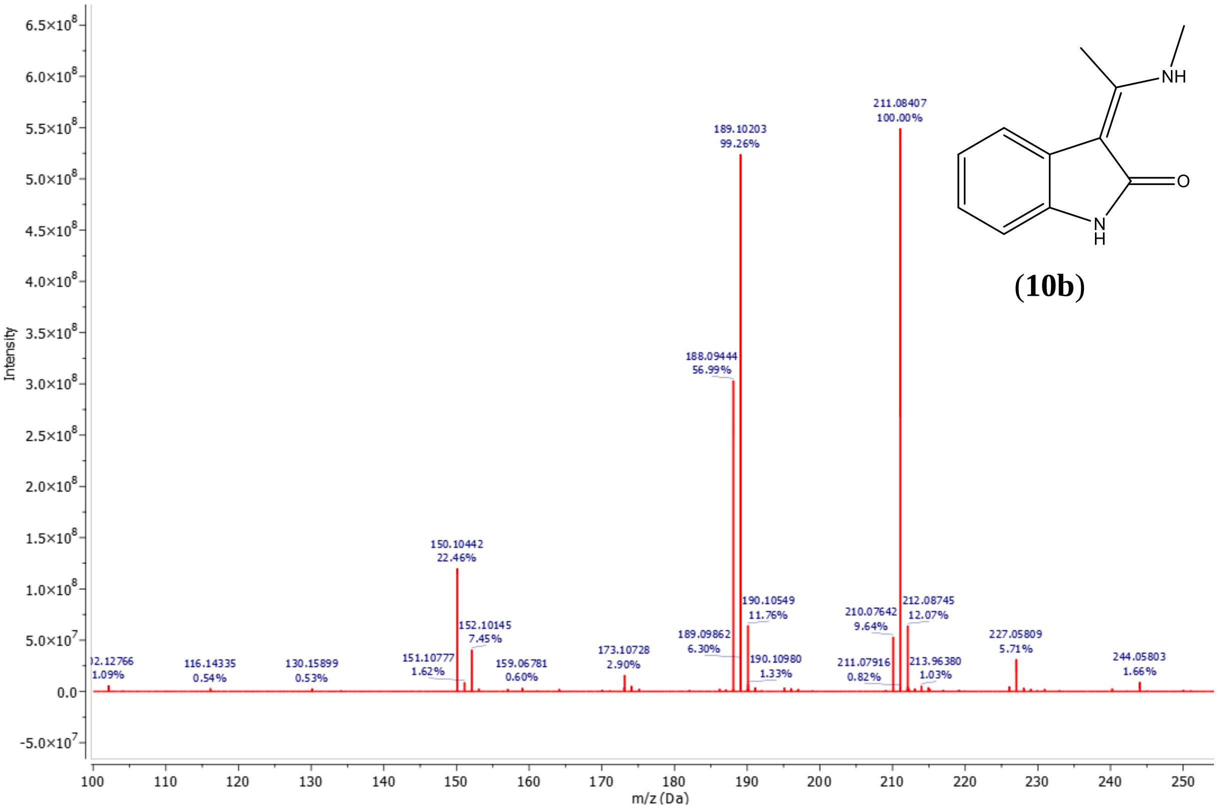

Intensity
m/z (Da)
)2.12766 1.09%
116.14335 0.54%
130.15899 0.53%
151.10777 1.62%
150.10442 22.46%
152.10145 7.45%
159.06781 0.60%
173.10728 2.90%
189.09862 6.30%
188.09444 56.99%
189.10203 99.26%
190.10549 11.76%
190.10980 1.33%
210.07642 9.64%
211.07916 0.82%
211.08407 100.00%
212.08745 12.07%
213.96380 1.03%
227.05809 5.71%
244.05803 1.66%
NH
O
N
H

(10b)

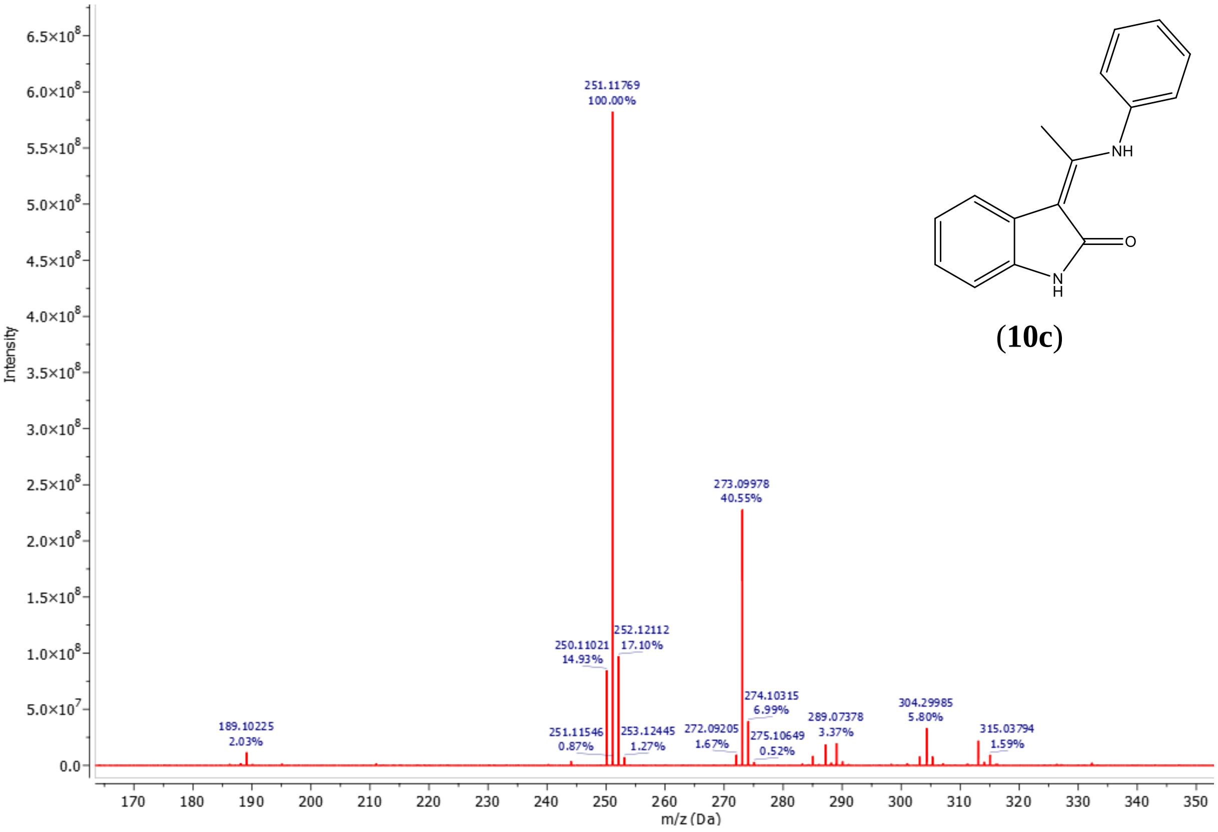

Intensity
m/z (Da)
189.10225 2.03%
251.11546 0.87%
250.11021 14.93%
251.11769 100.00%
252.12112 17.10%
253.12445 1.27%
272.09205 1.67%
273.09978 40.55%
274.10315 6.99%
275.10649 0.52%
289.07378 3.37%
304.29985 5.80%
315.03794 1.59%
NH
O
N
H

(10c)

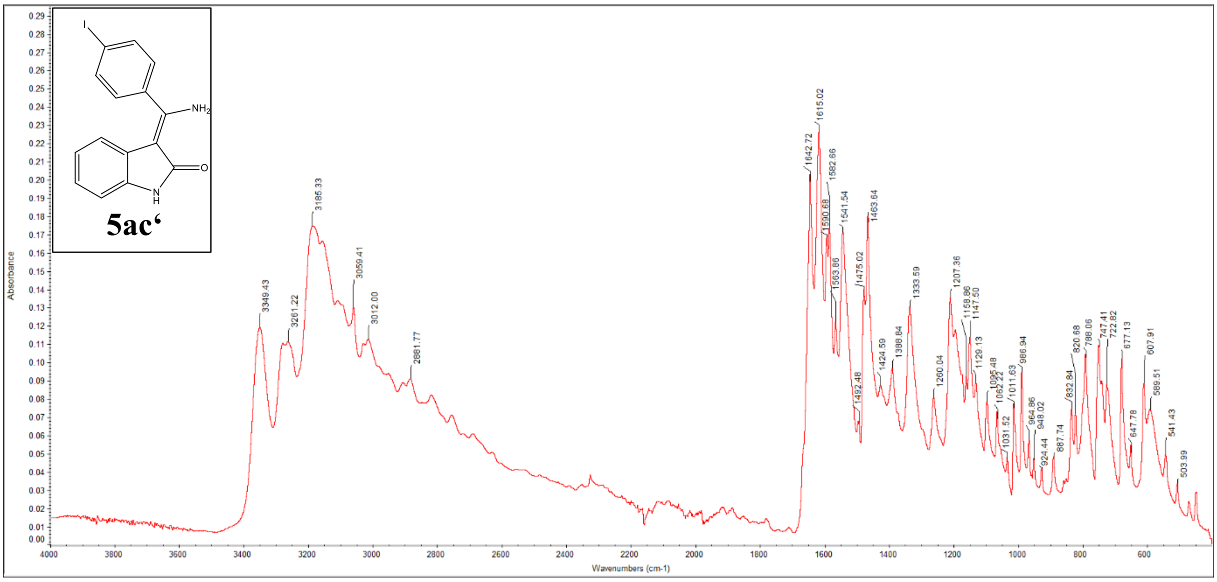
5ac‘
NH2
O
H
I
Absorbance
Wavenumbers (cm-1)

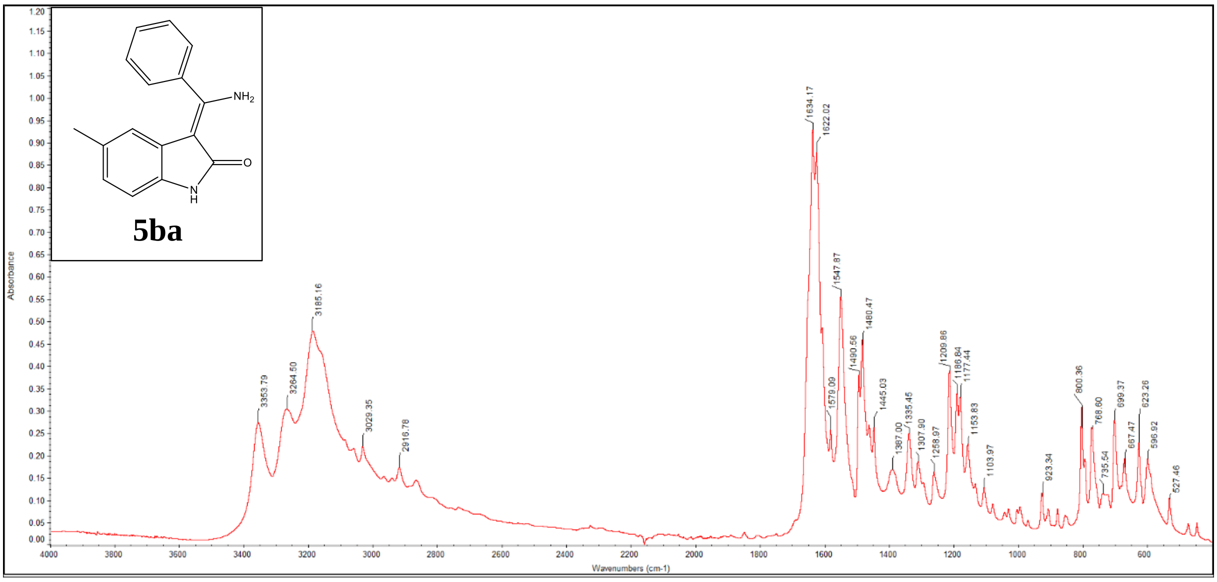
5ba
NH2
O
H
Absorbance
Wavenumbers (cm-1)

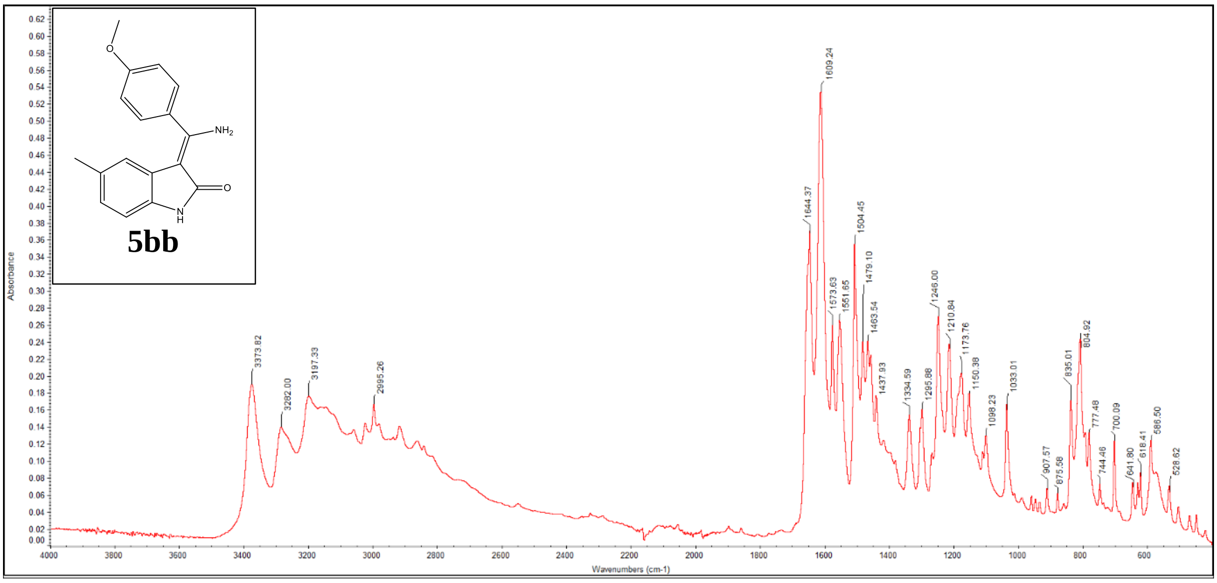
5bb
NH2
O
H
Absorbance
Wavenumbers (cm-1)

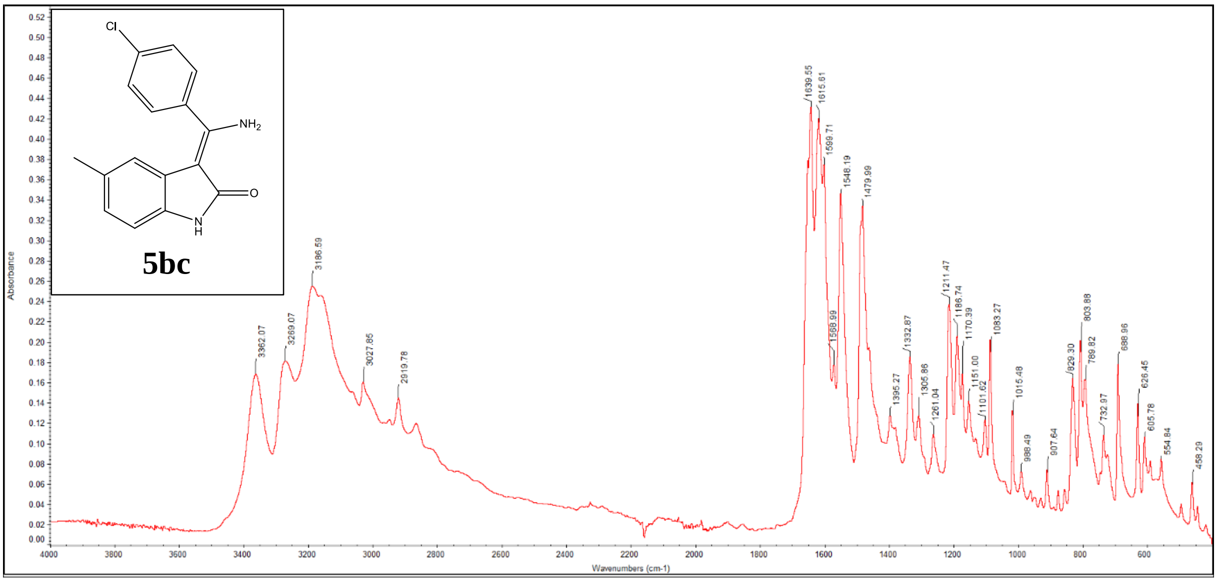
5bc
Absorbance
Wavenumbers (cm-1)
3362.07
3269.07
3186.59
3027.85
2919.78
1639.55
1615.61
1599.71
1548.19
1479.99
1568.99
1395.27
1332.87
1305.86
1261.04
1211.47
1186.74
1170.39
1151.00
1101.62
1083.27
1015.48
988.49
907.64
829.30
803.88
789.82
732.97
698.96
626.45
605.78
554.84
458.29

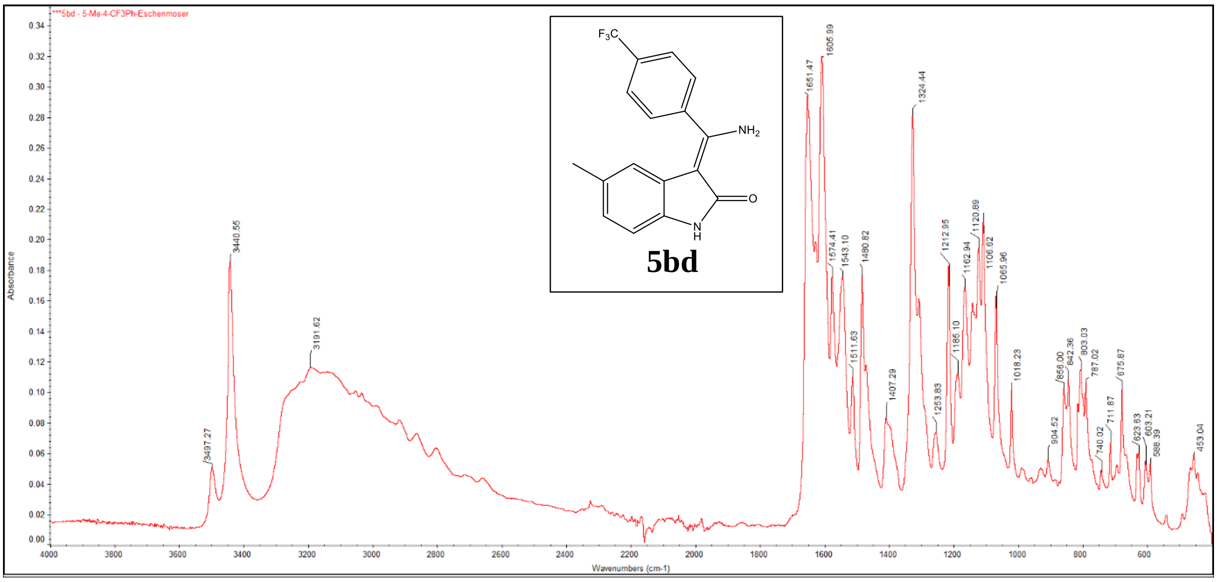
***5bd - 5-Me-4-CF3Ph-Eschenmoser
5bd
Absorbance
Wavenumbers (cm-1)
3497.27
3440.55
3191.62
1651.47
1605.99
1574.41
1543.10
1511.63
1480.82
1407.29
1324.44
1253.83
1212.95
1185.10
1162.94
1120.89
1106.62
1065.96
1018.23
904.52
856.00
842.36
803.03
787.02
740.02
711.87
675.87
623.63
603.21
588.39
453.04

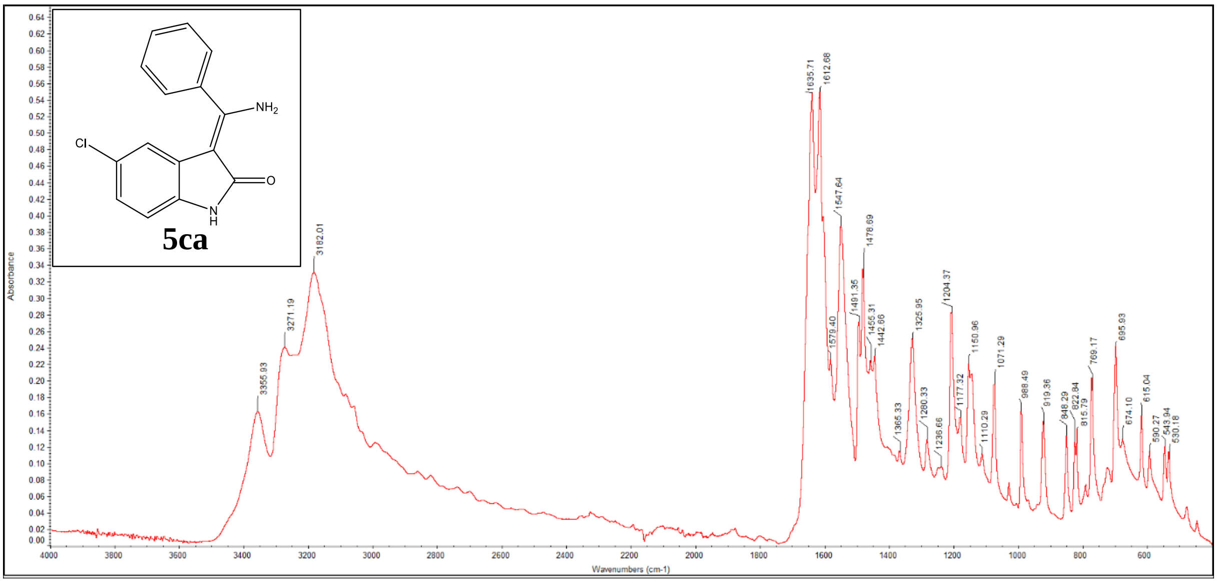
5ca
Absorbance
Wavenumbers (cm-1)
3355.93
3271.19
3182.01
1635.71
1612.68
1579.40
1547.64
1491.35
1476.69
1455.31
1442.66
1365.33
1325.95
1280.33
1236.66
1204.37
1177.32
1150.96
1110.29
1071.29
988.49
919.36
848.29
822.84
815.79
769.17
695.93
674.10
615.04
590.27
543.94
530.18

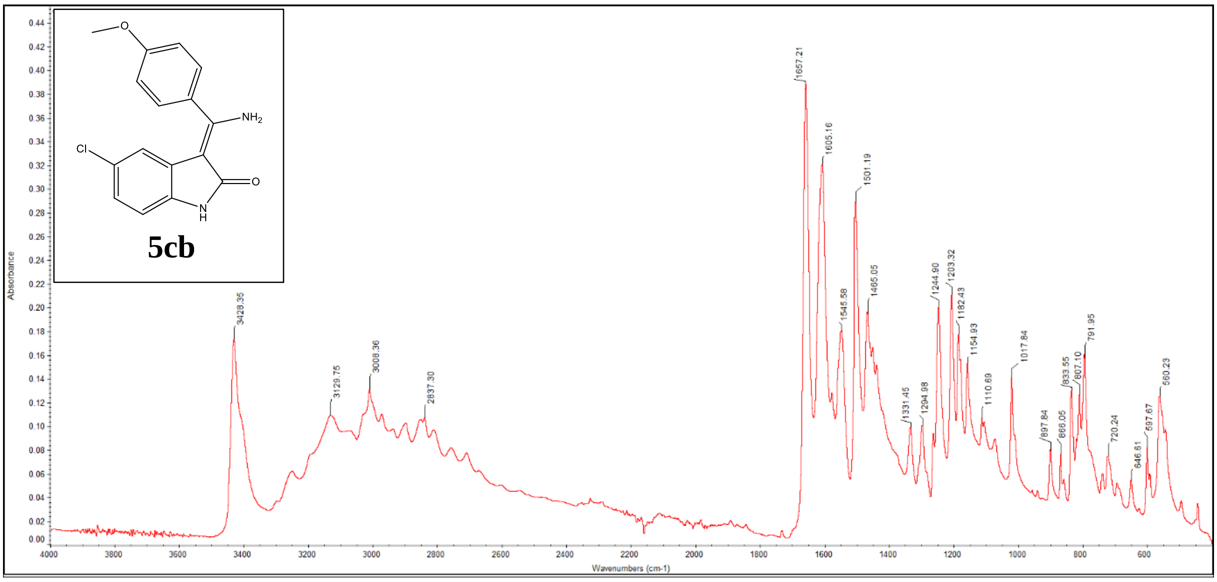
5cb
NH2
Cl
O
N
H
Absorbance
Wavenumbers (cm-1)
3428.35
3129.75
3008.36
2837.30
1657.21
1605.16
1545.58
1501.19
1465.05
1331.45
1294.98
1244.90
1203.32
1182.43
1154.93
1110.69
1017.84
897.84
866.05
833.55
807.10
791.95
720.24
646.61
597.67
560.23

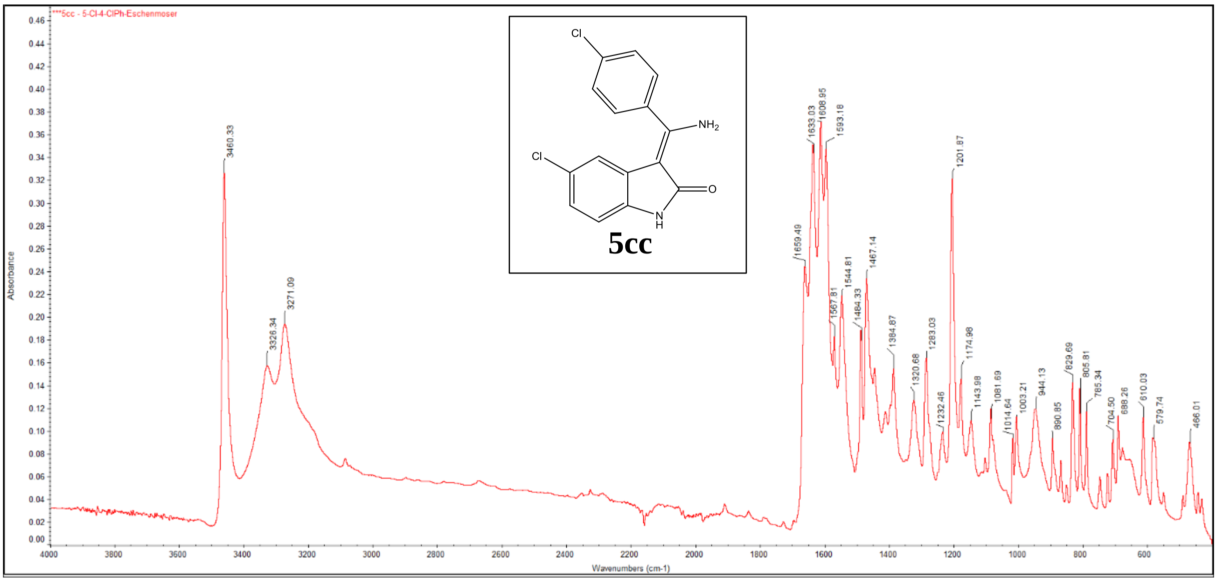
***5cc - 5-Cl-4-ClPh-Eschenmoser
5cc
Cl
NH2
Cl
O
N
H
Absorbance
Wavenumbers (cm-1)
3460.33
3326.34
3271.09
1659.49
1633.03
1608.95
1593.18
1567.81
1544.81
1484.33
1467.14
1384.87
1320.68
1283.03
1232.46
1201.87
1174.98
1143.98
1081.69
1014.64
1003.21
944.13
890.85
829.69
805.81
765.34
704.50
688.26
610.03
579.74
466.01

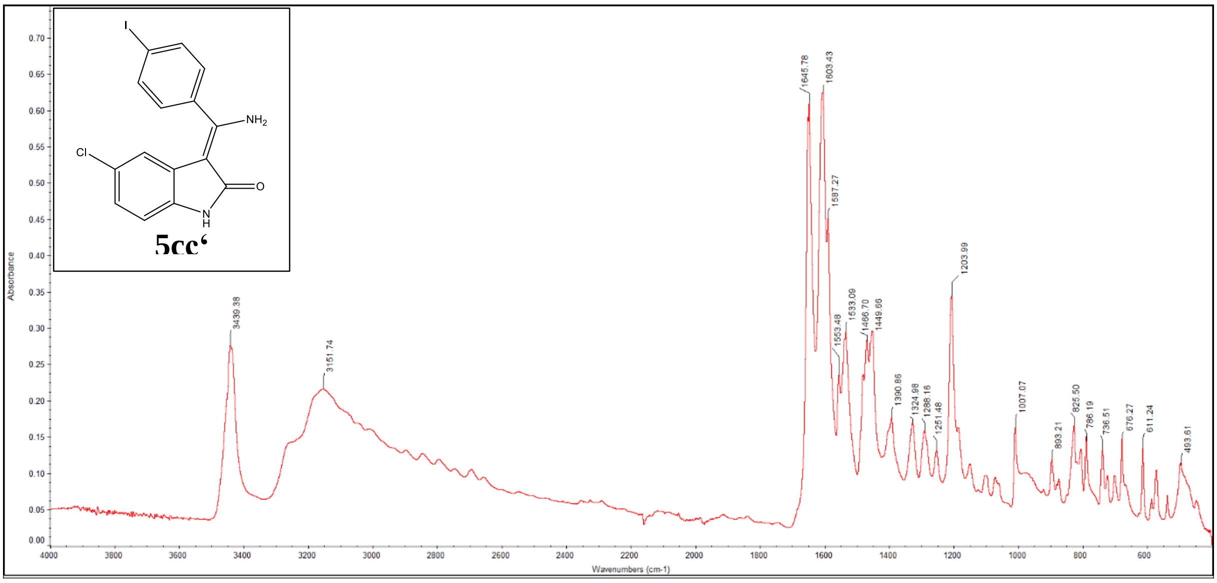
5cc‘
I
NH2
Cl
O
N
H
Absorbance
Wavenumbers (cm-1)
3439.38
3151.74
1645.78
1603.43
1587.27
1553.48
1533.09
1466.70
1449.66
1390.86
1324.98
1288.16
1251.48
1203.99
1007.07
893.21
826.50
786.19
736.51
676.27
611.24
493.61

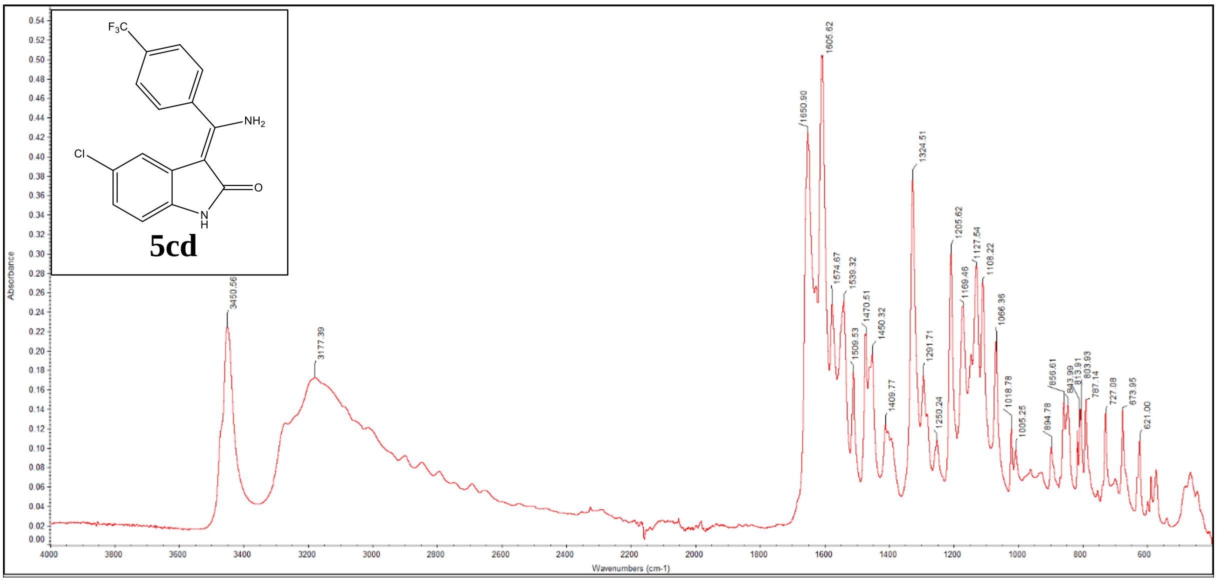
5cd
Absorbance
Wavenumbers (cm-1)
3450.56
3177.39
1650.90
1605.82
1574.67
1539.32
1509.53
1470.51
1450.32
1409.77
1324.51
1291.71
1250.24
1205.62
1169.46
1127.54
1108.22
1066.36
1018.78
1005.25
894.78
856.61
843.99
813.91
803.93
787.14
727.08
673.95
621.00

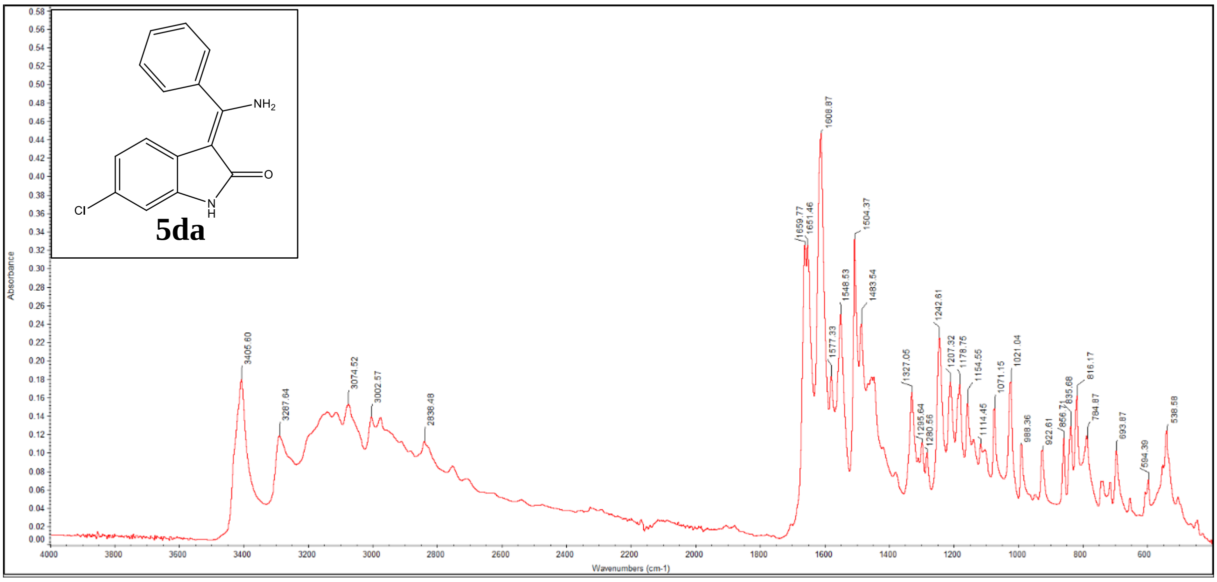
5da
Absorbance
Wavenumbers (cm-1)
3405.60
3287.64
3074.52
3002.57
2838.48
1659.77
1651.46
1608.87
1577.33
1548.53
1504.37
1483.54
1327.05
1295.64
1280.56
1242.61
1207.32
1178.75
1154.55
1114.45
1071.15
1021.04
988.36
922.61
856.71
835.68
816.17
784.87
693.87
594.39
538.58

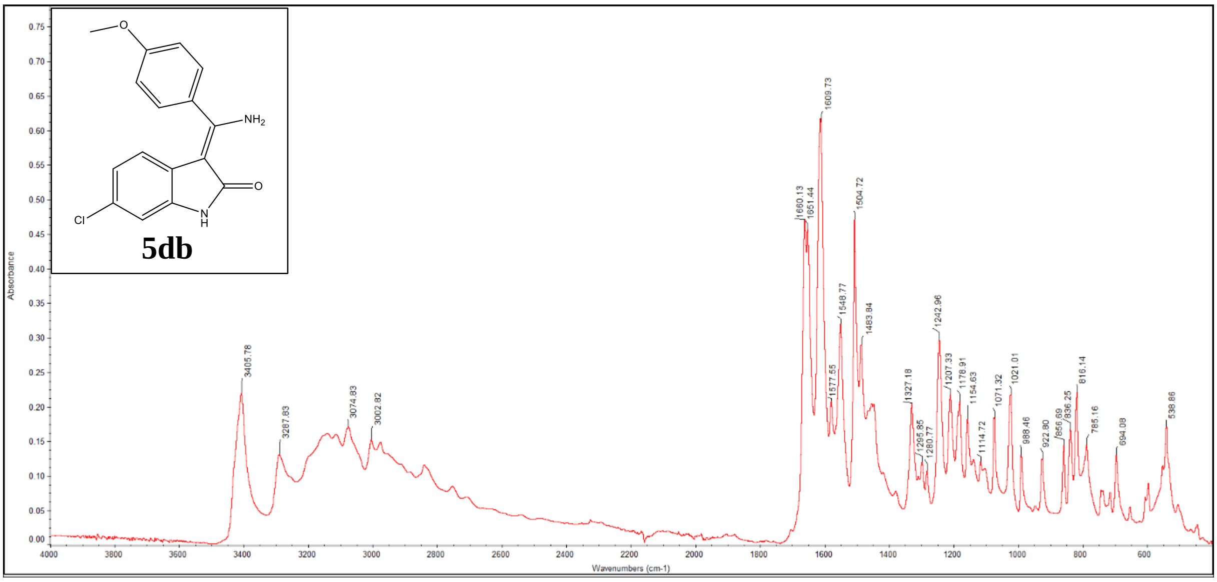
5db
Absorbance
Wavenumbers (cm-1)
3405.78
3287.83
3074.83
3002.82
1660.13
1651.44
1609.73
1577.55
1548.77
1504.72
1483.84
1327.18
1295.85
1280.77
1242.96
1207.33
1178.91
1154.63
1114.72
1071.32
1021.01
988.46
922.80
856.69
836.25
816.14
785.16
694.08
538.86

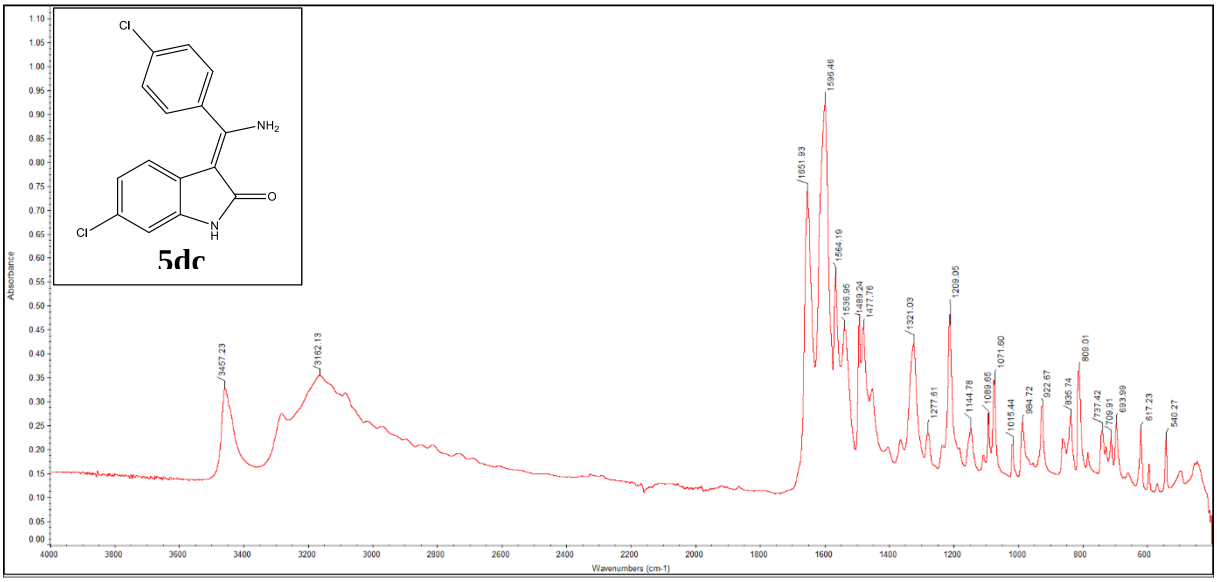
5dc
Absorbance
Wavenumbers (cm-1)

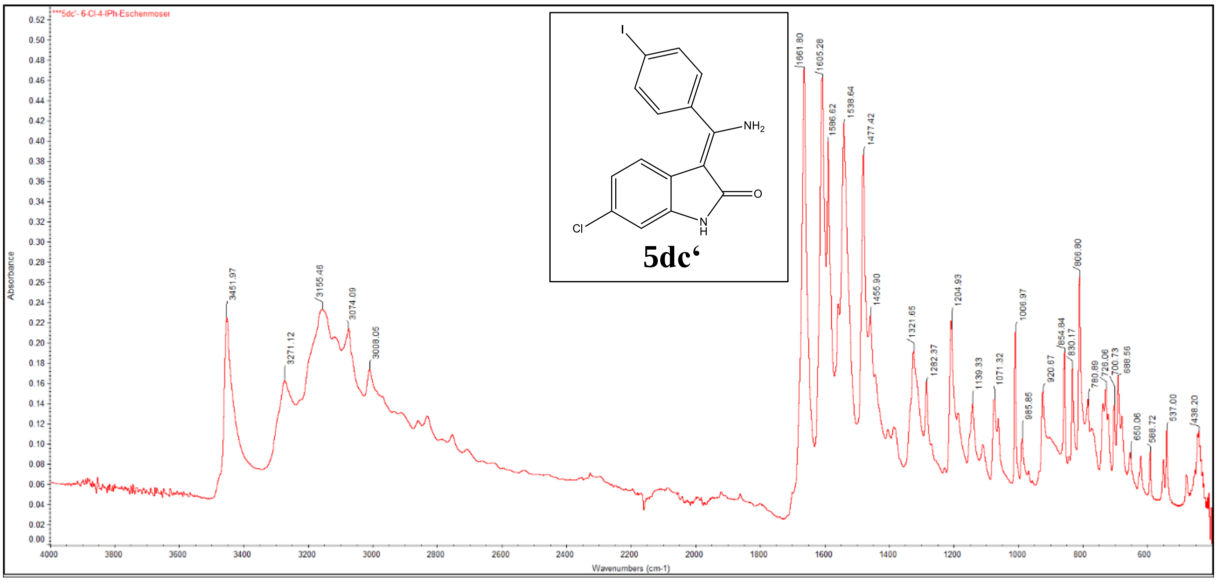
5dc'
Absorbance
Wavenumbers (cm-1)

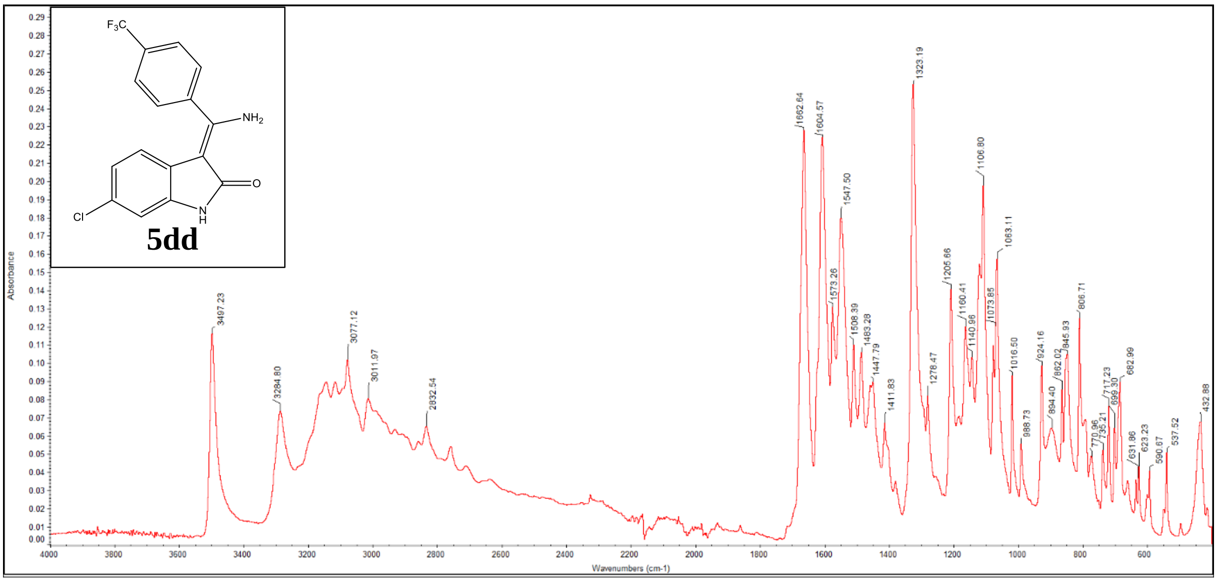
5dd
Absorbance
Wavenumbers (cm-1)

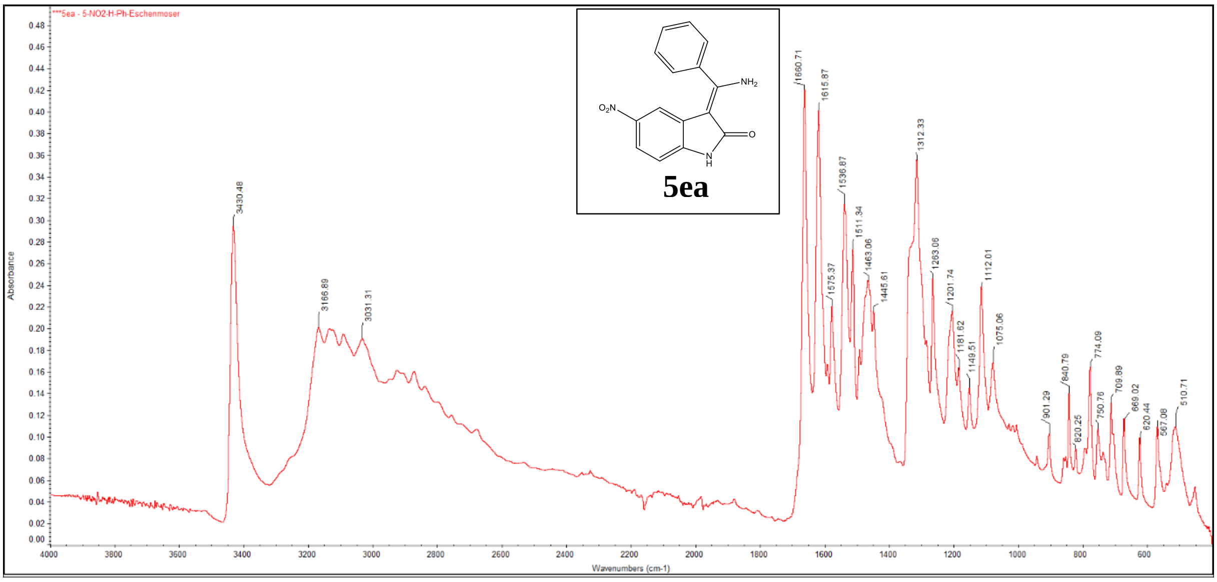
***5ea - 5-NO2-H-Ph-Eschenmoser
5ea
Absorbance
Wavenumbers (cm-1)

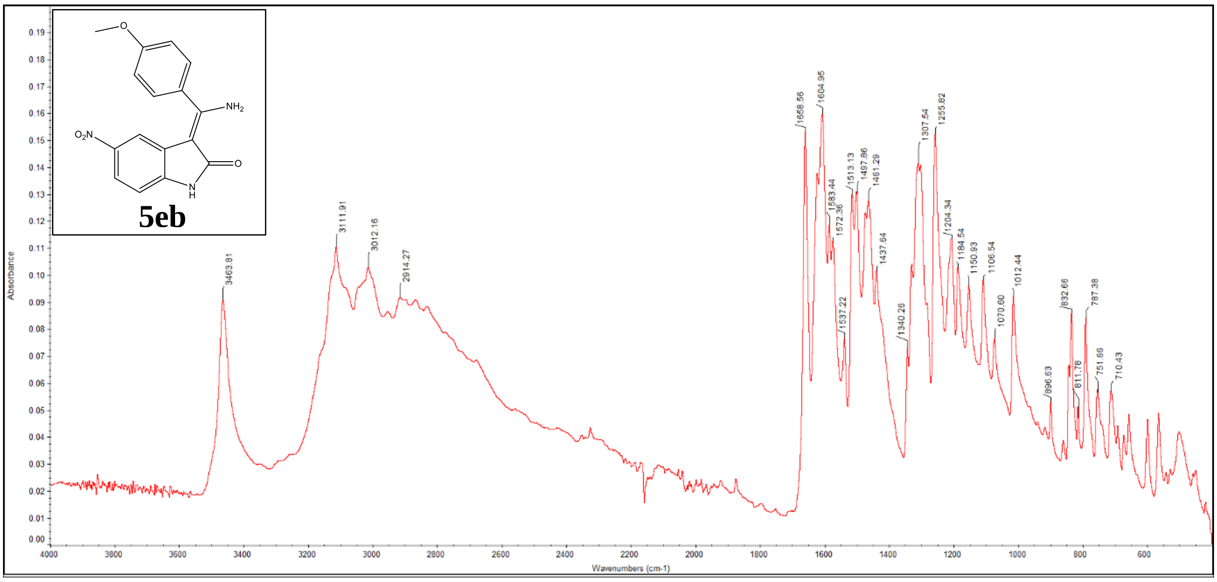
5eb
Absorbance
Wavenumbers (cm-1)

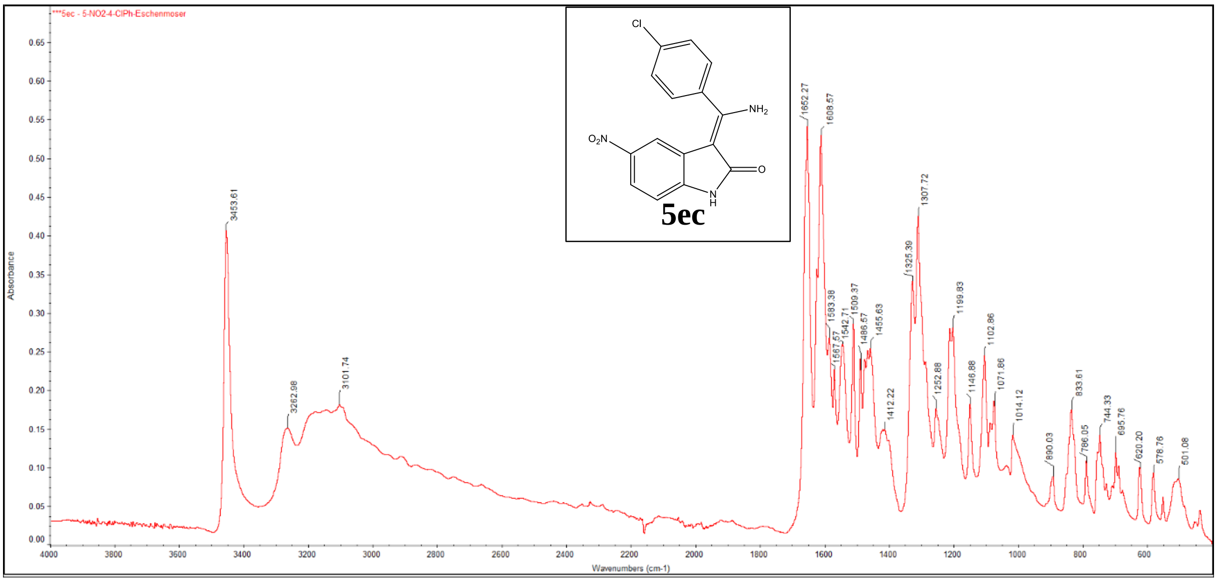
***5ec - 5-NO2-4-ClPh-Eschenmoser
5ec
Absorbance
Wavenumbers (cm-1)

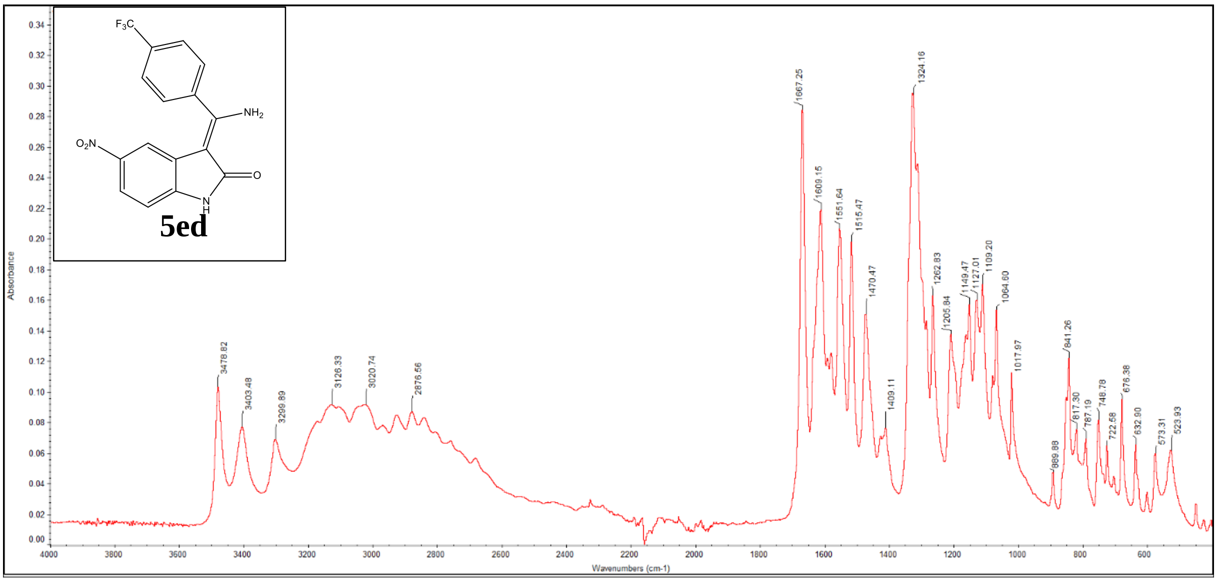
5ed
Absorbance
Wavenumbers (cm-1)

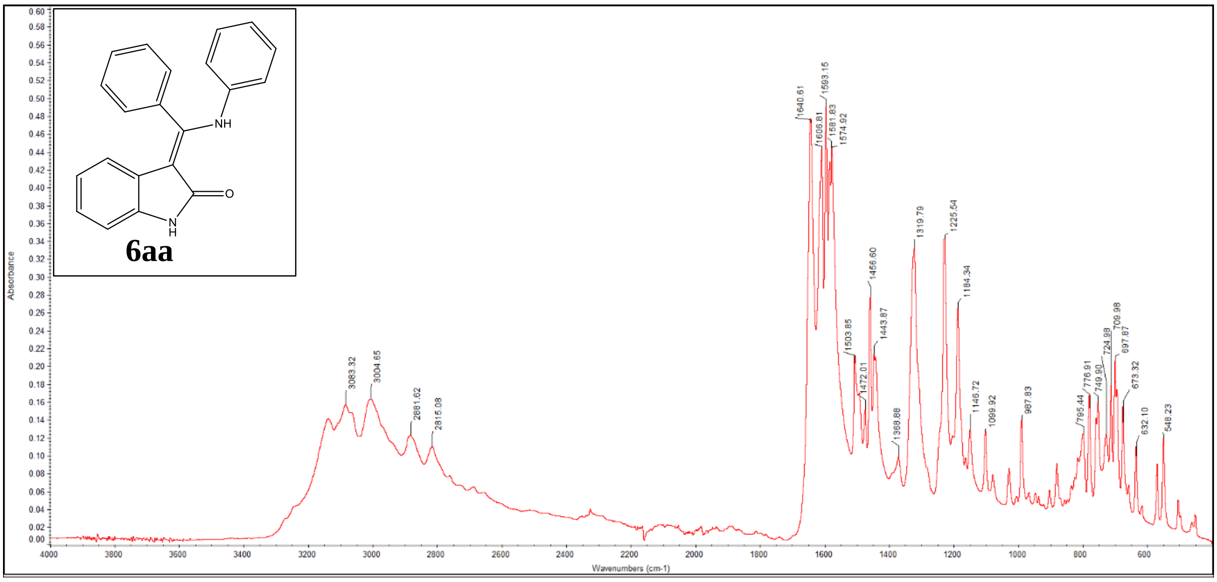
6aa
Absorbance
Wavenumbers (cm-1)

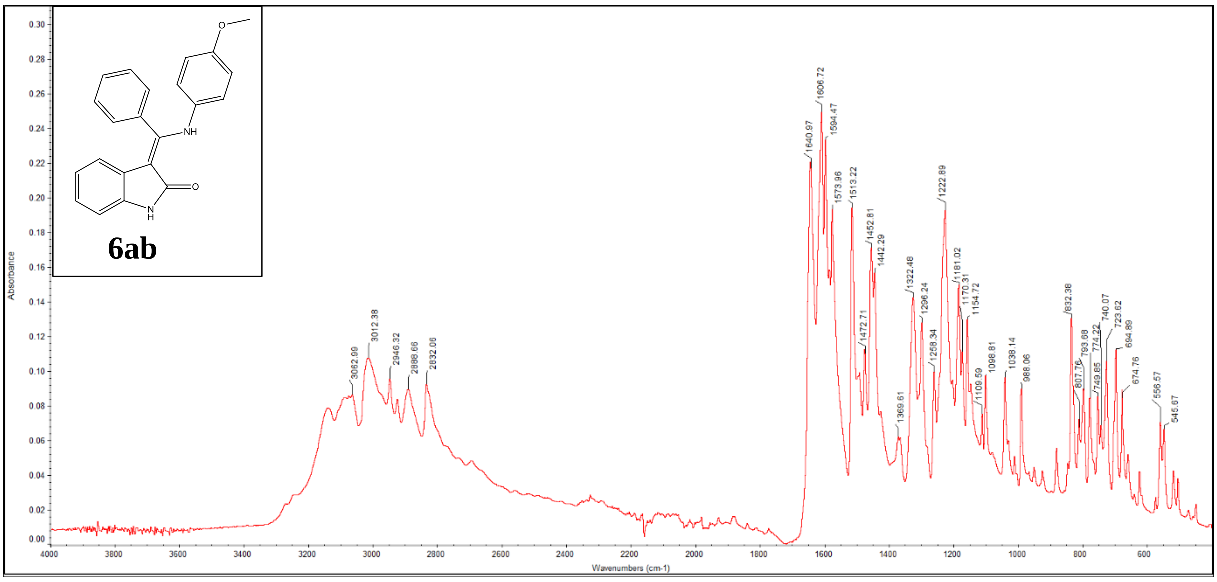
6ab
Absorbance
Wavenumbers (cm-1)

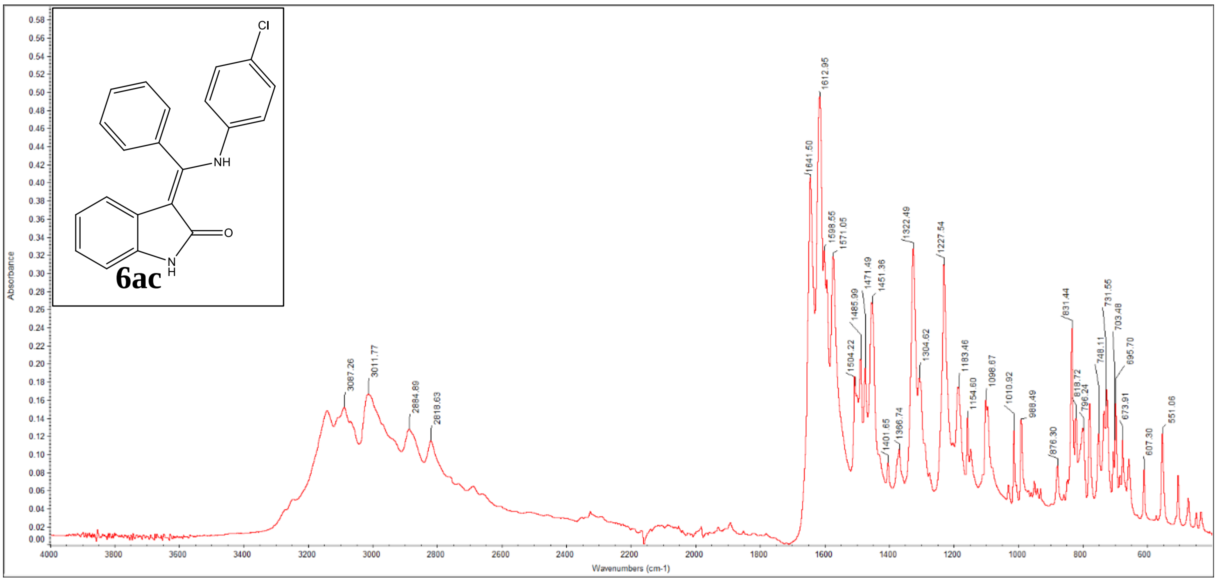
6ac
Absorbance
Wavenumbers (cm-1)

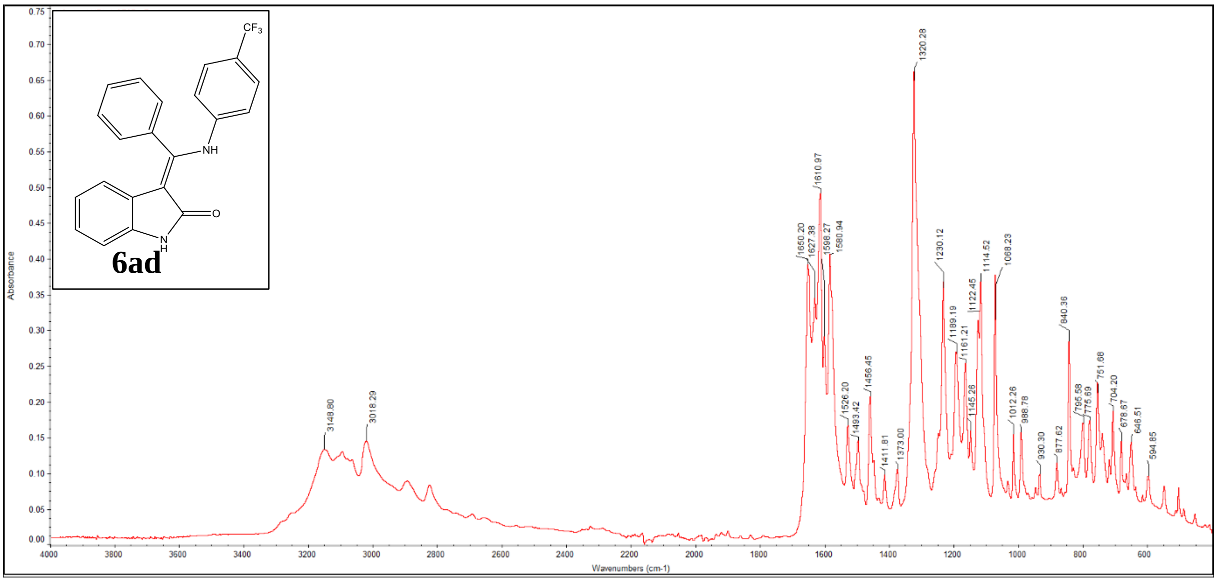
6ad
Absorbance
Wavenumbers (cm-1)

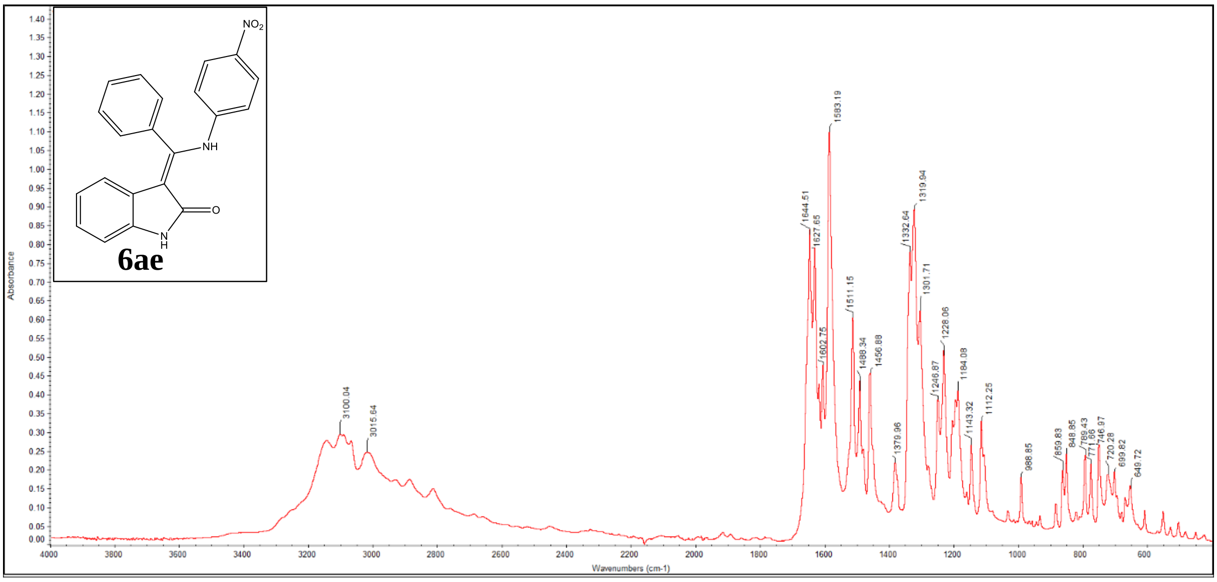
6ae
Absorbance
Wavenumbers (cm-1)

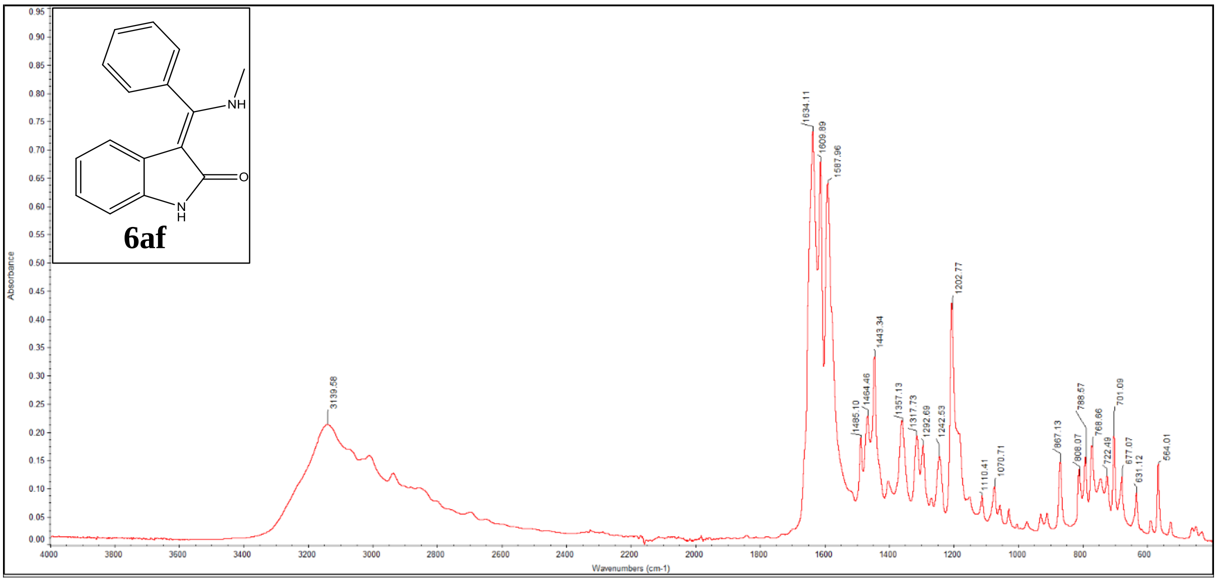

6af
NH
O
N
H
Absorbance
Wavenumbers (cm-1)
3139.58
1634.11
1609.89
1587.96
1485.10
1464.46
1443.34
1357.13
1317.73
1292.69
1242.53
1202.77
1110.41
1070.71
867.13
808.07
788.57
768.66
722.49
701.09
677.07
631.12
564.01

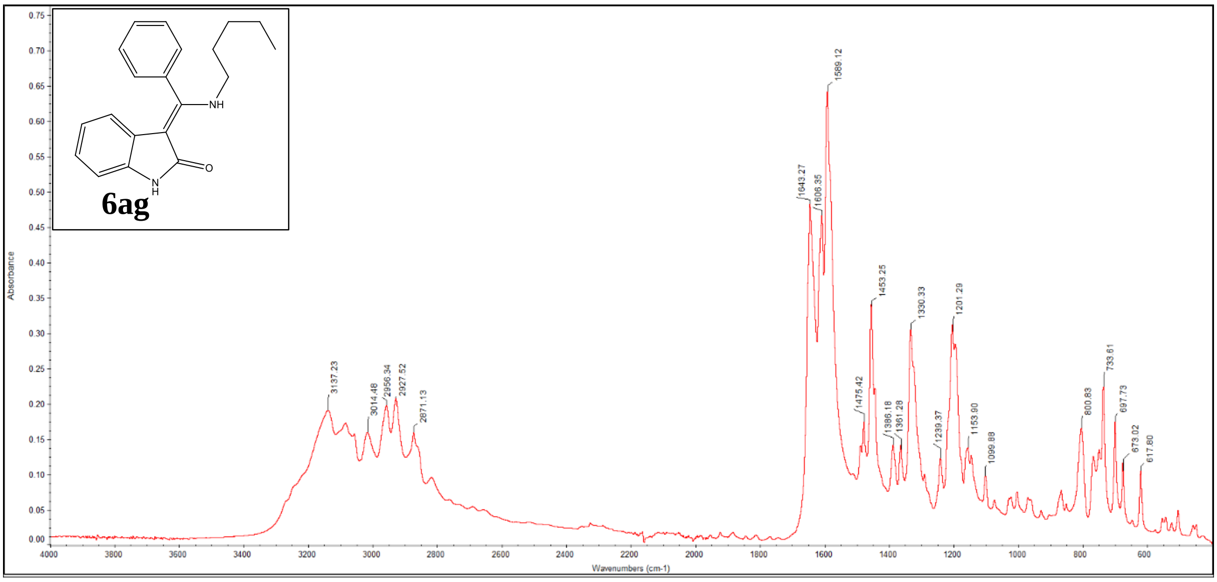

6ag
NH
O
N
H
Absorbance
Wavenumbers (cm-1)
3137.23
3014.48
2956.34
2927.52
2871.13
1643.27
1606.35
1589.12
1475.42
1453.25
1386.18
1361.28
1330.33
1239.37
1201.29
1153.90
1099.88
800.83
733.61
697.73
673.02
617.80

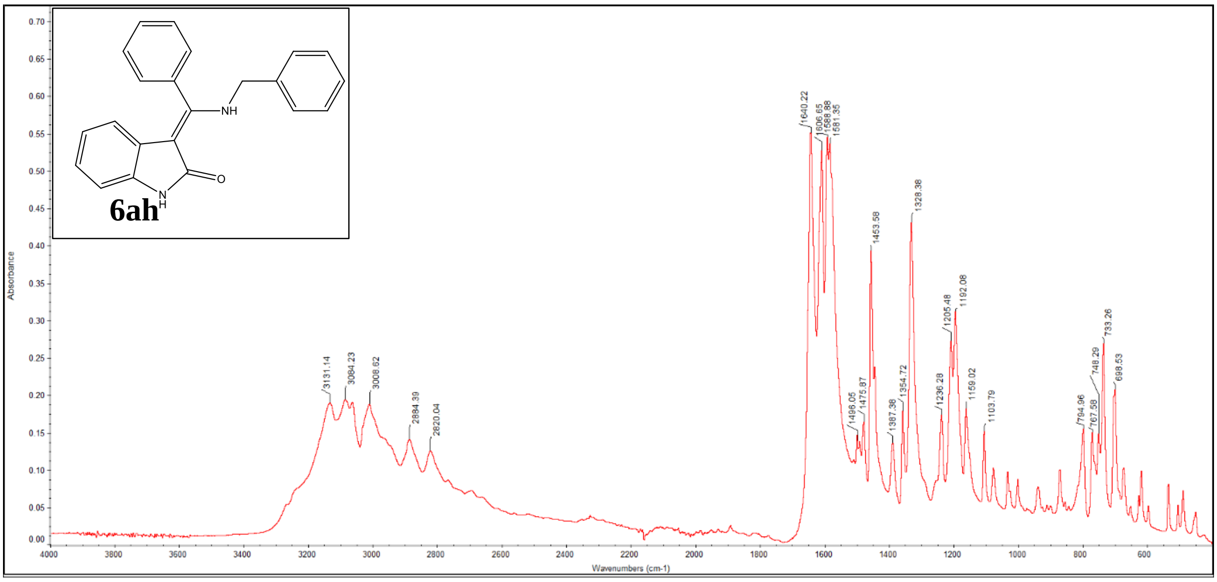

6ah
NH
O
N
H
Absorbance
Wavenumbers (cm-1)
3131.14
3084.23
3008.62
2884.39
2820.04
1640.22
1606.65
1588.88
1581.35
1496.05
1475.87
1453.58
1387.38
1354.72
1328.38
1236.28
1205.48
1192.08
1159.02
1103.79
794.96
767.58
748.29
733.26
698.53

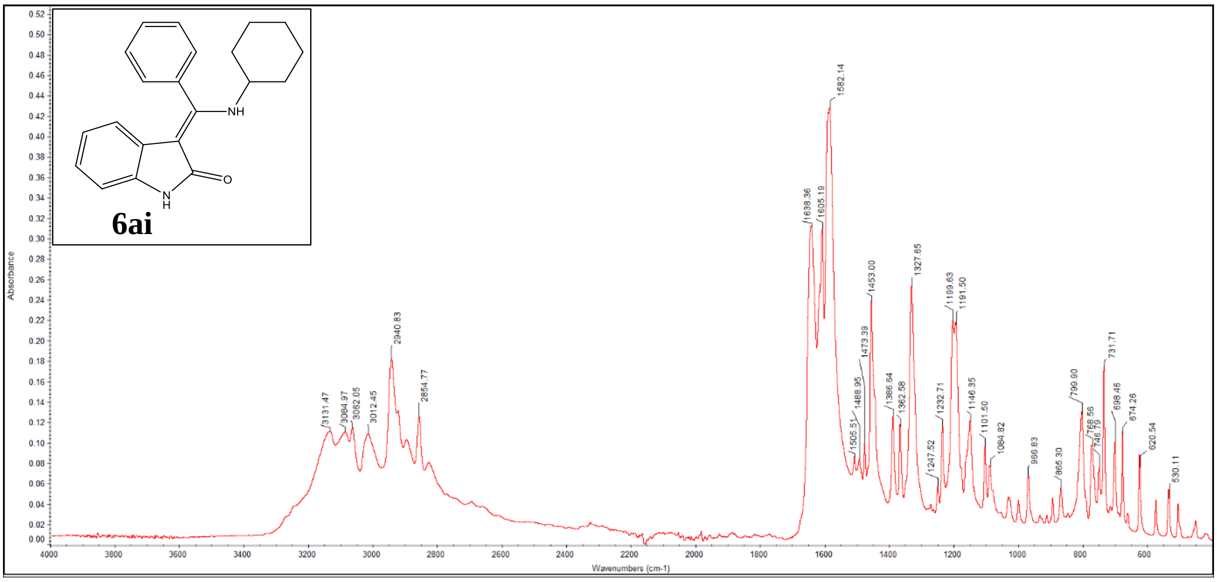
6ai
NH
N
H
O
Absorbance
Wavenumbers (cm-1)
1582.14
1638.36
1605.19
1327.65
1453.00
1199.63
1191.50
2940.83
731.71
1473.39
3131.47
3084.97
3062.05
3012.45
2854.77
799.90
1488.95
1386.64
1362.58
1232.71
1146.35
698.46
674.28
1505.51
1101.50
768.56
746.19
1084.82
966.83
865.30
620.54
1247.52
530.11

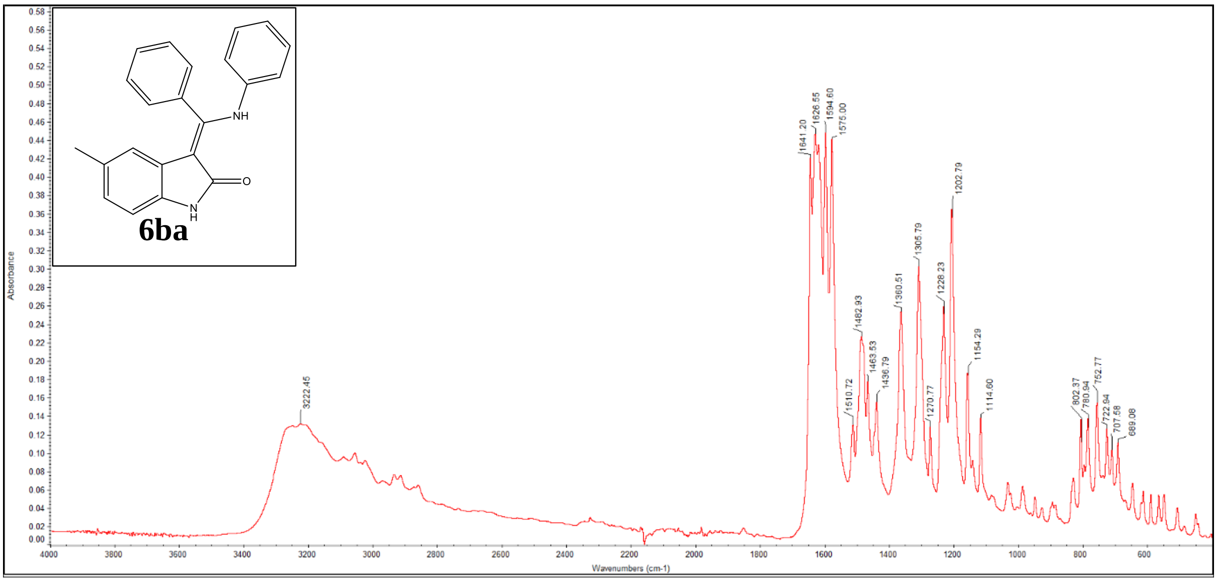
6ba
NH
N
H
O
Absorbance
Wavenumbers (cm-1)
1641.20
1626.55
1594.60
1575.30
1202.79
1305.79
1360.51
1228.23
1482.93
1463.53
1436.79
1154.29
3222.45
1510.72
1270.77
1114.60
802.37
780.94
752.77
722.94
707.58
689.08

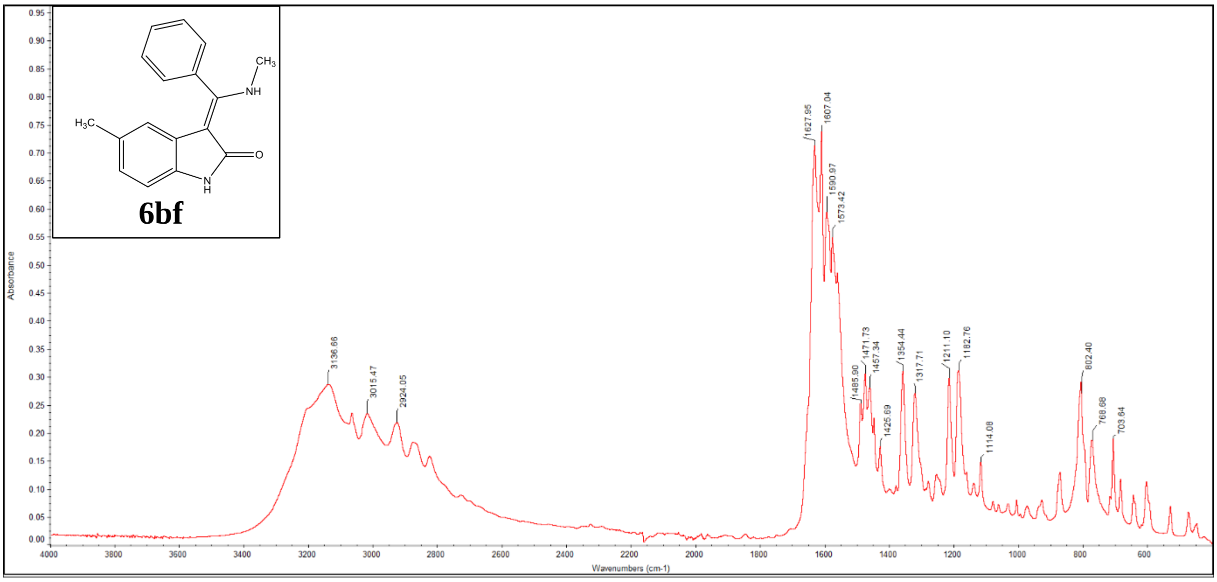
6bf
CH3
NH
H3C
N
H
O
Absorbance
Wavenumbers (cm-1)
1627.95
1607.04
1590.97
1573.42
1471.73
1457.34
1354.44
1211.10
1182.76
1485.90
1317.71
802.40
3136.66
3015.47
2924.05
1425.69
768.68
703.64
1114.08

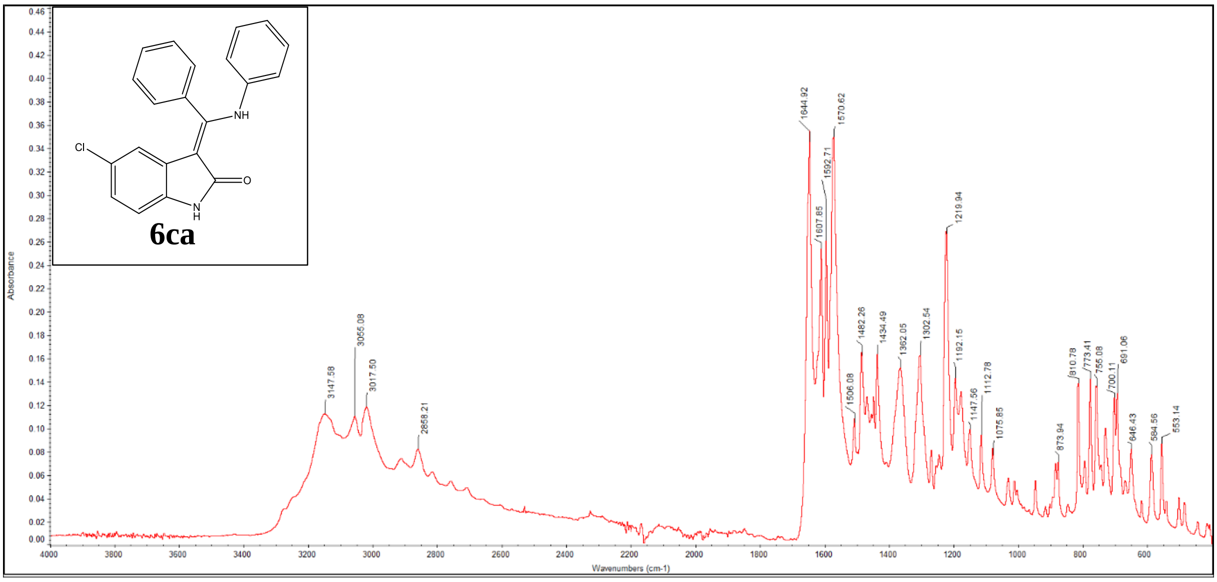
6ca
Cl
NH
O
N
H
Absorbance
Wavenumbers (cm-1)
3147.58
3055.08
3017.50
2858.21
1644.92
1607.85
1592.71
1570.62
1506.08
1482.26
1434.49
1362.05
1302.54
1219.94
1192.15
1147.56
1112.78
1075.85
873.94
810.78
773.41
755.08
700.11
691.06
646.43
584.56
553.14

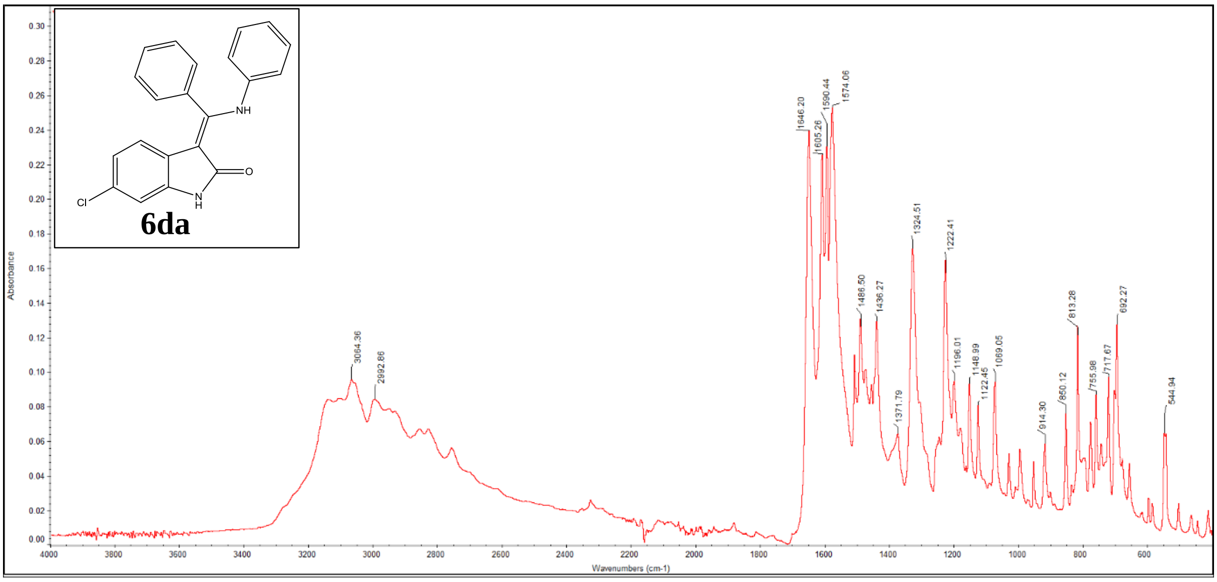
6da
Cl
NH
O
N
H
Absorbance
Wavenumbers (cm-1)
3064.36
2992.86
1646.20
1605.26
1590.44
1574.06
1486.50
1436.27
1371.79
1324.51
1222.41
1196.01
1148.99
1122.45
1069.05
914.30
850.12
813.28
755.98
717.67
692.27
544.94

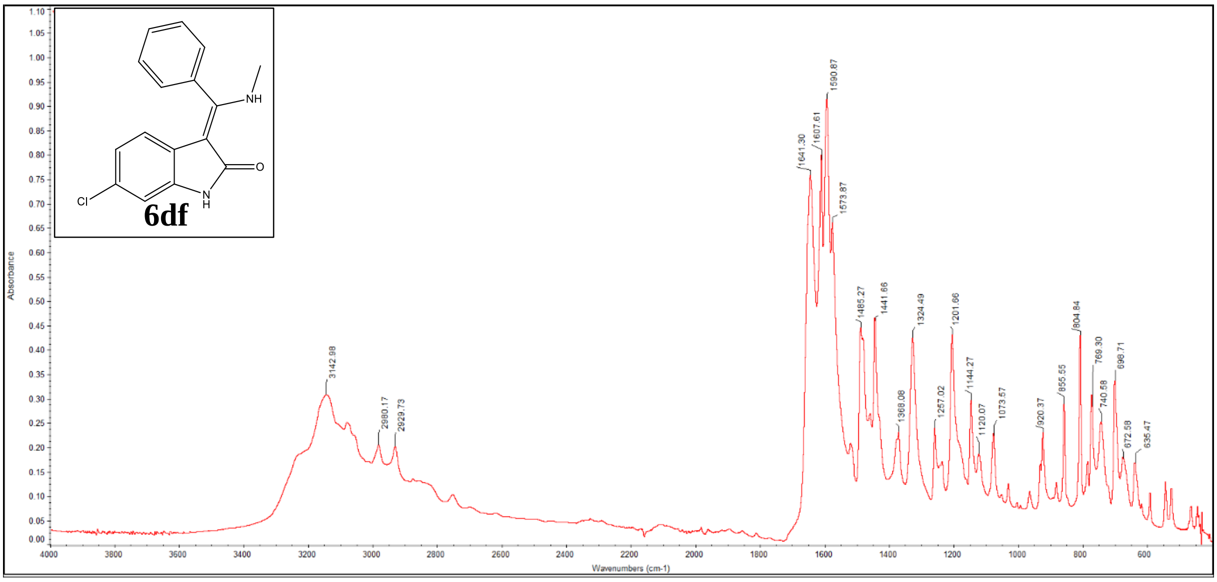
6df
Cl
NH
O
N
H
Absorbance
Wavenumbers (cm-1)
3142.98
2980.17
2929.73
1641.30
1607.61
1590.87
1573.87
1485.27
1441.66
1368.08
1324.49
1257.02
1201.66
1144.27
1120.07
1073.57
920.37
855.55
804.84
769.30
740.58
698.71
672.59
635.47

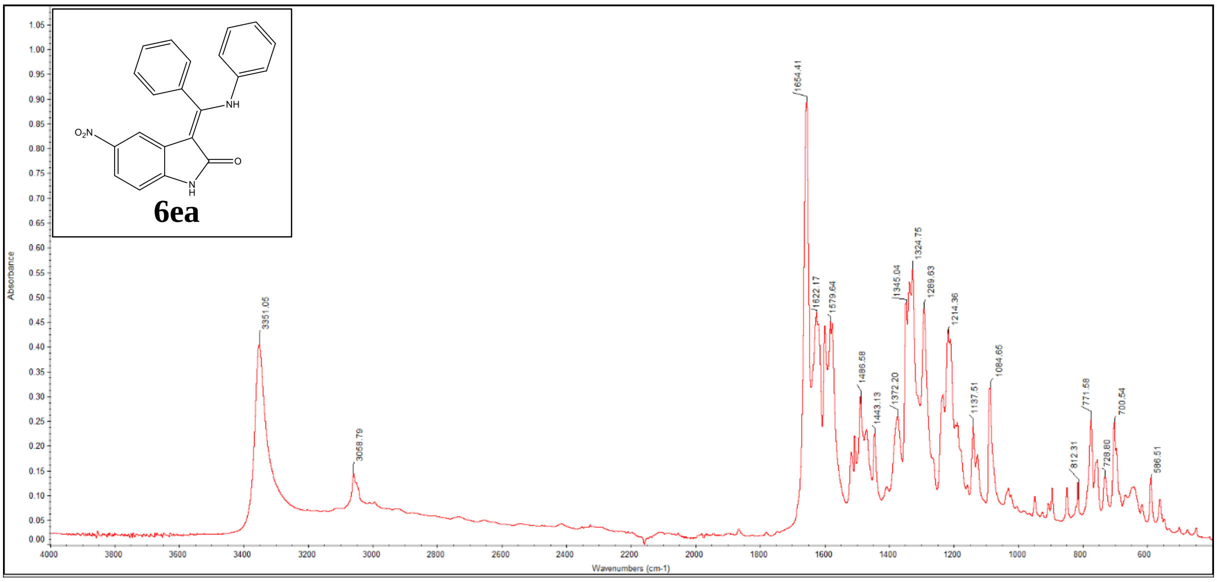

6ea
O_2N
NH
H
Absorbance
Wavenumbers (cm-1)
3351.05
3058.79
1654.41
1622.17
1579.64
1496.58
1443.13
1372.20
1345.04
1324.75
1289.63
1214.36
1137.51
1084.65
812.31
771.58
728.80
700.54
586.51

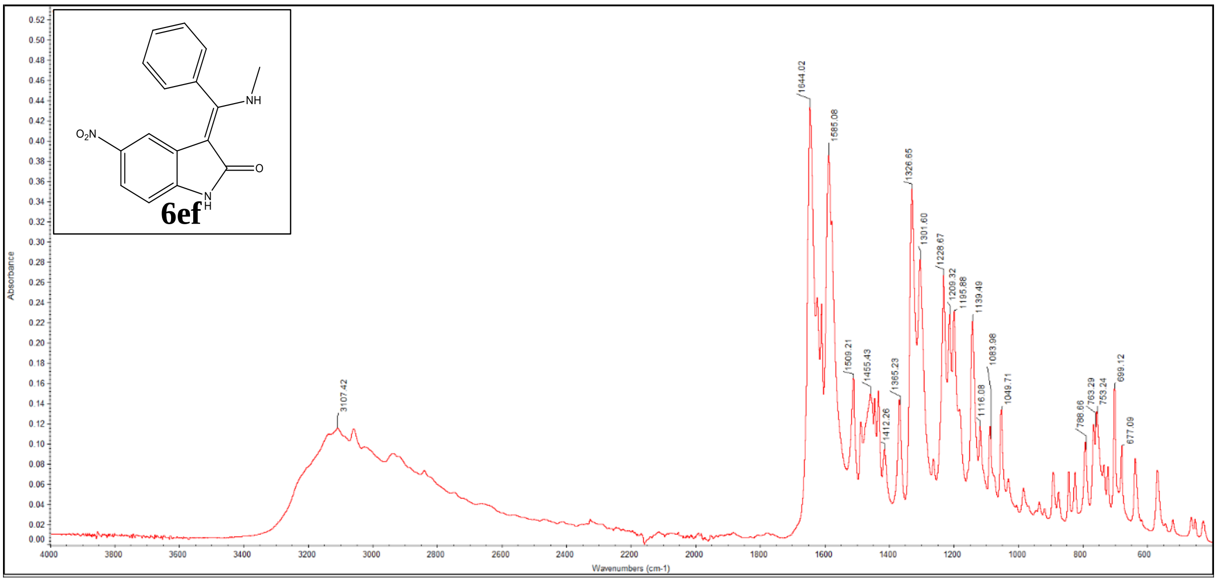

6ef
O_2N
NH
H
Absorbance
Wavenumbers (cm-1)
3107.42
1644.02
1585.08
1509.21
1455.43
1412.26
1365.23
1326.65
1301.60
1228.67
1209.32
1195.88
1139.49
1116.08
1083.98
1049.71
788.66
763.29
753.24
699.12
677.09

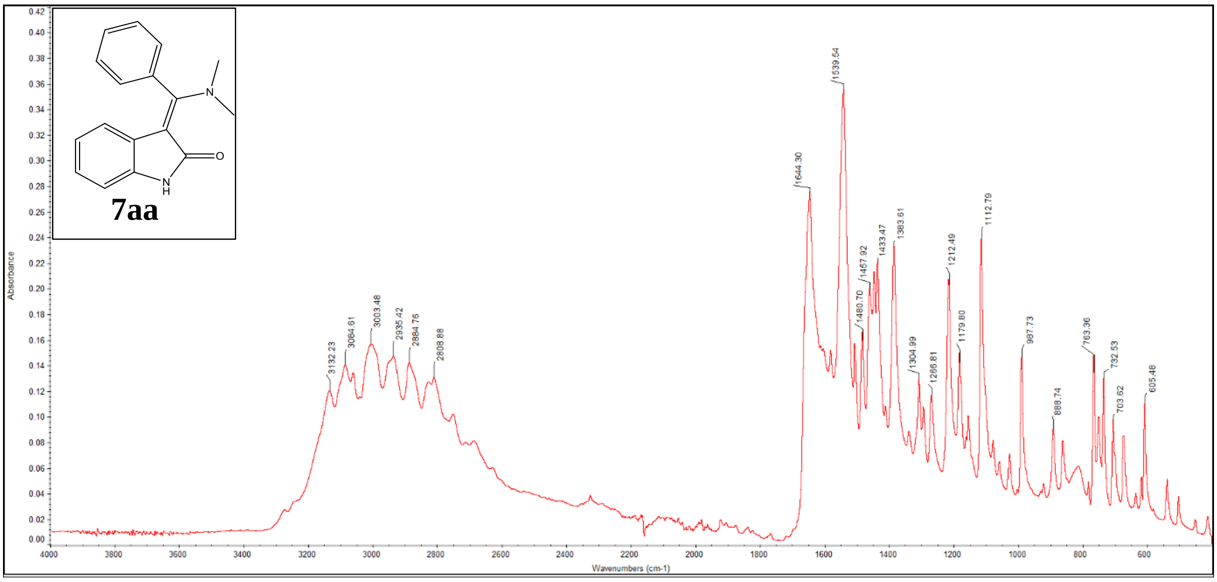

7aa
N
H
Absorbance
Wavenumbers (cm-1)
3132.23
3064.61
3003.48
2935.42
2884.76
2808.88
1644.30
1539.54
1480.70
1457.92
1433.47
1383.61
1304.99
1266.81
1212.49
1179.80
1112.79
987.73
888.74
763.36
732.53
703.62
605.48

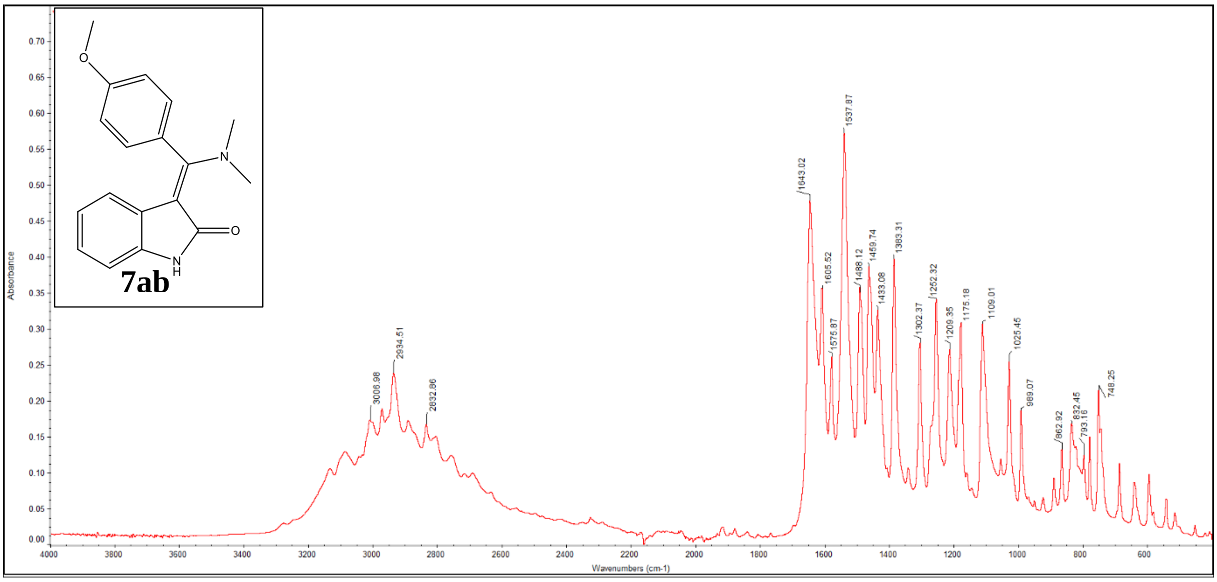
7ab
Absorbance
Wavenumbers (cm-1)
1643.02
1537.87
1605.52
1575.87
1488.12
1459.74
1433.08
1383.31
1302.37
1252.32
1209.35
1175.18
1109.01
1025.45
989.07
862.92
832.45
793.16
748.25
3006.98
2934.51
2832.86

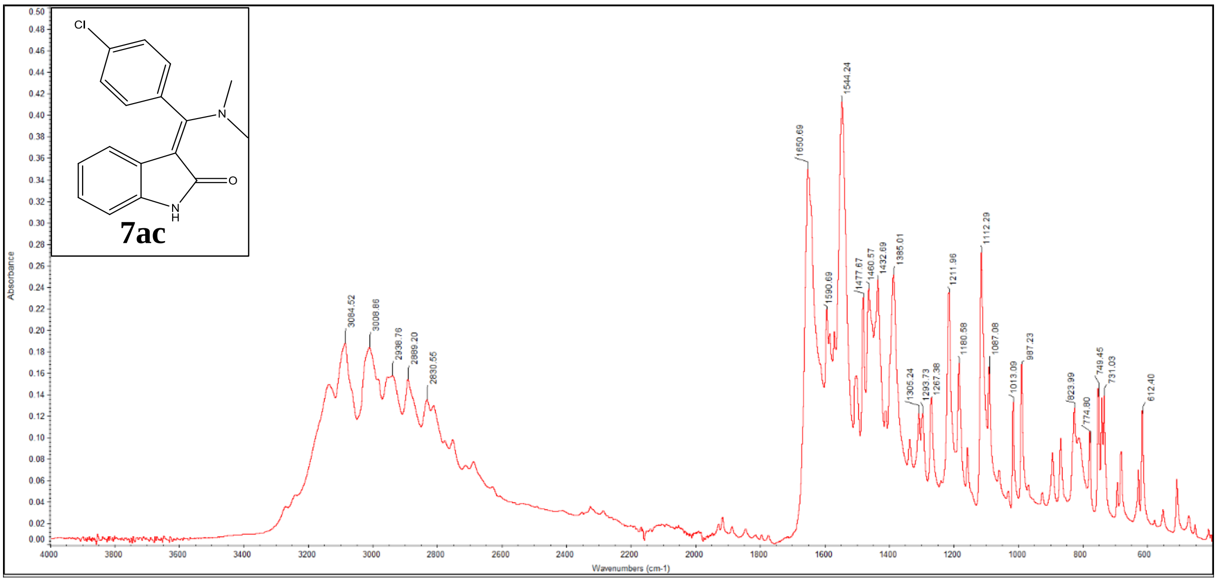
7ac
Absorbance
Wavenumbers (cm-1)
1650.69
1544.24
1590.69
1477.67
1460.57
1432.69
1385.01
1305.24
1293.73
1267.38
1211.96
1180.58
1112.29
1087.08
1013.09
987.23
823.99
774.80
749.45
731.03
612.40
3084.52
3008.86
2938.76
2889.20
2830.55

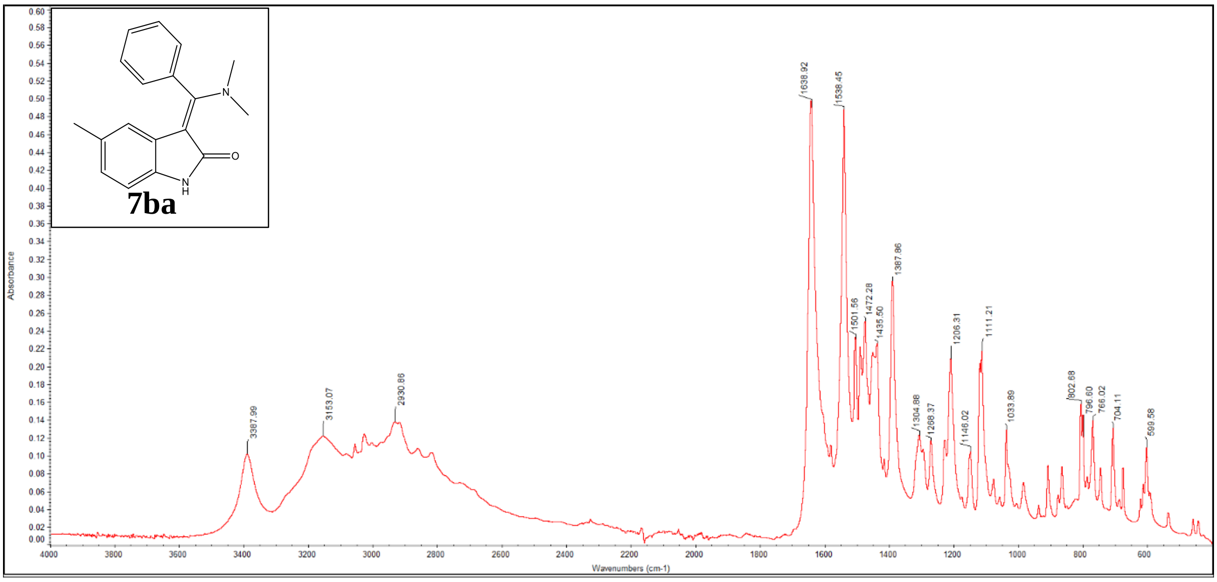
7ba
Absorbance
Wavenumbers (cm-1)
1638.92
1538.45
1501.56
1472.28
1435.50
1387.96
1304.88
1268.37
1206.31
1146.02
1111.21
1033.89
802.68
796.60
766.02
704.11
599.58
3387.99
3153.07
2930.86

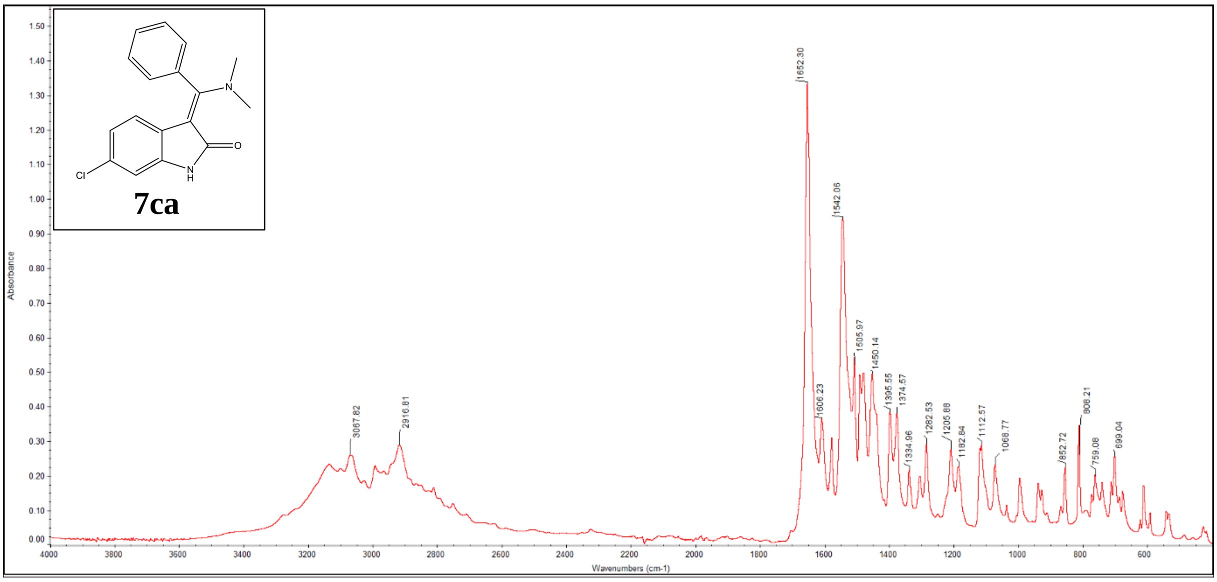
7ca
Absorbance
Wavenumbers (cm-1)

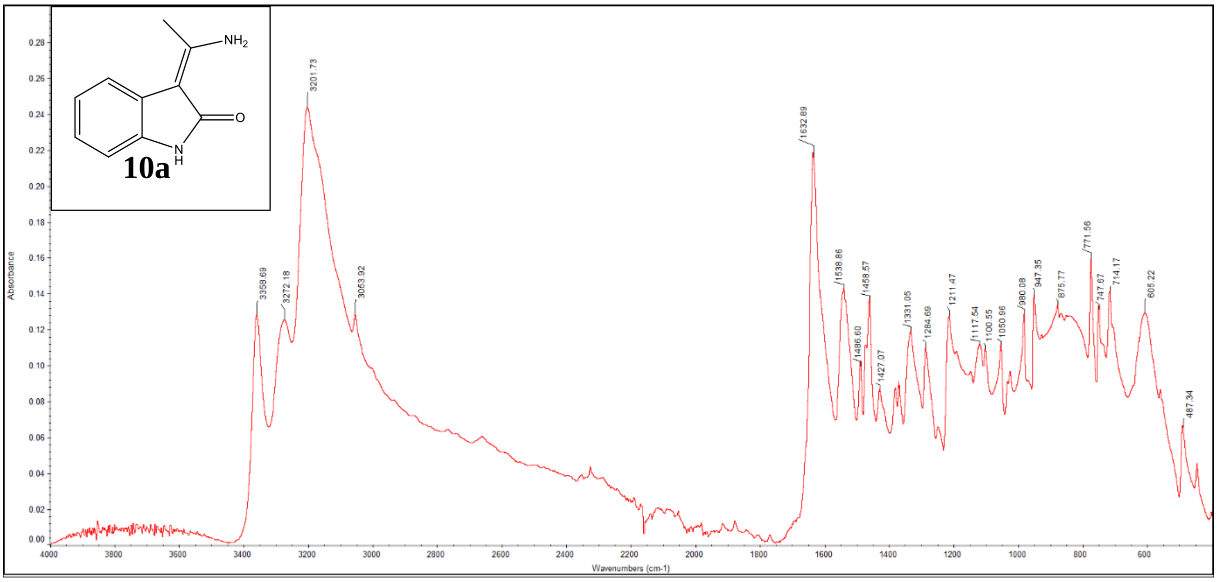
10a
Absorbance
Wavenumbers (cm-1)

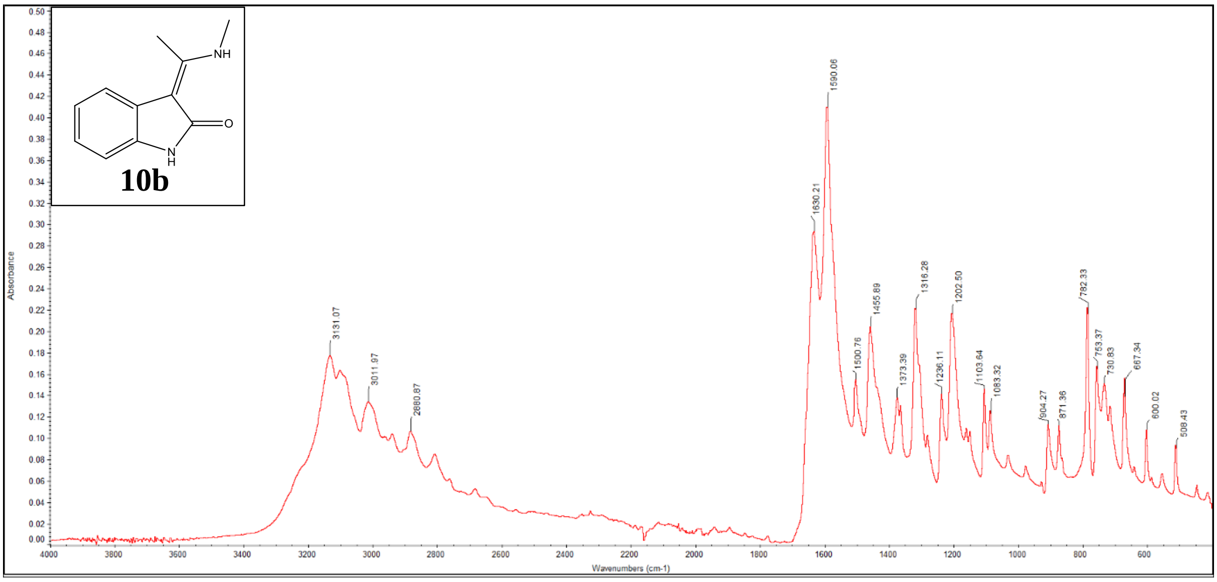
10b
Absorbance
Wavenumbers (cm-1)

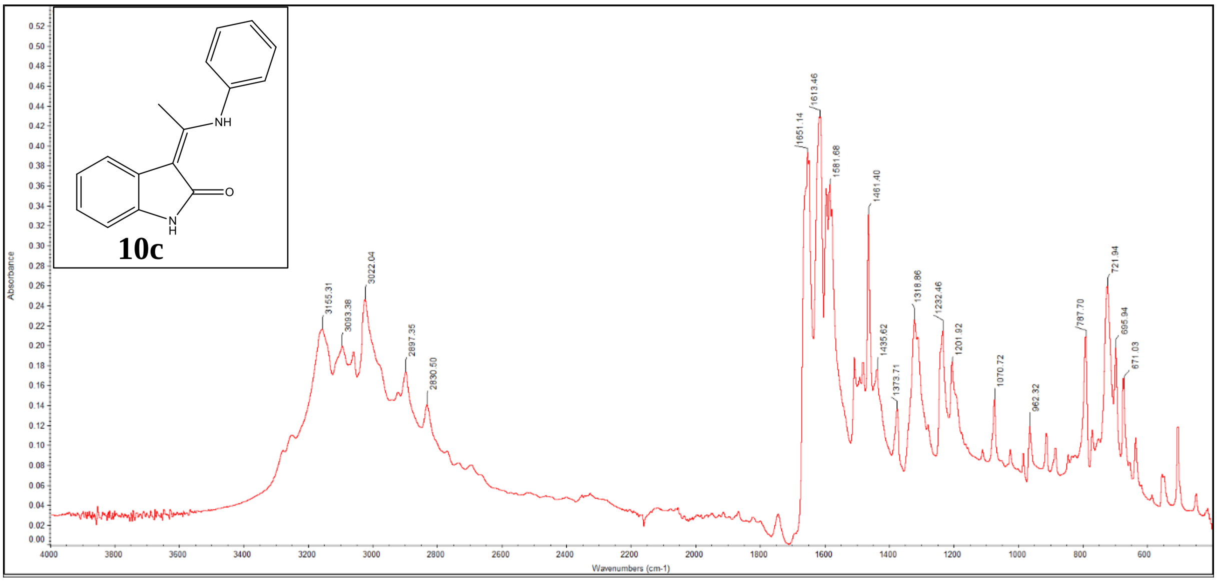

References

1. Kammel, R.; Tarabová, D.; Brož, B.; Hladíková, V.; Hanusek, J. *Tetrahedron* **2017**, *73*, 1861–1866.
2. Muzalevskiy, V. M.; Balenkova, E. S.; Shastin, A. V.; Magerramov, A. M.; Shikhaliev N. G.; Nenajdenko, V. G. *Russ. Chem. Bull.* **2011**, *60*, 2343–2346.
3. Varano, F.; Catarzi, D.; Colotta, V.; Calabri, F. R. Lenzi, O.; Filacchioni, G.; Galli, A.; Costagli, C.; Deflorian, F.; Moro, S. *Bioorg. Med. Chem.* **2005**, *13*, 5536–5549.
4. Ma, B.; Wu, P.; Wang, X.; Wang, Z.; Lin, H.-X.; Dai, H.-X. *Angew. Chem. Int. Ed.* **2019**, *58*, 13335 – 13339.
5. Trost, B. M.; Hirano, K. *Org. Lett.* **2012**, *14*, 2446–2449.
6. Bergman, J.; Stensland, B. *J. Heterocycl. Chem.* **2014**, *51*, 1–10.
7. Guillaumel, J.; Demerseman, P.; Clavel, J.-M.; Royer, R.; Platzer, N.; Brevard, C. *Tetrahedron* **1980**, *36*, 2459–2465.
8. Rokade, B. V.; Guiry P. J. *J. Org. Chem.* **2020**, *85*, 6172–6180.
9. Kammel, R.; Tarabová, D.; Růžičková, Z.; Hanusek, J. *Tetrahedron Lett.* **2015**, *56*, 2548–2550.
10. Wenkert, E.; Bernstein, B. S.; Udelhofen, J. H. *J. Am. Chem. Soc.* **1958**, *80*, 4899 – 4903.
11. Bisht, G. S.; Gnanaprakasam, B. *J. Org. Chem.* **2019**, *84*, 13516-13527.
12. Reisch, J.; Ossenkop, W. F. *Chem. Ber.* **1973**, *106*, 2070–2071.
13. Otomasu, H.; Yoshida, K.; Natori, K. *Chem. Pharm. Bull.* **1975**, *23*, 1436–1439.
